# HMC3 revealed: how much do these “Microglia” really tell us?

**DOI:** 10.3389/fimmu.2026.1778798

**Published:** 2026-06-03

**Authors:** Lucia Buccarello, Anna Privitera, Konstantinos Partsinevelos, Karolina Serwa, Giuseppe Carota, Lucia Di Pietro, Vincenzo Cardaci, Renata Mangione, Jay Sibbitts, Giuseppe Lazzarino, Angela Maria Amorini, Francesco Bellia, Valentina Di Pietro, Romana Jarosova, Fabio Di Domenico, Barbara Tavazzi, Emiliano Maiani, Giacomo Lazzarino, Giuseppe Caruso

**Affiliations:** 1 Departmental Faculty of Medicine, UniCamillus—Saint Camillus International University of Health and Medical Sciences, Rome, Italy; 2 Research Unit of Biology and Preclinical Research, IRCCS San Camillo Hospital, Venice, Italy; 3 Department of Drug and Health Sciences, University of Catania, Catania, Italy; 4 Department of Biomedical and Biotechnological Sciences, University of Catania, Catania, Italy; 5 Department of Pharmacy, Jagiellonian University, Krakow, Poland; 6 Assisted Residential Community (CRA) Mirabilis, Fondazione Mantovani, Arconate, MI, Italy; 7 Department of Physical Sciences, Truman State University, Kirksville, MO, United States; 8 Advanced Technologies Laboratory (LTA) Biotech S.r.l., Catania, Italy; 9 Department of Inflammation and Ageing, University of Birmingham, Birmingham, United Kingdom; 10 Department of Chemistry, Colorado State University, Fort Collins, CO, United States; 11 Department of Biochemical Sciences, Sapienza University of Rome, Rome, Italy

**Keywords:** HMC3 cell line, microglia, neurodegeneration, neuroinflammation, neuroprotective strategies, oxidative stress

## Abstract

Microglia are a key driver of neurodegenerative disease, orchestrating inflammatory signaling, metabolic stress responses, synaptic remodeling, and neuronal fate within the central nervous system (CNS). Among experimental models, the human microglial cell line, HMC3, is one of the most widely used models for mechanistic investigation and pharmacological screening of microglial dysfunction, particularly in neurodegenerative contexts. Nevertheless, a key question remains: how faithfully does HMC3 reflect human microglial biology? This review integrates current evidence on HMC3 cells, including their molecular and metabolic features, functional plasticity, and disease-oriented applications. HMC3 cells reproduce hallmark neurodegeneration-associated programs, such as stimulus-dependent polarization, oxidative and endoplasmic reticulum stress signaling, inflammasome activation, autophagy dysregulation, lipid remodeling, angiogenic cross-talk, and phagocytic clearance of amyloid and apoptotic debris, modeling processes relevant to Alzheimer’s disease, Parkinson’s disease, ischemic injury, and metabolic neurodegeneration. Neuron-microglia co-culture systems further demonstrate the direct impacts of HMC3 activation states on neuronal vulnerability and survival. We also summarize the expanding repertoire of pharmacological and genetic interventions applied to HMC3, highlighting their compatibility with high-throughput and multi-omics discovery platforms. Despite inherent limitations of immortalized models, HMC3 represents a powerful front-line tool for dissecting neurodegenerative microglial mechanisms and steering early therapeutic discovery.

## 1 Reframing brain function: microglia as the lens on neurons

The concept of synaptic communication was established at the end of the 19th century with Charles Sherrington’s coining of the term ‘synapse’ in 1897 and his foundational theoretical work on neural integration. These concepts were subsequently supported and extended in the 1920s–1930s by Edgar Adrian’s pioneering electrophysiological recordings, which quantitatively characterized neuronal electrical activity and provided experimental validation of synapses as key sites of information transfer in the nervous system. These advances provided experimental support for what became known as the “neuron doctrine”, largely conceptualized through the work of Santiago Ramón y Cajal, which defined the central nervous system (CNS) as an assembly of distinct, autonomous cells functioning as independent structural, genetic, and trophic units. Ramón y Cajal’s anatomical studies demonstrated that the neuronal soma and its processes represent an independent functional entity, thereby consolidating the notion that neurons are the sole electrically excitable and information-processing units of the CNS, receiving inputs through dendrites and the soma, and transmitting signals along axons to synaptic terminals.

This framework established a long-standing, strictly neuron-centric interpretation of brain function, in which synapses and neuronal circuits were regarded as the exclusive substrates of cognition, behavior, and neuropathology, while glial cells were erroneously sidelined, commonly described as passive “supportive” or “cementing” elements, reflecting the entrenched view that their role was ancillary to neuronal activity rather than mechanistically relevant to CNS function.

Over the past several decades, this classical neuron-centered paradigm has undergone a fundamental transformation. Converging evidence from molecular biology, functional imaging, and single-cell profiling has revealed that CNS physiology is governed not solely by neuronal networks but also by continuous, reciprocal communication between neurons and glial cells. As a result, astrocytes, oligodendrocytes, and microglia are now deservedly recognized as active determinants rather than passive supporters of neural function, positioning glia as central regulators of development, plasticity, metabolic homeostasis, and disease.

Astrocytes and oligodendrocytes, the two most abundant macroglial populations, originate from a common radial glial lineage and were historically viewed as electrically inert structural elements (1–3). It is now established that astrocytes exert multifunctional regulatory actions, regulating ion and water balance, extracellular pH, cerebral blood flow, synaptic remodeling, and neuroplasticity, thereby providing essential trophic and metabolic support to CNS cells (4–7). Through the formation of tripartite synapses, astrocytes directly modulate synaptic development and transmission and participate in higher cognitive processes, such as learning and memory (1).

A hallmark of astrocytes is represented by their context-dependent phenotypic plasticity, generating neurotoxic inflammatory states characterized by cytokine and chemokine release, upregulation of innate immune receptors and major histocompatibility complex class II (MHCII) molecules, and/or neuroprotective phenotypes associated with secretion of anti-inflammatory mediators, heat-shock proteins, and trophic factors (5, 8, 9).

Oligodendrocytes, generated via maturation of oligodendrocyte precursor cells (OPCs), are traditionally known for their role in axonal myelination, enabling saltatory conduction, but also provide essential energy support to neurons via the monocarboxylate transporter 1 (MCT1)-mediated lactate shuttle and regulate synaptic plasticity and long-term memory consolidation (10–13). Importantly, oligodendrocytes display immune-related phenotypic plasticity, adopting inflammatory profiles that contribute to immune-mediated CNS disorders (14, 15). Together, astrocytes and oligodendrocytes form interactive regulatory networks that shape the CNS glial microenvironment through reciprocal, paracrine signaling mechanisms essential for myelin production and network integration (16, 17). Completion of this paradigm shift has placed microglia at center stage of CNS biology. Initially described by Ramón y Cajal in 1913 as the “third element” of the nervous system, microglia are now recognized as highly dynamic cells accounting for up to 20% of the total glial population and relying on paracrine signals provided by astrocytes and oligodendrocytes to modulate their immunological activities (18, 19). These myeloid-derived specialized phagocytes represent the only innate immune cells permanently resident in the CNS.

Microglial cells are ubiquitously distributed throughout the brain and act as resident immune sentinels that continuously monitor the neural microenvironment, participate in both innate and adaptive immune responses, and protect the CNS from several toxic insults, such as injury and infection (20–23). Growing evidence, however, indicates that microglia predominantly reside in a non-inflammatory but still highly dynamic state, in which they exert crucial roles in CNS development and the maintenance of tissue homeostasis (24, 25). Although microglia are capable of mounting robust inflammatory responses to immunological challenges (26–28), their primary physiological functions appear to be developmental and homeostatic rather than immune-reactive (29). This perspective increasingly underscores that microglial activity extends beyond a solely defensive role, positioning these cells as central regulators of CNS development and maintenance through ongoing interactions with neurons and other glial populations.

### 1.1 Microglia-neural cells crosstalk in CNS homeostasis

Microglia communicate with nearly all cellular constituents of the brain, thereby regulating key processes that include neurodevelopment, tissue homeostasis, injury repair, and disease pathogenesis (24, 30–33). Through dynamic interactions with astrocytes, oligodendrocyte lineage cells, and neurons, microglia act as a central coordinator of neural function and immune surveillance.

Crosstalk between microglia and astrocytes is a major determinant of neuroinflammatory regulation within the CNS. Despite the fact that the canonical M1/M2 (microglia) and A1/A2 (astrocyte) polarization framework does not fully capture the complexity of glial activation states (34, 35), it remains a valuable conceptual framework for characterizing their functional interactions. Microglia express a broad repertoire of Toll-like receptors (TLRs) and thus serve as primary sensors of pathogens and tissue damage. Activation of pattern recognition receptors by pathogen-associated (PAMPs) or damage-associated (DAMPs) molecular patterns, such as during lipopolysaccharide (LPS) exposure or ischemic injury, drives microglia toward a pro-inflammatory M1 phenotype (36, 37). In contrast, astrocytes display limited TLR expression and therefore rely predominantly on microglial signaling for their activation (38).

**Figure 1:**
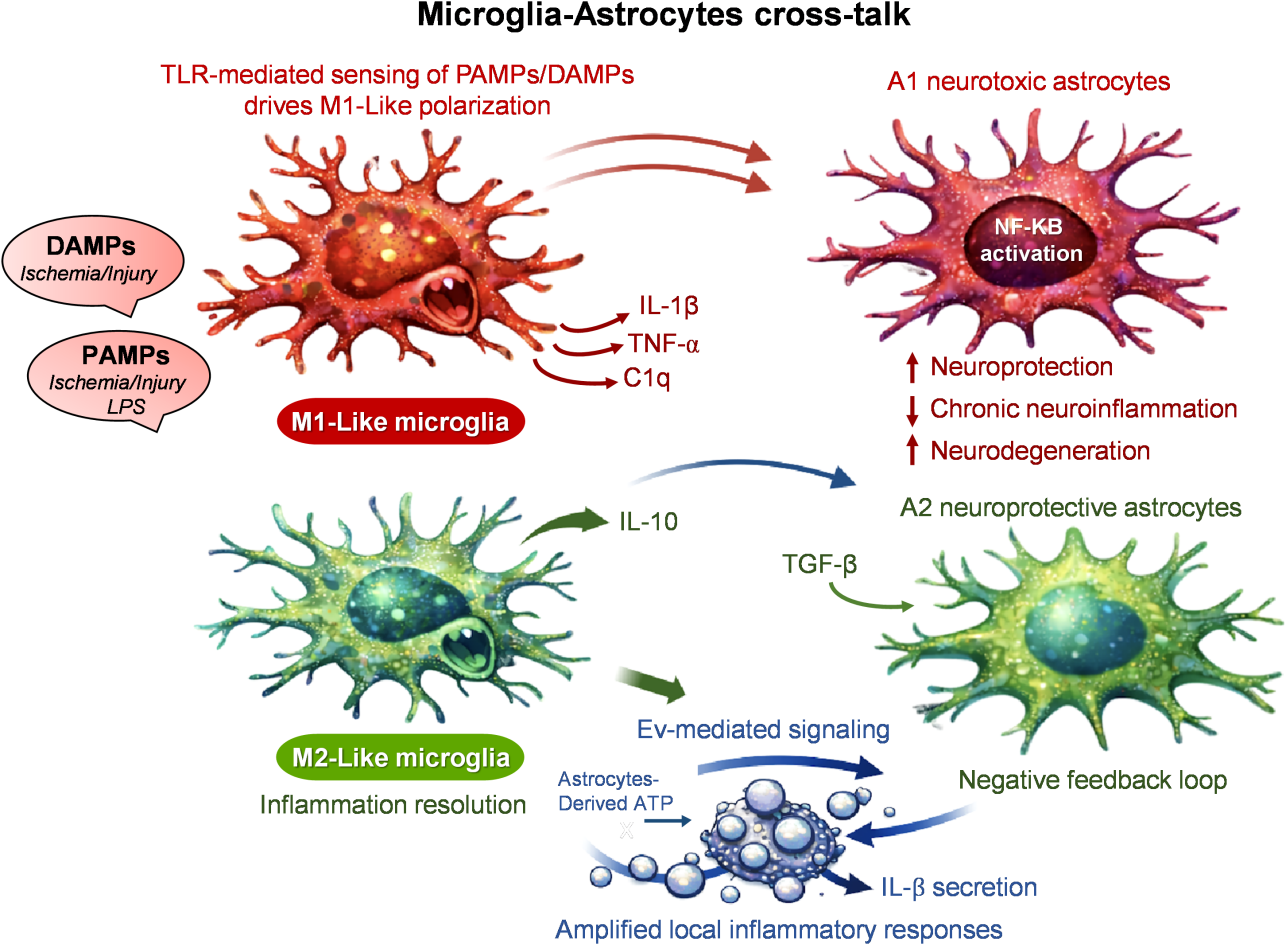
Microglia-astrocyte crosstalk in neuroinflammation and neurodegeneration. Schematic representation of bidirectional signaling between microglia and astrocytes during neuroinflammatory responses. Pathogen-associated molecular patterns (PAMPs) and damage-associated molecular patterns (DAMPs) activate microglia (left side panel) via Toll-like receptor (TLR) signaling, promoting a M1-like pro-inflammatory phenotype (labeled in red) characterized by the release of IL-1β, TNF-α, and C1q. These mediators induce A1 neurotoxic astrocytes (right side panel) through NF-κB activation, contributing to chronic neuroinflammation and neurodegeneration. Conversely, M2-like microglia (labeled in green) release anti-inflammatory cytokines (IL-10, TGF-β) and extracellular vesicles, supporting A2 neuroprotective astrocytes and inflammation resolution via negative feedback mechanisms.

Once activated, microglia shape astrocytic responses through the release of soluble mediators and engagement of nuclear factor kappa-light-chain-enhancer of activated B cells (NF-κB) signaling in astrocytes (39, 40). In particular, microglial secretion of interleukin-1α (IL-1α), tumor necrosis factor-α (TNFα), and complement component 1q (C1q) induces the neurotoxic A1 phenotype of astrocytes, characterized by loss of neuroprotective functions and the promotion of chronic neuroinflammation associated with neurodegenerative diseases progression. Conversely, anti-inflammatory M2-like microglia produce IL-10, which activates its receptor primarily expressed on A2 astrocytes and stimulates the release of transforming growth factor-β (TGF-β), establishing a negative feedback loop that dampens microglial activation (41, 42). Beyond soluble mediators, extracellular vesicles (EVs) represent an important additional communication pathway in microglia–astrocyte interactions. Astrocyte-derived ATP induces microglial EV release and IL-1β secretion, thereby amplifying local inflammatory signaling (43). Taken together, these reciprocal signaling mechanisms highlight the critical role of microglia–astrocyte crosstalk in shaping CNS immune responses and inflammatory outcomes (**Figure 1**).

**Figure 2:**
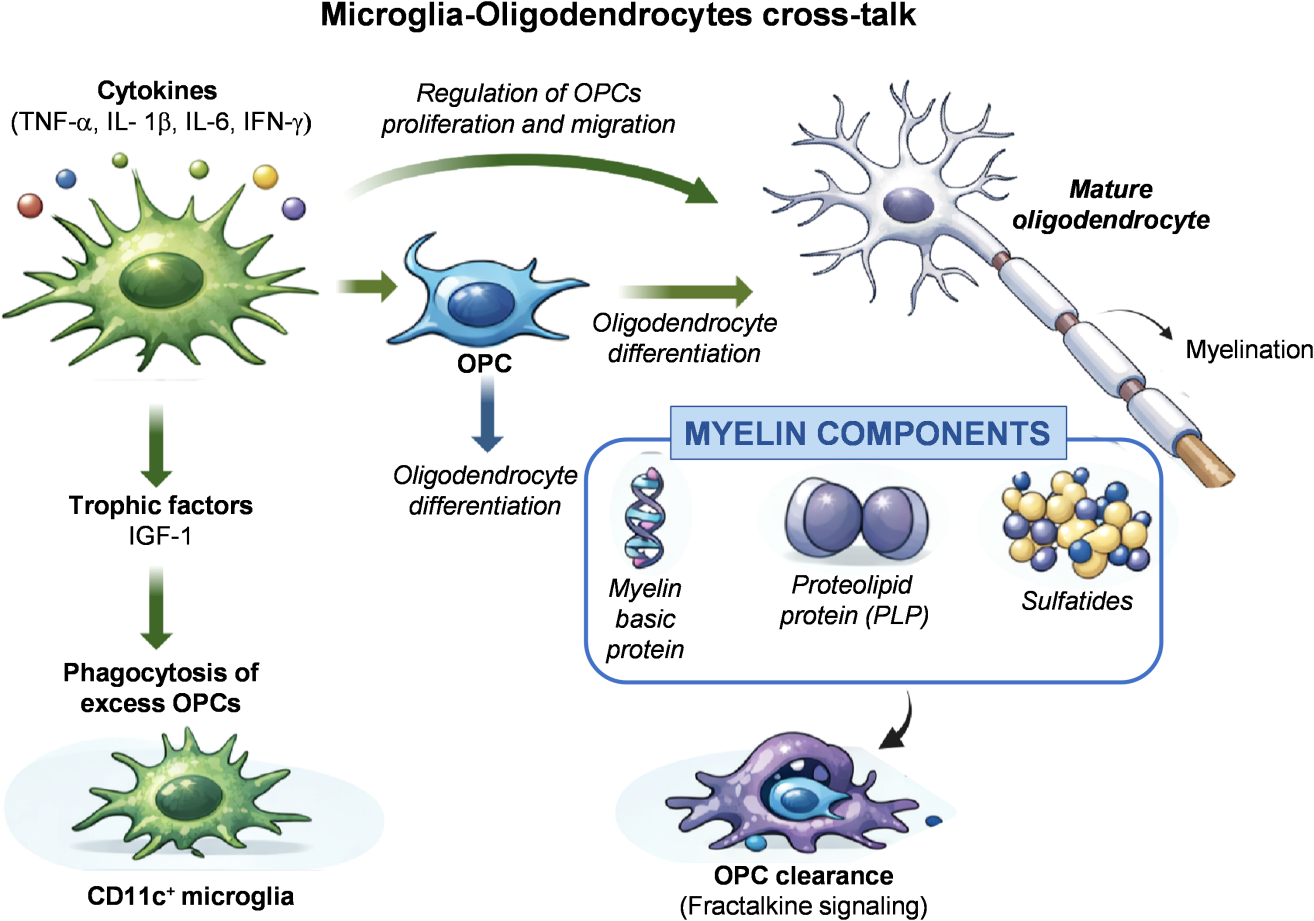
Microglia-oligodendrocyte lineage interactions in myelination. Illustration of microglia-oligodendrocyte progenitor cell (OPC) cross-talk during CNS development and repair. Microglia regulate OPC proliferation, migration, and differentiation through the release of cytokines (TNF-α, IL-1β, IL-6, IFN-γ) and trophic factors such as IGF-1. CD11c^+^ microglia contribute to OPC clearance via fractalkine signaling, ensuring appropriate myelination. Differentiated oligodendrocytes generate myelin components including myelin basic protein (MBP), proteolipid protein (PLP), and sulfatides.

Microglia also exert a fundamental influence on oligodendrocyte lineage cells and myelination. They regulate the development and homeostasis of OPCs and mature oligodendrocytes, thereby directly impacting the myelination process (44). Reactive microglia release cytokines, such as TNFα, IL-1β, IL-6, and interferon-γ (IFN-γ), that promote oligodendrocyte differentiation, whereas reduced availability of these factors results in impaired oligodendrogenesis (45, 46). A distinct CD11c^+^ microglial subset predominates in early myelinating regions of the developing brain and expresses genes involved in neuronal and glial survival, migration, and differentiation. These cells represent a major source of insulin-like growth factor-1 (IGF-1) and other trophic factors that support neurogenesis and myelin formation during critical developmental windows (46). Beyond trophic support, microglia fine-tune myelination through regulated phagocytosis of excess OPCs via fractalkine-dependent signaling. Mice lacking the fractalkine receptor show reduced microglial clearance of OPCs, resulting in increased oligodendrocyte numbers paralleled by impaired myelin thickness (47). *In vitro* co-culture studies further demonstrate that microglia stimulate oligodendrocytes to synthesize essential myelin components, including sulfatides, myelin basic protein, and myelin proteolipid protein (PLP) (48). Together, these findings underscore the multifaceted role of microglia in orchestrating oligodendrocyte maturation and ensuring proper myelin development (**Figure 2**).

**Figure 3:**
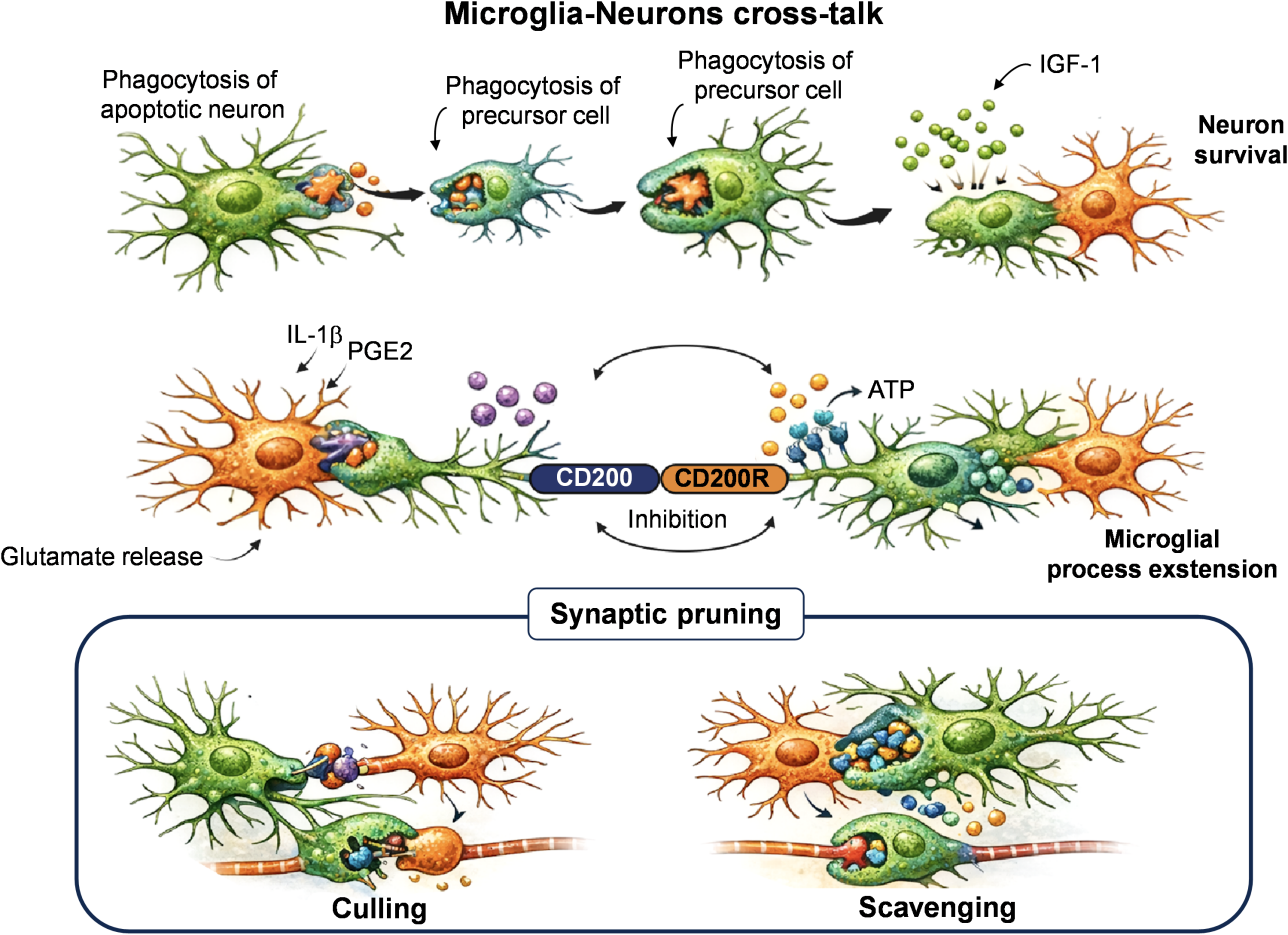
Microglia-neuron interactions in synaptic remodeling and neuronal survival. Schematic overview of microglial roles in neuronal homeostasis. Microglia phagocytose apoptotic neurons and precursor cells, regulating synaptic pruning through culling and scavenging mechanisms. Neuron-microglia inhibitory signaling via CD200-CD200R limits excessive activation, while ATP release promotes microglial process extension. Microglia-derived IGF-1 supports neuronal survival, whereas IL-1β and PGE2 modulate neurotransmitter release and synaptic activity.

Among neural interactions, microglia-neuron crosstalk has been the most intensively studied, particularly during brain development. During this period, microglia proliferate and accumulate near apoptotic neurons, acting as professional phagocytes to regulate neuronal turnover and synaptic remodeling (49). Microglia influence neurogenesis through bidirectional mechanisms: they limit cortical neuron production by phagocytosing neural precursor cells while also promoting progenitor survival, as demonstrated by the depletion of microglia that has been associated to the reduction of basal progenitor populations within the cerebral cortex (50, 51). Microglia-derived IGF-1 supports neuronal survival, whereas microglial signaling prevents neuronal hyperexcitability (52). Additionally, IL-1β released by microglia enhances presynaptic glutamate release via N-methyl-D-aspartate (NMDA) receptor-dependent synthesis of arachidonic acid and prostaglandins (53). Bidirectional neuron–microglia communication further modulates synaptic plasticity. Neuronal CD200 binds to microglial CD200 receptor (CD200R), restraining microglial activation and contributing to synaptic regulation (54, 55). Neuronal activity also induces microglial process extension through NMDA receptor-dependent ATP release (56), which in turn activates microglial P2Y12 receptors (P2RY12) and directs their dynamic interactions with synapses (57). Recent work has expanded this framework by describing microglial involvement in active synaptic pruning, whereby microglia sculpt neural circuits through targeted elimination of synapses. Two complementary mechanisms have been proposed: “culling”, in which microglia employ a contractile mechanism to sever neuronal membranes and remove unwanted synapses, and “scavenging,” a neuron-driven process in which synapses are shed by neuronal membrane fission and subsequently phagocytosed by microglia (58). These findings highlight the essential contribution of microglia to circuit refinement and functional connectivity (**Figure 3**). Overall, the above-described discoveries have reframed the view of the brain from a neuron-centric signaling network to a glia-centered, multicellular interactive system. Within this framework, microglia emerge as pivotal integrators of immune signaling, synaptic remodeling, and circuit homeostasis, thereby exerting a direct and profound influence on neural development, physiological function, and disease progression.

### 1.2 Microglial ontogeny and brain colonization

Although their ontogeny was historically controversial, first attributed to a mesodermal lineage (59) and later reinterpreted through alternative hypotheses (60), definitive studies have established their derivation from yolk sac erythromyeloid progenitors, confirming microglia as a self-renewing CNS immune population. Indeed, recent studies have revealed species-specific differences in microglial ontogeny: in both mice and zebrafish, microglia originate from macrophage populations generated during primitive hematopoiesis in the yolk sac, which begin invading the neuroepithelium around embryonic day 8.5 (E8.5) (61–64). In humans, microglial precursors colonize the developing brain primordium at approximately 4.5–5.5 gestational weeks (65, 66).

In rodents, hematopoiesis occurs in at least three distinct waves: the first, or “primitive,” wave generates primary erythromyeloid precursors (EMPs) (67). These yolk sac-derived primary EMPs give rise to yolk sac macrophages that subsequently differentiate into parenchymal microglia, non-parenchymal CNS macrophages, and peripheral tissue macrophages (68). Primary EMPs express colony-stimulating factor 1 receptor (CSF1R), which is required for their survival and differentiation (63). The second, or transient, wave produces secondary EMPs that lack CSF1R expression and depend on c-Myb gene for development (69). The third, or definitive, wave generates immature hematopoietic stem cells from hemogenic endothelium in the para-aortic splanchnopleure region (67).

**Figure 4:**
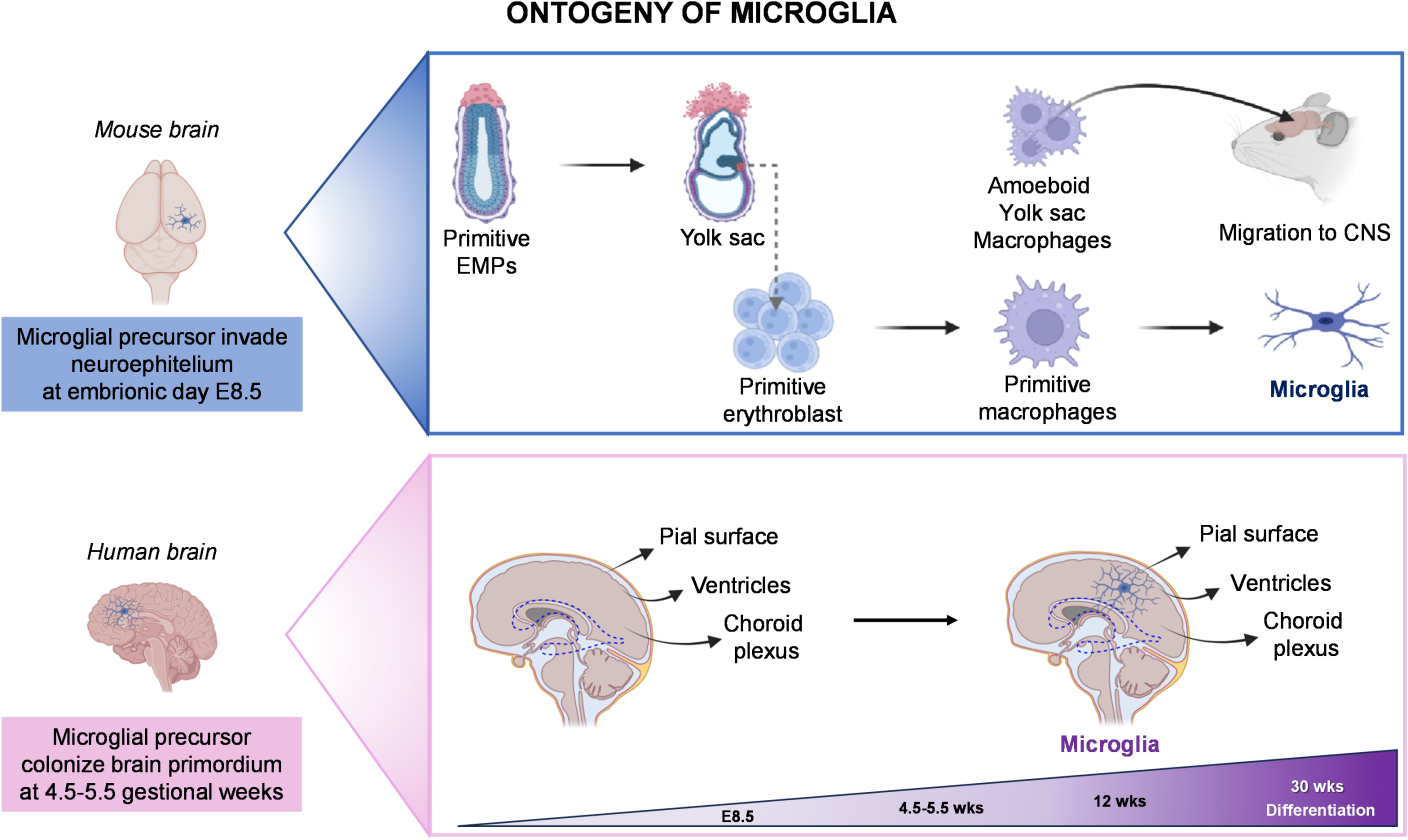
Ontogeny and colonization of microglia in mouse and human brains. Developmental origin of microglia from yolk sac-derived erythromyeloid progenitors (EMPs). In mice, microglial precursors invade the neuroepithelium at embryonic day E8.5 and differentiate into resident microglia. In humans, colonization of the brain primordium occurs at 4.5-5.5 gestational weeks, with subsequent differentiation during fetal development. Routes of entry include the pial surface, ventricles, and choroid plexus.

In humans, microglia invade the cortex through multiple entry routes, including the pial surface, ventricles, and choroid plexus, beginning at approximately 4.5 gestational weeks, followed by a secondary vascular wave targeting the white matter at 12–13 weeks. Once within the brain parenchyma, microglial precursors receive CNS-derived instructive cues that promote their maturation from amoeboid macrophage-like cells into ramified surveillant microglia. Throughout life, microglial populations are maintained predominantly through self-renewal with minimal contribution from endogenous CNS progenitors. Proliferation rates remain low (∼0.7% in mice and ∼2% in humans), with region-specific turnover: complete renewal of mouse microglia occurs over months to years depending on the brain region, whereas human cortical microglia exhibit an average lifespan of approximately 4.2 years. Homeostasis is achieved by balancing proliferation and apoptosis, with stochastic renewal that can switch to clonal expansion under pathological conditions (33, 70–72) (**Figure 4**).

### 1.3 Microglial morphology as a readout of functional state

Microglia should be considered highly dynamic and plastic cells that display coexisting and interchangeable phenotypes in response to environmental stimuli, functioning as “multitasking” cells capable of continuously adapting to local tissue demands. Using several murine *in vitro* models, microglia were historically classified into two opposing types based on experimental findings: pro-inflammatory and neurotoxic (M1), versus anti-inflammatory and neuroprotective (M2) (34, 73). However, this oversimplified classification does not reflect the true complexity of microglial responses in physiological and pathological settings, neglecting the highly dynamic nature of microglia even in their so-called “resting state”, which stems from their exceptional plasticity. Reactive microglia, defined as cells undergoing morphological, molecular, and functional remodeling in response to brain challenges, such as amyloid-β (Aβ) or α-synuclein (α-Syn) deposition, or interaction with infected, damaged or degenerating neurons, have been described across multiple neurological conditions (31, 32, 74–76). Recent evidence has demonstrated a poor correspondence between reactive microglia and the canonical M1/M2 framework in neurodegenerative diseases (77–79). In this context, single-cell technologies, multi-omics approaches, and integrative analyses of gene and protein expression have not only enabled a more precise mapping of microglial phenotypes but have also provided insights into the molecular mechanisms shaping specific cell states across developmental stages, disease conditions, and injury models.

For example, single-cell RNA sequencing (scRNA-seq) studies have identified a disease-associated microglia (DAM) state in mouse models and human Alzheimer’s disease (AD) specimens (78–81). DAMs localize near Aβ plaques and contribute to Aβ clearance (80), while distinct Aβ- and tau-associated microglial signatures have also been reported in AD patients (81). Additional context-dependent phenotypes have been reported in neurodegeneration, including activated response microglia (ARMs), interferon-responsive microglia (IRMs) (77, 82–84), and human AD microglia (HAMs) (79), as well as in amyotrophic lateral sclerosis (ALS) and Parkinson’s disease (PD) (77, 85, 86), inflammatory disorders such as multiple sclerosis (MIMS) (87, 88), aging (lipid-droplet-accumulating microglia, LDAMs) (89, 90), and cancer (glioma-associated microglia, GAMs) (91, 92). During development and aging, specialized populations such as white matter–associated microglia (WAMs), axon tract-associated microglia (ATMs), and proliferative region-associated microglia (PAMs) appear, sharing features of a core DAM-like program linked to phagocytosis and tissue remodeling (93, 94). Similar DAM-related transcriptional programs have been detected in the developing human CNS, suggesting partially conserved multifunctional responses across conditions (95). Moreover, scRNA-seq studies have revealed a strong association between altered levels of secreted phosphoprotein 1 (SPP1) and macrophage populations within DAM, suggesting a possible SPP1 role as microglial stimulators in both inflammation and neurodegenerative processes (77, 96–98). The SPP1 contribution in microglial activation is also described in age-related macular degeneration, highlighting as retinal microglia activated by SPP1 induced synaptic loss and photoreceptors death, neuroinflammation, and pro-angiogenesis microenvironment formation (99). Although SPP1 is a DAM signature gene upregulated in human microglia during the aging and neurological disorders, only a single recent study has employed the HMC3 cell line to model DAM-like features *in vitro* (100) underling the necessity to further investigate the mechanisms involved in SPP1-microglial activation. Nonetheless, defining microglial states solely based on gene or protein expression is limited, as mRNA levels do not reliably predict protein abundance, and neither ramification measurements alone accurately reflect cell function. Although multiple classification strategies, including transcriptomics, proteomics, morphology, and epigenetic profiling are available, their variable coverage has contributed to inconsistent labeling and conceptual confusion. While distinct microglial states are thought to correspond to specialized functions, many of these roles remain incompletely understood.

**Figure 5:**
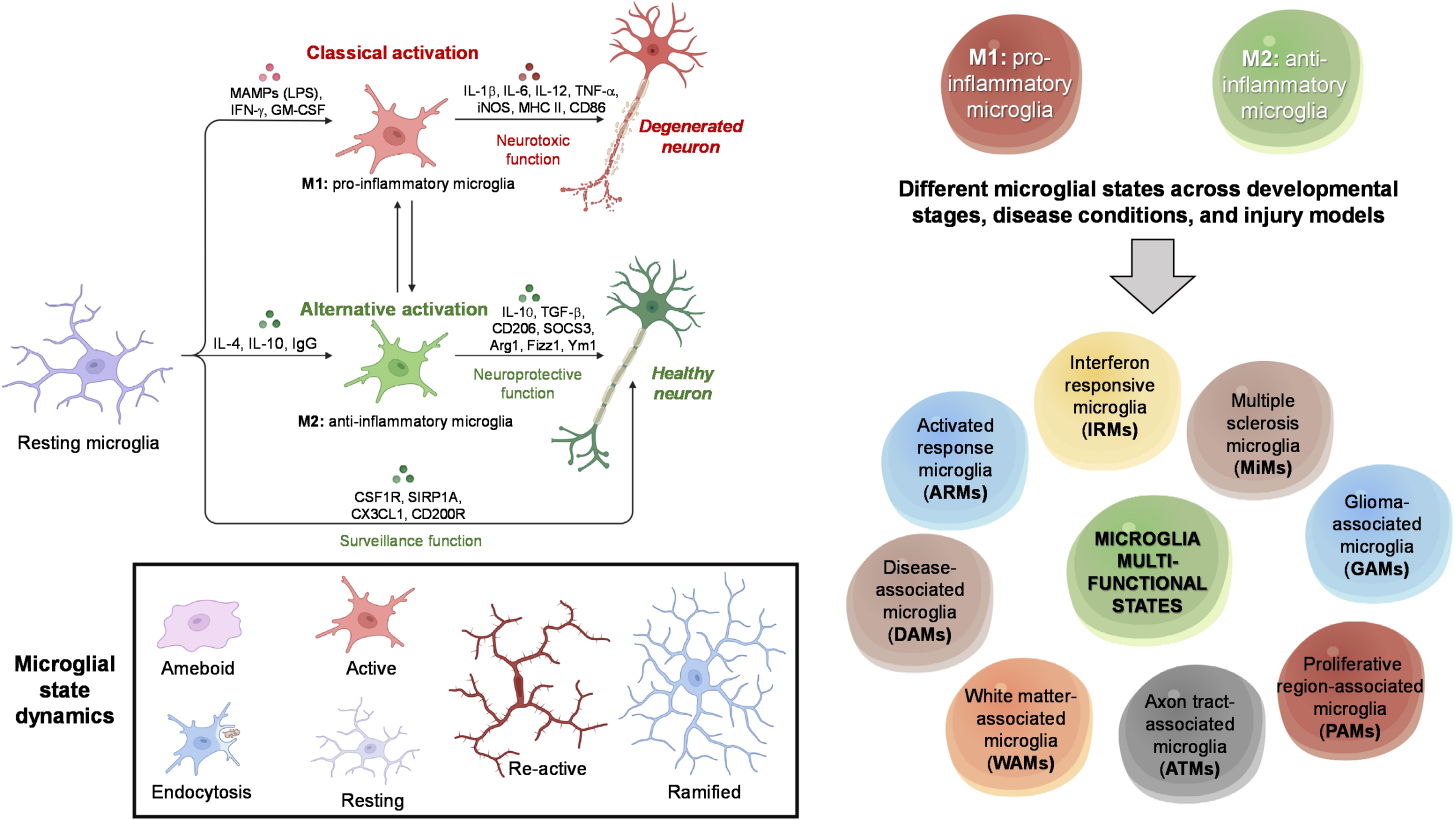
Microglial activation states and functional heterogeneity. Overview of microglial phenotypic diversity across homeostasis, development, and disease. Classical (M1-like) activation induced by MAMPs and IFN-γ is associated with pro-inflammatory mediators release and/or expression (IL-1β, IL-6, TNF-α, iNOS), whereas alternative (M2-like) activation driven by IL-4 and IL-10 promotes neuroprotective functions (Arg1, TGF-β, CD206). The figure highlights dynamic microglial states, including disease-associated microglia (DAMs), interferon-responsive microglia (IRMs), activated response microglia (ARMs), and other specialized subtypes observed in development and pathology.

As extensively discussed by Paolicelli et al. (66), substantial efforts have been made to establish a more accurate nomenclature for microglial state dynamics, moving beyond the traditional M1/M2 paradigm. The following recommendations have been proposed: I) microglia are highly dynamic, plastic cells with diverse structural, molecular, metabolic, and functional states in health and disease; II) their characterization should integrate multiple layers of complexity, including ontogeny, morphology, motility, multi-omics profiles, and functional readouts, within a defined species and spatiotemporal context; III) the term “homeostatic” should replace “resting,” while “surveillant” should be reserved to describe active surveillance behavior; IV) in experimental settings, microglia should be described as “reactive to” or “responding to” specific stimuli rather than broadly “activated”; V) simplistic dichotomies (e.g., M1/M2 or pro- vs. anti-inflammatory) should be avoided, with results always interpreted within their biological context, and VI) the term “DAM” should be used cautiously, as it may not be universally applicable across diseases or models, particularly in the human brain (**Figure 5**).

Notably, most concepts and classifications discussed above are derived primarily from studies of murine microglia. Despite shared core signatures, murine and human microglia differ significantly in transcriptional programs, activation dynamics, metabolic regulation, and disease-associated responses, with human microglia exhibiting greater heterogeneity and reduced polarization compared to their murine counterparts (101–103). Consequently, careful validation in human systems is essential to ensure accurate translation of microglial biology and nomenclature to human disease contexts.

### 1.4 Bridging the gap: from murine microglia to the HMC3 human cell line

#### 1.4.1 *In vitro*: murine microglial models

As previously mentioned, microglia play different essential roles in maintaining CNS homeostasis and regulating immune responses, and their dysfunction contributes to a wide spectrum of neurological disorders, including neurodegenerative diseases such as AD and PD, as well as malignancies such as glioblastoma (GBM) (104, 105). To investigate microglial biology and disease mechanisms, a variety of *in vitro* models have been developed, including primary microglial cultures, immortalized cell lines, and induced pluripotent stem cell (iPSC)-derived microglia. Each system offers distinct advantages and limitations with respect to accessibility, cost, scalability, and fidelity to the *in vivo* human microglial phenotype (106). Immortalized microglial cell lines derived from mouse, rat, and human tissues represent widely employed experimental tools. These lines are typically generated through oncogenic transformation of primary cultures using viral oncogenes such as v-myc, v-raf, v-mil, or SV40 large T antigen. While they provide practical benefits including ease of culture, long-term propagation, and suitability for high-throughput screening, immortalization is associated with phenotypic drift, altered differentiation states, and genetic and functional discrepancies compared with primary or “freshly” isolated microglia (107, 108). Moreover, many lines are derived from embryonic or neonatal tissues and may therefore not recapitulate adult microglial identity. Nonetheless, these models remain valuable for biochemical, molecular, and pharmacological studies requiring large cell numbers.

The first murine microglial cell lines were generated by Righi and colleagues in 1989 (109) from embryonic mouse brain cultures immortalized using retroviral vectors encoding v-myc or v-mil. The resulting lines, designated as N3, N9, N11, and N13, display microglial hallmarks including rapid proliferation, growth factor independence, and adaptability to serum-free media. They express microglial markers such as F4/80, Fc receptors, and macrophage-1 antigen (Mac-1), while lacking markers of astrocytes, oligodendrocytes, glial precursors, and lymphocytes. Among these, N9 cells are particularly well characterized; they secrete pro-inflammatory cytokines following LPS stimulation and efficiently phagocytose Aβ fibrils (110).

In 1990, Blasi et al. developed the BV2 microglial cell line (111), now among the most widely used murine microglial models alongside N9 cells. BV2 cells were derived from neonatal mouse microglia immortalized using a v-raf/v-myc retroviral construct. They express macrophage/microglial markers, including Mac-1 and Mac-2 (galectin-3), and lack astrocytic (glial fibrillary acidic protein, GFAP) and oligodendrocytic (galactosylceramidase, GalC) markers, supporting their application in neuroinflammation research (112, 113). BV2 cells exhibit an intrinsically activated phenotype and respond robustly to inflammatory stimuli such as LPS, show enhanced phagocytosis, increased production of reactive oxygen and nitrogen species (ROS and RNS, respectively), and induction of pro-inflammatory gene programs following exposure to Aβ or α-Syn. Consequently, they are extensively used in studies of neurodegenerative disease mechanisms (32, 76, 110, 114–116).

Additional microglial lines derived from neonatal mouse cultures include the C8-B4 model developed by Alliot et al. (117). C8-B4 cells express classical microglial markers (Mac-1, F4/80, 2-4G2) and uniquely display the CD4 surface glycoprotein, which can enhance interactions with CD4^+^ T helper lymphocytes. Functionally, this line has a unique signature represented by its ability to produce and release high levels of glutamate. Walker et al. established a group of immortalized microglial lines known collectively as the EOC series (118), which differ in expression of B7-2 (CD86), F4/80, Ly-6C, and MHCII. These sub-lines, renamed EOC-2, EOC-13.31, and EOC-20, share the capacity to secrete cytokines and reactive species including nitric oxide (NO), an RNS implicated in Aβ-induced neurotoxicity in AD (119, 120). EOC-20 cells are widely used to investigate regulation of NO production, including studies demonstrating that TNFα markedly enhances nitrite release, an effect inhibited by lanthionine ketimine (120). Notably, EOC-2 cells do not express MHCII, that can instead be induced by IFN-γ in EOC-20 (but not in EOC-13.31 cells).

The MG5 microglial cell line, developed by Ohsawa et al. from p53-deficient neonatal mice (121), represents a model closely preserving the phenotypic features of primary microglia, as demonstrated by the expression of typical microglial markers, such as Mac-1, F4/80, and ionized calcium-binding adapter molecule 1 (Iba1), while also retaining morphological, biochemical, and physiological properties consistent with primary microglial populations, enhancing their utility in the context of functional studies. Using this platform, Iwamaru and colleagues developed the MG20 line from neonatal tga20 mice overexpressing the murine prion protein (122). MG20 cells exhibit a chronically activated microglial phenotype and are employed in studies of neurotoxicity and sustained neuroinflammation.

An intermediate phenotype between MG5 and MG20 is represented by the MG6 cell line, described by Takenouchi et al. (123). MG6 cells express functional P2X7 receptors, ATP-gated ion channels critical for microglial activation signaling. These cells display a surveillant or “primed” phenotype with phagocytic activity that more closely resembles that of primary microglia compared to highly activated cell lines such as MG20.

Two additional neonatal mouse-derived cell lines, RA2 and SIM-A9 (124, 125), model distinct states of microglial activation. RA2 cells exhibit a primed pro-inflammatory signature characterized by elevated basal cytokine levels and inducible NO synthase (iNOS) expression together with strong immune responsiveness, making them suitable for modeling chronic inflammatory conditions. In contrast, SIM-A9 cells present a more homeostatic, resting-like profile with low basal inflammatory activity and high inducibility, offering a physiologically relevant system for examining transitions between surveillant and activated states.

Microglial cell lines have also been generated from adult mouse brains as in the case of the immortalized microglia (IMG) line. IMG cells express CD11b and F4/80 and lack markers of neurons and astrocytes. Compared to BV2 cells, they display more robust inflammatory and anti-inflammatory responsiveness, including strong induction of both iNOS and arginase-1, and efficiently phagocytose Sβ oligomers, highlighting their utility for a more accurate modeling of adult microglial responses (126).

Another important category of murine microglial *in vitro* models is represented by spontaneously immortalized cell lines, including SIM-A9, derived from neonatal mouse brain as previously mentioned, and HAPI and MLS-9 cells, derived from neonatal rat brain (127, 128). The HAPI cell line was derived from enriched primary microglial cultures prepared using the “shake-off” method (129) and exhibits classical microglial features, including high proliferative capacity, phagocytic activity, and expression of the complement receptor marker OX42 (C3R), while lacking progenitor (A2B5) and astrocytic markers. Functionally, LPS stimulation induces robust gene upregulation of pro-inflammatory mediators such as TNFα and iNOS, accompanied by substantial release of NO and TNFα. Exposure to methamphetamine further activates HAPI cells, triggering the production of cytotoxic factors including IL-1β, IL-6, TNFα, ROS, and RNS (130).

The MLS-9 microglial cell line was derived from primary rat microglial cultures initially maintained in the presence of CSF-1 and subsequently adapted to growth factor–independent proliferation, resulting in spontaneous immortalization and long-term expansion capability (128). MLS-9 cells faithfully recapitulate core features of the microglial phenotype, expressing isolectin B4, exhibiting uptake of DiI-acetylated LDL and Lucifer Yellow (indicative of endocytic and pinocytic activity), and staining positive for OX-42 and ED-1, while lacking astrocytic and fibroblastic markers. Of note, MLS-9 cells also express human Ether-à-go-go-related gene (hERG)-like potassium channels, resembling currents observed in cardiac muscle and neuronal tissues, making this line particularly valuable for studies investigating microglial ion channel physiology and signaling mechanisms *in vitro*.

**Figure 6:**
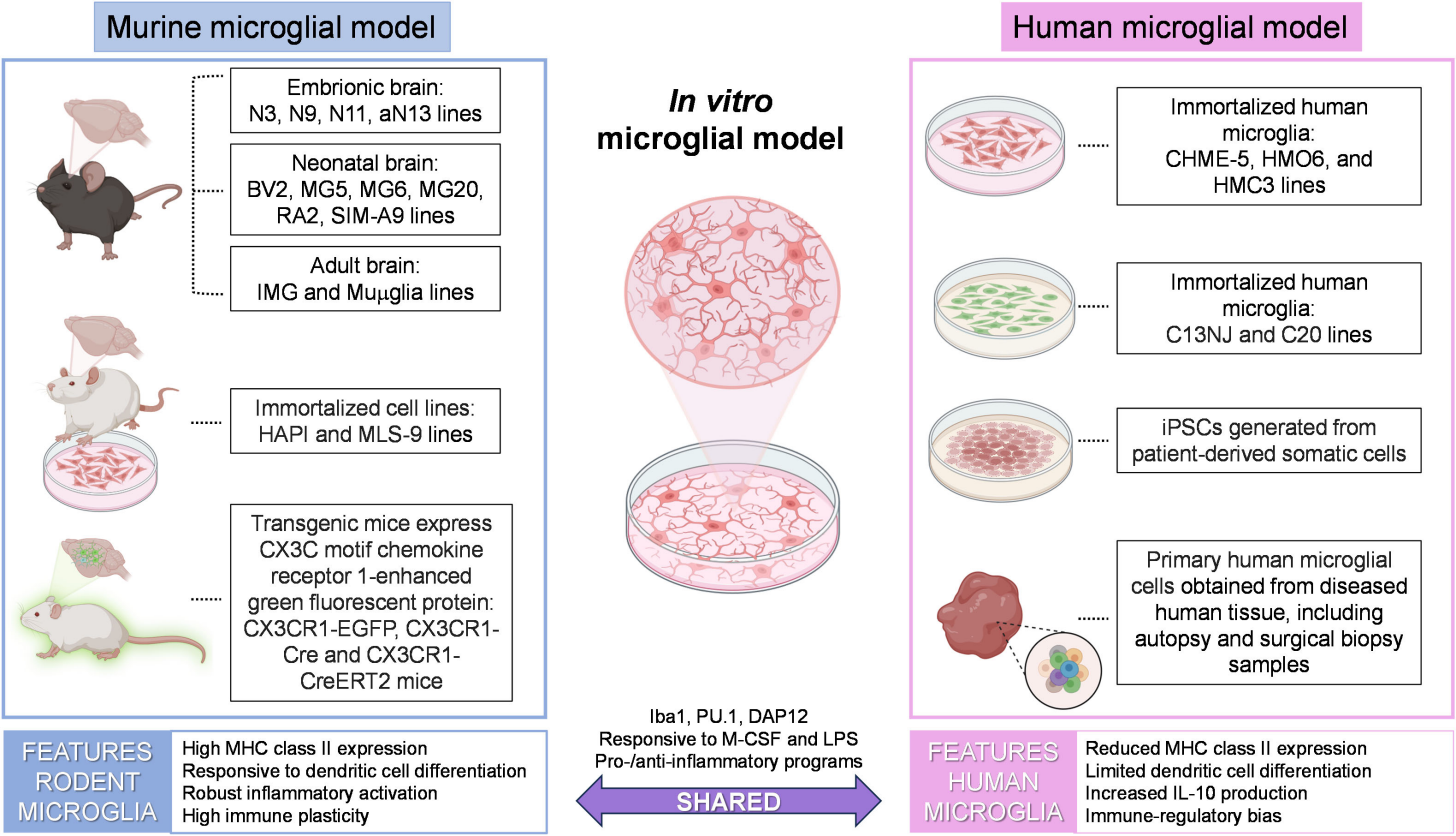
*In vitro* murine and human microglial models. Comparison of experimental microglial models used in neuroinflammation research. Murine systems include embryonic, neonatal, adult, and immortalized microglial cell lines, as well as CX3CR1 reporter and Cre-based transgenic mice. Human models comprise immortalized microglial lines, iPSC-derived microglia, and primary microglia isolated from human tissue. Shared and species-specific features are highlighted, including differences in MHC class II expression, immune plasticity, and cytokine profiles.

To better investigate the multifaceted roles of microglia, recent advances in genetic and pharmacological tools have substantially expanded the ability to visualize these cells and manipulate their function *in vivo*. Widely used approaches include C-X3-C motif chemokine receptor 1–enhanced green fluorescent protein (CX3CR1-EGFP), CX3CR1-Cre, and CX3CR1-CreERT2 transgenic mouse models, as well as microglial depletion strategies and knockout (KO) lines informed by transcriptomic discoveries (50, 131, 132). Although these systems have been instrumental in advancing our understanding of microglial biology, each one has notable limitations, including CX3CR1 haploinsufficiency, off-target expression in neurons and other macrophage populations, and unintended effects on peripheral immune cells (**Figure 6**).

Despite extensive efforts to characterize microglial dynamics using rodent isolation protocols (106, 133), species- and age-related differences in rodent-derived cultures limit their relevance to human neurodegenerative disease, highlighting the need for models that more faithfully capture the multifaceted roles of microglia in humans.

#### 1.4.2 *In vitro*: human microglial models

The use of primary microglia isolated from transgenic mice has been essential in elucidating the roles of specific genes related to microglial activation and function. However, important limitations characterize rodent primary microglial models, including their evolutionary divergence from humans, reduced genetic heterozygosity resulting from inbred strains, as well as the impact of aseptic housing conditions on immune development and responsiveness (134). Taken together, these factors contribute to interspecies differences that can limit the translational relevance of rodent models (134–136).

To overcome these limitations, different microglial human cells lines have been developed. The HMO6 cell line is an immortalized human microglial model generated by v-myc retroviral transduction of embryonic telencephalon-derived primary microglia (137). HMO6 cells retain microglial identity, expressing CD11b and ricinus communis agglutinin-1 (RCA-1) lectin, while lacking neuronal, astrocytic, and oligodendrocytic markers, and display ATP responsiveness and phagocytic capacity comparable to primary microglia. However, their inflammatory response can be considered attenuated: stimulation with LPS or Aβ_25–35_ induces only limited cytokine secretion (primarily IL-8 and TNFα) compared to the broad cytokine profile produced by primary human microglia. This reduced responsiveness may reflect transformation-related effects. Although historically representing the only human microglial cell line available, its use has been limited by patent restrictions.

Other immortalized human microglial cell lines include C13NJ and C20 (138), which both originate from the CHME-5 cell line (139). The C13NJ and C20 microglial cell lines are clonal derivatives of the CHME-5 line, originally reported as human but subsequently demonstrated to be of rat origin based on genetic profiling (140). C13NJ and C20 display similar expression of canonical microglial markers, including CD11b and Iba1, also exhibiting comparable proliferative capacity and inflammatory responsiveness to immune stimuli such as LPS and viral proteins. To date, no consistent or clearly defined functional distinctions have been established between the two lines beyond minor phenotypic variability associated with clonal selection. Immortalized microglial cell lines are widely used for their accessibility and scalability, but immortalization-driven de-differentiation and transcriptional alterations limit their ability to faithfully model primary human microglial states (106, 107, 141). To overcome these limitations, stem cell-based approaches have emerged as a complementary strategy to investigate the multifaceted roles of microglia. Stem cell technologies provide an expandable source of microglia for *in vitro* studies, primarily through the differentiation of embryonic stem cells (ESCs) and induced pluripotent stem cells (iPSCs), with the latter being particularly advantageous because they can be generated from patient-derived somatic cells, enabling disease modeling while preserving individual genetic backgrounds. Initial differentiation protocols guided stem cells through neuroectodermal lineages; however, lineage-tracing studies subsequently demonstrated that microglia originate from yolk sac–derived macrophage progenitors. This paradigm shift led to the development of improved differentiation strategies that recapitulate embryonic myelopoiesis, generating embryonic/myeloid precursors before inducing microglial maturation (142–144).

Several methods have been established to produce microglia-like cells over 4–10 weeks using defined growth factors, serum-free conditions, and, in some cases, astrocyte or neuronal co-culture (145–148). Transcriptomic analyses show that these cells closely resemble human fetal microglia, with some protocols achieving partial similarity to adult microglia (149, 150). Although iPSC-derived microglia represent the *in vitro* model that better recapitulates the expression profile of primary human microglia, these models can be expensive and time-consuming to establish (148, 151). However, variability among methods remains high, and most stem cell-derived microglia display immature phenotypes or overlap with macrophage and dendritic cell signatures, warranting caution when modeling adult microglial function, particularly in aging or neurodegenerative contexts. Moreover, iPSC reprogramming resets cellular aging signatures, which may limit disease relevance for late-onset disorders.

The last *in vitro* method used to study microglial cells that best recapitulate the human microglial function/phonotype corresponds to primary human microglial cells. Primary human microglial cultures can be established using several isolation methods (152–156) and remain a valuable model due to the absence of species differences and the ability to study microglia directly obtained from diseased human tissue, including autopsy and surgical biopsy samples. However, their practical use is limited by restricted access to human brain tissue, low cell yields, and the limited proliferative capacity *in vitro* (106).

Analyzing deeply the existing differences between human and rodent microglia, the first share many conserved features with their rodent counterparts, including expression of key markers such as Iba1, the transcription factor PU.1, and the adaptor protein DNAX-activating protein of 12 kDa (DAP12), along with similar responses to stimuli, including macrophage colony-stimulating factor (M-CSF) and LPS (157–161). Both species display comparable activation profiles consistent with classical pro/anti-inflammatory phenotypes. However, notable differences exist: adult human microglia show limited responsiveness to dendritic cell differentiation signals, reduced expression of MHC class II molecules, and increased production of the anti-inflammatory cytokine IL-10 compared with rodent microglia and human monocytes. Overall, despite a substantial overlap, many microglial functions may differ between species, and it remains unclear to what extent rodent models accurately recapitulate human microglial biology (**Figure 6**).

Despite recognized limitations regarding species origin and immortalization-associated phenotypic drift, the HMC3 cell line, described in details in the next section, remains the most widely utilized *in vitro* microglial model due to its exceptional practicality and experimental versatility (162, 163). Compared to primary microglia and iPSC-derived microglial systems, HMC3 offers unmatched accessibility, robustness in culture, rapid expansion, and compatibility with routine genetic manipulation and high-throughput screening workflows. These properties enable reproducible large-scale studies of inflammatory signaling pathways, neuroimmune interactions, and pharmacological modulation that would be technically demanding and/or financially prohibitive using primary or stem cell-derived microglia. The extensive existing literature and standardized protocols further support cross-study comparability, reinforcing HMC3 as a powerful discovery platform for mechanistic and screening applications, particularly when findings are subsequently validated in more physiologically faithful human microglial models.

## 2 HMC3 cell line

The HMC3 cell line was originally established in 1995 through the SV40-dependent immortalization of primary microglial cultures derived from 8–12-week-old human embryonic spinal cord and cortical tissues (139). As reported by Peudenier et al. (164), primary human microglia were isolated by circular shaking from mixed neural cultures and exhibited slow proliferation kinetics, reaching confluence within 8–12 days. Morphologically, these cells display heterogeneous profiles characterized by vacuolated cytoplasm and short cellular processes. Immunophenotypic analysis showed expression of myeloid lineage markers, including CD68, CD11b, and CD14, although detection of CD68 varied depending on the antibody clone used. In contrast, expression of CD4 and MHCII antigens was minimal and progressively diminished during serial cultivation. Functionally, primary microglia exhibited consistent non-specific esterase (NSE) activity and strong phagocytic capacity, as demonstrated by the internalization of zymosan particles during early culture stages.

SV40 immortalization generated multiple microglial clones, among which HMC3 was selected for its robust growth characteristics. This clone demonstrated a marked increase in proliferative capacity with doubling times of approximately 24–48 hours, while retaining most key morphological and immunophenotypic features of the primary microglial population. Notable changes associated with immortalization included an increased proportion of CD68 (EBM/11)-positive cells and a relative reduction of phagocytic activity. Antigen expression remained stable for at least 35 passages, supporting the phenotypic consistency of clones. Under basal conditions, HMC3 cells were positive for NSE, CD68, and CD11b in approximately 80-90% of cells, while remaining negative for CD14, MHCII, and CD4 (139, 162). Importantly, upon stimulation with recombinant IFN-γ, HMC3 cells markedly upregulated MHCII expression, reaching levels comparable to primary human microglia and exceeding those observed in other immortalized microglial clones. The cells consistently lacked astrocytic and neuronal lineage markers, including GFAP and neurofilament proteins, confirming their microglial identity.

From a functional standpoint, HMC3 cells secrete substantial levels of IL-6 under resting conditions, exhibiting higher baseline production than other immortalized microglial clones. Pro-inflammatory stimulation with IL-1α or LPS further increases IL-6 secretion; however, the magnitude of this response remains lower than that observed in primary human microglial cultures, consistent with partial functional attenuation associated with immortalization. In contrast, HMC3 cells exhibit a relatively attenuated inflammatory secretory profile, as the induction of detectable TNFα is minimal or often absent under basal conditions and following pro-inflammatory stimulation (133, 162, 165). The low inducibility of TNFα production, together with the loss of CD14 expression, is consistent with previously reported features of embryonic human microglia, highlighting functional differences between the HMC3 clone and adult primary microglial populations (139, 162).

Currently, only two commercially available immortalized human microglial cell lines are available: HMC3 and the SV40-immortalized human microglia distributed by Applied Biological Materials. The HMC3 line has recently been authenticated by the American Type Culture Collection (ATCC) and is now distributed as HMC3 (ATCC^®^ CRL-3304) (166, 167). While early literature searches using the terms HMC3 or HMC-3 identified relatively few directly relevant publications, broader searches incorporating the alternative designations CHME3 or CHME-3 revealed an additional body of more than 20 studies published between 1999 and 2018 employing the same cell line, as described by Dello Russo et al. (162). This reconstruction shows that human microglial clone 3 has circulated under multiple names, including HMC3, CHME3, C13-NJ, and CHME-5, across different laboratories, complicating accurate tracking of its scientific use (162). Interestingly, with the aim of more comprehensively providing information on the state of the art of these cells, Dello Russo and collaborators also provided original data in their review.

### 2.1 From C13-NJ to HMC3: establishment of a human microglial model

Since the mid-1990s, the HMC3 cell line has been extensively used to investigate both the morphological and functional properties of human microglia, as well as their pivotal roles in neurodegeneration, neuroinflammation, and tumor-associated processes, with the ultimate goal of identifying microglia-mediated neuroprotective strategies.

The human microglial HMC3 cell line was originally distributed under the name CHME-5 (168) and subsequently propagated primarily through the laboratory of Prof. Talbot (University of Quebec, Montreal). The same cell line appeared in the literature under alternative denominations, including C13-NJ (138), and was disseminated among laboratories often without formal documentation of provenance, resulting in widespread, but poorly traceable circulation. Early investigations reported that CHME-5 cells exhibited hallmark features of microglia, including characteristic morphology and immune functionality, such as constitutive IL-6 secretion, robust phagocytic activity toward latex beads, yeast, and zymosan particles, elevated oxygen consumption, and high basal production of ROS (169–173). The cells also demonstrated resistance to heat-shock stress, associated with increased expression of heat shock proteins (Hsc70 and Hsp70), metabolic remodeling, mitochondrial adaptations, and cytoskeletal reorganization (169, 174). Furthermore, co-culture with rat C6 glioma cells induced additional metabolic shifts, including reduced mitochondrial activity and oxygen consumption, decreased mitochondrial membrane potential and ATP levels, increased lactate production, cytoskeletal remodeling, enhanced phagocytic capacity, and nuclear translocation of Hsp60 (170).

Molecular characterization demonstrated the expression of functional triggering receptor expressed on myeloid cells 2 (TREM2) and receptor for advanced glycation end-products (RAGE) in CHME-5 cells (168). Challenge with glycation end-products resulted in increased ROS generation, reduced cell viability, and induction of microglial activation markers, including upregulation of the glucose transporter GLUT-5, enhanced MHCII expression, and elevated release of TNFα (175, 176), while exposure to low-energy electromagnetic fields produced no detectable effects, high-dose ionizing radiation induced oxidative stress responses, increased inflammatory marker expression, and reduced cell survival (177, 178).

The CHME-5 line became widely employed as a model for microglial viral infection, particularly in the context of human immunodeficiency virus (HIV)-1 neuropathogenesis, as the cells were shown to support efficient replication of M-tropic HIV-1 strains comparable to that observed in primary human monocytes (179). HIV-1 infection conferred resistance to apoptosis induced by LPS and cycloheximide, an effect mediated by the viral Tat protein through activation of the Akt survival pathway (180). Consequently, CHME-5 cells have been extensively used to investigate the contribution of microglia to HIV persistence in the CNS and the pathogenesis of HIV-associated neurocognitive disorders (HAND) (181).

Beyond HIV, CHME-5 cells were evaluated for susceptibility to other pathogens. The cells were found to be resistant to murine norovirus and human coronavirus 229E (182, 183), but susceptible to *Chlamydia pneumoniae*, chikungunya virus, coronavirus OC43, and Zika virus (ZIKV), with Zika infection disrupting mitotic spindle formation (184–186) and Acute OC43 infection inducing increased matrix metalloproteinase activity and enhanced NO production, although persistent infection could not be established (182, 183). Additional functional studies reported induction of iNOS expression and consequent increased NO release following overexpression of endogenous retroviral envelope proteins or stimulation with pro-inflammatory cytokines (TNFα, IL-1β, and IFN-γ) (185, 187). In contrast, as expected, exposure to IL-4 increased arginase activity, which could be attenuated by several antiretroviral drugs (186).

Despite the substantial body of literature supporting an activated and immunoresponsive phenotype, subsequent analyses raised significant concerns regarding the authenticity and consistency of CHME-5 stocks. Several canonical human cytokine transcripts, including IL-1α/β, IL-6, IL-10, IL-12, TNFα, TGF-β, IFN-γ, macrophage inflammatory protein-1α (MIP-1α), and monocyte chemoattractant protein-1 (MCP-1), were reported as undetectable using human-specific RT-PCR assays (185). Most importantly, genotyping studies demonstrated that multiple CHME-5 stocks circulating across different laboratories were cross-contaminated with rat GBM cells (140), questioning the validity of earlier findings. These observations underscored the critical need for rigorous cell line authentication, which was addressed through re-characterization and certification by the ATCC, giving the official designation code “CRL-3304”. This authentication ensures human origin and excludes cross-species contamination, limiting the impact of genetic drift. Moreover, it may explain the phenotypic variability reported by the different laboratories, arising from historical contamination issues and serial passaging rather than intrinsic biological differences in the microglial model itself.

### 2.2 HMC3: a reliable *in vitro* system for the study of human microglia

ATCC certifies HMC3 human origin, genetic identity, and absence of cross-species contamination through cytochrome c oxidase I (COI) PCR analysis and short tandem repeat (STR) profiling. ATCC also provides standardized guidelines for optimal culture and maintenance, including the use of Eagle’s Minimum Essential Medium (EMEM) supplemented with 10% fetal bovine serum (FBS) and antibiotics under humidified conditions at 37 °C, 5% CO_2_. Early passages require frequent medium replacement to limit acidification. Experiments with these cells are often performed starting from passage 3, following sub-culturing to ensure population homogeneity. Routine maintenance involves plating them at approximately 20000 cells/cm², replacing medium daily or every other day, and passaging twice weekly avoiding full confluency. HMC3 cells can also be successfully maintained in alternative media, including MEM or DMEM/F12 supplemented with 10% serum, which provide enhanced pH stability, support more rapid proliferation, and preserve typical microglial morphology (162). The immunophenotypic characterization of HMC3 across multiple studies confirms both its microglial lineage identity and functional plasticity. Resting cells are consistently reported as CD68-positive (antibody-dependent) and GFAP-negative, with low basal expression of CD11b and MHCII molecules (137, 139, 162). Transcriptional analyses have validated CD11b expression and confirmed the absence of GFAP transcripts. The basal expression of various toll-like receptors (TLR1, TLR2, and TLR6) has been documented, with increased receptor expression occurring following exposure to viral proteins, consistent with inducible innate immune responsiveness. The expression of CSF1R has also been demonstrated (188, 189).

While the classical dichotomy of microglial polarization into M1/M2 states is now recognized as overly simplistic, HMC3 cells express markers associated with both pro-inflammatory (M1-like: CD40, CD86) and anti-inflammatory/reparative (M2-like: CD163, CD206) activation states. Under basal conditions, approximately 30-60% of cells express CD40, whereas other polarization markers including CD86, CD80, and MHCII are present in smaller sub-populations (5-20%). Phenotypic expression is stimulus-dependent: exposure to low concentrations of Aβ_1_–_42_ reduces CD40 expression and favors M2-like phenotypes, particularly during amyloid phagocytosis (CD163 and CD206 expression). In contrast, higher concentrations of Aβ_1_–_42_ aggregates promote a shift toward an M1-like phenotype characterized by enhanced CD40 and CD11b expression, with minimal modulation of M2 markers (190–194). Co-culture with neuronal precursor cells induces marked upregulation of MHCII and CD206, whereas incubation with GBM cell-CM suppresses M1-associated genes (e.g., TNFα and C-X-C motif chemokine ligand (CXCL) 10 (CXCL10)), while increasing M2-associated markers, including the IL-1 receptor antagonist and CD204. Moreover, glioma-derived M-CSF enhances the pro-angiogenic activity of HMC3 cells without significantly altering polarization patterns. Independent investigations from the depositor group further validated this antigenic profile, demonstrating that resting HMC3 express Iba1 and low CD14, but are GFAP-negative, while stimulation with IFN-γ robustly induces expression of MHCII, CD68, and CD11b. Variability in basal CD68 and CD11b detection among studies appears largely attributable to antibody-dependent sensitivity differences, as flow cytometric analyses have identified low but still consistent basal expression of CD68, Iba1, MHCII, CD14, and CD45 (195, 196). The chemokine receptor expression profile of HMC3 cells closely mirrors adult primary human microglia. Cells demonstrate high surface expression of C-C motif chemokine receptor (CCR) 3 (CCR3), C-X-C motif chemokine receptor (CXCR) 1 (CXCR1), and CXCR3, low expression of CCR2, and intracellular localization of CCR5. Stimulation with TNFα or IFN-γ induces further upregulation of CCR3 and CXCR3, while transcriptional analyses reveal inducible expression of CCR10, supported by functional migration assays (191). Under standard culture conditions, CCR2 remains minimally expressed, whereas CX3CR1, a receptor now considered a relatively specific microglial marker, is robustly detected. Beyond innate immune receptors, HMC3 cells express several neurotransmitter receptors, reflecting integration into neuroimmune signaling networks. Notably, expression of the α7 nicotinic acetylcholine receptor (α7nAChR) is dynamically regulated by inflammatory stimuli, with upregulation following LPS exposure and downregulation in response to high concentrations of Aβ_1_–_42_ (31, 195, 197).

Among available human microglial *in vitro* models, including HMC3/CHME-5, HMO6, and iPSC-derived microglia, marked differences exist in accessibility, scalability, and physiological fidelity. HMC3/CHME-5 remains the most robust and broadly applicable model for replicating fundamental microglial morphology and functional activity, owing to its ease of culture, rapid proliferation, commercial availability, and extensive literature validation. These characteristics make HMC3 particularly well suited for high-throughput screening and mechanistic investigations, especially within the context of neurodegenerative and neuroinflammatory research, while preserving essential functions such as phagocytosis, metabolic responsiveness, and receptor-mediated signaling (106, 133, 163, 198). Although immortalization and early authentication concerns place some limitations on its translational accuracy, the ATCC-certified HMC3 line represents the most reliable and reproducible platform for routine experimental studies (162). By comparison, HMO6, a v-myc–immortalized embryonic microglial line, retains several microglial markers and phagocytic activity, but provides a markedly attenuated inflammatory cytokine response compared to primary cells, limiting its suitability for detailed immune functional studies (146, 148, 163). iPSC-derived microglia offer the highest degree of physiological and genetic relevance, closely reproducing developmental and transcriptional microglial signatures and enabling patient-specific disease modeling. However, their widespread application is restricted by prolonged differentiation protocols, high cost, batch-to-batch variability, and limited scalability (88, 106, 199).

Overall, the above described model systems fulfill complementary roles: HMC3/CHME-5 provides the most accessible, reproducible, and experimentally tractable platform for discovery-based and high-throughput applications, whereas iPSC-derived microglia offer superior translational relevance for patient-centered disease modeling and mechanistic validation.

### 2.3 Morphological features and functional properties of HMC3 cells

Early morphological descriptions of immortalized human microglial cells reported predominantly globular or elongated cell shapes with thick processes, abundant dark cytoplasmic granules, and clear cytoplasm (139, 162, 200). When cultured on fibronectin-coated substrates, HMC3 cells largely displayed a globular morphology with prominent perinuclear actin accumulation, as revealed by rhodamine-phalloidin staining (200). Exposure to chemokines such as MCP-1 and RANTES rapidly triggered actin polymerization and cytoskeletal reorganization, demonstrating the preservation of chemotactic responsiveness and migratory potential. Compared to rodent microglia, HMC3 cells showed reduced process branching, greater sensitivity, and faster migration in response to chemokine gradients. Under conventional culture conditions in DMEM, HMC3 cells maintained their characteristic globular, vacuolated morphology. In contrast, subsequent studies employing more enriched media (DMEM-F12 supplemented with 15% fetal calf serum) documented increased morphological heterogeneity, with the appearance of globular, bipolar, and markedly elongated phenotypes, underscoring the strong influence of culture conditions on HMC3 morphology.

The intrinsic plasticity of microglial morphology in response to external stimuli has been further detailed by Gunasegaran et al. (163), who quantitatively characterized the morphology of HMC3 cells under homeostatic and pro-inflammatory conditions. In the homeostatic state, HMC3 cells display a balanced polygonal shape with centrally located ellipsoid nuclei and a cytoskeletal organization characterized by peripheral enrichment of F-actin, producing a smooth cytoplasmic appearance with a well-defined cortical actin rim. These cells exhibit a relatively rough surface texture and a higher nuclear-to-cytoplasmic ratio (NCR) associated with less circular nuclear geometry, consistent with their ellipsoid nuclear morphology compared to murine microglia. Following LPS stimulation, HMC3 cells undergo pronounced cytoskeletal and morphological remodeling, including a significant increase in cell size, modest enlargement of nuclear area, and a reduction in surface roughness paralleled by a NCR decrease, reflecting cellular spreading, while overall cell and nuclear circularity remain largely unchanged, indicating preservation of basic geometric features. IFN-γ stimulation induces similar morphological responses, characterized by increased cell size, reduced surface roughness, and decreased NCR. Differently from LPS exposure, IFN-γ also results in a modest increase in cell circularity, while nuclear size and shape remain unaffected. Under both pro-inflammatory stimuli, the emergence of rare giant multinucleated cells has been observed, especially following IFN-γ treatment (163). To quantitatively capture the highly dynamic and heterogeneous microglial morphologies, including intermediate states linked to migration, surveillance, phagocytosis, and process remodeling, fractal analysis has emerged as a valuable analytical approach (201). Fractal analysis enables objective quantification of complex cellular patterns that are not adequately described by conventional morphometric methods, facilitating detection of both gross morphological changes and subtle transitional forms.

Among the fractal methods, box-counting analysis and the calculation of fractal dimension are the most widely applied to define microglial morphology because they are highly sensitive to features directly related to branching complexity and cell contour geometry. These approaches have demonstrated high utility in microglial studies also becoming readily accessible through dedicated software tools, including automated pipelines (202–204). Alternative methods, such as dilation, mass-radius analysis, and local connected fractal dimension approaches, have also been explored, though to a far lesser extent (205–208). Despite the expanding application of fractal tools in neuroscience and biomedical image analysis, reports specifically applying fractal analysis to microglial cells remain relatively limited. Notably, to date only two studies have directly implemented these approaches in HMC3 cells, using fractal metrics to derive complementary quantitative descriptors of cell morphology (202, 203). The influence of basal culture conditions and external stimuli on HMC3 morphological plasticity across the main representative studies is summarized in **Table 1**.

**Table 1:** Culture conditions and stimuli used to modulate HMC3 morphological plasticity.

	Culture Conditions					
Reference	Basal medium	Substrate/supplements	Stimulus applied	Plasticity state/condition	Morphological features	Main features
Peudenier et al., 1995 (164)	Standard culture medium	Uncoated plastic	None (basal)	Baseline/homeostatic-like	Predominantly globular or elongated cells with thick processes, abundant cytoplasmic granules	Immortalized HMC3 retain core microglial morphology under basal conditions
Dello Russo et al., 2004 (162)	DMEM	Uncoated plastic	None (basal)	Baseline/resting-like	Globular, vacuolated morphology maintained	Conventional DMEM favors homogeneous morphology
Dello Russo et al., 2004 (162)	DMEM	Fibronectin-coated substrate	None (basal)	Adhesion-influenced plasticity	Predominantly globular morphology with strong perinuclear F-actin accumulation	Substrate composition alters cytoskeletal organization
Dello Russo et al., 2004 (162)	DMEM	Fibronectin	MCP-1, RANTES	Chemotactic/migratory state	Rapid actin polymerization and cytoskeletal reorganization	HMC3 preserve chemokine-induced motility responses
Gunasegaran et al., 2017 (163)	DMEM/F12	10–15% FCS	None	Homeostatic-like	Polygonal cells, ellipsoid nuclei, cortical F-actin rim, high nuclear-to-cytoplasmic ratio	Enriched media increase morphological complexity
Gunasegaran et al., 2017 (163)	DMEM/F12	10–15% FCS	LPS	Pro-inflammatory–like	Increased cell size, cellular spreading, reduced surface roughness, decreased NCR	Inflammatory activation induces marked cytoskeletal remodeling
Gunasegaran et al., 2017 (163)	DMEM/F12	10–15% FCS	IFN-γ	Pro-inflammatory–like	Increased cell size, decreased NCR, increased cell circularity	IFN-γ induces distinct geometric changes compared to LPS
Gunasegaran et al., 2017 (163)	DMEM/F12	10–15% FCS	LPS or IFN-γ	Reactive inflammatory	Rare giant multinucleated cells observed	Strong inflammatory cues promote extreme morphological states
Gunasegaran et al., 2017 (163)	DMEM/F12	10–15% FCS	Multiple conditions	Mixed/transitional states	Coexistence of globular, bipolar, and elongated cells	Morphological heterogeneity reflects intrinsic plasticity

### 2.4 Molecular and proteomic characteristics of HMC3 cells

The human HMC3 microglial cell line has been extensively characterized at both molecular and metabolic levels, supporting its designation as a metabolically active and immune-responsive *in vitro* model that preserves core features of human microglia, including innate immune signaling, oxidative bioenergetics, lipid mediator synthesis, and neurotrophic competence (162). Although baseline expression of classical microglial surface markers varies with culture conditions and activation state, HMC3 cells retain fundamental myeloid attributes. Early studies reported expression of CD68 and CD11b alongside robust phagocytic capacity, while resting cultures typically display low basal levels of CD68, CD11b, CD14, CD45, and MHCII, which are strongly upregulated upon inflammatory stimulation, highlighting preserved phenotypic plasticity. In contrast, the microglia-specific marker Iba1 is consistently expressed, whereas astrocytic (GFAP) and neuronal markers are absent (195). Functionally, HMC3 cells express a broad array of pattern-recognition and chemokine receptors, including TLR1/2/6, CCR3, CCR5, CXCR1, CXCR3, and CX3CR1, ensuring competency in pathogen sensing, chemotaxis, and immune activation. In addition, neuroimmune receptors such as α7 nicotinic acetylcholine receptors, α2-adrenergic receptors, and neurotensin receptor-3 enable investigation of interactions between inflammatory signaling and neuromodulatory pathways.

Consistent with this receptor repertoire, HMC3 cells exhibit dynamic polarization capacity and can adopt both M1- and M2-associated phenotypes depending on environmental cues. Exposure to amyloid aggregates, neuronal signals, or tumor-derived factors drives polarization toward either pro-inflammatory activation or enhanced phagocytic programs. At baseline, HMC3 cells display a cytokine secretion profile dominated by IL-6 production, with other inflammatory cytokines (TNFα, IL-1α/β, IL-4, IFN-γ) largely absent or minimally detectable, alongside low basal expression of regulatory mediators such as IL-10 and TGF-β. Pro-inflammatory stimulation with LPS or IL-1β markedly amplifies IL-6 release. Viral immune activation, including exposure to HCV NS3 protein, triggers TLR/NF-κB and IFN-dependent signaling cascades that promote secretion of IL-1β, IL-6, TNFα, IL-8, and type I IFNs, while co-culture with HIV-activated monocytes elicits chemokine release (CXCL10, C-C motif chemokine ligand (CCL) 5 (CCL5), CCL2), and additional cytokine output (166, 167, 209). These immune responses are modulated by microRNA networks, particularly miR-17, which couple redox metabolism to inflammatory signaling by repressing NADPH oxidase 2 (NOX2) and NOX4 expression.

Metabolically, HMC3 cells exhibit a highly oxidative phenotype characterized by elevated mitochondrial respiration, sustained ATP production, and constitutive generation of ROS, consistent with metabolic features described in the CHME-5 progenitor line and validated in HMC3 cultures (210, 211). Upon activation, microglia amplify production of ROS and RNS, including NO generated by iNOS. This imbalance between oxidant production and antioxidant defenses drives oxidative stress and formation of peroxynitrite, a highly cytotoxic effector molecule capable of inducing DNA, protein, and lipid damage, a pathological signature widely associated with neurodegeneration (212–215).

Therapeutic modulation of this inflammatory–metabolic axis has been explored in HMC3 using dietary nutraceuticals. Among these, carnosine (β-alanyl-L-histidine) treatment, at a maximal non-cytotoxic concentration (10 mM), significantly reduced NO bioavailability without affecting intracellular superoxide levels, while improving cellular energy status, as shown by increased ATP/ADP ratio and elevated cellular energy charge. This study provided the first direct characterization of basal bioenergetic metabolism in HMC3 cells, identifying a predominant reliance on mitochondrial oxidative phosphorylation paired with high biosynthetic activity (216).

HMC3 metabolism exhibits pronounced plasticity under external stressors. In tumor-microenvironment models such as glioma co-culture, mitochondrial membrane potential, enzymatic activity, and ATP synthesis decline, while extracellular lactate production increases, indicating a metabolic shift from oxidative phosphorylation toward aerobic glycolysis (217). These changes are accompanied by cytoskeletal remodeling and enhanced phagocytic behavior, paralleling immunometabolic adaptations typical of tumor-associated macrophages and activated microglia. Conversely, anti-inflammatory polarization promotes mitochondrial translation, oxidative phosphorylation, antioxidant defense programs, and fatty-acid oxidation, reinforcing the link between M2-like states and oxidative phosphorylation-dominant metabolism.

Lipid signaling further defines HMC3’s functional identity. Expression of 5-lipoxygenase (5-LOX) and 15-lipoxygenase-2 (15-LOX-2) enables biosynthesis of specialized pro-resolving lipid mediators, including lipoxin A4 and resolvin D1 (197). Dietary ω-3 fatty acids modulate these pathways: eicosapentaenoic acid (EPA) enhances IL-6 and brain-derived neurotrophic factor (BDNF) secretion also promoting phagocytosis, whereas docosahexaenoic acid (DHA)-derived mediators such as maresin-1 markedly increase Aβ uptake, highlighting the utility of HMC3 cells for studying resolution-phase inflammatory signaling (218, 219). In parallel, HMC3 cells secrete neurotrophic and angiogenic factors, including BDNF, vascular endothelial growth factor-a (VEGF-A), and insulin-like growth factor-binding protein-1 (IGFBP-1), contributing to neurovascular cross-talk, although amyloid exposure suppresses BDNF release, linking metabolic stress to impaired neuroprotective output (218).

Proteomic analyses have further refined the molecular landscape of HMC3 cells. Comprehensive proteome profiling identified 3713 proteins, including 591 unique to HMC3, distinguishing this line molecularly from C20 microglia and revealing enrichment of pathways associated with mitochondrial oxidative phosphorylation, ribosomal translation, DNA repair, cell-cycle regulation, and antiviral immunity. EMG1, a ribosome biogenesis factor, emerged as the most highly upregulated protein, supporting the elevated biosynthetic phenotype of HMC3 cells (163). Complementary surfaceome mapping identified 857 plasma membrane proteins encompassing cytokine and chemokine receptors, pattern-recognition receptors, g-protein coupled receptors (GPCRs), integrins, ion channels, and adhesion molecules. Notably, several homeostatic microglial markers, including P2RY12, MER tyrosine-protein kinase (MERTK), protein S1 (PROS1), apolipoprotein E (APOE), hexosaminidase subunit β (HEXB), colony-stimulating factor 1 receptor (CSF1R), TGF-β1 receptor type II (TGFBR2), spalt-like transcription factor 1 (SALL1), and fc receptor-like (FCRL) family members, were detected, confirming retention of key microglial identity features (220). Anti-inflammatory cytokine exposure under serum-deprived conditions drove adoption of a M2-like, tumor-supportive phenotype marked by extracellular matrix remodeling, enhanced motility, and upregulated mitochondrial gene expression.

Finally, recent lipidomic investigations have demonstrated that disease-relevant metabolic perturbations reshape lipid signaling in HMC3 cells. Exposure to LPS or palmitic acid (PA) altered ceramide and triacylglyceride pools and differentially regulated TREM2 expression, recapitulating aspects of DAM or neurodegenerative microglia (NAM) lipid phenotypes (221). Targeted phosphoinositide profiling further revealed distinct phosphatidylinositol/phosphatidylinositol phosphates (PI/PIP) signatures relative to murine BV2 cells and dynamic responsiveness to SH2-containing inositol polyphosphate 5-phosphatase 1 (SHIP1) modulation, implicating the phosphoinositide 3-kinase (PI3K)-SHIP1-TREM2 regulatory axis as a tractable signaling pathway in this human microglial model (222).

## 3 The relevance of HMC3 in neurological research

In the homeostatic brain, microglia are well known to play essential roles in synaptic pruning, tissue repair, maintenance of CNS homeostasis, phagocytosis, support of other glial cells, and intercellular communication (29, 40, 223–225). In response to CNS injury or disease, microglia undergo complex functional and phenotypic changes commonly referred to as “activation.” Activated microglia have been described in a wide range of neurological disorders as well as in other pathological conditions characterized by extensive inflammation triggered by diverse insults (213, 226, 227). Early studies primarily identified microglial activation based on morphological changes, observing a transition from the ramified phenotype, typical of the healthy brain, to a more amoeboid appearance in pathological states (228–230). However, it is now evident that microglial activation is far more heterogeneous and dynamic than previously predicted. Advances in functional and omics profiling have revealed a spectrum of activation states with distinct molecular signatures and context-dependent roles, demonstrating that microglia respond differently across diseases and pathological environments (**Supplementary Table 1**).

Within this framework, the human HMC3 microglial cell line has emerged as a widely used *in vitro* model to investigate microglia-driven processes, including neuroinflammation, oxidative stress, neurodegeneration, and tumor-associated pathology. In this section, we describe the diversity of microglial states associated with disease conditions and summarize the current evidence regarding the contributions of HMC3 cells across different pathological contexts. As outlined in **Supplementary Table 1**, HMC3 cells have been employed to study a broad spectrum of disorders, including classical neurodegenerative diseases (AD, PD, and ALS), acute CNS injuries (ischemic stroke, SCI, and intracerebral hemorrhage), metabolic and vascular insults (hyperglycemia, DR, and angiogenesis), cancer (gliomas and brain metastases), infectious diseases (HIV, SARS-CoV-2, *Treponema pallidum*, and enterovirus), and environmental or chemical neurotoxicity. In these works, HMC3 cells are typically exposed to pro-inflammatory stimuli (LPS, cytokines, Aβ, pro-oxidants, lipids, pathogen-derived molecules, or conditioned media) and then treated with pharmacological agents, nanoparticles, natural compounds, or subjected to genetic manipulations to either exacerbate cellular responses or rescue the basal conditions. Together, they portray HMC3 as a flexible human microglial platform to interrogate signaling pathways, microglial polarization, secretory profiles and, very importantly, crosstalk with other glial cells, neurons, endothelial cells, and tumor cells.

### 3.1 Modeling neuroinflammation in HMC3 microglia: classical pro-inflammatory triggers and immune modulation

A major source of experimental heterogeneity across HMC3 studies derives from the nature of the primary inflammatory insult used to model neuroinflammation and induce microglial activation. LPS remains the most widely adopted stimulus to trigger a robust pro-inflammatory (M1-like) phenotype, either alone or in combination with cytokines such as IFN-γ or TNFα, which further potentiate downstream signaling through NF-κB, Janus kinase/signal transducer and activator of transcription (JAK/STAT), mitogen-activated protein kinase (MAPK), and inflammasome pathways (**Supplementary Table 1**) (231–258). In these models, LPS stimulation consistently enhances the expression and secretion of hallmark neuroinflammatory mediators, including IL-1β, IL-6, TNFα, and chemokines (e.g., CXCL8/IL-8), alongside increased production of ROS/RNS and upregulation of classical M1-associated markers such as iNOS, CD86, and human leukocyte antigen - dr isotype (HLA-DR).

The administration of pharmacological or biological co-treatments has demonstrated the capacity to attenuate LPS-driven neuroinflammatory activation, as described in several works (231, 233, 234, 238, 247–249, 254, 259–261). Different anti-inflammatory or immunomodulatory strategies, including carbon quantum dots (233), parthenolide (248), resveratrol (249, 261), N,N-dimethylacetamide (DMA) (254), daphnetin (247), trace amine-associated receptor 1 (TAAR1) modulators (234, 259), as well as conditioned media (CM) approaches including micro-fragmented adipose tissue-derived conditioned media/liver x receptors (MFAT-CM/LXR) activation (231) and exosome-based interventions derived from neural stem cells or bone-marrow mesenchymal stem cells (238, 260), effectively suppressed pro-inflammatory mediator release, reduced oxidative stress, and shifted HMC3 polarization toward anti-inflammatory, M2-like phenotypes. These treatments were consistently associated with increased levels of immunoregulatory mediators such as IL-10, IL-4, TGF-β, and arginase-1, providing functional proof-of-concept for therapeutic modulation of microglia-mediated neuroinflammation in the HMC3 model.

**Figure 7:**
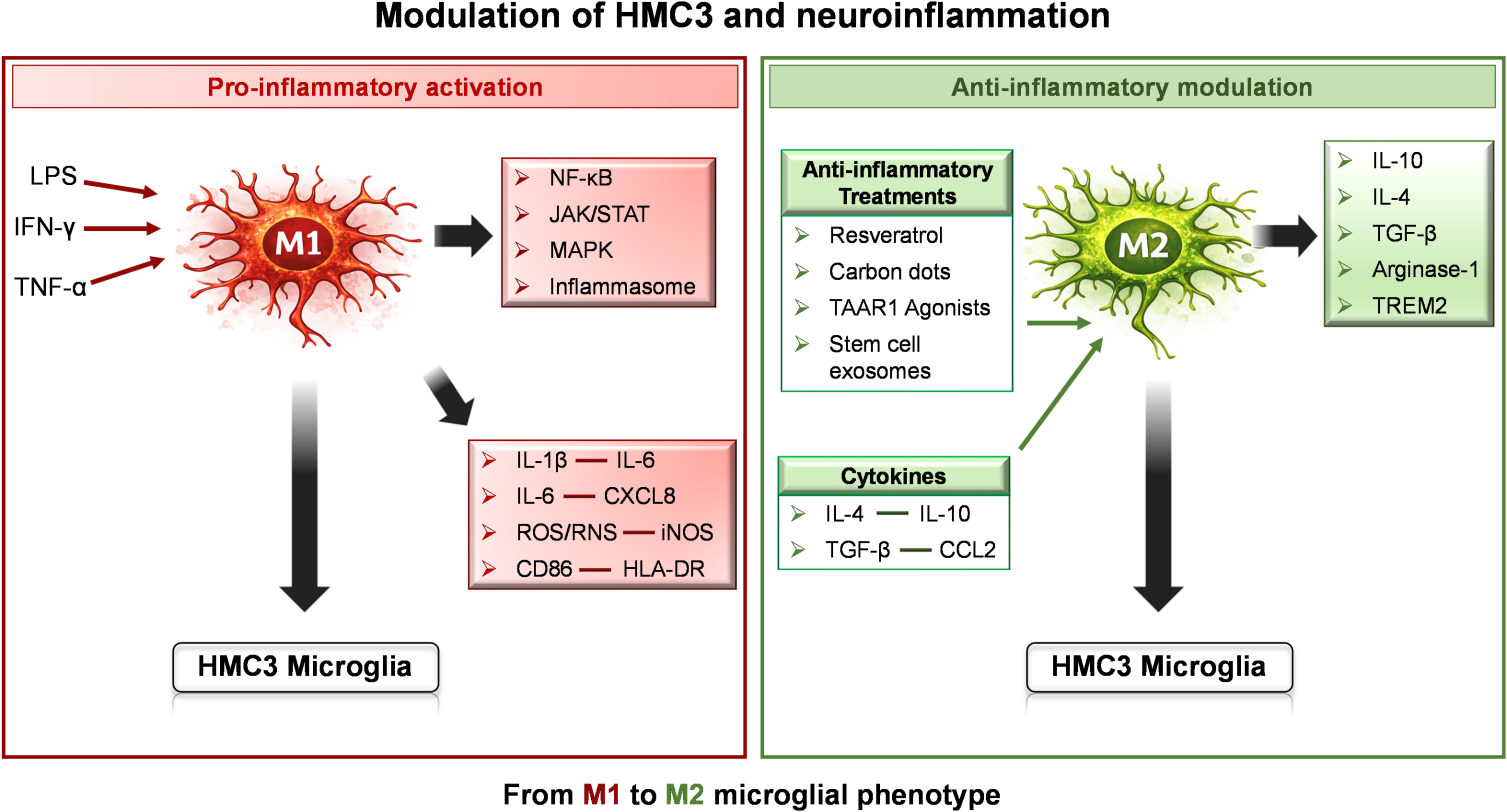
Modulation of HMC3 microglial polarization. Schematic depiction of pro- and anti-inflammatory signaling pathways in the human HMC3 microglial cell line. Pro-inflammatory stimuli (LPS, IFN-γ, TNF-α) activate NF-κB, JAK/STAT, MAPK, and inflammasome pathways, inducing IL-1β, IL-6, CXCL8, ROS/RNS, and antigen-presentation markers. Anti-inflammatory modulation by pharmacological agents, cytokines, and stem cell-derived exosomes promotes an M2-like phenotype characterized by IL-10, IL-4, TGF-β, Arg1, and TREM2 expression.

Beyond pharmacological reprogramming, anti-inflammatory cytokines themselves, including IL-4, IL-10, IL-13, TGF-β1, TGF-β2, and CCL2, have been applied as direct drivers of alternative activation or immune remodeling in HMC3 cells (243, 257). Notably, treatment with cytokine cocktails enhanced endosomal trafficking, promoted MHCII-dependent antigen processing and cross-presentation, and upregulated gene networks involved in myeloid activation and innate immune responses, further highlighting the remarkable immunophenotypic plasticity of HMC3 microglia within neuroinflammatory contexts (243) (**Figure 7**).

Overall, these studies underscore the dynamic responsiveness of HMC3 cells during neuroinflammation, demonstrating that classical inflammatory activators robustly induce M1-like phenotypes, while targeted immunomodulatory interventions or anti-inflammatory cytokine environments can effectively reprogram microglial function toward inflammation-resolving states.

### 3.2 Neurodegeneration-specific microglial activation in HMC3 models

AD is the leading cause of dementia, accounting for 60-70% of cases worldwide, with prevalence projected to reach approximately 139 million by 2050 (262). Its defining neuropathological features include extracellular Aβ plaques and intracellular tau neurofibrillary tangles (263). AD pathogenesis involves a complex interplay of genetic risk, aberrant Aβ and tau, APOE-dependent lipid dysregulation, and chronic neuroimmune activation (264). Importantly, genome-wide association studies indicate that many AD risk genes are selectively enriched in microglia, underscoring their central role in disease development and progression (265).

Studies using AD mouse models have provided important mechanistic insights into the role of microglia in regulating both Aβ deposition and tau pathology. These investigations revealed that microglia can promote Aβ clearance through pathways such as LC3-associated endocytosis (LANDO), regulation of phagocytic receptors, and TREM2-dependent conversion to DAM, while cooperating with astrocytes and being influenced by APOE isoforms (266–270). Conversely, murine studies also demonstrated that microglia may contribute to plaque propagation and tau spreading via sustained neuroinflammation, NLR family pyrin domain containing 3 (NLRP3)/NF-κB activation, defective autophagy, and secretion of EVs containing hyperphosphorylated tau (80, 271–273). Although these models have been crucial for defining molecular pathways governing microglial clearance and propagation of pathology, they do not fully recapitulate the complexity, heterogeneity, and species-specific features of human microglia.

**Figure 8:**
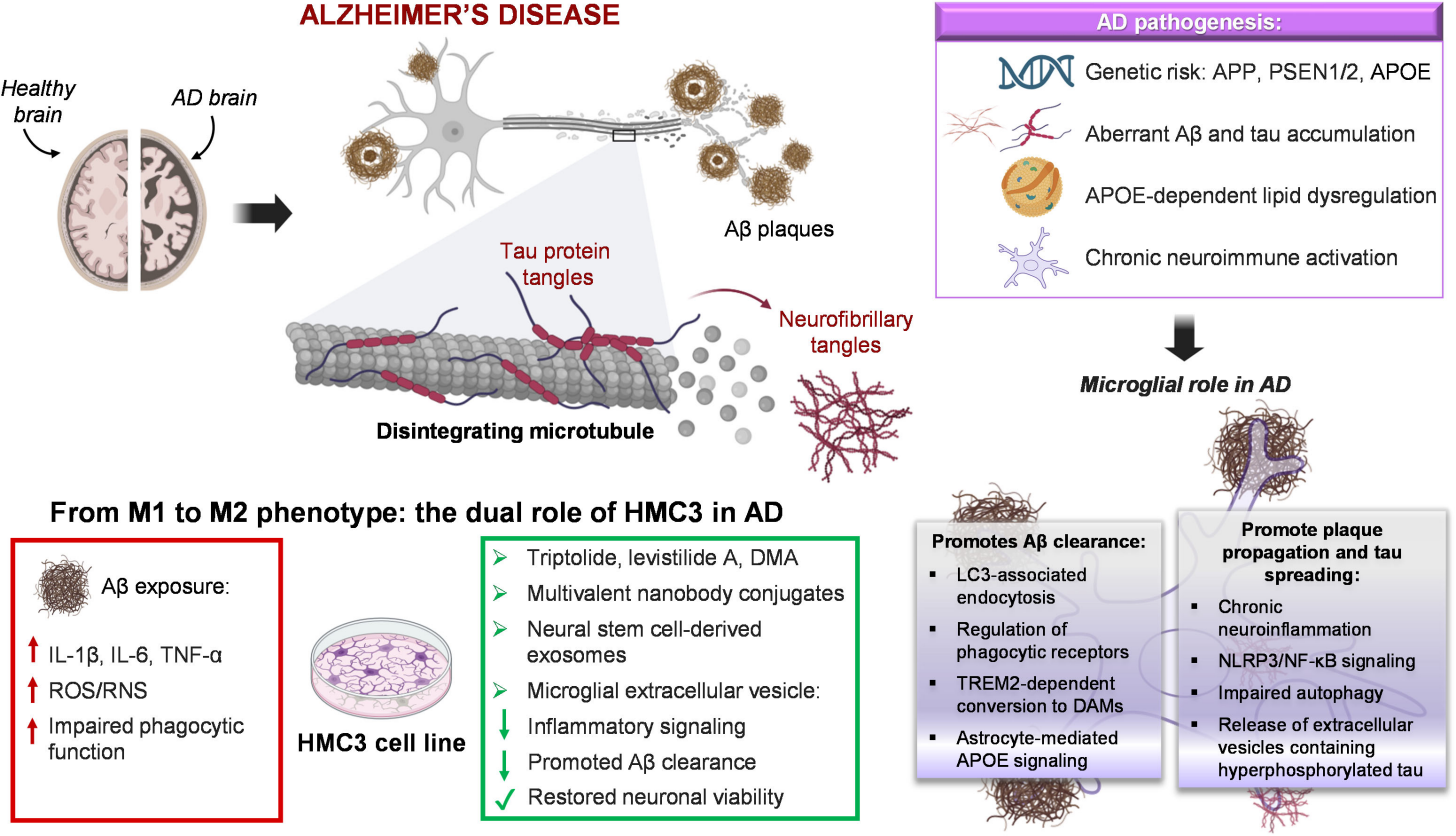
Dual role of HMC3 microglia in Alzheimer’s disease. Conceptual model illustrating the context-dependent functions of microglia in Alzheimer’s disease (AD). Aβ exposure induces pro-inflammatory cytokines release, oxidative stress, and impaired phagocytosis, contributing to plaque propagation and tau pathology via NLRP3–NF-κB signaling. Conversely, therapeutic modulation promotes Aβ clearance through LC3-associated endocytosis, TREM2-dependent conversion to DAMs, and astrocyte-mediated APOE signaling, ultimately supporting neuronal viability.

To overcome the limitations, *in vitro* studies using the HMC3 cell line have been widely adopted to investigate microglial responses to Aβ and tau-related insults under controlled conditions that better reflect species-specific signaling relevant to translational research. Many studies employed AD-relevant insults such as Aβ_1_–_42_ or Aβ_1_–_40_, alone or in combination with cholesterol, fructose, or LPS, to mimic complex metabolic–inflammatory *milieu* (204, 235, 239, 240, 242, 254, 255, 259, 260, 274–277). Under these conditions, Aβ exposure increased IL-1β, IL-6, TNFα, ROS/RNS, and impaired phagocytic function (204, 235, 239, 242, 254, 255, 259, 274, 277). Conversely, several interventions, including triptolide (240), levistilide A (239), DMA (254), multivalent nanobody conjugates (277), neural stem cell-derived exosomes (260), and microglial EVs-based approaches (276) attenuated inflammatory signaling, promoted Aβ clearance, and restored neuronal viability in co-culture systems (**Figure 8**).

**Figure 9:**
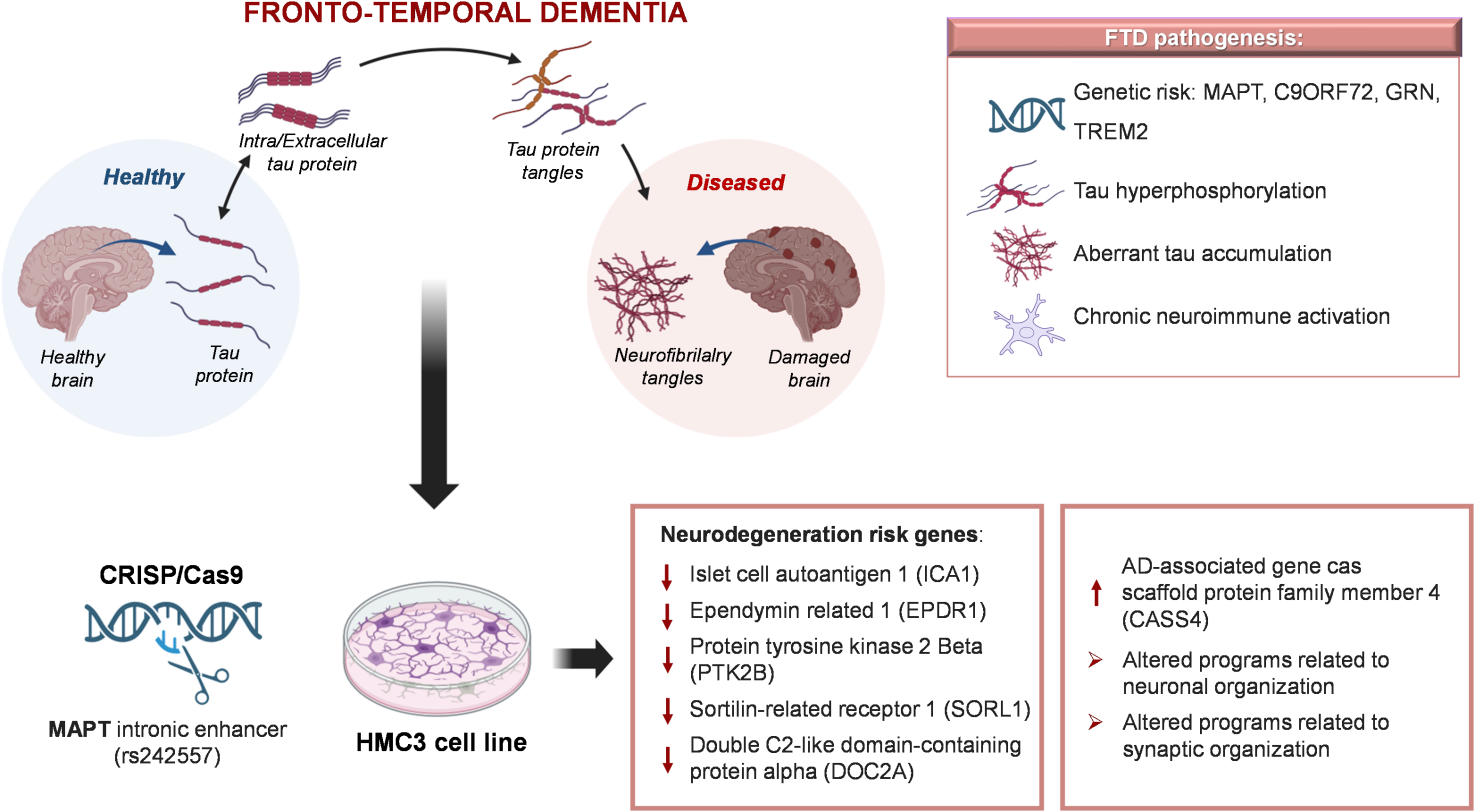
Microglial contribution to fronto-temporal dementia. Schematic overview of fronto-temporal dementia (FTD) pathogenesis, highlighting tau hyperphosphorylation, aberrant tau accumulation, and chronic neuroimmune activation. Genetic risk factors (MAPT, C9ORF72, GRN, TREM2) and CRISPR/Cas9-based modeling in HMC3 cells are shown to affect neuronal and synaptic organization pathways. Neurodegeneration-associated genes implicated in microglial dysfunction are indicated.

Frontotemporal dementia (FTD), the second most common cause of early-onset dementia, is characterized by pronounced microglial activation in the frontal and temporal cortices. Genetic variants in microtubule-associated protein tau (MAPT), chromosome 9 open reading frame 72 (C9orf72), granulin (GRN), and TREM2 represent major risk factors, implicating microglia in both protective and deleterious processes during disease progression. Despite this relevance, most mechanistic studies use iPSC-derived microglia or animal models, while application of HMC3 cells in FTD research remains limited, with the model primarily employed in AD studies. To date, only one study has utilized HMC3 to investigate FTD-related mechanisms, focusing on the MAPT intronic enhancer variant rs242557 (278). Using CRISPR/Cas9-mediated deletion of this region, the authors demonstrated widespread transcriptomic remodeling in HMC3 cells, with downregulation of several neurodegeneration risk genes, including islet cell autoantigen 1 (ICA1), ependymin related 1 (EPDR1), protein tyrosine kinase 2 Beta (PTK2β), sortilin-related receptor 1 (SORL1), double C2-like domain-containing protein alpha (DOC2A), translocator protein-associated protein 1 (TSPOAP1), and α-Syn. Conversely, deletion resulted in the upregulation of the AD-associated gene cas scaffold protein family member 4 (CASS4). Pathway analyses revealed altered programs related to neuronal and synaptic organization, supporting a regulatory role for the MAPT locus in human microglial gene networks relevant to tauopathies, including FTD (**Figure 9**).

PD is the second most common neurodegenerative disorder after AD, clinically characterized by progressive motor symptoms, including bradykinesia, tremor, rigidity and postural instability, together with a wide spectrum of non-motor manifestations, such as hyposmia, gastrointestinal dysfunction, sleep disturbances, and depression (279, 280). Neuropathologically, PD is defined by the selective degeneration of dopaminergic neurons in the *substantia nigra pars compacta* and the accumulation of misfolded α-Syn aggregates forming Lewy bodies. Disease risk reflects a multifactorial interaction between genetic susceptibility (PARK-associated genes) and environmental neurotoxins, and experimental modeling traditionally relies on neurotoxin-based paradigms (e.g., rotenone, 6-hydroxydopamine (OHDA), 1-methyl-4-phenyl-1,2,3,6-tetrahydropyridine (MPTP), and paraquat) or manipulation of PD-linked genes such as SNCA (codifying for α-Syn), leucine-rich repeat kinase 2 (LRRK2), PTEN-induced putative kinase 1 (PINK1), parkin RBR E3 ubiquitin protein ligase (PRKN), and parkinsonism associated deglycase-1 (DJ-1) (281–283).

In PD, early and sustained microglial activation is a central pathological feature, particularly within the *substantia nigra*, where inflammatory signaling driven by α-Syn pathology promotes chronic neuroinflammation, oxidative stress, and inflammasome activation (284–288). These pathways contribute to mitochondrial dysfunction and ferroptosis-associated molecular programs in microglia, providing a mechanistic rationale for employing oxidative stressors (H_2_O_2_, rotenone) and oligomeric α-Syn to model disease-relevant microglial activation in human HMC3 cells (250, 289–292).

**Figure 10:**
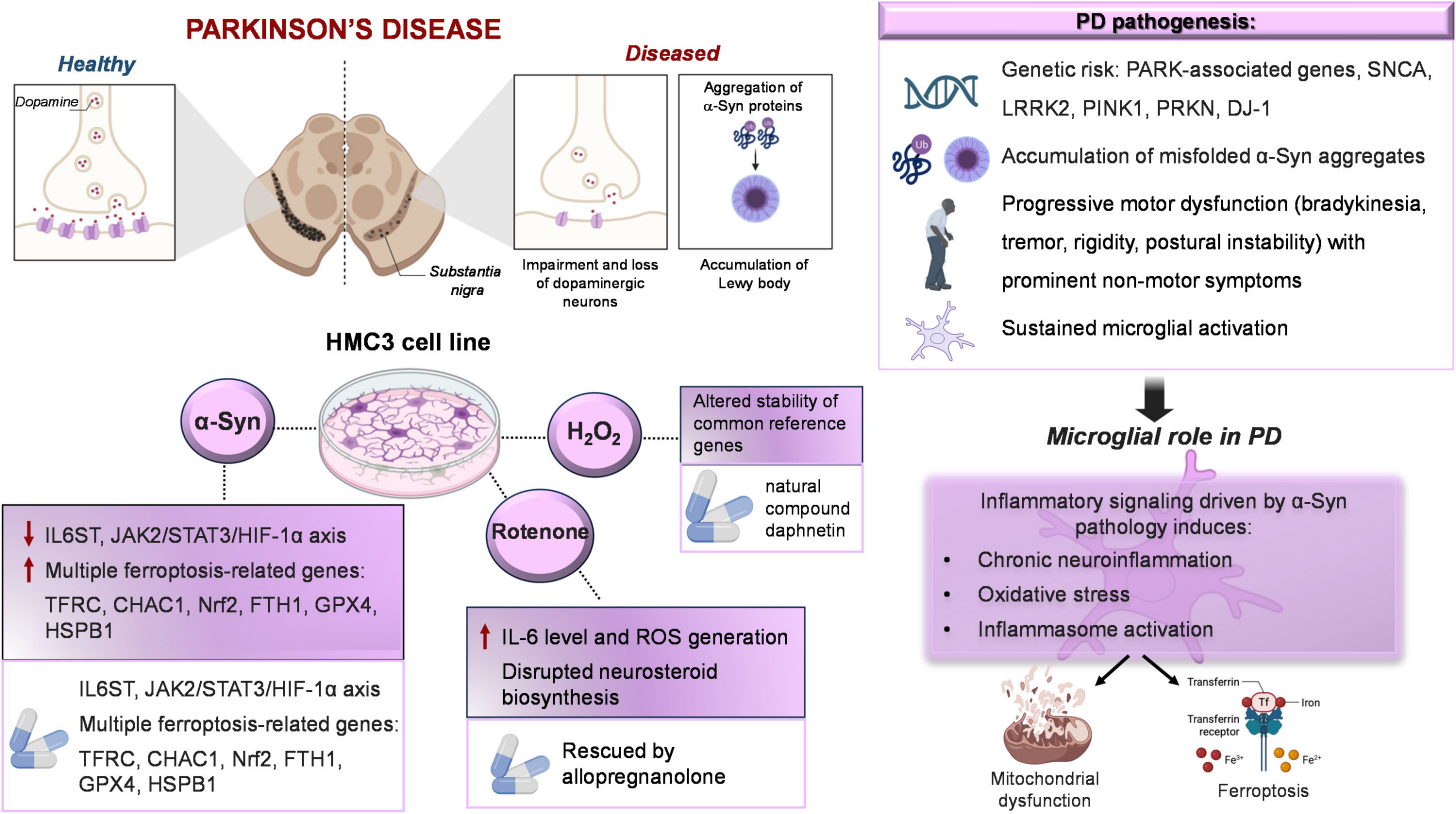
Microglial involvement in Parkinson’s disease. Illustration of Parkinson’s disease (PD) pathology and microglial responses. Accumulation of misfolded α-synuclein (α-Syn) aggregates drives sustained microglial activation, oxidative stress, inflammasome signaling, and ferroptosis-related pathways. HMC3-based models highlight the involvement of IL6ST–JAK2/STAT3–HIF-1α signaling and mitochondrial dysfunction, contributing to dopaminergic neuronal loss.

Still referring to **Supplementary Table 1**, HMC3-based PD studies have applied H_2_O_2_, rotenone, or oligomeric α-Syn to induce neuroinflammatory and oxidative phenotypes directly relevant to PD pathogenesis (250, 258, 291, 293). Toxin exposure resulted in altered stability of common reference genes, emphasizing the transcriptional vulnerability of microglia under oxidative stress conditions and underscoring the importance of careful molecular normalization in PD-related *in vitro* studies (291). Rotenone stimulation disrupted neurosteroid biosynthesis, accompanied by increased IL-6 production and ROS generation, without significantly reducing cell viability at early time points. Importantly, these effects were functionally rescued by supplementation with exogenous allopregnanolone, highlighting a neurosteroid-mediated mechanism capable of attenuating microglial oxidative toxicity (250). Direct modeling of pathogenic PD proteinopathy has been achieved through the application of oligomeric α-Syn to HMC3 cells (293). This stimulus induced cytotoxicity and concentration-dependent modulation of intracellular ROS, while triggering extensive transcriptional remodeling of stress-response pathways. Specifically, α-Syn exposure suppressed components of the IL-6 signal transducer (IL6ST)/JAK2/STAT3/hypoxia-inducible factor 1 α subunit (HIF-1α) axis and promoted marked upregulation of multiple ferroptosis-related genes, including transferrin receptor (TFRC), ChaC glutathione-specific gamma-glutamylcyclotransferase 1 (CHAC1), nuclear factor, erythroid 2–like 2 (NFE2L2, most commonly known as Nrf2), ferritin heavy chain 1 (FTH1), glutathione peroxidase 4 (GPX4), and HSP B (small) member 1 (HSPB1). Pharmacological activation of STAT3 using Colivelin TFA further enhanced STAT3 phosphorylation, HIF-1α stabilization, HSPB1 expression, and ferroptosis gene induction, whereas the STAT3 inhibitor Stattic suppressed these responses, confirming STAT3 as a critical regulator of α-Syn-mediated oxidative and ferroptotic responses in microglia (293). In parallel, therapeutic screening approaches have demonstrated the potential to modulate pro-oxidative microglial activation. Treatment with the natural compound daphnetin significantly reduced inflammatory cytokine output and attenuated microglial toxicity following exposure to H_2_O_2_ or LPS, supporting its potential neuroprotective properties in PD-relevant contexts (258) (**Figure 10**).

ALS represents a progressive neurodegenerative disease marked by selective motor neuron loss leading to muscle weakness and paralysis. Increasing evidence implicates microglia as key contributors to disease pathogenesis, and their capability to adopt diverse, context-dependent activation states, exacerbates neuroinflammation and neuronal injury while also displaying protective functions (294). The upregulation of genes as TREM2, TYRO protein tyrosine kinase-binding protein (TYROBP), APOE, CD33, and membrane-spanning 4-domains, subfamily A (MS4A) detected in ALS patients (295) indicate ALS-specific microglial activation states that likely contribute to disease progression.

**Figure 11:**
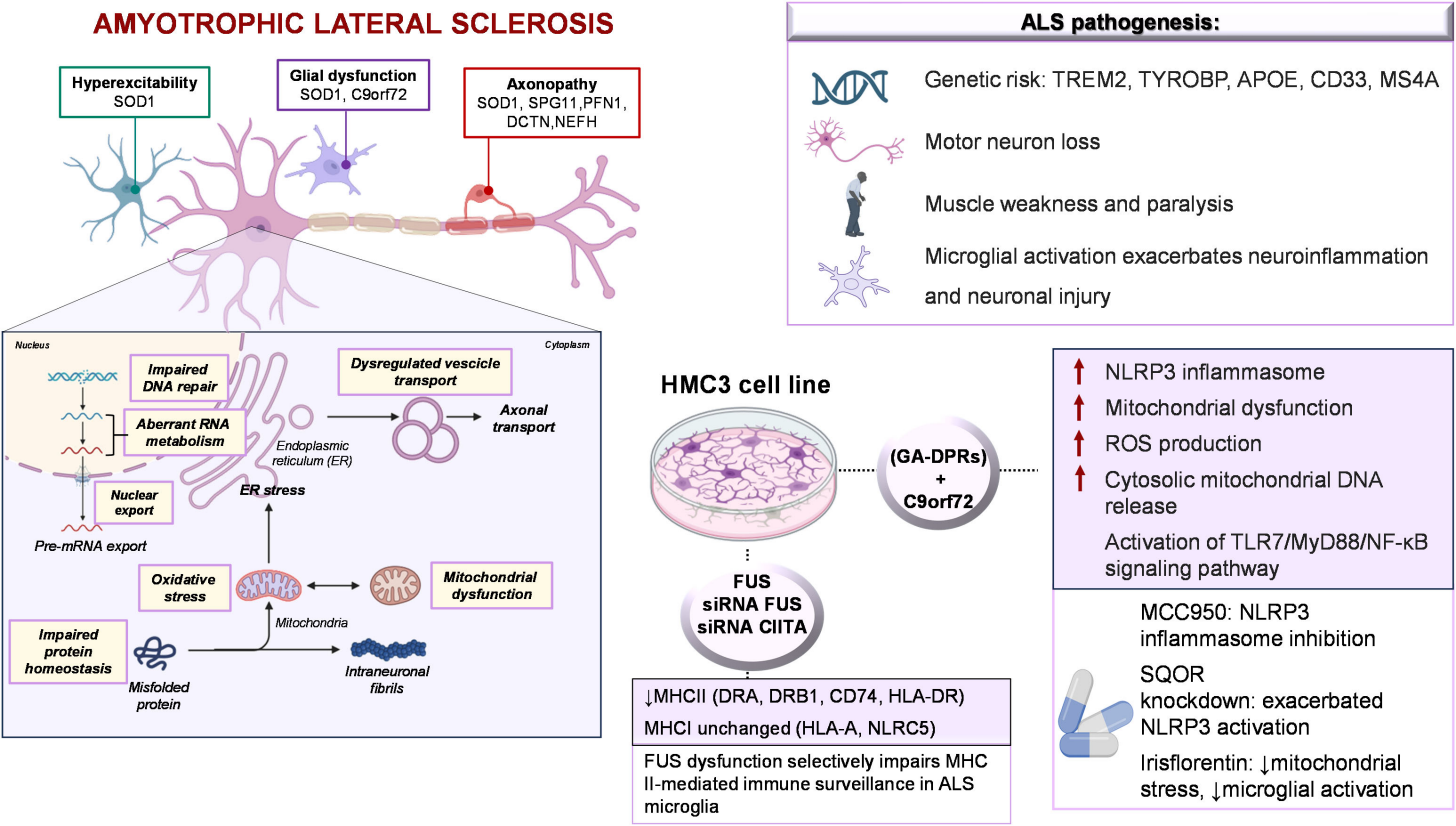
Microglial mechanisms in amyotrophic lateral sclerosis. Overview of amyotrophic lateral sclerosis (ALS) pathogenesis focusing on microglial activation. Genetic risk factors (TREM2, TYROBP, APOE, CD33) and C9orf72-related pathology induce NLRP3 inflammasome activation, mitochondrial dysfunction, ROS production, and aberrant immune signaling. HMC3 models illustrate altered MHCII–mediated immune surveillance and the impact of inflammasome modulation on disease progression.

A limited number of studies have employed the human HMC3 microglial cell line to investigate microglia-specific mechanisms relevant to ALS (296, 297). These works primarily leverage disease-linked molecular triggers, including glycine-alanine dipeptide repeat proteins (GA-DPRs) associated with C9orf72 expansions and modulation of the fused in sarcoma/MHCII transactivator (FUS/CIITA) axis, to dissect innate immune signaling pathways implicated in ALS-related neurodegeneration. Fu et al. (296) demonstrated that exposure of HMC3 cells to GA-DPR (GA50) induces robust NLRP3 inflammasome activation, driven by mitochondrial dysfunction, increased ROS production, and cytosolic mitochondrial DNA release. Mechanistically, these effects were mediated through activation of the TLR7/myeloid differentiation primary response 88 (MyD88)/NF-κB signaling pathway, establishing a direct link between ALS-associated DPR toxicity and innate immune responses in human microglia. Pharmacological inhibition of NLRP3 with MCC950 effectively suppressed inflammasome activation, whereas sulfide:quinone oxidoreductase (SQOR) knockdown further amplified NLRP3 signaling, highlighting the contribution of mitochondrial metabolic pathways to inflammatory regulation. Conversely, treatment with irisflorentin attenuated mitochondrial stress and dampened microglial inflammatory activation, underscoring the relevance of HMC3 cells as a platform for evaluating immunomodulatory strategies in ALS models. In a complementary genetic study, Chi et al. (297) identified the ALS-associated RNA-binding protein FUS as a critical regulator of antigen presentation in HMC3 microglia. siRNA-mediated targeting of FUS or CIITA significantly reduced the transcription of MHCII genes (HLA class II, DR alpha (DRA), HLA class II, DR beta 1 (DRβ1), CD74) and decreased HLA-DR surface expression following IFN-γ stimulation, without affecting MHCI components, such as HLA-A or NOD-like receptor family CARD domain containing 5 (NLRC5). These findings indicate a selective disruption of MHCII-dependent antigen presentation pathways linked to ALS-associated genetic alterations, suggesting a role for FUS in maintaining immune surveillance functions in activated human microglia (**Figure 11**).

### 3.3 Stroke-related neuroinflammation and secondary neurodegeneration in HMC3 microglia models

Ischemic brain injury and stroke represent major causes of secondary neurodegeneration, in which acute hypoxic stress evolves into sustained neuroinflammation and microglial dysregulation, contributing to progressive neuronal loss (298–300). Microglia play a central role in post-ischemic pathology through the production of pro-inflammatory cytokines, oxidative mediators, and chemotactic factors, while also participating in tissue remodeling and repair processes (301–303). The HMC3 microglial cell line has been employed to model these mechanisms using hypoxic stress paradigms and ischemia-associated inflammatory stimuli, providing insight into pathways governing microglia-mediated injury and recovery following cerebral ischemia.

Sawkulycz et al. (231) mimicked stroke-associated inflammation by treating HMC3 cells with LPS and the monomeric and native isoforms of C-reactive protein (mCRP and nCRP, respectively). Among these stimuli, mCRP elicited the strongest pro-inflammatory activation, inducing morphological changes and significant upregulation of IL-1β, IL-6, and TNFα. Notably, activation of LXR signaling with GW3965 as well as exposure to MFAT-CM markedly suppressed cytokine production and attenuated inflammatory markers without altering gene expression profiles, demonstrating the efficacy of metabolic and paracrine immunomodulatory strategies in restraining ischemia-related microglial hyperactivation (231).

Hypoxia-driven microglial responses were further examined using oxygen-glucose deprivation/reoxygenation (OGD/R) models, as reported by Yi et al. (238) that demonstrated a decreased miR-148b-3p expression induced by OGD/R, correlated to augmented TNFα, IL-6, and IL-1β secretion, reduced cell viability, and enhanced migratory capacity. Delivery of bone mesenchymal stem cell-derived exosomes (BMSC-Exos) restored miR-148b-3p levels, improved cell survival, and suppressed inflammatory output. Mechanistically, miR-148b-3p directly targeted the delta-like canonical notch ligand 4/neurogenic locus notch homolog protein 1 (DLL4/Notch1) signaling axis, and knockdown of either DLL4 or Notch1 phenocopied the protective effects of exosome treatment, identifying this pathway as a key regulator of ischemia-induced microglial activation (238). A complementary work carried out by Li et al. (245) highlighted the contribution of long non-coding RNA networks to microglial responses under ischemic conditions. OGD/R stimulation upregulated lncRNA X inactive-specific transcript (XIST) while suppressing miR-25-3p, resulting in enhanced microglial apoptosis and cytokine release (IL-1β, IL-6, IL-8, and TNFα). Silencing XIST restored B-cell lymphoma 2 (Bcl-2) expression, reduced caspase activation, and attenuated inflammatory mediator production. Functionally, XIST acted as a miR-25-3p sponge regulating the expression of the inflammatory adaptor protein TNF receptor associated factor (TRAF) 3 (TRAF3), whose knockdown further blunted microglial activation (245).

### 3.4 Infection-induced neuroinflammation in HMC3 microglia

Microglia represent major targets and viral reservoirs for HIV within CNS and play a pivotal role in the pathogenesis of HIV-associated neurocognitive disorder (HAND). However, limited availability of primary human microglia has restricted investigation of viral infection dynamics in human-relevant systems. Consequently, multiple *in vitro* platforms have been developed, including immortalized microglial lines, latently infected microglial clones, monocyte-derived microglia, induced pluripotent stem cell (iPSC-derived microglia, and cerebral organoid models incorporating resident immune cells (181).

Due to their advantageous properties and high reproducibility, the HMC3 cell line has been extensively employed to model microglial responses to infectious stimuli, spanning viral, bacterial, and immune-mediated paradigms (274, 304–311). HIV-focused studies demonstrated that exposure to HIV-1 Tat or transduction with HIV-1 proviral constructs modulate microglial inflammatory signaling, cytotoxicity, phagocytic capacity, and m^6^A RNA methylation, implicating coordinated activation of the MAPK, serine/threonine-protein kinase hippo (Hpo), PI3K/Akt, rat sarcoma viral oncogene homologs/RAS-related protein 1 (Ras/Rap1), and interferon-responsive pathways in viral sensing and immune remodeling (304, 305, 310). Further viral models have explored endothelial-microglial cross-talk during SARS-CoV-2 infection. CM from endothelial cells exposed to the viral S1 protein triggered rapid activation of the IL-6/STAT3 axis in HMC3 cells, linking vascular infection to secondary microglia-driven neuroinflammatory responses (311).

Bacterial infection paradigms further highlighted innate immune engagement in HMC3 cultures. *Treponema pallidum* and its membrane protein Tp47 induced microglial activation and apoptosis, while disrupting autophagic flux via the mechanistic target of rapamycin complex 1/transcription factor EB (mTORC1/TFEB) pathway, with concurrent modulation of PI3K/Akt/forkhead box O1 (FOXO1) signaling (306, 308). Similarly, exposure to *Porphyromonas gingivalis* outer membrane vesicles (OMVs) provoked robust secretion of IL-6, IL-8, IL-1β, and TNFα in a gingipain-dependent manner, providing evidence of pathogen-derived vesicles as potent drivers of microglial neuroimmune activation (274). Viral infection with enterovirus 71 (EV71) elicited alterations in pro-inflammatory cytokine release and interferon signaling mediated through the miR-342-5p/catenin beta interacting protein 1 (CTNNβIP1)/Wnt-β-catenin axis, highlighting the contribution of post-transcriptional regulatory mechanisms in shaping antiviral responses (309).

Beyond direct infectious paradigms, fetal-maternal immune models, including exposure to inflammatory amniotic fluid and placental lesion conditions, revealed sex- and ancestry-dependent modulation of inflammatory markers and activation states in HMC3 cells, underscoring the impact of early immune programming on microglial phenotypes (307).

These findings highlight the dual role of human microglia as innate immune sentinels and active contributors to bystander neuroinflammatory injury, reinforcing the value of HMC3 cells as a versatile, human-relevant platform for dissecting infection-induced CNS immune responses.

### 3.5 Metabolic stress reprograms HMC3 microglia toward inflammatory and angiogenic phenotypes

Studies using HMC3 demonstrated that metabolic syndrome and related factors, including saturated fatty acids and inflammatory endotoxin, are potent activators of microglial inflammatory and oxidative responses (221, 241). Combined exposure to PA and LPS elicits robust secretion of pro-inflammatory cytokines (IL-6 and MCP-1), increased prostaglandin E2 (PGE2) production that was paralleled by a marked elevation of oxidative stress markers (ROS generation, cyclooxygenase-2 (COX-2) expression, and lipid peroxidation), highlighting, once again, the susceptibility of microglia to metabolic inflammatory stimuli (241). Exposure of HMC3 to metabolic stressors, such as hyperglycemia and lipid overload, further drives a pro-inflammatory, oxidative, and pro-angiogenic phenotype. Chronic high glucose (25 mM) induces endoplasmic reticulum (ER) stress characterized by activation of the PKR-like endoplasmic reticulum kinase (PERK)/eukaryotic translation initiation factor 2 α (eIF2α)/C/EBP homologous protein (CHOP) axis, increased ROS accumulation, upregulation of pro-apoptotic BCL2 associated X apoptosis regulator (Bax) and Bcl-2-associated death promoter (Bad), caspase-3 cleavage, and enhanced apoptosis. Treatment with the BiP inducer-X effectively attenuated these responses, confirming ER stress as a contributing mechanism underlying glucose-mediated microglial toxicity (312).

Lipid dysregulation markedly amplifies microglial dysfunction. Exposure to cholesterol, oxysterols (25-hydroxycholesterol and 27-hydroxycholesterol) and fructose, alone or in combination, promotes IL-1β secretion, MHCII upregulation, mitochondrial stress, and sustained inflammatory activation through Akt/extracellular signal-regulated kinase (ERK)/proto-oncogene tyrosine-protein kinase (Src) signaling pathways, often without immediate cytotoxicity (204, 242). In parallel, PA synergizes with LPS to enhance oxidative damage and inflammation, further increasing lipid peroxidation, ROS, COX-2 activity, PGE2 release, and cytokine production (IL-6 and MCP-1) under metabolically challenged conditions (241). Perturbation of cholesterol homeostasis via methyl-β-cyclodextrin–mediated cholesterol depletion downregulates the lipid transport receptors ATP binding cassette subfamily A member 7 (ABCA7) and low-density lipoprotein receptor-related protein 1 (LRP1), linking alterations in lipid handling to compromised microglial metabolic homeostasis (313). Under diabetic-like conditions, mimicked by treating HMC3 cells with hyperglycemic plasma or sustained high glucose, accumulation of lipid droplets, enhanced sequestosome 1 (SQSTM1)/p62 aggregation, increased ROS generation, induction of inflammatory mediators including IL-6, iNOS, TNFα and IL-1β, and suppression of autophagic flux were observed, correlating with dysregulated TREM signaling and activation of the NLRP3 inflammasome. Pharmacological inhibition of TREM-1 using LP17 reverted oxidative damage, lipid droplet formation, inflammasome activation, and cytokine secretion, establishing TREM-dependent pathways as drivers of microglial metabolic inflammation (314, 315).

In the context of DR and retinal vascular remodeling, LPS-activated microglia acquire a pro-angiogenic M1-like phenotype mediated by PI3K/Akt signaling, with pronounced upregulation of VEGF-A, fibroblast growth factor 2 (FGF2), hepatocyte growth factor α subunit (HGFα), and matrix metallopeptidase 9 (MMP9). Overexpression of the endogenous PI3K inhibitor PIK3IP1 suppresses Akt phosphorylation, reverses M1 polarization, and markedly reduces the release of angiogenic mediators, demonstrating a direct mechanistic link between inflammatory microglial activation and pathological neovascularization (316). At the metabolic-vascular interface, hypoxia-driven lactate accumulation induces YY1 lactylation via the p300 pathway, resulting in enhanced FGF2 and VEGF-A expression and stimulation of endothelial tube formation, migration, and proliferation. These effects emerged in a co-culture system combining HMC3 with human retinal microvascular endothelial cells. Pharmacological blockade of lactate generation using dichloroacetate or inhibition of YY1 lactylation abolishes this pro-angiogenic response, identifying lactate-dependent epigenetic regulation as a novel functional bridge linking metabolic stress to retinal angiogenesis (317). These studies highlight that metabolic stress profoundly reshapes HMC3 microglial function, driving a pathological phenotype characterized by ER stress, lipid mishandling, oxidative injury, inflammasome activation, and augmented angiogenic signaling.

### 3.6 HMC3 microglia as orchestrator of tumor progression

It has been demonstrated that HMC3 microglial cells also play a significant role in investigating tumor progression, especially in brain cancers, by modulating immune responses, cytokine secretion, and supporting tumor cell invasion. Their interactions with the tumor microenvironment are influenced by hypoxia, EVs, and matrix mechanics (318, 319). Studies using these cells demonstrate that tumor-derived signals profoundly reprogram microglial phenotypes toward pro-tumoral, immunomodulatory, and angiogenic states. Cell environment can significantly modulate microglia behavior; in fact, exposure to CM from breast cancer (SKBR3), astrocytes, and brain endothelial cells induces the release of multiple tumor-supportive mediators including CCL5, CXCL8/IL-8, pentraxin 3 (PTX3), colony stimulating factor 2 (CSF2), retinol binding protein 4 (RBP4), fms related receptor tyrosine kinase 3 ligand (FLT3LG), platelet-derived growth factor subunit A (PDGFA), and BDNF, which drive tropomyosin receptor kinase B (TrkB) and human epidermal growth factor receptor 2 (HER2) activation and stimulate proliferation of breast cancer cells and endothelial targets, highlighting the ability of HMC3-secreted factors to promote tumor growth and neurovascular niche formation (320).

Microglial involvement in GBM progression has been further demonstrated in both migration and invasion models. GPX8 released from GBM cells enhances HMC3 cell migration in co-culture systems, while siRNA-mediated GPX8 knockdown significantly reduces microglial motility, implicating a GPX8/IL-6/STAT3 axis in tumor-microglia crosstalk and recruitment (321). Likewise, extracellular matrix remodeling influences microglial polarization: degradation of hyaluronic acid reduces migration and M2-associated markers (CD68/CD163), whereas recombinant chitinase-3-like protein 1 (CHI3L1) restores migratory activity, linking IL-1/CHI3L1- and TGF-β/CHI3L1-dependent pathways to pro-tumoral microglial behavior (322).

At epigenetic and transcriptional reprogramming levels, comparison of HMC3 with multiple GBM cell lines reveals significant divergence in chromatin signatures, underscoring the unique regulatory profile of human microglia within the tumor microenvironment (323, 324). Functional interaction studies demonstrate that glioma-derived neuron exosomes (A-NDEs) promote an immunosuppressive M2-like polarization in HMC3 cells, increasing IL-10 and TGF-β1 secretion and CD163 expression via a miR-200c-3p/zinc finger CCCH-type containing 13 (ZC3H13)/dual specificity phosphatase 9 (DUSP9)/p-ERK signaling cascade. These changes are accompanied by increased uptake of tumor-derived exosomes and stabilization of pro-tumoral signaling pathways that reinforce microglial support of tumor growth (325).

**Figure 12:**
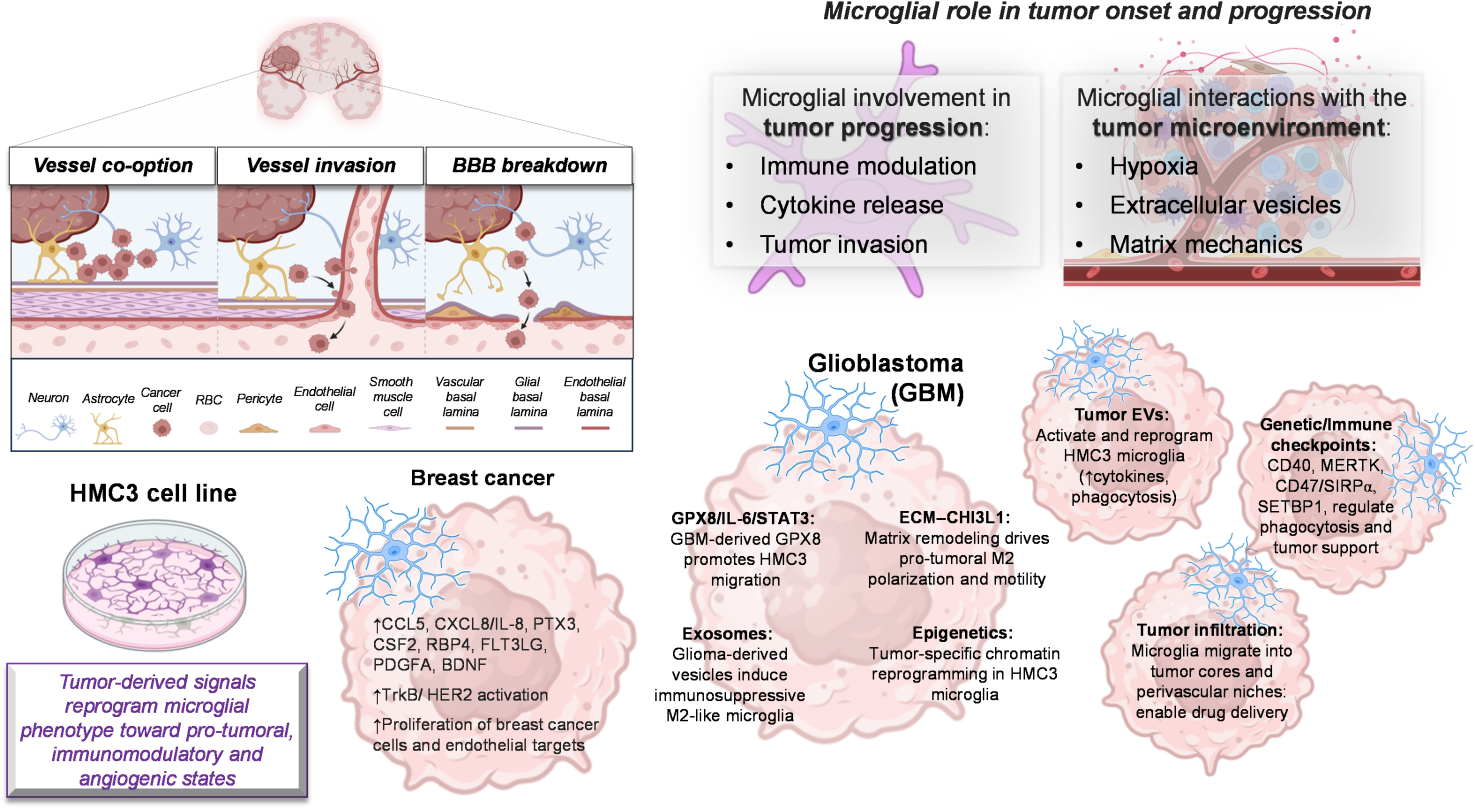
Microglial role in tumor onset and progression. Schematic representation of microglial involvement in CNS tumors and brain metastases, with a specific focus on breast cancer and glioblastoma (GBM). Tumor-derived signals reprogram microglia toward pro-tumoral, immunomodulatory, and angiogenic phenotypes. Microglia contribute to immune modulation, cytokine release, extracellular matrix remodeling, and vessel co-option. Interactions with tumor extracellular vesicles, hypoxia, and genetic checkpoints regulate tumor invasion and progression in GBM and breast cancer brain metastases.

Microglial plasticity is also evident following uptake of tumor EVs. A549 lung cancer-derived exosomes induce profound morphological changes in HMC3, increasing cell size and ramification, enhancing phagocytic activity, and boosting secretion of inflammatory and chemotactic factors such as IL-6, IL-8, CXCL1, with effects dependent on transfer of the oncomiR-1246 (326). These findings highlight the ability of tumor-derived vesicles to simultaneously drive microglial activation and reshape cytokine signaling, favoring tumor invasiveness and immune remodeling (**Figure 12**).

HMC3 infiltration into solid tumor models has been directly demonstrated in three-dimensional spheroid systems. When incorporated into multicellular glioblastoma tumor spheroids, HMC3 cells preferentially migrate toward the spheroid core and perivascular regions. Nanoparticle-mediated delivery of bortezomib enhances microglial infiltration, increasing tumor cytotoxicity and reducing spheroid viability to approximately 20%, supporting the concept that microglia can be leveraged therapeutically as intratumoral drug carriers (327).

Genetic studies further illustrate the tumor-interactive capacity of HMC3. CRISPR-based KO of CD40 and MERTK modifies surface receptor repertoires, alters viability, and increases microglial phagocytic engagement with amyloid aggregates and apoptotic glioma cells, particularly within TREM2+ and CD14+ subpopulations. Dual perturbation of the CD47/signal regulatory protein α (SIRPα) axis enhances phagocytic efficiency toward GBM cells, reinforcing the importance of immune checkpoint signaling in regulating microglia-tumor interactions (328). Finally, transcriptomic modulation of HMC3 behavior has been shown following manipulation of intracellular tumor-related targets. Knockdown of SET binding protein 1 (SETBP1) suppresses proliferation and invasion while enhancing apoptosis in tumor-microglial experimental systems, confirming that shared oncogenic regulators influence both glioma cells and associated microglia (329). Although not all genomic profiling studies produced direct functional outcomes, additional GBM analyses utilizing HMC3 comparisons further validate microglia as key stromal contributors to tumor dynamics (330, 331).

### 3.7 HMC3 as a platform for microglial drug screening

As previously mentioned and summarized in **Supplementary Table 1**, a broad array of pharmacological agents and genetic manipulation strategies has been applied to HMC3 human microglia, underscoring their value as a robust platform for mechanistic investigation and drug discovery. Both pharmacological modulation and targeted genetic interventions enable precise regulation of inflammatory signaling, polarization states, metabolic stress responses, autophagy, and cell survival pathways. Anti-inflammatory reprogramming can be achieved through activation of TAAR1 by 3-iodothyronamine (T1AM), which increases IL-10 production while suppressing NF-κB-dependent production of cytokines; these effects are selectively reversed or enhanced by TAAR1 antagonists or agonists, respectively (234, 259). Similarly, the JAK2/STAT3 inhibitor levistilide A attenuates LPS/Aβ-induced inflammation, promotes M2 polarization, and enhances neuronal viability through microglia-mediated conditioning (239).

Oxidative stress and inflammatory responses are modulated by compounds such as carbon quantum dots, which activate the Nrf2/heme oxygenase-1 (HO-1) antioxidant pathway (233); parthenolide, which suppresses the Akt/MAPK/NF-κB and NLRP3 inflammasome axes (248); nifedipine, which limits hypoxia-associated Ca²^+^ overload and HIF-1α signaling (332); and cofilin inhibition, which mitigates H_2_O_2_-induced cytoskeletal dysfunction and cytokine release (333). Cytokine- and receptor-based strategies, including treatment with IL-4/IL-10/TGF-β (243), daphnetin (247), rhBMP7 (251), and CCR5 blockade with maraviroc (334), further exemplify the dynamic regulation of M1/M2 microglial polarization.

Autophagic and intercellular signaling pathways can be selectively manipulated using rapamycin to enhance TFEB-mediated lysosomal clearance (306), or with stem cell-derived EVs that suppress MAPK/NF-κB signaling and inflammatory mediator production (238, 260). In parallel, genetic targeting approaches refine causal pathway analysis, including regulation of 5-hydroxytryptamine receptor 2B (Htr2b)-neuregulin 1 (Nrg-1)/erb-B2 receptor tyrosine kinase (ErbB) signaling (237), inhibition of inflammatory cascades through the XIST/miR-25-3p/TRAF3 axis (245) or the DEAD-box helicase 54 (DDX54)/MYD88/NLRP3 pathway (246), and suppression of pro-inflammatory signaling via the nuclear paraspeckle assembly transcript 1 (NEAT1)/miR-361-3p/TRAF2 network using triptolide (240).

Finally, targeted manipulation of lipid-handling and immune-regulatory pathways, including SHIP1-dependent phosphoinositide remodeling and altered TREM signaling (335), regulation of the cholesterol transporter ABCA7 (313), and CRISPR-mediated targeting of immune checkpoint receptors (CD40, MERTK, CD47, SIRPα) to boost microglial phagocytic activity (328), further demonstrates the exceptional responsiveness of HMC3 cells to both pharmacological and genetic reprogramming.

**Figure 13:**
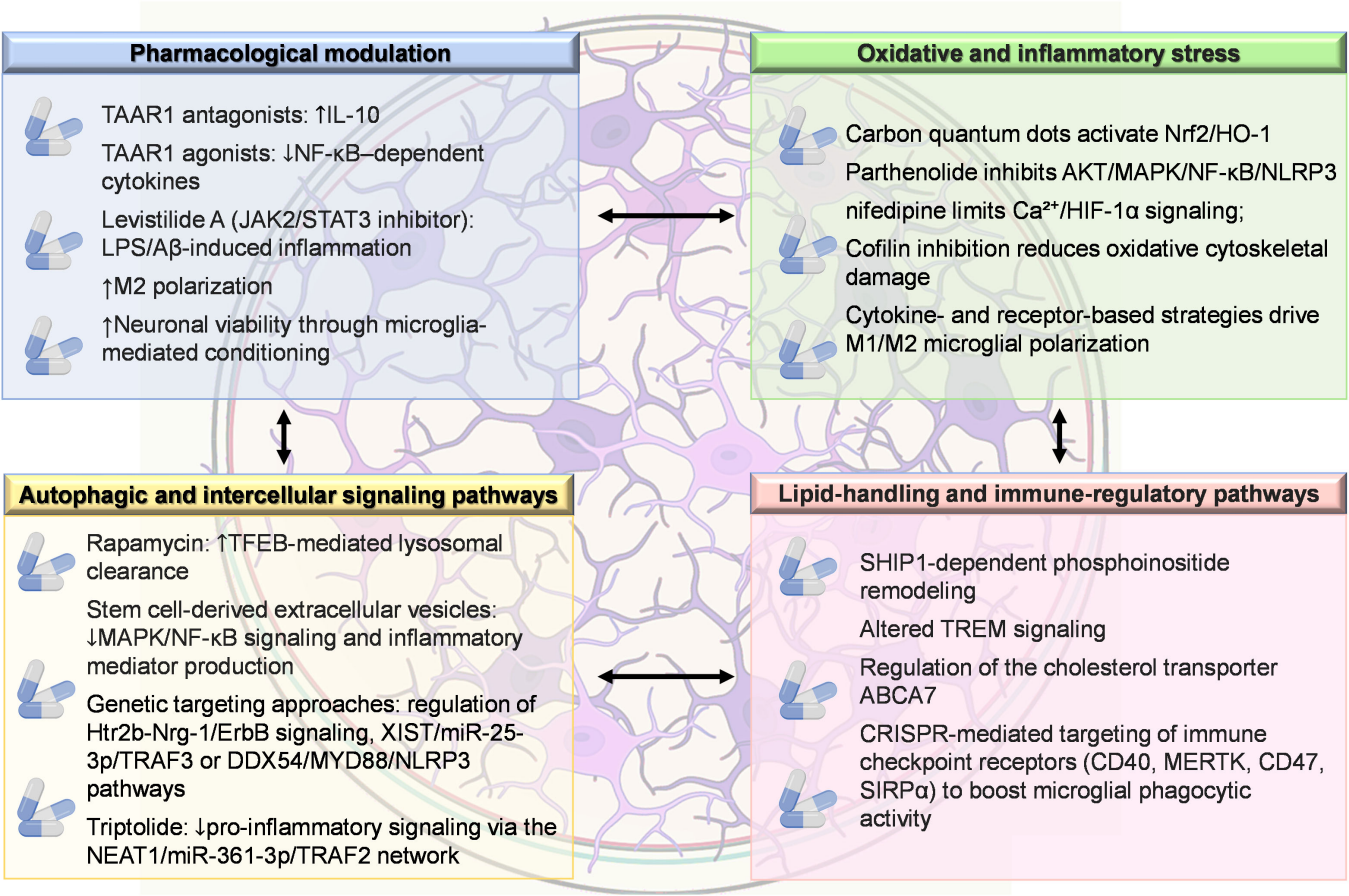
HMC3 human microglia as a platform for mechanistic studies and drug screening. Schematic overview of pharmacological and genetic strategies applied to HMC3 human microglia, highlighting their utility as a robust platform for mechanistic studies and drug discovery. Lipid-handling and immune-regulatory pathways involve SHIP1-dependent phosphoinositide remodeling, altered TREM signaling, regulation of the cholesterol transporter ABCA7, and CRISPR-mediated targeting of immune checkpoint receptors (CD40, MERTK, CD47, and SIRPα) to enhance microglial phagocytosis. Oxidative and inflammatory stress responses include activation of the Nrf2/HO-1 axis, inhibition of AKT/MAPK/NF-κB/NLRP3 signaling, modulation of Ca²^+^/HIF-1α signaling, and attenuation of cytoskeletal oxidative damage, collectively shaping M1/M2 polarization. Autophagic and intercellular signaling pathways comprise rapamycin-induced TFEB-mediated lysosomal clearance and stem cell-derived extracellular vesicle-mediated regulation of MAPK/NF-κB signaling. Additional genetic and pharmacological interventions target Htr2b–Nrg1/ErbB, XIST/miR-25-3p/TRAF3, DDX54/MYD88/NLRP3, and NEAT1/miR-361-3p/TRAF2 pathways, as well as TAAR1 and JAK2/STAT3 signaling, ultimately modulating cytokine production, microglial polarization, and neuronal viability.

Importantly, findings derived from HMC3-based studies can be contextualized using large-scale reference resources such as the Human Microglia Atlas (HuMicA (336);), which integrates transcriptomic data from 90716 microglial cells across 241 human donors representative of diverse neurological and inflammatory conditions, including AD, multiple sclerosis, Lewy body disorders, autism spectrum disorders (ASD), epilepsy, COVID-19, and healthy controls. HuMicA defines nine conserved microglial populations, including disease-associated microglial and inflammatory macrophage subsets originally characterized in murine models and subsequently validated in human tissue. Disease-specific shifts in population abundance highlight the context-dependent plasticity of human microglia, with a glycoprotein non-metastatic melanoma protein B (GPNMB)-high microglial subtype notably expanded in both AD and multiple sclerosis, implicating this population in neurodegenerative inflammatory cascades (**Figure 13**).

## 4 HMC3: rewriting the rules of microglial plasticity

Across the above-described studies, HMC3 consistently demonstrate robust functional plasticity that closely mirrors *in vivo* microglial dynamics, particularly in the context of neurodegenerative disorders. Classical immune stimuli (e.g., LPS, IFN-γ, TNFα, and Aβ), injury-related stressors (e.g., H_2_O_2_, rotenone, mitochondrial toxins, and hypoxia), metabolic overload (e.g., high glucose and PA), pathogen-associated molecules (e.g., HIV Tat, EV71, *Treponema pallidum*, and bacterial OMVs), and environmental pollutants (e.g., PM2.5, diesel exhaust particles, and microplastics) reproducibly induce an M1-like inflammatory phenotype, marked by increased expression of IL-1β, IL-6, TNFα, granulocyte-macrophage CSF (GM-CSF), iNOS, ROS, NF-κB activation, NLRP3 inflammasome components, MHCII, and apoptotic markers. This molecular profile parallels microglial activation signatures observed across multiple neurodegenerative disorders, including AD, PD, ischemic injury, diabetic encephalopathy, and spinal cord pathology. Conversely, therapeutic and experimental interventions effectively reprogram HMC3 cells toward M2-like or resolving states, recapitulating reparative microglial phenotypes associated with tissue recovery. Anti-inflammatory or neuroprotective agents (resveratrol, parthenolide, levistilide A, allopregnanolone, BMP7, and T1AM) and stem cell- or neuron-derived EVs enhance the expression of regulatory and trophic mediators (IL-10, IL-4, arginase1, CD206/CD163, IGF-1) while maintaining or augmenting phagocytic clearance of Aβ aggregates and apoptotic debris, demonstrating that HMC3 models reversible phenotype switching rather than irreversible inflammatory exhaustion.

A major strength of the HMC3 system lies in its integration into multicellular co-culture and CM platforms that allow functional investigation of intercellular communication relevant to CNS pathology. In neuron-microglia models, CM from pro-inflammatory HMC3 increases neuronal vulnerability to oxidative stress and Aβ toxicity in SH-SY5Y, HT-22, U87, and human neuronal cultures, whereas media from pharmacologically reprogrammed or exosome-treated microglia enhances neuronal survival, preserves synaptic and mitochondrial integrity, and limits neurotoxicity. Reciprocally, neuron-derived exosomes and glial-neuronal CM reshape HMC3 metabolic and inflammatory responses, illustrating bidirectional neuroimmune regulation (162, 250, 291).

In neurovascular and blood-brain barrier (BBB) co-cultures with retinal or brain endothelial cells, HMC3 recreate essential features of the neurovascular unit, modulating angiogenesis, endothelial migration, and barrier permeability, and responding dynamically to circulating EVs, including disease-associated vesicles from preeclampsia patients. Within the tumor microenvironment, co-culture with glioma or breast cancer cells reveals reciprocal signaling loops wherein tumor mediators (GPX8, SETBP1, and exosomal miRNAs) regulate microglial migration, immune checkpoint signaling, polarization, and phagocytosis, while microglial secretomes support tumor growth, invasion, and therapy (337, 338). Integration of HMC3 into three-dimensional tumor spheroids further enables modeling of microglial infiltration and nanoparticle-based drug delivery (146, 339, 340). Altogether, these studies establish HMC3 as a versatile human microglial platform that enables: I) reproducible modeling of inflammatory and reparative phenotypes associated with neurodegeneration; II) dynamic multicellular cross-talk among neurons, endothelial cells, and tumor cells, and III) translational applications for mechanistic investigation and CNS drug screening in complex microenvironments.

**Figure 14:**
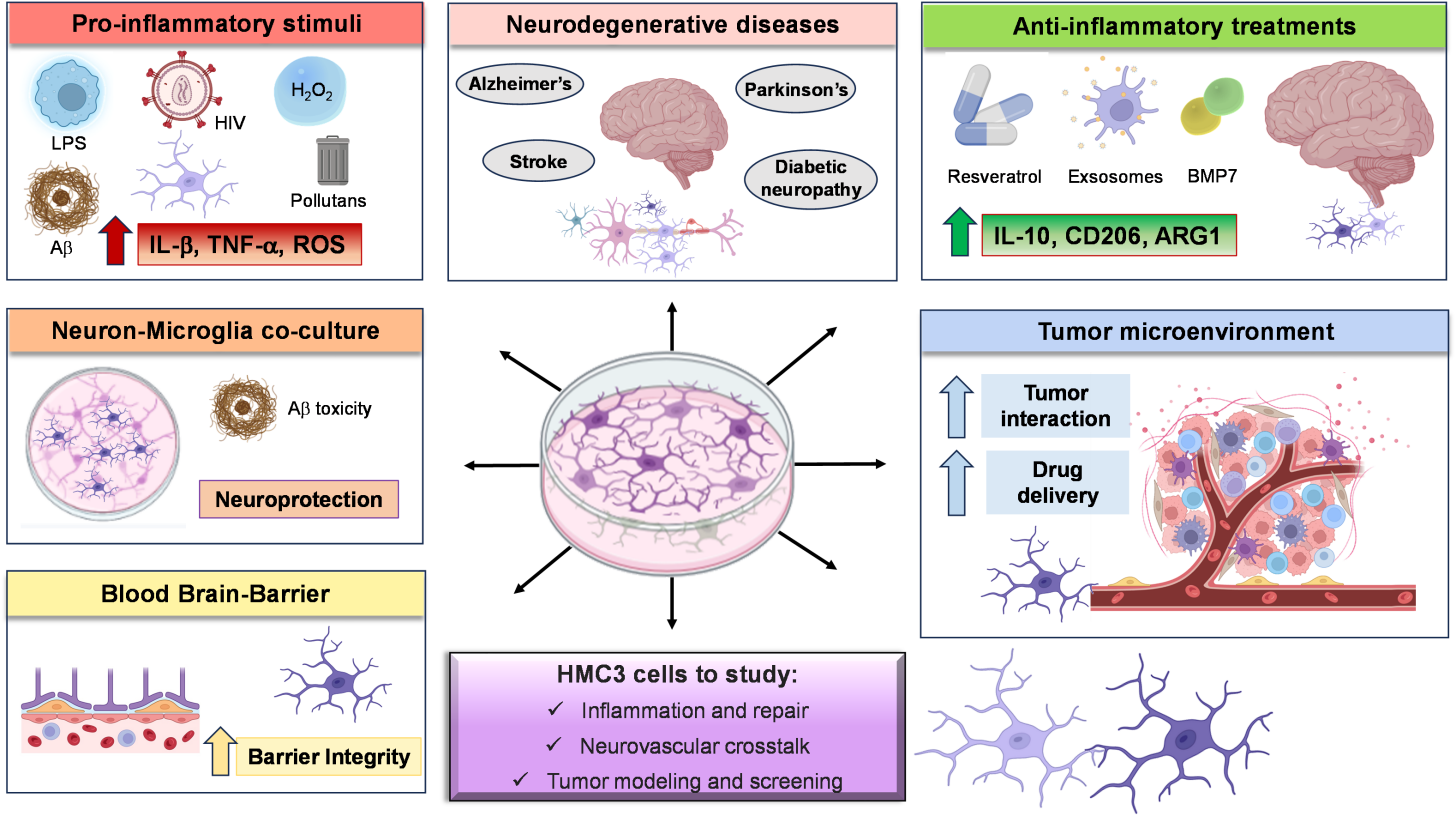
Experimental applications of the HMC3 human microglial cell line in neurological disorders and tumor progression. Graphical summary of the main experimental contexts in which the HMC3 human microglial cell line is employed. Pro-inflammatory stimuli, including LPS, H_2_O_2_, HIV, Aβ, pollutants are used to model neuroinflammatory conditions characterized by increased inflammatory mediators (IL-1β, TNF-α, and ROS). HMC3 cells are applied to study microglial responses in neurodegenerative diseases such as Alzheimer’s disease, Parkinson’s disease, stroke, and diabetic neuropathy. Anti-inflammatory treatments, including resveratrol, exosomes, and BMP7, promote alternative activation marked by increased IL-10, CD206, and ARG1 expression. Neuron-microglia co-culture systems are used to assess Aβ-induced neurotoxicity and microglia-mediated neuroprotection. Additional applications include modeling blood-brain barrier integrity, investigating tumor-microglia interactions within the tumor microenvironment, and evaluating microglia-based strategies for drug delivery. Overall, HMC3 cells represent a versatile *in vitro* platform to study inflammation, repair processes, neurovascular crosstalk, and tumor modeling.

Together, these findings position HMC3 as a powerful system for studying neurodegenerative inflammation, reparative microglial programming, and cell-cell interactions relevant to CNS disease and therapeutic discovery (**Figure 14**).

## 5 Limitations of the HMC3 cell line

Despite the broad utility of the HMC3 model, several limitations must be considered when interpreting experimental findings. As an immortalized human microglial cell line, HMC3 does not fully recapitulate the transcriptional heterogeneity, maturation states, and regional specialization that characterize primary microglia *in vivo*. Immortalization is associated with partial phenotypic drift, including alterations in basal metabolic programs, chromatin organization, and responsiveness to inflammatory stimuli, which may exaggerate or simplify activation dynamics relative to tissue-resident microglia (162, 341–345).

Comparative analyses between human and rodent microglia reveal substantial conservation of core features, including expression of Iba1, PU.1, and DAP12(SYK) and similar responsiveness to M-CSF and LPS stimulation (157–161). However, important species-specific differences persist. Adult human microglia exhibit limited sensitivity to dendritic-cell differentiation signals, lower basal and inducible expression of MHC II molecules, and higher IL-10 production compared with rodent microglia and human monocytes. These distinctions raise concerns regarding how accurately rodent and immortalized cell line models reflect human-specific microglial biology, particularly in complex neurodegenerative and neuroimmune contexts.

In the HMC3 line, discrepancies have been documented in the expression of key receptors and disease-relevant markers, including reduced or absent expression of molecules such as TREM2 and variability in immune surface proteins relative to primary or iPSC-derived microglia (162, 342, 344, 346, 347). These differences may bias signaling cascades central to lipid sensing, phagocytosis, and disease-associated microglial activation, limiting the fidelity of the model for studying certain pathways critical to neurodegeneration. Moreover, the continuously proliferative nature of HMC3 contrasts with the post-mitotic or slowly cycling status of adult microglia *in vivo*, potentially affecting metabolic demand, oxidative stress handling, and long-term responses to chronic perturbations (71, 77, 348–350). Subsequently, compared with iPSC-derived microglia, that better mimic the primary human microglia expression profile retaining developmental context and donor-specific genetic backgrounds, the HMC3 cell line exhibits reduced physiological relevance due to its transformed phenotype.

An additional source of variability arises from methodological heterogeneity across published studies, including differences in culture conditions, stimulus type and dose, exposure duration, and outcome measures. This lack of standardization restricts direct quantitative comparison across datasets and underscores the need for greater experimental harmonization.

Despite these limitations, HMC3 cells remain a highly valuable model for translational studies related to human microglial research. Their robustness in culture, scalability, and compatibility with genetic and pharmacological manipulation make them ideally suited for systematic pathway analysis, high-throughput screening, and mechanistic hypothesis testing. Core microglial functions, including inflammatory activation, phenotype switching, and phagocytosis are reliably preserved, allowing meaningful investigation of disease-relevant cellular mechanisms. When complemented by validation in higher-fidelity systems, HMC3 serve as a powerful bridge between discovery screening and translational neuroimmune research.

## 6 Future perspectives

The HMC3 human microglial model is expected to maintain a central role in neuroscience research while expanding into emerging interdisciplinary fields traditionally considered peripheral to neuroimmunology. Beyond classical applications in neurodegeneration, neuroinflammation, and tumor biology, HMC3-based platforms are increasingly relevant to studies of systemic and organ-brain interactions, including the gut–brain axis (351–353), metabolic disorders (241), immune-endocrine cross-talk, and reproductive system pathologies (250), where microglia contribute to neuroendocrine regulation and reproductive axis remodeling. These developments emphasize the growing recognition of microglia as integrative cellular sensors connecting neural, immune, and metabolic networks across physiological and pathological settings. Notably, integrating scRNA-seq approaches with mechanistic *in vitro* systems, such as HMC3 cells, will be crucial to clarify the SPP1 signaling contribution on microglial activity, with the final aim to evaluate potential targets to tackle inflammation/neurodegenerative mechanisms involved in different pathological conditions. Future advances will require greater standardization of activation protocols and phenotypic marker panels to enable robust, reproducible characterization of HMC3 responses. Integration with iPSC-derived human microglia and *in vivo* models will be critical to validate mechanisms and enhance translational relevance. The increasing adoption of multi-omics strategies will support pathway-level analyses of microglial remodeling, while embedding HMC3 into advanced culture platforms, including brain organoids and microfluidic systems (354, 355), will enable more physiologically accurate investigation of multicellular CNS interactions.

## 7 Conclusions

**Figure 15:**
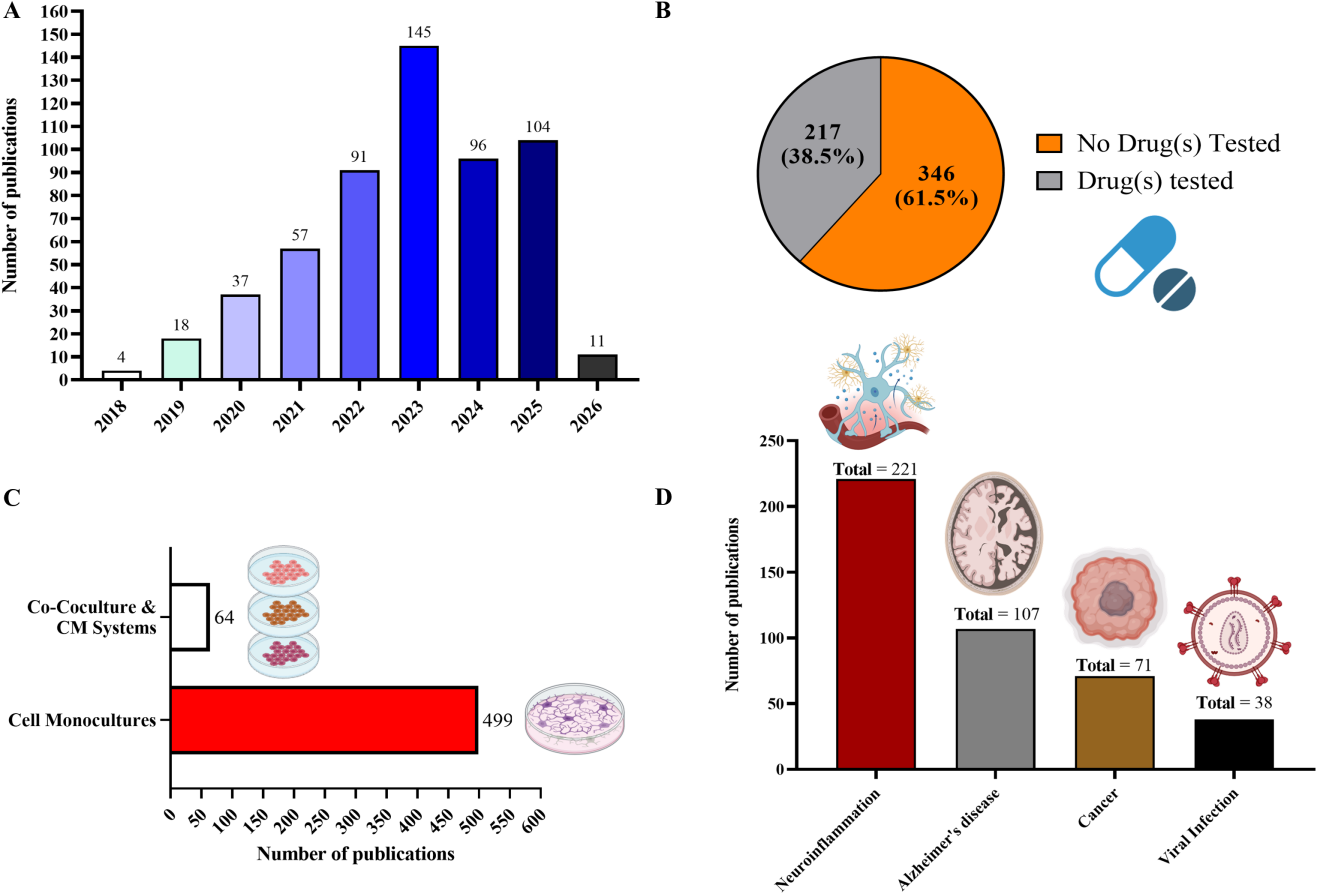
Overview of HMC3 application on neuroscience field during the last decade. **(A)** Number of publications per year; **(B)** HMC3 as a platform for drug testing: studies employing drug(s) versus studies with no drug(s) tested; **(C)** Number of studies in which HMC3 were used as a monoculture versus HMC3 usage as a part of co-culture or treated with conditioned medium (CM); **(D)** Most representative disease model categories in which HMC3 have been used.

One of the primary aims of the present review was to underline the huge number of publications produced by employing the HMC3 cell model during the last decade (almost 600!) (**Figure 15**), with the double purpose of giving an updated state-of-the-art, underlining the incredible attention attracted within the research community and highlighting its translational value.

The evidence reviewed here establishes the HMC3 human microglial cell line as a versatile and accessible platform for studying microglial biology across diverse disease contexts. HMC3 cells recapitulate core microglial behaviors associated with neurodegeneration, neuroinflammation, metabolic stress, vascular remodeling, infection, and tumor progression, including dynamic polarization, oxidative and metabolic remodeling, phagocytosis, cytokine signaling, and multicellular cross-talk with neurons, endothelial and cancer cells.

Their exceptional compatibility with high-throughput pharmacological screening, genetic manipulation, and multi-omics profiling positions HMC3 as an efficient discovery tool for pathway-level investigation that is difficult to achieve using primary or stem cell-derived microglia alone. Although immortalization-related limitations require cautious interpretation, cross-validation with higher-fidelity models and human reference datasets preserves the translational relevance of HMC3-derived findings. Finally, HMC3 should be regarded not as a replacement for primary microglia, but as a powerful front-line human model for hypothesis generation, mechanistic dissection, and early therapeutic screening, continuing to play a central role in advancing neuroimmune research.
