# Supplementary material for: HMC3 revealed: how much do these “Microglia” really tell us?
Source: Front Immunol. 2026 Jun 3;17:1778798. doi: 10.3389/fimmu.2026.1778798 (PMC13271951; doi:10.3389/fimmu.2026.1778798)
Supplement: Supplementary file 1 [file Table1.pdf]

SUPPLEMENTARY TABLE 1 **Overview of scientific publications describing the HMC3 cell line over the last decade, with a focus on the main findings of each study.**

ARTICLE	MODEL	TREATMENT	DRUG TESTED	PATHWAY INVOLVED	MAIN RESULTS	NOTES
**[1]**<br>Martinez Viedma et al., 2018	Viral infection (Zika Virus (ZIKV))	Infection with ZIKV,<br>siRNA for human TLR7, TLR8, and STAT2	N/A	TLR7/8<br>STAT2	• ↑ZIKV replication, time-dependent<br>• ↑STAT1, STAT2, IRF3, IRF7, IRF9, CXCL10, IFIT1, and MX1, 3 days after infection<br>• ZIKV infection<br>• ↺TLR7, TLR8 level after knokckdown<br>• ↑ZIKV after STAT2 knockdown<br>No changes in AXL after blocking TLR7, TLR8, TLR7/8, and STAT2	Experiment was conducted also in human placenta (JEG-3) and epithelial Vero cells.
**[2]**<br>Yu et al., 2018	Neuroinflammation (Cerebral ischemia)	OGD condition,<br>transfection with Smad3, Smad3 siRNA, TGF-β1 or TGF-β1 siRNA,	N/A	TGF-β1/Smad3	• ↑Apoptosis in OGD, Smad3 siRNA and TGF-β1 siRNA cells<br>• ↓Apoptosis in TGF-β1 and Smad3 cells<br>• ↑TGF-β1 in OGD and TGF-β1 cells<br>• ↓TGF-β1 in TGF-β1 siRNA cells<br>• TGF-β1 stable in Smad3 and Smad3 siRNA cells<br>• ↑Smad3 in OGD, TGF-β1 and Smad3 cells<br>↓Smad3 in Smad3 siRNA and TGF-β1 siRNA cells	Hypoxic conditions (95% N2 and 5% CO2) for 5 h and then<br>incubated under normoxic conditions for 12 h to induce OGD.<br>*In vivo:* on cerebral ischemia-induced rats.
**[3]**<br>Jašprová et al., 2018	Neuroinflammation	Bilirubin (UCB), lumirubin (LR, major photo-oxidative product), BOX A and B (monopyrrolic oxidative products)	N/A	TNFα	• ↓Viability by UCB<br>• ↑IL-6, IL-1 β, TNFα after LR<br>↑IL-6 after UCB, BOX A, BOX B	Experiment conducted also on SH-SY5Y, U-87 cells, organotypic rat hippocampal slices and human skin fibroblasts.<br>Concentration of tested compounds = 25 µmol/L.
**[4]**<br>Keck et al., 2018	Viral infection (Venezuelan Equine Encephalitis Virus (VEEV))	TC-83 infection,<br>supernatant from TC-83 infected U-87 MG cells,<br>BAY 11–7082 (BAY-82),<br>mitoquinone mesylate (MitoQ)	N/A	NF-κB,<br>caspase-3/-7	• ↑TC-83 titers in HMC3 > U-87MG<br>• ↑ROS and ↓MMP after 24 hpi<br>• ↑IL-1α, IL-1β, IL-6, and IL-8 after TC-83 and supernatant incubation<br>• ↑TC-83 in HMC3 with U-87 MG supernatants 1 hpi<br>• ↓Caspase 3/7 by MitoQ or BAY-82 after 24 hpi<br>• ↑MMP and ↓ROS by MitoQ or BAY-82<br>• ↑IL-1α, IL-6, IL-8 and ↓IL-1β after MitoQ treatment<br>↓IL-6 and IL-8 by BAY-82	N/A
**[5]**<br>Ahmed et al., 2019	Viral infection (VEEV)	Infection with TC-83 or TrD virus strains,	indolicidin derivatives A1-A6, A9-A12, and B1-9	TNF	↓TC-83 titers and gRNA after A2 or A3<br>↓TC-83 titers after A2 with A3 co-treatment	Antimicrobial peptides derived from indolicidin, resulted in two hits (G5 and G8) with VEEV TC-83 titer inhibition. From G8 sequence was

(Continued)

10.3389/fimmu.2026.1778798

SUPPLEMENTARY TABLE 1 Continued

ARTICLE	MODEL	TREATMENT	DRUG TESTED	PATHWAY INVOLVED	MAIN RESULTS	NOTES
					↓TrD titers after A2 or A3, with no differences wheather pre-treatment or immediate pi.<br>↑IL1α, IL1β, and TNF after A2 or A3<br>↑CCL16, CCL22, IL1RN and IL7 after A2 or A3<br>A2 exhibiting a more robust inhibitory response.	utilized to generate a second generation of peptides: A1-A6, A9-A12, and B1-9.<br>Experiment also conducted on BV2, EOC 20 CRL-2469, HMC3, CRL-3304, U87MG, HTB-14, Vero cells and CCL-81.
**[6]**<br>Bortolotti et al., 2019	Alzheimer's disease, Viral infection (HHV-6A)	infection by HHV-6A (strain U1102)	N/A	TREM2-ApoE pathway	• ↑ Aβ 1-42, IL-1β, ApoE<br>• ↓ IL-10<br>• TREM2 activation<br>• ↑ cell migration<br>↑ total-tau and p-tau (T181)	no significant induction was observed for Aβ 1-40
**[7]**<br>Ho et al., 2019	Depression	Knockdown of STAT3 using siRNA	ketamine or with its metabolites (2R,6R)-Hydroxynorketamine (HNK) or (2S,6S)-HNK alone or in combination with estradiol (E2)	IFN type 1, EEF2, STAT3 and its transcription co-repressor (SIN3A)	• ↑ STAT3, BDNF, PSD95 and SYN1 protein translation<br>• ↓ JAK1 and JAK2 gene expressions<br>↑ JAK3, SOCS1, SOCS3, MX1, MX2, OAS1, OAS2, C1R, IRF7 and IRF9 gene expressions	No expression of STAT4 in HMC3
**[8]**<br>Cappoli et al., 2019	Neuroinflammation	IFNγ, TNFα, IL1β (II)	Rapamycin (0.1-10 nM)	PI3K-AKT-mTOR	• ↑IL-6 expression and release<br>• ↓Intracellular protein content (both under basal condition and in activated cells) and cell viability (only in activated cells)<br>• ↻Microglial viability in cells activated with II<br>• ↓ ROS, IL-10 and STAT3<br>• ↑IL-1β, TNFα, COX2, MCP1 and IL-8 gene expressions (data not shown)<br>↓p-mTORC/mTORC ratio and p70S6K expressions (in cells treated with Rapamycin 1 nM alone or in cells activated with IFNγ, IL1β)	In contrast to rodent cells, rapamycin did not alter human microglial cell viability nor inhibited cell proliferation and did not exert any significant effect on the morphology of the HMC3 cells<br>IL1β (II) (10 ng/ml)
**[9]**<br>Yao et al., 2019	Neuroinflammation (Spinal cord injury, SCI)	Collagen or soybean protein isolate (SPI) or collagen (SPI-collagen) cross-linked with (1-ethyl-3-(3-(dimethylamino)propyl) carbodiimide (EDC)	N/A	Cell proliferation and motility	• ↑Cell grown better on collagen than on SPI-collagen scaffolds<br>↑Cell migration on the collagen and SPI-collagen scaffolds.	SPI-collagen conduit can replace collagen scaffold and improve the regeneration of wounded<br>nerves and spinal cords.
**[10]**<br>Li et al., 2019	Cancer (Glioblastoma Multiforme (GBM))	Suppresion of lncRNA SNHG15,<br>co-culturing with temozolomide sensitive (TMZ-S) or resistant (TMZ-R) cells	palbociclib	lncRNA SNHG15/ CDK6/miR-627 circuit	• ↑CD206, CD163, IL-6, TGF, IL-10, IL-4, IL-13 and CCL2 in both TMZ co-cultures than in HMC3 alone<br>• SNHG15-silencing leads to ↑ miR-627-5p and ↓ CDK6, SOX-2 and GBM tumorigenesis	Article include more *in vitro* analysis of TMZ-S and TMZ-R and *in vivo* model to evaluate the potential anti-GBM activity of CDK6 inhibitor, palbociclib, using TMZ-R PDX mouse models.

(Continued)

10.3389/fimmu.2026.1778798

SUPPLEMENTARY TABLE 1 Continued

ARTICLE	MODEL	TREATMENT	DRUG TESTED	PATHWAY INVOLVED	MAIN RESULTS	NOTES
					• In co-culture HMC3/TMZ-R gene-silencing leads to ↓ M2-polarization of HMC3 and ↓pro-GBM cytokines: TGF-β and IL-6 and sensitize to TMZ treatment<br>Palbociclib overcome TMZ resistance and ↓ M2 markers	
**[11]**<br>Miladinovic et al., 2019	Cancer	LPS, the system xC− inhibitor SSZ (200 μM), l-glutamic acid, or co-cultured with MDA-MB-231, xCT knockdown carcinoma cell, or empty vector control	N/A	14C-cystine uptake system	• ↑ xCT, IRF8, STAT3, ERK1/2, IRF8 when co-cultured with MDA-MB-231 cells<br>• ↑14C-cysteine uptake in HMC3 with LPS or L-flutamic acid<br>• ↑14C-cystine uptake with wildtype or vector control MDA-MB-231 cells, but not with xCT KD or wildtype SSZ-treated MDA-MB-231 cells<br>↓14C-cysteine uptake in HMC3 with SSZ, and SSZ reverse increasing effect of LPS	LPS, 1 μg/mL, SSZ, 200 μM, l-glutamic acid, 300 mM.<br>Simultanously, *in vivo* tests were performed.
**[12]**<br>Zhang et al., 2019	Viral infection (Borna disease virus 1, BoDV-1)	Overexpression and suppression of miR-146a	N/A	IRAK1/TRAF6/NF-κB	• ↑miR-146a after infection<br>• miR-146a overexpression in HMC3 cells promoted viral replication, while its inhibition inhibited it<br>BoDV-1 inhibit expression of IRAK1, TRAF6 what leads to ↓P65 and phosphorylated P65 in the downstream NF-κB pathway	N/A
**[13]**<br>Salsinha et al., 2019	Neuroinflammation	Inflammatory solution of PA and fructose (Western Pattern Diet),<br>miRFP703-IkBa sensor	omega 3, punicic acid isomers (CLA and CLNA)	NF-κB	• Fructose with PA activates NF-κB pathway by IkBa degradation<br>• ↑miRFP703-IkBa with omega 3, CLA and CLNA solutions, what prevents the activation of NF-κB pathway	N/A
**[14]**<br>Wani et al., 2019	Alzheimer's disease	Knocked-down PTEN by siPTEN or overexpression of PTEN by PTEN wild-type plasmid; or AKT by Myr-AKT-delta4-129 mutant plasmid	Alborixin (125 nM) in presence or absence of bafilomycin A1	PTEN<br>AKT	• Level of LC3B-II and SQSTM1 is stable after alborixin administration when PTEN was knocked down<br>• ↑LC3B-II and ↓SQSTM1 in HMC3 with time after alborixin<br>• knock down of PTEN inhibit the clearance of Aβ induced by alborixin<br>• alborixin clears Aβ by inhibition of AKT pathway though ↑PTEN and induced autophagy	Article contain the autophagy mechanism in N9 and N2a cells and *in vivo* expermients

(Continued)

10.3389/fimmu.2026.1778798

SUPPLEMENTARY TABLE 1 Continued

ARTICLE	MODEL	TREATMENT	DRUG TESTED	PATHWAY INVOLVED	MAIN RESULTS	NOTES
					• overexpression of PTEN leads to ↑ LC3B-II and ↓ SQSTM1, also bafilomycin A1 multiply the autophagy effect<br>AKT-overexpressing cells show ↑SQSTM1 what inhibit autophagy in alborixin treated cells	
**[15]** Ahmed et al., 2019	Viral infection (VEEV)	TC-83 or Trinidad donkey (TrD) strain of VEEV infection with<br>pretreatement with host defense peptide LL-37 (preLL-37), or LL-37<br>treatment administered at the time of infection	N/A	Viral replication	• The higest non-toxic and effective dose of LL-37 = 10 µg/mL.<br>• LL-37 leads to ↓TC-38 and TrD titer, and preLL-37 cause ↓TrD titer<br>• ↑ IFNβ1<br>• inhibiting viral replication in not cell type-dependent pattern<br>LL-37 and pre-LL-37 ↓genomic RNA copies	Pre-treatment started 30min before infection.<br>The experiment include also mouse brain microglia (BV2, EOC 20 CRL-2469), human astrocytoma (U87MG, HTB-14), and human microglia (HMC3, CRL3304) cell lines.
**[16]** Argenziano et al., 2019	Alzheimer's disease	High resolution chromatin conformation capture-based techniques	N/A	N/A	Contacts to<br>"open" promoters for RTFDC1 at the 'CASS4' locus and MADD and PACSIN3 at the 'CELF1' locus of target genes	Part of genome-wide association studies (GWAS).<br>High resolution Capture-C based target both ends of DpnII restriction fragments that overlap promoters of both protein-coding and noncoding transcripts, totaling 36,691 RNA baited ragments.
**[17]** Rai et al., 2019	Microglial cells comparison	N/A	N/A	N/A	HMC3 were negative for CD11b, CD45, CX3CR1, CD4, CXCR4.	Comparison of microglia-specific markers between PM, HMC3, C20, induced microglia (iMG) and induced microglia-like cells (iMGL).
**[18]** Kim et al., 2019	Neuroinflammation	Manganese (Mn)<br>GSK2578215A (GSK)	N/A	Leucine-rich repeat kinase 2 (LRRK2), MAPK-ERK	• ↑LRRK2, phosphorylated LRRK2 and LRRK2 mRNA in Mn-induced cells<br>• ↑viability and ↓apoptosis in GSK pre-treated Mn-induced cells<br>• ↓Bax, Daxx expression, ↓ROS and TNFα in GSK pre-treated Mn-induced cells<br>• ↑phosphorylation of MAPK p38, ERK and JNK in Mn-induced cells<br>↓phosphorylation of MAPK p38, ERK in GSK pre-treated Mn-induced cell	Pre-treatment with GSK (1 µM) for 90 min, after HMC3 were exposed to Mn (250 µM).<br>The article describes also role of LRRK2 in Mn-induced toxicity using LRRK2-wild-type (WT) and LRRK2-knockout (KO) RAW264.7
**[19]** Chen et al., 2019	Alzheimer's disease	Normal physiological glucose or HG.<br>BIX021895 (Bix, inhibitor of ERK5);<br>transfection with pcDNA3-MEK5DD-HA (MEK5DD)	N/A	ERK5, microglia M1/ M2 polarization	• ↑TNFα, IL-12, IL-6 and iNOS in HG medium<br>• ↑Arg-1, IL-10 and CD206, after 144 h ↓Arg-1, IL-10 and CD206 in HG medium<br>• ↑pERK5/ERK5, after 48 hours	Normal physiological glucose (80 mg/dl) or HG (350 mg/dl), for 288 hours.<br>Article also contain isolated CM from NG- and HG- cultured microglia added into a neuronal cell line HCN-2 in hypoxic conditions

(Continued)

10.3389/fimmu.2026.1778798

SUPPLEMENTARY TABLE 1 Continued

ARTICLE	MODEL	TREATMENT	DRUG TESTED	PATHWAY INVOLVED	MAIN RESULTS	NOTES
					↓pERK5/ERK5 in HG medium<br>• pERK5/ERK5, Arg-1 and CD206 stable in normal glucose and HG + Bix medium<br>• ↑pStat3/Stat3 ratio in HG medium<br>• ↑TNFα, IL-12, IL-6 and iNOS bigger for HG + Bix than HG<br>• ↓TNFα, IL-12, IL-6 and iNOS after MEK5DD in HG medium<br>• ↑pERK5/ERK5, IL-10, CD206 and Arg-1 after MEK5DD in HG medium<br>HG medium induces a polarization from M2a to M2b, and subsequently to M1	
**[20]**<br>Risner et al., 2019	Viral infection (VEEV)	vaccine strain VEEV TC-83, and the wild-type VEEV Trinidad donkey strain	celecoxib, rolipram, tofacitinib	COX-2, PDE4, JAK-STAT	• ↓TC-83 and TrD viral titer and ↓extracellular mRNA TC-83 after drugs pre-incubation<br>• ↓TC-83 viral titer when each of drugs were administered 2 h prioir to infection<br>• ↓TC-83 by Celecoxib as the most effective, and dose-dependent drug<br>↑mRNA levels of IL1A., IL17F, TNFα., CCR4, CCR8 and CX3CL1 after TC-83 injection, but Celoxim administered 2 h later ↓mRNA levels	24 h pre-treatment with celecoxib, rolipram and tofacitinib at dose 50 μM. 0.1% DMSO.<br>The work also includes astrocyte U87 MG cell line
**[21]**<br>Celis et al., 2019	Alzheimer's disease	pGL4.10 and pGL4.24 vectors with rs71352238 (T, C) or rs769449 (A, G)	N/A	N/A	• ↑promoter activity rs71352238 than the empty vector<br>• ↑transcriptional activity of allele C than T of rs71352238<br>The rs769449 posses enhancer activity than empty vector (no difference between the alleles)	Reporter gene assay:<br>rs71352238 (promoter in the TOMM40 gene) and rs769449 (predicted enhancer in brain tissue, APOE gene) cloned into pGL4.10 (without promoter) and pGL4.24 (with minimal promoter) vector, respectively.<br>U-118MG and SH-SY5Y were also used.
**[22]**<br>Lepiarz-Raba et al., 2019	Neuroinflammation	TNFα or IFNγ or pre-incubation with BAY 11-7082 (inhibitor of κB kinase) and IFNγ	N/A	NF-κB p65, ERK1/2	• ↑NF-κB p65, ERK1/2 and p38 phosphorylation with IFNγ or TNFα<br>• ↓NF-κB p65 phosphorylation BAY 11-7082 after IFNγ<br>• sion<br>↑TLR9 > TLR4 expression, ↑IL-6 but ↓Iba1 with IFNγ or TNFα	IFNγ (10 ng/ml) or TNFα (25 ng/ml) at different time points. In order to confirm NF-κB activation cells were pre-incubated with BAY 11-7082 for 30 min and subsequently stimulated with IFNγ
**[23]**<br>Voelz et al., 2020	Neuroinflammation	LPS, hypoxia	N/A	N/A	↓viability after 2.5 h of Oxygen-glucose deprivation (OGD), the cells displayed an increased toxicity compared to<br>No effect on LDH after OGD/R	hypoxic chamber with < 0.1% oxygen

(Continued)

10.3389/fimmu.2026.1778798

10.3389/fimmu.2026.1778798

SUPPLEMENTARY TABLE 1 Continued

ARTICLE	MODEL	TREATMENT	DRUG TESTED	PATHWAY INVOLVED	MAIN RESULTS	NOTES
					↑ROS after OGD<br>↑Hif1a, Il1b after OGD<br>↓Hif1a, Il1b after OGD+R<br>No effect on Hif1α after LPS<br>↑Il1b after LPS<br>↑miR-223-3p and miR-124-3p after hypoxia<br>↑Drosha, Ago1, Ago2, Ago4 after OGD<br>↓Drosha after OGD/R<br>↑Dgcr8, Dicer, Tarbp2, Ago2 after LPS<br>↓Xpo5 after OGD/R	
**[24]**<br>Tiwari et al., 2020	Viral infection (Epstein-Barr Virus (EBV))	EBV	N/A	N/A	5 major Raman peaks wavenumber ranges of 547–560, 1097–1109, 2047–2054, 2669–2676, and 3825–3840 cm–1 in nucleus and periphery<br>↑glucose and lipids in the nucleus at 0-2 hpi<br>↑PIP, DNA, and its phosphodiester backbone in the periphery 0-2 hpi<br>↑polysaccharide in the periphery 4-12 hpi<br>↑levels of DNA and its phosphodiester backbone, glycogen, nucleotides (guanine and cytosine), and amino acids such as proline and tyrosine in nuclei 6-12 hpi<br>24 to 36 hpi, the activity of<br>↑amide III, nucleotides of DNA (guanine, cytosine, adenine, and thymine), and fatty acids in the nucleus 24-36 hpi<br>↑cholesterol and polysaccharides was highest in the peripheral 24-36 hpi	N/A
**[25]**<br>Voelz et al., 2020	Neuroinflammation (Ischemia)	OGD/R, H2O2	N/A	N/A	↑HIF-1α, DROSHA, DICER1, AGO2 mRNA and protein after OGD/R 2.5h or H2O2<br>↑XPO5, TARBP2 after H2O2<br>↑DGCR8, colocalization of AGO2 with stress granules (G3BP1) after OGD/R	H2O2 100 µM for 24 h
**[26]**<br>Chiu et al., 2020	Polyglutamine mediated diseases	NC009-1, AM404, VB-037, LM-031), IFN-γ	N/A	IκBα/P65, JNK/JUN and/or P38/STAT1	↓NO, IL-1β, TNFα, IL-6, CD68 after IFN-γ+NC009/AM404/VB-037/LM-031<br>EC50 = 141 µM for NC009-1/VB-037<br>EC50 = 100 µM for AM404/LM-031	retinoic acid-differentiated ATXN3/Q75-GFP SH-SY5Y cells inflamed with IFN-γ-primed HMC3 CM

(Continued)

SUPPLEMENTARY TABLE 1 Continued

ARTICLE	MODEL	TREATMENT	DRUG TESTED	PATHWAY INVOLVED	MAIN RESULTS	NOTES
**[27]** Su et al., 2020	Cancer	MRX-2843	N/A	N/A	↓viability, IL-8 after MRX-2843<br>↑CD206, migration after MRX-2843<br>No effect on migration in pro-/anti-inflammatory HMC3 after MRX-2843 ± CM_U251	CM from U251. 100 nM of MRX-2843
**[28]** Landgraf et al., 2020	Neuroinflammation (Reperfusion injury and stroke)	Ebselen (EBS), EBS derivatives (5, 9, 23, 27), LPS	N/A	N/A	↓NO, TNFα after LPS + EBS/EBS derivatives<br>↑viability after LPS + EBS/EBS derivatives	EBS and EBS derivatives (5, 9, 23, 27) 10 µM, after LPS for 24 h
**[29]** Flak et al., 2020	Cancer	AT101-loaded GMO cubosomes	N/A	N/A	↑cytotoxicity after cubosomes (IC50 = 32.5 µg/mL)<br>↑cytotoxicity after cubosomes+AT101 (3.75 µM)	AT101, the R-(-)-enantiomer of the cottonseed-derived polyphenol gossypol
**[30]** Velez et al., 2020	Autoimmune encephalomyelitis	Angiotensin II (AngII)	losartan	N/A	↑IL-6, TNFα, NO, ROS, iNOS after AngII<br>↓IL-6, TNFα, NO, ROS, iNOS after AngII+losartan	N/A
**[31]** Benito et al., 2020	Metabolism	stable-isotope (13C) of β-Hydroxybutyrate (BHB)	N/A	N/A	↑Import and oxidise BHB by HMC3<br>↑Lactate and pyruvate after 13C4-BHB +glucose<br>↑NADH : NAD+ ratio after glucose<br>↑Glutamate after 13C4-BHB	5 mM 13C-labelled BHB (13C4-BHB) no added glucose, or 1 or 5 mM of unlabelled glucose (12C6-glucose)
**[32]** Nonaka et al., 2020	Alzheimer's disease	P. gingivalis, PAR2 antagonist peptides, PP2, β-arrestin-1 siRNA, β-arrestin-2 siRNA, U0126, KYT1, KYT36, GRSLIGKV, SLIGKV, GVTVE	N/A	N/A	↑Membrane ruffling, migration after P. Gingivalis<br>↓Membrane ruffling after *P. gingivalis* + (KYT1+KYT36)/PAR2 antagonist peptides/PP2/β-arrestin-1/β-arrestin-2 siRNA/U0126 or PAR2 synthetic peptides<br>↓Migration after P. Gingivalis+KYT1/KYT36	KYT1 + KYT36 (1 µM of each), PAR2 antagonist peptides (10 µg/mL), PP2 (0.2 mM), β-arrestin-1 and β-arrestin-2 siRNA (80 nM), and U0126 (15 µM). PAR2 synthetic peptides GRSLIGKV, SLIGKV, GVTVE
**[33]** Vasconcelos et al., 2020	Cancer	LPS	nanocapsules with lycopene-rich extract from red guava (nanoLEG),	N/A	↓ROS after LPS + nanoLEG 20min<br>↑NF-κB inhibition after LPS + nanoLEG	poly-ε-caprolactone lipid-core nanocapsules with lycopene-rich extract from red guava (Psidium guajava L.)
**[34]** Hankittichai et al., 2020	Neuroinflammation	IL-1β, LY294002 and U0126 (inhibitor of MEK1 and MEK2)	Oxyresveratrol (OXY)	PI3K/AKT/p70S6K, ERK1/2 MAPK	↓IL-6 and MCP-1 after IL-1β+OXY<br>No effect on TNFα, CXCL10, ROS, NO, iNOS, p-JNK after IL-1β alone or IL-1β+OXY<br>↓p-AKT, ERK1/2, MAPK, p-p38 after IL-1β+OXY 4 h<br>↓p-AKT, ERK1/2, IL-6, MCP-1 after IL-1β+LY294002/U0126	N/A

(Continued)

10.3389/fimmu.2026.1778798

SUPPLEMENTARY TABLE 1 Continued

ARTICLE	MODEL	TREATMENT	DRUG TESTED	PATHWAY INVOLVED	MAIN RESULTS	NOTES
**[35]**<br>Li et al., 2020	Cancer<br>(Glioma)	protein tyrosine phosphatase receptor-δ (PTPRD)	N/A	IL1RAP	↑PTPRD in cytoplasm	HMC3 cell line was used as a control cell. Glioma cell lines M059 J and U373 were used as cell models.
**[36]**<br>Jin et al., 2020	Alzheimer's disease	AAV-pTMEM119-shDNMT1	N/A	N/A	↓M1-associated iNOS, TNFα, IL-1β, IL-6, and IFNγ after AAV-pTMEM119-shDNMT1<br>↑M2-associated ARG after AAV-pTMEM119-shDNMT1	N/A
**[37]**<br>Nasi et al., 2020	Neuroinflammation	mitochondrial (mt)-derived damage-associated molecular patterns (MTDs), mtDNA, LPS, (IFN)-γ,	N-formyl peptides and cardiolipin (CL),	N/A	No effect on HLA-DR, TNFα, IL-6, TLR9, FPR2, JNK, viability after MTDs<br>↓ERK2, SPHK1 after CL<br>↓IL-1β after mtDNA<br>↑p38, ICAM1 after CL/fMLP<br>↑cellular oxidative stress after mtDNA/CL<br>no effect on mt ROS O2− after mtDNA/CL	N/A
**[38]**<br>dos Reis et al., 2020	Viral infection (Human Immunodeficiency Virus 1 (HIV-1))	3D human brain organoid (hBORG), HIV-1 NL(YU2-Env)-EGFP	N/A	N/A	30% infected cells after HIV-1 NL(YU2-Env)-EGFP 3 days<br>50% attached cells to hBORG 24 hpi<br>↑TNFα, IL-1β in HIV-1+hBORGs	
**[39]**<br>Angel-Ambrocio et al., 2020	Viral infection (Rhinovirus)	Huma Rhinovirus A (HRV)	N/A	N/A	↑number of copies, HRV genome after HRV 18 hpi<br>No cytopathic effect after HRV	N/A
**[40]**<br>Zhao et al., 2020	Alzheimer's disease	T0901317 (agonist of liver x receptor)	AZ7235 (robust apoE activity)	N/A	↑ApoE, ABCA1 after AZ7235<br>No effect on ApoE after T0901317	1 µM T0901317, 3 µM AZ7235 72 h
**[41]**<br>González-Prieto et al., 2020	Alzheimer's disease	Noradrenaline (NA), CX3CR1 promoter expression plasmids	N/A	N/A	↓CX3CR1 after NA<br>↓Promoter activity after NA + CX3CR1-plasmid	NA (0–50 µM) 24 h.
**[42]**<br>Tsilioni et al., 2020	Autism Spectrum Disorder (ASD)	IL-37b, IL-38 analogs (aa2-152, aa5-152, aa3-152),	neurotensin (NT) peptide	N/A	↓CXCL8 after IL-37/IL-38 analogs<br>↑CXCL8 after NT	human recombinant IL-37b (100 ng/mL) and the human recombinant IL-38 analogs (aa2-152, aa5-152, aa3-152, 100 ng/mL) for 24 h, then NT (10 nM) for 24 h
**[43]**<br>Huynh et al., 2020	Cancer<br>(GBM)	Co-culture with U87 and primary astrocytes	N/A	N/A	No changes in size, composition for HMC3 spheroids<br>Uniformity in shape, viability for HMC3 spheroids<br>↑Denser spheroids, immune cell infiltration with HMC3	U87, HMC3 and primary astrocytes (100:0:0, 0:100:0, 0:0:100, 50:50:0, 40:0:60 and 30:30:40) using the liquid overlay method.

(Continued)

10.3389/fimmu.2026.1778798

SUPPLEMENTARY TABLE 1 Continued

ARTICLE	MODEL	TREATMENT	DRUG TESTED	PATHWAY INVOLVED	MAIN RESULTS	NOTES
					↑GBM-related genes (vimentin, GFAP, MMP2, TIMP1, CCL2, SSP1, CHI3L1 and GPNMB) in heterogeneous spheroids<br>↓hypoxic core in the spheroids with HMC3	
**[44]**<br>Qiao et al., 2020	Viral infection (SARS-CoV-2)	SARS-CoV-2	N/A	N/A	↓ACE2 mRNA after SARS-CoV-2<br>↑CD147 mRNA, TMPRSS2 mRNA and protein after SARS-CoV-2	N/A
**[45]**<br>Schmitt et al., 2020	Cancer (GBM)	Co-culture with GBM and SVGA	TMZ, AT101 (antagonist of anti-apoptotic proteins like Bcl-2, BH3 mimetic)	N/A	↑SVGA to 80%<br>↓HMC3 to 10%<br>↑Viability in co-culture after TZM +AT101/AT101<br>No effect on HMC3 in monoculture after TMZ + AT101/AT101	Model consist of non-tumor cells 70% with 29% HMC3 and 1% of primary GBM<br>50 µM TMZ and 2.5 µM AT101 for three days, followed by 2.5 µM AT101 without additional TMZ
**[46]**<br>Schmitt et al., 2020	Neuroinflammation	LPS	Liposomal curcumin	N/A	↓Viability after 10 µM free curcumin or 1 µM liposomal curcumin or 90 µg/mL empty liposomes<br>No effect on IL1β, IL6, TNFα, TGFβ after LPS + curcumin/liposomal curcumin	80.6% (w/v) DPPC, 15.6% (w/v) cholesterol and 3.8% (w/v) curcumin 24 h, 100 ng/mL LPS
**[47]**<br>Schmitt et al., 2020	Cancer	graphene oxide (GO), reduced graphene oxide (rGO),	curcumin	N/A	↓Viability after GO/rGO<br>↑growth rate after curcumin + GO/rGO<br>↑IL6, TNFα after GO/rGO 24 h<br>↓IL6, TNFα after curcumin +GO/rGO 24 h	1 µm curcumin
**[48]**<br>Munoz-Pinto et al., 2020	Alzheimer's disease	interpenetrating polymer networks (mIPNs): Hyaluronic acid low or high MW, poly (ethylene glycol) diacrylate (PEGDA), collagen type I (Col I)	N/A	N/A	↓viability (85%) after mIPNs 85%<br>↑M1 markers and M2 markers iNOS, IL-1β, and CD206 after reduction in HAH in mIPNs day 3	mIPNs; PEGDA, Col I, and HA of high (HAH, ~1.5 MDa) or low (HAL, ~40 kDa)
**[49]**<br>Pan et al., 2020	Neuroinflammation	LPS, ATP, Heat shock protein (HSP27, HSP60)	N/A	N/A	↑IFNγ after LPS + ATP or alone<br>↑IFNγ and TNFα after HSP27/HSP60<br>Short dendritic processes and enlarged round cell body size after LPS/HSP27/HSP60	10ug/ml HSP27 or HSP60, 200ng/ml LPS or 100ng/ml LPS with or without 5mM ATP 30 minutes
**[50]**<br>Chen et al., 2020	Cancer (GBM)	GelMA hydrogels, co-culture with GBM12-seeded hydrogels (GBM-MG)	N/A	JAK/STAT	↑Smeboid shape, CD68 in GBM-MG<br>3409 DE genes (1563 upregulated and 1846 downregulated) in GBM-MG<br>↓NOD, NLRs, TNF, NF-κB, TLRs, MAPK, focal adhesion pathway, invasion in GBM-MG<br>↑JAK-STAT, central carbon metabolism,	N/A

(Continued)

10.3389/fimmu.2026.1778798

SUPPLEMENTARY TABLE 1 Continued

ARTICLE	MODEL	TREATMENT	DRUG TESTED	PATHWAY INVOLVED	MAIN RESULTS	NOTES
					FOXO, AMPK, EGFR tyrosine kinase inhibitor resistance in GBM-MG	
[51] Chen et al., 2020	Cancer	CM from CLOCK-depleted GSC272, CM from BMAL1-depleted GSC20, CM from CLOCK-overexpressed hNSCs, CM from CLOCK-OE GSC17, OLFML3-OE, LGMN-OE, LIPA-OE, CM OLFML3-depleted GSC272	N/A	N/A	↓Migration in CM from CLOCK-/BMAL-1-/OLML3 depleted GSC272<br>↑migration after CM CLOCK-overexpressed hNSCs/GSC17<br>↑CX3CR1, TMEM119 after OLFML3-OE/LGMN-OE/LIPA-OE	N/A
[52] An et al., 2020	Alzheimer's disease	Aβ42	PL201	NF-κB	↓IL-6, TNFα, MCP-1, IL-1β, iNOS, COX-2 after Aβ42+PL201<br>↑Nrf2 after PL201 dose-dependent 1 h<br>↑HO-1 afer PL201 6 h<br>↑p65 nuclear translocation after Aβ42 +PL201	PL201 (10–300 µM). 300 ng/ml LPS, or 3 µM oligomer Aβ42
[53] Venkateswarlu et al., 2020	3D imaging	electrospun meshes	N/A	N/A	Method used to elucidate correlations between calcium signatures and cell phenotype/activation and facilitate the rational design of scaffolds for biomedical applications.	HMC3 cells on randomly oriented electrospun fibers were stained with a fluorescent dye and imaged using a laser scanning confocal microscope
[54] Higuchi et al., 2020	Alzheimer's disease	[3H]-deltorphin II (a heptapeptide), fluorescence-labeled Aβ1–42, NaCl, iron	N/A	N/A	↑Na+-coupled deltorphin II after [3H]-deltorphin II<br>↓Fluorescence-labeled Aβ1–42 after delltorphin II<br>↑Fluorescence-labeled Aβ1–42 after Na+<br>↓Na+-coupled deltrophin II after Aβ1–42<br>↑Na+-coupled deltrophin II after Aβ1–42+iron	NaCl buffer (pH 7.5) with 1 mM Gly-Gly-Ile (NaCl+1 mM GGI)
[55] Dyne et al., 2020	Alzheimer's disease	magnetic nanoparticle (MNP), Aβ1–42, alternating magnetic field (AMF)	N/A	N/A	↑disaggregation of Aβ after MNP dose-dependent<br>↑IL-1β and TNF-α, in after Aβ<br>↓IL-1β and TNF-α, in after Aβ+MNP/AMF<br>↑Aβ after Aβ+MNP/AMF	Aβ1–42 16 h, (1-3 mg/mL) of MNPs (20 nm, paramagnetic nanoparticles) 2 h, AMF for 10 min of 30 kA/m.
[56] Zhang et al., 2020	Cancer	Ad5-Ki67/GFP, Ad5-Ki67/IL-15,	N/A	N/A	Ad5-Ki67/GFP, Ad5-GFP do not infect HMC3	Ad5-GFP, Ad5-Ki67/GFP and Ad5-Ki67/IL-15, MOI = 40) for 12 h,
[57] Ma'arif et al., 2020	Neuroinflammation	IFN-γ, genistein (positive control)	extract of *M. crenata* leaves	Arginase-1 (Arg1) ERβ	↑Arg1 and ERβ by IFN-γ<br>↓Arg1 and ERβ at dose 250 ppm of extract after IFN-γ<br>Arg1 and ERβ expression have a weak	The 96% ethanol extract was added with various doses of 62.5, 125, and 250 ppm. genistein, 50 µM, was used as a positive control.

(Continued)

10.3389/fimmu.2026.1778798

SUPPLEMENTARY TABLE 1 Continued

ARTICLE	MODEL	TREATMENT	DRUG TESTED	PATHWAY INVOLVED	MAIN RESULTS	NOTES
					negative relationship with the Pearson correlation test.	
**[58]** Ma'arif et al., 2020	Neuroinflammation	IFN-γ or genistein (positive control)	n-butanol fraction of *M. crenata* leaves	MHC II	The n-butanol fraction of *M. crenata* leaves has antineuroinflammatory activity due to its phytoestrogens that inhibit MHC II expression in the microglia HMC3 cell line	N/A
**[59]** Gaamouch et al., 2020	Alzheimer's disease	human TLQP-21 or C3aSA	N/A	VGF-TLQP-21, C3aSA	• ↑immediate early gene c-Fos: Trim47, Dusp18 Arl13b, Lmna, Furin and Mtmr10 after hTLQP-21 or C3aSA treatment<br>↑phagocytosis after hTLQP-21 treatment	Experiments were conducted also on BV2, primary microglia isolated from wild-type or C3aR1-null mice and<br>*In vivo*: on 5xFAD mice.
**[60]** Wang et al., 2021	Alzheimer's disease	miR-20b-5p, antagomiR,	N/A	N/A	↓APP after miR-20b-5p mimic<br>↑APP after antagomiR or antagomiR +miR-20b-5p mimic	MiR-20b inhibitor (antagomiR) 100 nM,
**[61]** Wang et al., 2021	Neuroinflammation	TNFα, cromolyn, F-cromolyn	N/A	PI3K/Akt/ mTOR, NF-κB, GSK-3β	↑fibrosis-associated genes, collagen XVIII alpha-1 (COL18A1), ECM component Tenascin-c (TNC), TTL, PROX1, Rab35, CSDE1, PIK3CD after TNFα<br>↓fibronectin, PLP1 after TNFα<br>↓KRT9, KRT1, KRT5 after cromolyn +TNFα<br>↓collagen XVIII, fibronectin, tenascin-c, PLP1, PELP1, HSP90, IL-2, GRO-α, Eotaxin, and VEGF-A after TNFα+cromolyn/F-cromolyn<br>↑IL-4, TTL, PROX1, Rab35 after TNFα+cromolyn/F-cromolyn<br>↓CSDE1, PIK3CD after TNFα+cromolyn/F-cromolyn	TNFα (0.3 μg/ml) and/or Cromolyn (0.3 μM, 3 μM, 10 μM, 30 μM) or F-cromolyn-diacid (0.3 μM, 3 μM, 10 μM, 30 μM) for 24 h.
**[62]** Wang et al., 2021	Cancer	Co-culture with U251/U87, sh-P4 hA1	N/A	N/A	↓Migration after sh-P4 hA1 in co-culture<br>↑TNFα and IFN-γ M1 phenotype after sh-P4 hA1 in co-culture	N/A
**[63]** Fernandes et al., 2021	Ocular disease	NEs F8 and F9	N/A	N/A	↓Viability after NEs, dose-dependent<br>↑DNA damage after F8<br>↓DNA damage after F9	200 μL of medium containing different concentrations of TA or 200 μL of NEs F8 and F9 (5%, 10% and 20%) for 24 h.
**[64]** Keane et al., 2021	Cancer	siEZH2	N/A	N/A	↑Migration capacity, phagocytose after siEZH2 in co-culture with H3-K27M SF8628 DIPG/H3 SF188 pHGG	N/A

(Continued)

10.3389/fimmu.2026.1778798

SUPPLEMENTARY TABLE 1 Continued

ARTICLE	MODEL	TREATMENT	DRUG TESTED	PATHWAY INVOLVED	MAIN RESULTS	NOTES
**[65]** Kwon et al., 2021	Microplastic (MP) pollution	polystyrene MPs (PS-MPs)	N/A	N/A	↑PS-MPs cytosol deposit after PS-MPs 0.2/2 μm dose-dependent<br>↓G0/G1 phase, Bax, cleaved-PARP, cleaved-Caspase 8, cleaved-Caspase 3, IL-1β, CCL2, TGF-β, NEAT1, MIR1290, MIR4694, MIR4668, MIR4532, MIR4703, MIR3165 after PS-MPs 0.2/2 μm<br>↑M phase, phagocytosis, amoeboid shape, Iba1, CD68, CD16, CD206 STAT3, NF-κB, STAT5A, BEFB132, IGKV1-12, IGHV3-38, IGHV3-37, IGLC2, LGHM, IGLV1-40, IGLC7, MIR4713, MIR4269 after PS-MPs 0.2/2 μm	PS<br>MPs of different sizes (0.2, 2, and 10 μm) (1, 5, and 10 μg/mL) 24 h
**[66]** Agil et al., 2021	Neuroinflammation	Genistein, FNγ, Marsilea crenata Presl. leaf extract	N/A	N/A	↑MHC II, 148.632 AU after IFNγ+Marsilea crenata Presl. leaf extract dose-dependent<br>ED50 = 1,590 ppm for Marsilea crenata Presl. leaf extract	62.5, 125, and 250 ppm, to previously induced by IFNγ 10 ng for 24 hours to
**[67]** Yang et al., 2021	Cancer (GBM)	N/A	N/A	PI3K/AKT	↓Williams syndrome transcription factor (WSTF) in HMC3	HMC3 used only for the comparison
**[68]** Lee et al., 2021	Age-related macular degeneration (AMD)	gypenoside (Gyp LXXV) from *Gynostemma pentaphyllum*	N/A	N/A	↓TNFα, IL-1β, IL-6 after Gyp LXXV	1 μM Gyp LXXV
**[69]** Hernández-Ochoa et al., 2021	Cancer (Pediatric glioma)	N/A	N/A	N/A	↑G6PD expression (CT=14), PFKM, GAPDH, and ACACA genes, (CT=17~23)<br>↓HK1, TPI1, PKM, LDHAL6A, PGD1, TKT1, SDHB, FASN, ELOV2, GAPDHa, GAPDHb, and TBP (CT=26~34)<br>↓gene variation for PKM, TPI1, GAPDH, and GAPDHa genes<br>↑HK1, PFKM, GAPDH, LDHAL6A, ACACA, ELOVL2 in GBM samples comparing to HMC3	N/A
**[70]** Li et al., 2021	Neuroinflammation	Glucagon-like peptide (GLP-1) (9–36), LPS	N/A	N/A	↓IL-6, TNFα after LPS + GLP-1 (9-36)<br>↑Iba1 and P2Ry12 after GLP-1 (9-36)	Preincubation with 1 μM GLP-1 (9–36)
**[71]** Salsinha et al., 2021	Neuroinflammation	fructose, saturated fatty acid PA, polyunsaturated fatty acids (omega-3 – EPA and DHA- and CLA	N/A	GPR120/FFA4	↑NF-κB pathway activation, ROS after fructose+ saturated fatty acid PA<br>↓NF-κB pathway activation after fructose + saturated fatty acid PA +	N/A

(Continued)

10.3389/fimmu.2026.1778798

ARTICLE	MODEL	TREATMENT	DRUG TESTED	PATHWAY INVOLVED	MAIN RESULTS	NOTES
		and CLNA isomers), agonists and antagonists of GPR120/FFA4			polyunsaturated fatty acid<br>↓ROS after fructose + saturated fatty acid PA + omega-3/CLA<br>Modulation of GPR120 after CLA/CLNA	
**[72]**<br>Salsinha et al., 2021	Obesity	(Palmitic Acid (PA), a Saturated Fatty Acids (SFA), and<br>fructose) and the preventive role of different Poly Unsaturated Fatty Acids (PUFAs) (Omega-3 – Eicosapentaenoic Acid (EPA) and Docosahexaenoic Acid (DHA), Conjugated Linoleic Acid (CLA) and Conjugated Linolenic Acid (CLNA) isomers)	N/A	N/A	↑IkBa degradation after CLA/CLNA + frut + PA time dependent<br>↓ROS after CLA/Omega-3 + fructose + PA<br>↑LynSrc activation after fructose + PA or CLA/Omega-3 + fructose + PA<br>↓Src activation after CLNA/Omega-3 +frut+PA	N/A
**[73]**<br>Cao et al., 2021	Neuroinflammation (Cerebral ischemia-reperfusion injury)	OGD/R, CHRFAM7A-OE, Nigericin sodium salt,	N/A	NLRP3/ Caspase-1	↓CHRFAM7A after OGD/R<br>↑proliferation, LDH activity, Arg1, M2 phenotype after OGD+ CHRFAM7A-OE<br>↓TNFα, IL-1β, IL-6, NLRP3, pyroptotic cells (Caspase-1, GSDMD-N, IL-1β, and IL-18), iNOS after OGD+ CHRFAM7A-OE<br>↑Caspase-1, GSDMD-N, IL-1β, IL-18, LDH activity after Nigericin sodium salt<br>↓Caspase-1, GSDMD-N, IL-1β, IL-18, LDH activity after Nigericin sodium salt + CHRFAM7A-OE<br>↓viability after Nigericin sodium salt	N/A
**[74]**<br>Li et al., 2021	Cancer	AEG-1-KD, co-culture withU251/U87	N/A	N/A	↓M2 polarization (CD206, CD163), IL-6 and TGF-β1 after shAEG-1 in co-culture<br>No effect on TNFα and IFN-γ after shAEG-1 in co-culture	N/A
**[75]**<br>Pallio et al., 2021	Neuroinflamation	IL-1β	metaxalone	N/A	↑TNFα, IL-6, NF-κB, MAO-A expression/activity and malondialdehyde level after IL-1β<br>↓IL-13, PPARγ, PGC-1α, Nrf2 after IL-1β<br>↓MAO-A activity/expression, NF-κB, TNFα, IL-6 after IL-1β+metaxalone<br>↑IL-13, PPARγ, PGC-1α, Nrf2 after IL-1β+metaxalone	metaxalone (10, 20, and 40 µM) 6 h, IL-1β stimulation 5 ng/mL 1 h
**[76]**<br>Hou et al., 2021	Alzheimer's disease	Aβ42, NR, H-151, cGAS-KD, STING-KD, etoposide, irradiation	N/A	cGAS-STING	↑IL-6 after Aβ42<br>↓IL-6 after Aβ42+NR<br>⟳IL-6 after Aβ42+H-151<br>No effect on IL-6 after Aβ42+H-151+NR	Aβ42 (5 µM) and/or NR (1 mM) and/or STING inhibitor (H-151) or cGAS-KD and/or STING-KD for 48 h. Irradiation (IR) 3 Gy, followed by 9 d in culture.

(Continued)

10.3389/fimmu.2026.1778798

SUPPLEMENTARY TABLE 1 Continued

ARTICLE	MODEL	TREATMENT	DRUG TESTED	PATHWAY INVOLVED	MAIN RESULTS	NOTES
					or Aβ42+cGAS-KO/STING-KO+NR<br>↓senescence (SA-β-gal) after etoposide +NR/cGAS/STING-KO/H-151<br>↓senescence after IR+NR<br>No effect on senescence after IR+NR +cGAS/STING-KO	
**[77]**<br>Ho et al., 2021	Major Depressive Disorder (MDD)	TSPAN5-KO, EtOH, acamprosate	N/A	IFN	↓Kynurenine, IRF7, IRF9, MX1, MX2, OAS1, OAS2, IFITM1, DDIT3, GRP78, STAT1 after TSPAN5-KO<br>↓TSPAN5 mRNA, kynurenine, IFN pathway (IRF7, IRF9, MX1, MX2, OAS1, OAS2, IFITM1, DDIT3, GRP78, STAT1) after EtOH or acamprosate	N/A
**[78]**<br>Lai et al., 2021	Cancer	[1-13C]pyruvate, irradiation	N/A	N/A	↓kPL [Pyr.], LDHA, LDHB, pyruvate-to-lactate flux on DNP 13C-MRI after irradiation<br>[1-13C]alanine detected in non-/irradiated<br>No effect on MCT1, MCT4 after irradiation	15 Gy X-ray irradiation for 30–79 min analyzed via DNP 13C-MRI. Pyruvate-to-lactate conversion rate (kPL [Pyr.])
**[79]**<br>Fang et al., 2021	Neuroinflammation (Intracerebral Hemorrhage (ICH))	miR-124-3p mimics, LPS, pcDNA3.1-TRAF6 (pTRAF6), si-TRAF6	N/A	TRAF6/NLRP3	↓miR-124-3p after LPS<br>↓IL-1β, IL-6, TNFα, apoptosis, TRAF6 after LPS + miR-124-3p mimics,<br>↑viability after LPS + miR-124-3p mimics<br>↓TRAF6, viability after LPS + pTRAF6 +miR-124-3p mimics<br>↑IL-1β, IL-6, TNFα, NLRP3, apoptosis after LPS + pTRAF6+miR-124-3p mimics<br>↓TRAF6, NLRP3, IL-1β, IL-6, TNFα, apoptosis after LPS + si-TRAF6	
**[80]**<br>Panda et al., 2021	Alzheimer's disease	PHD Finger Protein 6 (PHF6), aggregated PHF6 (aPHF6), MCC950 (NLRP3 inhibitor)	N/A	N/A	No effect on viability after aPHF6/PHF6<br>↑metabolic activity after aPHF6/PHF6 6 h<br>↑NLRP3 after aPHF6>PHF6 6 h<br>↑PYCARD, ASC, Caspase-1, IL-18, IL1β after aPHF6 6 h<br>↓Caspase-1 after aPHF6+MCC950<br>↑BECN1, autophagy, Beclin-1, p62, LC3I after aPHF6 6 h	6 h or 24 h of 20 μM PHF6 or aPHF6

(Continued)

10.3389/fimmu.2026.1778798

ARTICLE	MODEL	TREATMENT	DRUG TESTED	PATHWAY INVOLVED	MAIN RESULTS	NOTES
[81]<br>Ahuja et al., 2021	Cancer	N/A	coctail consisting of IL-4, IL-13, IL-10, TGFβ, and CCL2	N/A	Identification of ~10 000 proteins<br>↑mitochondrial gene, immune response, and impaired cell cycle progression.<br>Intracellular immune activation (MAPK, STAT, TGFβ, NF-KB, integrin)<br>↑collagen formation, matrix components, growth factors, proteases and protease inhibitors, ECM remodeling<br>↑antigen processing/presentation (HLA I), proteasomal subunits, vesicular/viral transport, and secretory processes.	
[82]<br>Ceylan et al., 2021	Multiple Sclerosis (MS)<br>(Autoimmune encephalomyelitis)	iron sulfate (FeSO4), tert-Butyl hydroperoxide (t-BHP)	clozapine	N/A	↓Viability after iron/clozapine >100 μM or t-BHP 800 μM 2 h<br>↑Viability after clozapine 1μM or clozapine + t-BHP 50 μM 4 h<br>↓IL-6 after iron+clozapine 10μM<br>No effect on CCL5, IL-6 after iron<br>↑ Phagocytosis after iron 10μM<br>↓ Phagocytosis after iron 100μM<br>↺ Phagocytosis after iron + clozapine	clozapine 1 h prior to t-BHP 50 μM-800 μM, iron 25 μM
[83] Nuytemans et al., 2021	Alzheimer's disease	N/A	N/A	N/A	Identification of a region in the first introns of TOMM40 with increased EUR/JPT enhancer activity	results presented at conference
[84]<br>Hill et al., 2021	COVID-19 (Neurodevelopment of Children)	SARS-CoV2 spike protein	N/A	N/A	↑IL-6 protein after SARS-CoV2 infection, dose-dependent	N/A
[85]<br>Li et al., 2021	Lyme neuroborreliosis	live Borrelia burgdorferi (Bb)	N/A	N/A	↑GAP-43 mRNA and protein after Bb	Live Bb strain 4680 or PBS for 6, 12, and 24 h
[86]<br>Chen et al., 2021	Alzheimer's disease	LPS	N/A	N/A	↑Amoeboid shape, TOMM40, TNFα, and NLRP3 mRNA after LPS treatment	LPS 10 ng/ml, 100 ng/ml, for 24 hours
[87]<br>Tu et al., 2021	Cancer (GBM)	N/A	N/A	N/A	↑Cytokines (SPP1, G-CSF, NT-3, and TNFα) and<br>↓Cytokine (ENA-78/CXCL5) in CM U87/U251 compared to HMC3	Comparison of HMC3 medium to HUVECs cultured with CM from U87 and U251 glioma cells
[88]<br>Gopalakrishnan et al., 2021	Neuroinflammation (SCI)	α-gal nanoparticles, IFN-γ	N/A	N/A	↑CD68, ameboid shape, activation, M2 phenotype, Arginase-1 and CD206 and VEGF after α-gal nanoparticles<br>↓IL-6 after IFN-γ+α-gal nanoparticles<br>No effect on TNFα, iNOS after α-gal nanoparticles	100 mg/mL α-gal liposomes 3h, IFN-γ 5 pg/mL 24 h

(Continued)

10.3389/fimmu.2026.1778798

SUPPLEMENTARY TABLE 1 Continued

ARTICLE	MODEL	TREATMENT	DRUG TESTED	PATHWAY INVOLVED	MAIN RESULTS	NOTES
[89]<br>Phitthayaphong et al., 2021	Alzheimer's disease	PA, Fc gamma receptors (FcγRs)-blocker	N/A	N/A	↑Aβ, BACE1, TNFα, IL-1β, IL-6, FcγRs activation after PA<br>↓viability after PA<br>No effect on p-Tau/Tau ratio after PA<br>↓FcγRs Aβ, BACE1, TNFα, IL-1β, IL-6, cell death after PA+FcγRs-blocker	200 and 400µM PA-conjugated BSA 24 h, FcγRs 1 h before PA
[90]<br>Chiu et al., 2021	Alzheimer's disease	IFN-γ	N/A	N/A	↑iNOS, TNFα, IL-1β, IL-6, CD68, MHCII	CM from HMC3/IFN-γ was used to treat Aβ-GFP SH-SY5Y cells.
[91]<br>Iannucci et al., 2021	Alzheimer's disease	ApoE isoforms (ApoE2/3/4)	N/A	N/A	↓TREM2, Clec7a, TNFα after ApoE2/E3<br>↑TREM2, Clec7a, TNFα after ApoE4<br>↓IL-6 after ApoE4	ApoE2, ApoE3, or ApoE4 (20 nM) 24 h
[92]<br>Das et al., 2021	Alzheimer's disease	rs242557-KO (homozygous/heterozygous),	N/A	N/A	↓Nsf, Kansl1, Kansl1-AS1, LRR37A, LRR37A2, MAPT, MAPT isoforms (3R, 4R, 0N, 1N and 2N), CRHR1, LRR37A3 mRNA after rs242557-homo/hetero-KO<br>↓NSF and KANSL1 after rs242557-homo-KO	Microtubule associated protein tau (MAPT)
[93]<br>Burton et al., 2021	Alzheimer's disease	rs6024870-KO	N/A	N/A	14 putative effector genes in microglial model after variant-to-gene mapping<br>↓RTFDC1 mRNA and protein after rs6024870<br>↑RTFDC1 mRNA and protein after rs6024870-KO	SNP rs6024870 at the 'CASS4' GWAS locus. In comparative analysis was used iPSC-derived microglia (iMg)
[94]<br>Zhang et al., 2021	Cancer	U251 VCT/PDIA5, U251 siNC/siPDIA5	N/A	N/A	↑Dimension of organoid in co-culturing with U251 PDIA5 at 10 days pt<br>↓Dimension of organoid in co-culturing with U251 siPDIA5<br>↑Viability in co-culturing with U251 siPDIA5<br>↑Proliferation and exhausted HMC3 after PDIA5<br>↓Malignant behavior of glioma cells in immune cells exhausting after siPDIA5	N/A
[95]<br>Zhang et al., 2021	Parkinson's disease	LPS, si-SNHG7, SNHG7-OE miR-425-5p mimics, rotenone	N/A	miR-425-5p/ TRAF5/NF-κB	↑MDA after LPS<br>↓SOD, GSH-PX after LPS<br>↓MDA, TRAF5, p-NF-κB after LPS + siSNHG7<br>↑SOD, GSH-PX, I-κB, Nrf2, HO-1after LPS + siSNHG7<br>↓SNHG7 WT after miR-425-5p mimics<br>No effect on SNHG7 MUT after miR-425-5p mimics	LPS (10 µg/ml), rotenone (500nM) 24 h

(Continued)

10.3389/fimmu.2026.1778798

SUPPLEMENTARY TABLE 1 Continued

ARTICLE	MODEL	TREATMENT	DRUG TESTED	PATHWAY INVOLVED	MAIN RESULTS	NOTES
					↑Ago2 after miR-425-5p mimics<br>SNHG7, LDH release after<br>↓IL-1β, IL-6, TNFα, MDA after LPS + miR-425-5p mimics ± SNHG7-OE<br>↑SOD, GSH-PX after LPS + miR-425-5p mimics ± SNHG7-OE<br>↓TRAF5 and p-NF-κB after LPS + miR-425-5p mimics<br>↑I-κB, Nrf2, and HO-1 after LPS + miR-425-5p mimics<br>↑TRAF5 and p-NF-κB after LPS + miR-425-5p mimics+SNHG7-OE<br>↓I-κB, Nrf2, and HO-1 after LPS + miR-425-5p mimics+SNHG7-OE	
**[96]**<br>Zhang et al., 2021	Cancer	TGF-β1, M-CSF	N/A	N/A	↑IL10 after M-CSF<br>No effect on CCL2 and CCL22 after M-CSF or TGFβ1	TGF-β1 (10 ng/ml), human M-CSF (10 ng/ml) or in a combination diluted in medium daily for 3 days.
**[97]**<br>Wang et al., 2021	Neuroinflammation	*Brucella suis* S2 strain (S2), HMC3-CALR, HMC3-sh-CALR	doxycycline (Dox)	JNK/p53	↑calreticulin (CALR) after S2 2 h<br>↓Apoptosis, p-ASK1, p-MEK4 and p-JNK after S2<br>↑p-JNK, p-p53 after S2 8 h<br>↑CALR after S2/CALR+S2<br>↓p-JNK, p-p53, apoptosis after CALR<br>↑p-ASK1 and p-MEK4 after sh-CALR<br>↓P-p53 after S2/CALR+S2<br>↓CALR after Dox 160μM<br>↓Viability after Dox 40μM<br>↑p-ASK1, p-MEK4, p-JNK, apoptosis after S2+Dox	N/A
**[98]**<br>Song et al., 2021	Neuroinflammation	LPS, KCNQ1OT1-KD, KCNQ1OT1-OE, miR-30e-3p mimic, pc-NLRP3,			↑KCNQ1OT1 and NLRP3 after LPS<br>↓miR-30e-3p after LPS/KCNQ1OT1-OE<br>↓LDH release, apoptosis, TNFα, IL-1β and IL-6 after LPS + KCNQ1OT1-KD or LPS + miR-30e-3p mimics<br>↑CD86 but ↓CD206 after LPS + KCNQ1OT1-OE<br>↓CD86, NO,ROS but ↑CD206 after LPS + KCNQ1OT1-KD<br>↑TNFα, IL-1β, IL-6, CD86 but ↓CD206 after KCNQ1OT1-OE+LPS + miR-30e-3p mimics or pc-NLRP3<br>↓TNFα, IL-1β, IL-6, CD86 but ↑CD206 after LPS + miR-30e-3p mimics<br>↓NLRP3 after LPS + miR-30e-3p mimics	

(Continued)

10.3389/fimmu.2026.1778798

SUPPLEMENTARY TABLE 1 Continued

ARTICLE	MODEL	TREATMENT	DRUG TESTED	PATHWAY INVOLVED	MAIN RESULTS	NOTES
					or LPS + KCNQ1OT1-KD<br>↑NLRP3 after LPS + miR-30e-3p mimics +KCNQ1OT1-OE<br>↑Apoptosis, release of LDH, NO and ROS after pc-NLRP3	
**[99]**<br>Garcia-Contreras et al., 2021	Neuroinflammation (Neurodegenerative diseases)	LPS, hAD-Mesenchymal Stem Cells (MSCs), hAD-MSC-EVs	N/A	N/A	↓iNOS, CD11b, ameboid morphology after LPS + hAD-MSCs/Evs<br>↓IL-6, IL-8, and MCP-1 after LPS + hAD-MSCs<br>↑IL-10 or TIMP-1 after LPS + EVs	hAD-MSCs (200,000, 100,000, and 50,000 cells) or their secreted EVs (50, 20, or 10 µg/ml) 24 h, 1 µg/ml LPS
**[100]**<br>Roblain et al., 2021	AMD	miR-142-3p inhibitor, miR-142-3p mimic, LPS, IFNγ	N/A	N/A	↓BCLAF1 after miR-142-3p mimic<br>↑CD68, VEGF-A after miR-142-3p mimic<br>No effect on BCLAF1, CD68, VEGF-A after miR-142-3p inhibitor<br>↑miR-142-3p, CD68 after LPS/IFNγ	N/A
**[101]**<br>Gao et al., 2021	Viral infection (ZIKV, Severe Fever with Thrombocytopenia Syndrome (SFTS) Phlebovirus, and Enterovirus A71)	ZIKV-SZ01, ZIKV-MR766, SFTSV-A, SFTSV-E, Enterovirus A71 (EV71)	fludarabine	N/A	IC50 = 0.71 ± 0.07 µM for ZIKV +fludarabine<br>IC50 = 0.42 ± 0.01 µM for SFTSV-E/-A +fludarabine<br>IC50 = 0.94 ± 0.09 µM for EV-A71 +fludarabine<br>CC50 = 12.68 ± 2.30 µM for fludarabine	N/A
**[102]**<br>Caruso et al., 2021	Alzheimer's disease	Aβ42, EX527 (selective inhibitor of SIRT1), BML-275 (AMPK inhibitor)	Melatonin, luzindole	N/A	↑IL-4, IL-13, BDNF, SIRT1 (expression, nuclear localization, activity) after Aβ42<br>↓BDNF after Aβ42+EX527 (2 µM)<br>↑SIRT1, phosphorylation of AMPK after Aβ42 30min<br>↓SIRT1 after BML-275+Aβ42<br>↑IL-1β, TNFα, NF-κB nuclear localization after EX527/BML-275<br>↑SIRT1, BDNF after Aβ42+melatonin<br>↓NF-κB nuclear translocation and acetylation Aβ42+melatonin<br>↓SIRT1, BDNF after Aβ42+melatonin +luzindole<br>↑NF-κB nuclear translocation and acetylation Aβ42+melatonin+luzindole	Aβ42; 0.2 µM) initially (6 h), EX527 (2 µM), BML-275 (2 µM), melatonin (1 µM), luzindole (25 µM)
**[103]**<br>Wu et al., 2021	Cancer	Estrogen (E2), Co-cultured 231BrM, shERα, shEzβ	tamoxifen, stattic	JAK/STAT3	↑estrogen receptor α and β, M2-related genes (Arg1, Arg2, CD206) after E2<br>↓CD86, Iba1 after E2<br>↓M2 polarizaton after E2+tamoxifen/ shERα/shERβ	Microglia's polarization by incubating human microglia cells with or without Phenol red and with or without 2% of FBS or charcoal-stripped FBS for 1 and 3 days. E2 (1 nM) and stattic (0.5 µM) or tamoxifen (1 µM) for 24 h.

(Continued)

10.3389/fimmu.2026.1778798

SUPPLEMENTARY TABLE 1 Continued

ARTICLE	MODEL	TREATMENT	DRUG TESTED	PATHWAY INVOLVED	MAIN RESULTS	NOTES
					No effect on M1/M2 polarization after phenol red/FBS/carcoal-stripped FBS<br>↑activation JAK/STAT3 after E2<br>↓activation JAK/STAT3 after E2 +tamoxifen<br>↑Arg1, STAT3, CD86 after E2 +STATTIC/tamoxifen<br>↓CD206 after E2+STATTIC/tamoxifen	
**[104]**<br>Ma'arif et al., 2021	Neuroinflammation	IFN-γ	genistein	N/A	↓MHC II after IFN-γ+genistein<br>↑Arg1, free-Erβ after IFN-γ+genistein	10 ng IFN-γ 24 h.<br>Genistein 50 µM 48 h.
**[105]**<br>Germelli et al., 2021	Neuroinflammation	Trilostane, SU-1060317 (inhibitor of 17α-hydroxylase/C17–20 lyase (P450c17 or CYP17A1))	forskolin, XBD-173, PIGA1138 (phenylindolyilglyoxylamydes), etifoxine (nonbenzodiazepine anxiolytic agent)	N/A	↑expression of StAR, TSPO (homozygous for Ala147), CYP11A1, 3β-HSD, 5α-reductase<br>↑pregnenolone after trilostane/SU-1060317/XBD-173/PIGA1138/etifoxine or serum-free + forskolin<br>↑BDNF after XBD-173/PIGA1138<br>No effect on TGF-β after XBD-173/PIGA1138	N/A
**[106]**<br>Li et al., 2021	Alzheimer's disease	elastin-like polypeptide (ELP90), Aβ42	N/A	N/A	↑extracellular Aβ and sAPPα after ELP<br>↓intracellular Aβ after ELP	100 µg/ml of ELP90 3h
**[107]**<br>Sreenivasan et al., 2021	Cancer (Medulloblastoma)	co-culture with Med8A-S cells	ruxolitinib	gp130/JAK/STAT	MB cells subjected to co-culture with microglia exhibited ↑expression of phosphorylated JAK1 and STAT3, which was correlated with enhanced resistance to vincristine<br>HMC3 cells secreted high levels of IL-6, which is likely to act in paracrine fashion in a co-culture to activate IL-6/STAT3 signaling in Med8A-S cells	N/A
**[108]**<br>Lu et al., 2021	Neuroinflammation	LPS, PA, Pam2CSK4, Pam3CSK4, LPS-RS, PD98059, SP600126, SB203580, Bay11-7082	N/A	MAPK/NFκB/P-1	↑IL-6, IL-2, IL-4, IL-8, IL-17A, IL-10, IFNγ, TNFα, CCR7, CCR2, CXCL1, CXCL2, CXCL10 after LPS + PA<br>↓CCL5, CD14, TLR4 after LPS + PA<br>↓viability after PA 400 µM<br>↓IL-6 after LPS + PA+LPS-RS/Pam2CSK4/PD98059/SP600126/SB203580/Bay11-7082<br>No effect on IL-6 after PA<br>↓MCP-1 after PA<br>↑ceramide, dihydrosphingosine, total dihydroceramide (C16-, C18-, C22-	LPS (100 ng/ml) and PA (200 µM)

(Continued)

10.3389/fimmu.2026.1778798

SUPPLEMENTARY TABLE 1 Continued

ARTICLE	MODEL	TREATMENT	DRUG TESTED	PATHWAY INVOLVED	MAIN RESULTS	NOTES
					ceramide), sphingosinesphingomyelin hydrolysis after PA alne or LPS + PA	
**[109]** Kim et al., 2021	Aging and Neuroinflammation (Stroke)	TNFα, IFNγ, OGD/R	N/A	N/A	↑IFI27L2 mRNA after TNFα+IFNγ+OGD<br>↑IFI27L2 protein in the peri-nuclear membrane and mitochondria after TNFα+IFNγ+OGD 20 h	TNFα (20 ng/ml) and IFNγ (20 ng/ml) plus OGD
**[110]** Meng et al., 2021	Alzheimer's disease	betaine, hilyte Aβ1-42 555	N/A	PI3K/AKT	↑autophagy, LC3-I/LC3-II, beclin1, ATG5, ATG7, ATG12 after betaine<br>↓SQSTM1, p-AKT, mTOR after betaine<br>↓Aβ1-42 after hilyte Aβ1-42 555 +betaine	betaine 5 mM 24 h, hilyte Aβ1-42 555 (10 mM)
**[111]** Rodriguez-Matos et al., 2021	Neuroinflammation (Hypoxic-ischemic brain injury)	Hypoxia, ET-1, BQ123	N/A	N/A	↑ROS, TNFα, IL-6, ET-1 after hypoxia<br>↑ROS, TNFα, IL-6 after ET-1<br>↓ROS, TNFα, IL-6 after ET-1+BQ123	Hypoxia chamber (1% O2, 5% CO2, and 92% N2) at 37°C 24 h
**[112]** Liu et al., 2021	Gene analysis	co-culture of iNGN,	N/A	N/A	16 common genes in co-culture and 40 HMC3-enriched genes (e.g. COL1A1, SERPINE1, AXL, FLNA, MYL9, GREM1)<br>↑PNMA3, RXRG and TRIM55 in HMC2_1<br>↓ZBED2, TERF2, HOXB4 in HMC3_1<br>Lack of NHLH1, NEUROG2, NCAM1 in HMC3_1<br>↑homo-interaction HMC3_2+HMC3_2 or HMC3_1+HMC3_1<br>↓OLIG3, NEUROD1, TLR4 in HMC3_2	barcoded oligonucleotides ligated on RNA amplified for multiplexed and parallel *in situ* analyses (BOLORAMIS). identified two sub-clusters within the HMC3 cluster,
**[113]** os Reis et al., 2021	Viral infection (HIV-1)	HIV-1	N/A	N/A	↑inflammatory response, proliferation on top of the organoids after HIV-1<br>↑cells embedded in the hBORGs after HIV-1 3day<br>HMC3 outnumber the organoids cell number, but prolong culture lasting after 10 day	Commentary article
**[114]** Kumar et al., 2021	Neuroinflammation	N/A	cocaine	N/A	↓viability, CD63, dectin-1 after cocaine<br>↑apoptotic marker histone H2A.x after cocaine<br>No effect on cleaved caspase-9/-3, Hsp70, Hsp90-β after cocaine	Cell derived EVs from conditioned medium after 1 μM, and 100 μM of cocaine 24 h

(Continued)

10.3389/fimmu.2026.1778798

SUPPLEMENTARY TABLE 1 Continued

ARTICLE	MODEL	TREATMENT	DRUG TESTED	PATHWAY INVOLVED	MAIN RESULTS	NOTES
**[115]** Akhter et al., 2021	Alzheimer's disease	Aβ42, TREM2-OE, siTREM2	N/A	TREM2	↑phagocytosis after Aβ42+TREM2-OE<br>↓phagocytosis after Aβ42+siTREM2<br>↑TREM2 after Aβ42<br>↓Bax, Bad, and pBad after Aβ42<br>↑Mcl-1/Bim Aβ42+TREM2-OE<br>↑IP-10, MIP-1a, IL-8 Aβ42+TREM2-OE<br>↓FGF-2, VEGF, GRO after Aβ42 +TREM2-OE<br>↓IP-10, MIP-1a, IL-8 Aβ42+siTREM2<br>↑FGF-2, VEGF, GRO after Aβ42 +siTREM2	5 µM Aβ42 24 h
**[116]** Rai et al., 2021	Viral infection (HIV)	IFN, HIV-1BaL, VSV-pseudotyped HIV-1,	N/A	N/A	↓CD45, CD11b<br>↑P2RY12, TREM1119, IBA-1,<br>↑CD317, CD169 after IFN<br>Similar gene expression profile of C20<br>↓HIV-1 restriction factors (SAMHD1, BST2, APOBEC3G)<br>HMC3 could not be infected by HIV-1BaL<br>↑p24 release, proliferation VSV-pseudotyped HIV-1	Comparison of iPSC-MG, MMGs, C20, HMC3 cell lines
**[117]** Wang et al., 2022	Mitochondrial proteome studies	LPS	N/A	N/A	↑NLRP3 after LPS<br>dysregulation of 744 proteins including 383 mitochondrial proteins (most down-regulated) after LPS<br>↺Mitochondrial proteins after LPS >48 h<br>↑PCK2, CHCHD2, GPT2 after LPS >48 h<br>↓PON2 after LPS >48 h	1 µg/mL LPS for 24 h and 48 h
**[118]** Richardson et al., 2022	Neuroinflammation (Fetal)	Amniotic Fluit (AF), CSE, LPS	N/A	N/A	GFAP, Iba1 and SRY in non-treated cells<br>No effect on migration after CSE or LPS<br>↑CD11b but ↓Iba1after CSE or male AF +LPS<br>No effect on HMC3 after AF+LPS or AF<br>No changes in pro-inflammatory cytokines after male AF+LPS<br>↑IL1β, CD11b but ↓Iba1after WH AF +LPS<br>↑IL-8, CD11b but ↓Iba1after AA AF +LPS	AF from male and female fetuses of White Hispanic (WH) and African-American (AA) pregnant women with or without LPS (100 ng/ml) and incubated for 48 h. SVGp12 and HMC3 were co-cultured at an 80:20 ratio

(Continued)

10.3389/fimmu.2026.1778798

SUPPLEMENTARY TABLE 1 Continued

ARTICLE	MODEL	TREATMENT	DRUG TESTED	PATHWAY INVOLVED	MAIN RESULTS	NOTES
[119] Richardson et al., 2022	Alzheimer's disease	SHIP1 inhibitors	N/A	N/A	Novel, and selective SHIP1 inhibitors have been discovered.<br>The enzyme mode of action, cellular activity, and drug-like properties were determined	
[120] Hou et al., 2022	Neurotoxicity	Nano-zinc oxide particles (N-ZnO Ps)	N/A	N/A	No effect on viability, IC50 = 287.86 μg/mL	N-ZnO Ps (6.25, 12.5, 25, 50, and 100 μg/mL) 24 h
[121] Wang et al., 2022	Cancer	N/A	N/A	N/A	↓miR-21 in HMC3 < DAOY	HMC3 used only as a comparison
[122] Ferrisi et al., 2022	Neuroinflammation	LPS/TNFα, LV62, SR144528	JR64a	N/A	↑IL-6 but ↓IL-10 after LPS/TNFα or LPS/TNFα + JR64a+SR144528 or LPS/TNFα+EC21a + LV62<br>↓IL-6 but ↑IL-10 after LPS/TNFα + JR64a/LV62<br>↓viability after JR64a 25 μM	LPS (10 μg/ml)/TNFα (50 ng/ml) for 24 h. JR64a orthosteric-allosteric ligands of the cannabinoid receptor type 2 (CB2R), the CB2R antagonist (SR144528, 1 μM), the CB2R positive allosteric modulator (EC21a 1 or 10 μM) 15 min before agonist administration
[123] Fu et al., 2022	Amyotrophic lateral sclerosis (ALS)	sh-C1QBP, si-C1QBP, PR50, MCC950, Syringin	N/A	N/A	↑APR50 aggregate in nucleus, ASC, caspase 1, pro-IL-1β, IL-1β, pro-IL-18, IL-18 NF-κB and NLRP3 after PR50<br>No changes in NLRP3 and ASC after MCC950+ PR50<br>↓Caspase 1, IL-18/IL-18, IL-1β after MCC950+ PR50<br>↑C1QBP after PR50<br>↓C1QBP after PR50+si-C1QBP<br>↑NF-κB, NLRP3, ASC, caspase 1, IL-1β, IL-18 after sh-C1QBP<br>No effect on NF-κB, NLRP3, ASC, caspase 1, IL-1β, IL-18 after sh-C1QBP +PR50<br>↓NF-κB, NLRP3, ASC, caspase 1, IL-1β, IL-18after PR50+SRG	proline-Arginine (PR)-Dipeptide Repeat Protein (PR50). MCC950 (an NLRP3 inhibitor), 100nM. NSC-34 motor neuron cells treated with a CM of PR50-expressing HMC3. SRG 2 μM for 24 h
[124] Durur et al., 2022	Alzheimer's disease	small neuron-derived EVs (sNDEVs)	N/A	N/A	↑sNDEVs after PKH67-labeled EV<br>↑IL-6 after sNDEV	dysregulated miRNA, let-7e in sNDEVs isolated from AD patients
[125] Chen et al., 2022	Neuroinflammation	H2O2, Gancao nourishing yin (GCNY) medium	N/A	N/A	↑ROS, pNF-κB p65 (ser536), IL-6, HO1, ABCC2, GLCM, ME1, NQO1, and TKT after H2O2<br>↓ROS, pNF-κB p65 (ser536), IL-6, HO1, ABCC2, GLCM, ME1, NQO1, and TKT after H2O2+GCNY	*In vivo*: GCNY treated rats to acquire medicated serum. H2O2 200 μM

(Continued)

10.3389/fimmu.2026.1778798

SUPPLEMENTARY TABLE 1 Continued

ARTICLE	MODEL	TREATMENT	DRUG TESTED	PATHWAY INVOLVED	MAIN RESULTS	NOTES
					Dysregulation of 58 expressed genes (DEGs) after H2O2<br>↓CENPF, MKI67, PRR11, and TOP2A after H2O2<br>↑CENPF, MKI67, PRR11, and TOP2A after H2O2+GCNY	
**[126]** Chen et al., 2022	Cancer (nasopharyngeal carcinoma, NPC)	NPC-derived-EV	Y27632 (a specific ROCK inhibitor),	N/A	↑Phagocytosis, IL-6, IL-8, CXCL1 and TGF-β1 after EV<br>↑miRNA-miR196a-5p, which can bind to ROCK1 (Rho-associated kinase1)-3′ UTR to decrease<br>↓ROCK1 after miRNA-miR196a-5p or Y27632<br>↑Phagocytosis and secretion of CXCL1 and TGF-β after Y27632	N/A
**[127]** Jia et al., 2022	Neuroinflammation, Neurotoxicity	Hemin, si-IGF1, LY294002,	kurarinone (Kur)	IGF1/PI3K/Akt	↑CD32, iNOS, TNFα, IL-6, IL-1β but ↓Arg-1, CD206 after hemin or si-IGF1 +hemin+Kur or hemin+Kur+LY294002<br>↓CD32, iNOS, TNFα, IL-6, IL-1β but ↑Arg-1, CD206 after hemin + Kur<br>↓IGF1, p-IGF1R/IGF1R, p-P13K/P13K, p-Akt/Akt after hemin or si-IGF1<br>↑IGF1, p-IGF1R/IGF1R, p-P13K/P13K, p-Akt/Akt after hemin + Kur or si-IGF1 + hemin + Kur + LY294002	SH-SY5Y cells co-cultured with hemin-treated HMC3 cells. Kur (10, 20, 40, or 80 µM) 24 h, hemin (60 µM)
**[128]** Wei et al., 2022	Alzheimer's disease	Aβ, isorhamnetin (ISN)	N/A	JAK2/STAT3	↑IL-6, Iba1, CD68, CD11b after Aβ<br>↓IL-6, Iba1, CD11b, NF-κB after Aβ+ISN<br>↓Viability after Aβ<br>↑Viability after Aβ + ISN	AβO (200 nM) for 24 or 48 h and then treated with ISN 10 µM 48 hours.<br>Treatment of the SH-SY5Y cells with the Aβ-activated HMC3-CM.
**[129]** Wei et al., 2022	Inflammation (retinal angiogenic microenvironment)	erucamide	N/A	N/A	Several novel binding protein candidates were subsequently found to be essential for activation of microglia regulated by erucamide	N/A
**[130]** Tu et al., 2022	Neuroinflammation	TAK-242 (a specific TLR4 inhibitor), Nec-1 (a specific RIPK1 inhibitor).	chlorpyrifos (CPF)	TLR4/TRIF/ RIPK/MLKL	Developmental exposure to CPF enhanced the expression of ↑inflammatory cytokines, TSPO, RIPK/ MLKL, TLR4/TRIF after CPF<br>↓TLR4/TRIF, RIPK/MLKL, necroptosis and pro-inflammatory cytokines after CPF +TAK-242 or CPF+Nec-1	N/A

(Continued)

10.3389/fimmu.2026.1778798

10.3389/fimmu.2026.1778798

SUPPLEMENTARY TABLE 1 Continued

ARTICLE	MODEL	TREATMENT	DRUG TESTED	PATHWAY INVOLVED	MAIN RESULTS	NOTES
**[131]** Liang et al., 2022	Alzheimer's disease	Aβ42, VER155008 (HSP70 inhibitor)	HLXL (Chinese medicine which contains 56 bioactive natural products identified in 11 medicinal plants)	Phagocytosis, HSP70	↑Aβ42 phagocytosis after HLXL	*In vivo:* 5XFAD transgenic animals were treated with HLXL and Aβ42, and/or LPS.<br>HLXL (100 and 200 μg/ml) in combination with Aβ42 (2 μg/ml) for 5 h. HSP70 inhibitor (VER155008, 12.5 μM)
**[132]** An et al., 2022	Alzheimer's disease	Aβ42-1, si-SIRT1, oligomycin, FCCP (H+ ionophore), rotenone, antimycin A (electron-transport chain inhibitor)	N/A	SIRT1/NRF2	↑SA-β-gal, p21, PAI-1, TNFα, IL-1β, IL-6 after Aβ<br>↓ATP, OCR, MMP but ↑ROS after Aβ<br>↓SIRT1, NRF2 mRNA and protein, phagocytosis after Aβ<br>↑SIRT1, NRF2 mRNA and protein, phagocytosis after Aβ + si-SIRT1 OE	Aβ42-1 10 μM for 24/48/72 h.
**[133]** Chen et al., 2022	Alzheimer's disease	LL-37, IAA-94 (CLIC1 inhibitor)	N/A	N/A	↑CLIC1 activation, membrane translocation and integration after LL-37<br>↑IL-1β, IL-6, TNFα, ROS, Ca2+, cell death after LL-37<br>↓IL-1β, IL-6, TNFα, ROS, Ca2+, cell death after LL-37+IAA-94	
**[134]** Ma'arif et al., 2022	Neuroinflammation	*M. crenata* extract, IFNγ	N/A	N/A	↑MHC II after IFNγ<br>↓MHC II after IFNγ +M. crenata<br>79 compounds in the extract (19 phytoestrogen as agonist toward 3OLS)	96% ethanolic extract of the M. crenata leaves of 62.5, 125, and 250 μg/mL.
**[135]** Zhu et al., 2022	Diabetes-associated cerebral atherosclerosis	si-IL-10, HG medium,	N/A	N/A	↑CD163, CD206 after HG 4 h, but then ↓CD163, CD206<br>↑IL-10, TGFβ1, TGFBI, TGFβ2 after HG 4 h<br>↑iNOS after HG 16 h<br>↓TGIF1, TGIF2 after HG 4 h<br>↓IL-10 after AAVs + si-IL-10	normal glucose (2.8 mmol/l) or HG (16.7 mmol/l)
**[136]** He et al., 2022	Neurotoxicity	cadmium (Cd2+), celastrol	N/A	N/A	↓Viability, proliferation, adhesion after Cd2 + 40 μmol/L<br>↑NO, MDA, p-PI3K mRNA, p-PI3K, p-AKT protein, membrane damage, after Cd2+<br>↓NO, MDA after Cd2++celastrol (10-7 mol/L)<br>↓p-PI3K, p-AKT, apoptosis after Cd2+ +celastrol	Celastrol (10–7 mol/L, 10–6 mol/L),

(Continued)

ARTICLE	MODEL	TREATMENT	DRUG TESTED	PATHWAY INVOLVED	MAIN RESULTS	NOTES
**[137]** Martin et al., 2022	Neuroinflammation	Tetrahydrocannabinol (THC)	N/A	N/A	↑Activated/resting cells after THC 10nM<br>↓Ameboid morphology, phagocytosis, activation after THC 10nM<br>No effect on Tat after THC	THC (1, 10, 100, or 1000 nM)
**[138]** Spampinato et al., 2022	Neuroinflammation	BAF-312, CM, TI (TNFα + IFNγ), UC-42-WP04 (Selective S1P5 agonist), NIBR-0321 (S1P1 antagonist), GM-6001 (Non-selective MMP inhibitor), MMP2 inhibitor III, MMP9 inhibitor I.	N/A	CCL5/CCR5	↓Migration after TI or NIBR alone or TI+NIBR or UC-42-WP04 or GM+BAF or MMP9 inhibitor or MMP2 inhibitor or MMP2 inhibitor+TI+BAF or CM_BBB_TI or CM_BBB_BAF + TI<br>↑Migration after TI+S1P1/5<br>No effect on migration after S1P1/5 alone<br>↓Mobility after CM_BBB_TI and CM_BBB_BAF + TI<br>TI significantly reduced<br>↓CCR5, claudin-5 but ↑CCL5, FITC-dextran after TI<br>↑CCR5, CCL5, claudin-5 after TI+ BAF<br>↓FITC-dextran after TI+BAF or MG-pre-TI+BAF<br>↑Claudin-5 after MG-pre-TI+BAF	TI=inflammatory cytokines (TNFα, 10 UI and IFNγ, 5 UI). CM from astrocyte/endothelial co-cultures after TI (BBB_CM_ TI) or TI + BAF (100 nM, BBB_CM_BAF + TI) for 6 h.<br>HMC3 incubated with CM and TI or BAF + TI 24 h further. S1P1/5 agonist BAF. NIBR-0321 1 µM)., UC-42-WP04, 1 µM). GM-6001, 5 µM, added 20 min before BAF exposure). MMP2 inhibitor III and MMP9 inhibitor I, both at 200 nM.
**[139]** Lacavalla et al., 2022	Neuroinflammation	Oxygen-ozone (O2-O3), LPS, DMF,	N/A	N/A	↑Larger roundish shape, migration, HO-1, IL-6, TNFα, cytoplasmic accumulation of lipid droplets after LPS<br>↓Electron density of the cytoplasm and mitochondria, Nrf2 and increased migration<br>No effect on viability, proliferation and morphology after O3 or O3+LPS or O3_LPS + DMF<br>↓motility and IL-6 after O3 or O3+LPS or O3_LPS + DMF	
**[140]** Gumbs et al., 2022	Viral infection (HIV)	HIVbal	N/A	N/A	CD68, CD11b, CD45, IBA1, CX3CR1 markers of HMC3<br>HMC3 not infected with HIVbal	10 ng (p24 Gag) HIVbal with a luciferase tag and cultured for the indicated day
**[141]** Yamazaki et al., 2022	Neuroinflammation	LPS	dexmedetomidine (DEX)	N/A	↑IL-6, IL-8, p38 MAPK, IκB and the translocation of NF-κB after LPS<br>↓IL-6, IL-8, p38 MAPK, IκB and the translocation of NF-κB after LPS + DEX or DEX alone	1000 nM DEX for 30 min and further incubated with or without 100 ng/mL LPS for 15 min.

(Continued)

10.3389/fimmu.2026.1778798

SUPPLEMENTARY TABLE 1 Continued

ARTICLE	MODEL	TREATMENT	DRUG TESTED	PATHWAY INVOLVED	MAIN RESULTS	NOTES
**[142]**<br>Liu et al., 2022	Cancer<br>(Uveal melanoma)	oHSV-1, poly(I:C)	N/A	N/A	↑Viability after poly(I:C) or poly(I:C) +oHSV-1 or CM-MUM2B+poly(I:C) +oHSV-1	CM from 92.1 and MUM2B cells treated with poly(I:C), oHSV-1
**[143]**<br>Yoo et al., 2022	Neuroinflammation	LPS, DMSO, siIRF1	JQ1	N/A	No effect on morphology or viability after DMSO or JQ1<br>↑larger cell bodies, fewer branches, and an amoeboid phenotype after LPS or LPS + JQ1<br>↑Iba1, P2RY12 and CSF1R after DMSO or LPS or JQ1<br>↑CCL2, CXCL10, IFNB1, IL1B, and TNFa after LPS<br>50 genes upregulated (CCL2, PCDH1, TNF, CSF3, and NKD1) after LPS + JQ1<br>273 genes were upregulated, and 249 genes were downregulated after JQ1<br>↑CSF2, IDO1, MMP3, MMP13, TNFSF10, VCAM1, IRF1, IRF2 after LPS<br>↓CSF2, IDO1, MMP3, MMP13, TNFSF10, VCAM1, IRF1, IRF2 after LPS + JQ1<br>↓IL1B, CCL2, IRF1, MMP13, VCAM1, CXCL10 after siIRF1	The bromodomain and extra-terminal (BET) inhibitor JQ1 (50, 500, and 5000 nM) for 4 h and LPS (100 ng/ml)
**[144]**<br>Zhukovsky et al., 2022	Neuroinflammation	IFNγ, hAAT, rhAAT	N/A	N/A	↑MHCII after IFNγ 10ng/ml or >IFNγ+ATT<br>↑IL-1β, HLA-DOB and HLA-DRAafter IFNγ+<br>↓IL-1β, HLA-DOB and HLA-DRA after ATT	IFNγ (10 and 100 ng/mL)
**[145]**<br>Mitra et al., 2022	Alzheimer's disease	Encapsulated cell biodelivery (ECB) and human mature nerve growth factor (hmNGF), IL1β/IFNγ/Aβ40/Aβ42	N/A	N/A	↑Activation after Aβ<br>stable hmNGF release from the ECB-NGF	ECB devices releasing hmNGF, termed ECB-NGF
**[146]**<br>Carota et al., 2022	Neuroinflammation, Neurotoxicity	α-lipoic acid (ALA), FAC	N/A	N/A	↓Proliferation, viability after FAC 8 h<br>↑Proliferation, viability after FAC+ALA<br>↑HO-1, FPN1, DMT1, IL-6, iNOS, mitochondrial mass, ROS, GSH after FAC<br>↓HO-1, FPN1, DMT1, IL-6, iNOS mitochondrial mass, ROS, GSH after FAC+ALA<br>↑ARG1 after ALA	FAC-induced iron overload 400 µM. ALA 100 µM 24 h

(Continued)

10.3389/fimmu.2026.1778798

SUPPLEMENTARY TABLE 1 Continued

ARTICLE	MODEL	TREATMENT	DRUG TESTED	PATHWAY INVOLVED	MAIN RESULTS	NOTES
**[147]** Auzmendi-Iriarte et al., 2022	Neurotoxicyty (aging)	Bleomycin, passages	N/A	N/A	↑histone deacetylases (HDACs) expression and senescence after bleomycin/serial passage	HDACs was assesed in mice and human hippocampal biopsies
**[148]** He et al., 2022	Neuroinflammation (Cerebral ischemia)	NR_002323.2, NR_145459.1, NR_144567.1,	N/A	TNF	↑CD68, M1 activation after NR_002323.2 overexpression	Lentivirus was used to upregulate the H_NR_002323.2
**[149]** Nguyen et al., 2022	Neuroinflammation	LPS	Efonidipine	JNK/NF-κB	↓IL-1β, migration after LPS + efonidipine	LPS 0.1 μg/mL, Efonidipine 1 μM
**[150]** Solan et al., 2022	Cytotoxicity	Short- and long-chain perfluoroalkyl and polyfluoroalkyl substances (PFAS)	N/A	N/A	↓Viability after PFAS<br>EC50 ranging from 1.34 to 2.73 μM for short- and long-chain PFAS	PFAS 10^-11 M to 10^-4 M tested on 6 cell lines
**[151]** Wang et al., 2022	Alzheimer's disease	N/A	N/A	N/A	IQCK is not expressed in HMC3	
**[152]** Liu et al., 2022	Alzheimer's disease	Aβ1-42	glycitin	PI3K/AKT	↑Autophagy, ATG5, ATG7, ATG12, beclin 1, LC3 puncta and LC3-I to LC3-II conversion after glycitin<br>↓p62, p-mTOR, p-AKT and Aβ after glycitin dose-dependent<br>↑Toxicity and cell death after Aβ	Glycitin (1, 5 and 10 μm) for 12 h as phytochemical constituent of soybean. Aβ1-42 24 h +glycitin (30, 60, 120 μm)
**[153]** Zhao et al., 2022	Angiogenesis (retinopathy)	siLGALS3BP, hypoxia, LY294002 (inhibitor of phosphoinositide 3-kinases)	N/A	PI3K/AKT	↑LGALS3BP, HIF-1α, MMP-9, MMP-2, and VEGF-A after hypoxia<br>↑migration, proliferation, and tube formation of HUEVECs and levels of angiogenesis-related factor in co-culture<br>↓migration, proliferation, and tube formation of HUEVECs and levels of angiogenesis-related factor in co-culture +siLGALS3BP<br>↑proangiogenic ability and angiogenesis-related factor expression after hypoxia<br>↓HIF-1α, MMP-9, MMP-2, and VEGF-A proangiogenic ability and angiogenesis-related factor expression after hypoxia+siLGALS3BP<br>↓Phosphorylation of PI3K, AKT, HIF-1α, VEGF-A, and MMP-2 after hypoxia +siLGALS3BP or hypoxia + LY294002	HUVECs co-cultured with hypoxic HMC3

(Continued)

10.3389/fimmu.2026.1778798

SUPPLEMENTARY TABLE 1 Continued

ARTICLE	MODEL	TREATMENT	DRUG TESTED	PATHWAY INVOLVED	MAIN RESULTS	NOTES
**[154]** Garliyev et al., 2022	Alzheimer's disease	cholesterol modulators	N/A	N/A	No effect on ABCA7 expression after intracellular cholesterol level or regulators of intracellular cholesterol metabolism.	
**[155]** Lin et al., 2022	Viral infection (ZIKV)	E143K SRIP (Single Round Infectious Particles (SRIPs) of ZIKV Asian-Lineage Natal RGN Strain with a Single Reverse Mutation at Residues 139 and 143 within the prM protein.)	N/A	N/A	↑ E143K at an MOI=0.5, localized near the cell plasma membrane<br>E143K > ZIKV WT<br>ZIKV WT in cytoplasm	N/A
**[156]** Urdánoz-Casado et al., 2022	Alzheimer's disease	circTREM2s	N/A	N/A	circTREM2_1 and circTREM2_3 detected in HMC3	N/A
**[157]** Zhang et al., 2022	Inflammation (hepatic encephalopathy)	AL137857.1, shAL137857.1-KO<br>siAL137857.1- OE<br>LPS	N/A	N/A	↑Cell activation, phagocytosis, cytokine biosynthetic process, IL-6 production and TNF after AL137857.1<br>↑AL137857.1 after LPS<br>↓IL-1, IL-6, TNFα, Cox2 and iNOS after AL137857.1-KO<br>↑IL-1, IL-6, TNFα, Cox2 and iNOS after AL137857.1-OE	N/A
**[158]** Adrianna et al., 2022	Neurotoxicity (Nanoparticle)	F12511 (selective inhibitor of Acyl-CoA), Nanoparticle F	N/A	N/A	↓ACAT after F12511 or F12511+nanoF or F12511-washout<br>No effect on morphology after F12511/nanoF	Washout performed after 0, 2, 4, 8 h.
**[159]** Zhang et al., 2022	Cancer (glioma)	Si-PTX3, co-culture	N/A	N/A	↑CD68 after si-PTX3<br>↓CD163 after si-PTX3<br>↓migration after si-PTX3 in co-culture	HMC3 was cocultured with si-PTX transfected U251 and U87 72 h
**[160]** Blusch et al., 2022	Huntington´ s disease	LPS, INFγ, AZD1480 (STAT3 inhibitor), Q25, Q97 (Q25 and Q97 for mutant huntingtin mHTT expresssion.)	N/A	JAK/STAT	↑HLA-DR, immunostaining, IL-6 and SOCS-3 RNA after LPS + INFγ<br>↓HLA-DR, STAT1 after LPS + INFγ+AZD1480<br>↑mHTT, cell death and ↓cell volume after Q25/Q97<br>↓mHTT, cell death and ↑cell volume after Q25/Q97+AZD1480	Q25 and Q97 for mutant huntingtin mHTT expresssion.
**[161]** Ceccarelli et al., 2022	Neuroinflammation, Cancer (GBM)	Large EVs (LEVs), Small EVs (SEVs), Extracellular Vesicles (EVs)	N/A	N/A	HMC3 EVs were not able to interfere with the proliferation of U87 GBM<br>↑α-Actinin-4, GAPDH after SEVs<br>↑α-Actinin-4, CD81, CD63, GAPDH after LEVs<br>LEVs and SEVs are heterogeneous (mean diameter ± SEM was 50.77 ±	LEVs:16K or SEVs: 100K

(Continued)

10.3389/fimmu.2026.1778798

SUPPLEMENTARY TABLE 1 Continued

ARTICLE	MODEL	TREATMENT	DRUG TESTED	PATHWAY INVOLVED	MAIN RESULTS	NOTES
					20.76 nm)<br>↑miR-146a-5p, IL-4, M2 phenotype after EVs<br>↓miR-155-5p, TNFα and IL-6 after EVs	
**[162]** Baghbanbashi et al., 2022	Neuroinflammation	LPS	fisetin ± polymersomes	ERK1/2	↑Viability after fisetin nanocarriers >fisetin alone<br>Lack of toxicity of fisetin and nanocarriers at 25×10−6 M.<br>↓ROS, p-ERK1/2 after LPS + fisetin or LPS + fisetin nanocarriers<br>No morphological changes after fisetin nanocarriers	Fisetin, both incorporated and nonincorporated into polymersomes,<br>(50×10−6<br>to 100×10−6 M) 1/24 h
**[163]** Arik et al., 2022	Neuroinflammation	OGD/R, MCC950	rhEPO	N/A	↓metabolic activity and viability after OGD (>3h)<br>↑HIF-1α, CAIX, IL-1β and LDH after OGD 4 h<br>O2<1% in medium after OGD (>4 h)<br>↑viability after OGD+R 12 h or OGD +rhEPO (1, 10, 200 IU/ml)<br>↑ROS after OGD or OGD+R 3h<br>↓ROS after OGD+rhEPO (1, 10, and 100 IU/ml)<br>no effect on LDH release, viability, metabolism and ROS after rhEPO +normoxic conditions<br>↑metabolic activity after OGD+rhEPO<br>↓LDH after OGD+rhEPO (1, 10, 200 IU/ml)<br>↑migration after rhEPO or OGD +rhEPO<br>↑phagocytosis after OGD+R<br>↑CASPASE1, IL-1β, and GSDMD after OGD<br>↑NLRP3, NLRC4, NLRP1 and AIM2 after OGD<br>↓NLRP3 after OGD+R<br>↓NLRC4 after OGD+R 3h<br>↓NLRP1 and AIM2 after OGD+R 24 h<br>↑NLRP1, NLRC4, and AIM2 after OGD+R+MCC950<br>NLRP1, NLRC4, and AIM2 after OGD +R<br>No effect on NLRP3, CASPASE1, IL-1β after MCC950	Recombinant human erythropoietin (rhEPO) [0.1 – 200 IU/ml] or MCC950 [1 μM] administered 1 h before OGD/R at different time point 1-6 h. MCC950 to functionally inhibit the NLRP3 inflammasome.

(Continued)

10.3389/fimmu.2026.1778798

SUPPLEMENTARY TABLE 1 Continued

ARTICLE	MODEL	TREATMENT	DRUG TESTED	PATHWAY INVOLVED	MAIN RESULTS	NOTES
**[164]** Ren et al., 2022	Hypoxia/ reoxygenation injury (H/R)	Circ-Memo1	N/A	miRNA-17-5p/ SOS1	↑TNFα, IL-1β, and IL-6 after H/R in co-culture<br>↓TNFα, IL-1β, and IL-6 after H/R +circ-Memo1 in co-culture<br>↑p-ERK and p-p65 but ↓IκB-α after H/R in co-culture<br>↓p-ERK and p-p65 but ↑IκB-α after H/R+circ-Memo1 in co-culture	HBMVEC/HMC3 co-culture
**[165]** Shaw et al., 2022	Alzheimer's disease	FL-TREM2, D2-TREM2	N/A	N/A	↑FL-TREM2, D2-TREM2 in Golgi complex after transfection	N/A
**[166]** Merlo et al., 2022	Alzheimer's disease	Aβ42	melatonin	N/A	↓SIRT1 and BDNF<br>↑anti-inflammatory activation, SIRT1 and BDNF expression/release after Aβ42+MEL<br>↑Caspase-1 and p-ERK after Aβ42<br>↓Caspase-1 and p-ERK after Aβ42 +MEL<br>were prevented by MEL. In addition, MEL partially restored<br>↓proteasome functionality, 20S and 26S chymotrypsin-like activity after Aβ42<br>↑proteasome functionality, 20S and 26S chymotrypsin-like activity after Aβ42 +MEL	Aβ42 (200 nM) in the presence of melatonin (MEL; 1 µM) added since the beginning (MELco) or after a 72 h-exposure to Aβ42 (MELpost).
**[167]** Zhao et al., 2022	Alzheimer's disease	LILRB2, oAβ, PS, Ab29	N/A	N/A	↑oAβ-lipoprotein complex phagocytosis, p-SYK, SYK, TREM2 pathway after LILRB2+Ab29<br>↓pSHP1,SHP1 and LILRB2 pathway after LILRB2+Ab29	LILRB2, an inhibitory receptor bearing ITIM motifs.
**[168]** Kubelt et al., 2022	Neuroinflammation	Deep brain stimulation (DBS)	N/A	N/A	↑proliferation after DBS 24 h<br>No effect on viability after DBS 24 h<br>No expression of CXCL12, CCL2, or CCL20 after DBS<br>↑IL-1β, IL-6, CXCL16 after DBS	Rlectrically stimulated (2 mV)
**[169]** Lin et al., 2022	Alzheimer's disease	Aβ40, BIRB 796 or SB202190, UV exposure	N/A	N/A	↑IL-6 after Aβ40 dose-dependent<br>↑IL-6 and TNFα mRNA, p38 MAPK, CD68, mROS after Aβ40<br>No effect on NO, ErK, JNK, MAPK after Aβ40<br>↑p-JNK after UV<br>↓IL-6 after Aβ40+BIRB 796 or Aβ40 +SB202190	2 µM Aβ40 or Scr-Aβ40 4 h, incubation with BIRB 30 min before.

(Continued)

10.3389/fimmu.2026.1778798

SUPPLEMENTARY TABLE 1 Continued

ARTICLE	MODEL	TREATMENT	DRUG TESTED	PATHWAY INVOLVED	MAIN RESULTS	NOTES
**[170]** Gao et al., 2022	Viral infection SFTS	JS-2010, JS-2014, GSK872, Nec-1s, VX765, Z-VAD, Z-DEVD, shNLRP1, shNLRP3, shAIM2, shNLRC4, MCC950, UV-infection, heat-infection, CHX, NSs-OE, NSs-66	N/A	NLRP3	↓Viability after SFTSV 48 h<br>↑Viral S genomic segments, IL-1β, NP after SFTSV<br>↓CPE and cell viability after JS-2010 +VX765/Z-VAD/Z-DEVD<br>↓IL-1β after SFTSV+shNLRP3 or CHX<br>↓Viability after SFTSC+shNLRP3/ shASC/sh-caspase-1<br>↓caspase-1, GSDMD, viral S gene after SFTSV+MCC950/VX765 or UV/heat-SFTSV or CHX<br>↓viral S gene after SFTSV+shNLRP3/ shASC/sh-casapse-1<br>↓caspase-1, GSDMD after UV/heat-SFTSV<br>↺ caspase-1, GSDMD after SFTSC+sh-caspase-1/GSDMD<br>↑NLRP3 aggregate into speckle-like structures, after NSs-OE<br>↑IL-1β, caspase-1, GSDMD after NSs1-66	SFTSV two strains JS-2010 and JS-2014 in multiplicity of infection (MOI) of 0.04, 0.2, or 1 and measured for 24, 48 and 72 h. Incubation of JS-2010 with inhibitors of RIPK3 (GSK872), RIPK1 (Nec-1s), procaspase-1 (VX765), pan-caspases (Z-VAD), and procaspase-3 (Z-DEVD)
**[171]** Hen et al., 2022	Cancer	co-culture with U251	N/A	N/A	↓invasion of macrophges in co-culture HMC3 and U251 cells	N/A
**[172]** Garcia et al., 2022	Viral infection (HIV)	nanodiscs (empty), nanodisc- tenofovir (TFV)	N/A	N/A	↓Viability after nanodisc >0.05%<br>↓Viability after 1:20 ND-TFV >0.075 mg/ml<br>↑ROS after 1:20 ND-TFV but not 1:40 ND-TFV	Lipid concentrations of the nanodiscs (ND) 0.01–0.53%. nanodiscs at drug-to-lipid ratios of 1:20 and 1:4, and also with TFV free drug (0.01–0.1 mg/ml)
**[173]** Dedert et al., 2022	Diabetes	hyperglicemia	N/A	N/A	No changes in Progranulin in HG	Glucose at 100nM 72 h
**[174]** Cook et al., 2022	Aging (senescence)	BAY 11-7082 (NF-κB inhibitor), Ionizing radiation (IR)	N/A	p53/p21 NF-κB/p65	↑β-galactosidase, senescence, p53, p21, SASP, IL-6 after IR<br>↓IL-6, senescence after IR + BAY 11-7082	10 Gray (Gy) of ionizing radiation (IR) using a 137Cs γ-irradiator
**[175]** Hu et al., 2022	Viral infection Herpes simplex virus 1 (HSV1)	HSV-1, MCC950	N/A	NLRP3	↑IL-1β, LDH after HSV-1<br>↓GSDMD after HSV-1<br>↑GSDMD after HSV-1+MCC950<br>↓LDH after HSV-1+MCC950	MCC950 (5 μg/mL), HSV-1 (MOI = 5) for 24 h

(Continued)

10.3389/fimmu.2026.1778798

SUPPLEMENTARY TABLE 1 Continued

ARTICLE	MODEL	TREATMENT	DRUG TESTED	PATHWAY INVOLVED	MAIN RESULTS	NOTES
**[176]**<br>Sanjay et al., 2022	Alzheimer's disease	Aβ42, Cyanidin-3-O-glucoside (C3G), GW9662 (irreversible PPARγ antagonist)	N/A	PPARγ/TREM2	↓CD86, CD80, M1 phenotype, IL-Iβ, IL-6, TNFα, ROS after C3G<br>↑CD206, CD163, M2 phenotype, PPARγ, Aβ42 phagocytosis after C3G<br>↓PPARγ after C3G + GW9662	GW9662 (10 µM for 30 min), followed by co-treatment with C3G (25, 50, 100, 200 µM) co-treated with of $A\beta_{42}$ (1 µM) and for 24 h incubation
**[177]**<br>Li et al., 2022	Neurotoxicity (neurosyphilis)	Tp47 (recombinant *Treponema pallidum*)	N/A	PERK/ATF4, IRE1/XBP1	↑IBA-1, phagocytosis, MHC-II, P62, Beclin1, Lamp2, LC3-I/LC3-II, autophagosomes and lysosomes fusion after Tp47<br>↓phagocytosis after Tp47 + 3-AM/Baf A1<br>↑PERK, IRE1α, GRP78, ATF4 and XBP1s Tp47<br>↓IRE1α after Tp47+TUDCA<br>↓PERK, ATF4 after Tp47 + 4-PBA	Tp47 MOI of 10:1, 50:1, 100:1, or 150:1 for 24 h
**[178]**<br>Zhang et al., 2022	Cancer (GBM)	Co-culture with U251/U87, irradiation, GW4869 (inhibitor of exosome generation), CCR2-KO, circ_0012381, miR-340-5p inhibitor, miR-340-5p mimics	N/A	CCL2/CCR2	↓Phagocytosis after IR in co-culture<br>↑Phagocytosis after IR+GW4869 in co-culture<br>↓M2 phenotype after IR<br>↑M2 phenotype after IR+CCR2-KO<br>↑circ_0012381 after IR<br>↑TGFβ, CCL2, IL-1, IL-10, CD163, M2 phenotype after circ_0012381<br>↓TNFα after circ_0012381<br>↑M2 phenotype, ARG1 after miR-340-5p<br>↓Phagocytosis after miR-340-5p<br>↓ARG1 after miR-340-5p inhibitor	Irradiation (2Gy), collection of exosomes
**[179]**<br>Zhang et al., 2022	Alzheimer's disease	fSNPs, fSNPs-KO, shGATA2, shGATA3, shELAVL1, shHNRNPA0, shNFIB, shNFIC, shCUX1, shILF2, shILF3, shILF2, shILF3	N/A	HLA-DQA1/ DRB1	Different patterns of the allele-imbalanced gel shifting in NE<br>↓GATA2, GATA3, ELAVL1, HNRNPA0, ILF2, ILF3, NFIC, NFIB, CUX1 in NE after fSNPs-KO<br>↓HLA-DRB1, HLA-DQA1 after shGATA2/shGATA3/shELAVL1/ shHNRNPA0/shNFIB/shNFIC/shCUX1<br>↑HLA-DRB1, HLA-DQA1 after shILF2/ shILF3<br>↓HLA-DMA, HLA-DQB1 and lack of HLA-DQA2, HLA-DQB2, HLA-DRB5, HLA-DRB6 after shGATA2/shGATA3/ shCUX1	6 candidate fSNPs, rs9271198, rs9271200, rs9271213, rs9271243, rs9271247, and rs9281945 isolated from nuclear extract (NE)

(Continued)

10.3389/fimmu.2026.1778798

10.3389/fimmu.2026.1778798

SUPPLEMENTARY TABLE 1 Continued

ARTICLE	MODEL	TREATMENT	DRUG TESTED	PATHWAY INVOLVED	MAIN RESULTS	NOTES
[180]<br>Zhang et al., 2022	Cancer (GBM)	co-cultured with primary pericyte cells (1:1), MCAM-siRNA-1/2/3	N/A	CD163/MCAM	↓MCAM, migration, M2 phenotype after siRNA-1/siRNA-2<br>↑M1 phenotype after siRNA-1/siRNA-2	N/A
[181]<br>Sreenivasan et al., 2022	Cancer	Co-culture with Med8A-S or D283 or D341	Vincristine, ruxolitinib, Bazedoxifene	gp130/STAT3	No effect on Med8A-S(HMC3) or Med8A-S(HMC3)+gp130-/- after vincristine<br>↑chemoresistance, pJAK1, pY705-STAT3 in Med8A-S(HMC3)+IL-6R-/- after vincristine<br>↓pY705-STAT3 in Med8A-S(HMC3) +IL-6R−/− after ruxolitinib<br>↑IL-6, IL-11, LIF in Med8A-S(HMC3)<br>↓chemoresistance, pJAK1, pY705-STAT3 in Med8A-S(HMC3)+IL-6R-/- after vincristine+ruxolitinib<br>No effect on Med8A-S(HMC3) +gp130-/- after v<br>↓chemoresisance in Med8A-S(HMC3) after vincristine+Bazedoxifene<br>↑chemoresistance, pJAK1, STAT3, in co culture D283/D341after vincristine	N/A
[182]<br>Cai et al., 2022	Parkinson’s disease	LPS, BMSC-exosomes, siGli1, shGli1, shSp1,	N/A	N/A	↑TNFα, IL-1β, IL-6, Sp1, LRRK2, COX2, after LPS or LPS + BMSC-sxosomes+shGli1<br>↓TNFα, IL-1β, IL-6, Sp1, LRRK2, COX2, after LPS + BMSC-exosomes or LPS + shSp1<br>↑Gli1 after BMSC-exosomes<br>↓Sp1 after BMSC-exosomes+siGli1<br>↑Sp1 after BMSC-exosomes+shGli1	Exosomes from bone marrow-derived mesenchymal stem cells (BMSC) 20, 40, and 80 µg/mL for 24 h.
[183]<br>Sharifi et al., 2022	Neuroinflammation	HSA methacrylate (HSAMA), resulting hydrogels	N/A	N/A	Lack of toxicity after HSAMA or hydrogel<br>normal morphology on glass slides<br>only a few spots, <40% confluence, smaller perimeter, filopodia on the hydrogel slides<br>↓IL-6 >M-CSF> MCP-1> IL-12p40> FGF-2> IL-8> IL-27> IL-1β>RANTES> EGF on hydrogel slides<br>↓IL-6> IL-8> VEGF-A> MCP-1> PDGF-AA> GROα> IL-27> RANTES> IL-22> FGF-2 on glass slides	gelMA of 10 mg/mL, HSAMA 5, 10, and 20 mg/mL for 24 h

(Continued)

SUPPLEMENTARY TABLE 1 Continued

ARTICLE	MODEL	TREATMENT	DRUG TESTED	PATHWAY INVOLVED	MAIN RESULTS	NOTES
**[184]** Jeong et al., 2022	Viral infection (SARS-CoV-2)	SARS-CoV-2, CR3022 (SARS-CoV-2 Spike Protein), Z-DEVD-FMK (caspase-3 inhibitor), Z-IETD-FMK (caspase-8 inhibitor), and Z-VAD-FMK, Ac-FLTD-CMK (caspase-1 inhibitor) and Belnacasan (VX-765, caspase-1 inhibitor)	N/A	N/A	↑M1 phenotype, CD68, CX3CL1, CX3CR1, IL-1β, IL-6, TNFα after SARS-CoV-2 infection<br>No effect on IL-10, NLRP3, GSDMD, caspase-1 after SARS-CoV-2 infection<br>↓relative infection % after CR30022 2 dpi<br>↓viability after SARS-CoV-2 4 dpi<br>↑ER stress (DNAJB7, DDIT3, HSPA5, HSP90B1, HYOU1, PDIA4, SEL1L) and IFN after S3 and S6<br>↑CD16, phospho-Stat1, and Stat1 after SARS-CoV-2 4 and 6 dpi<br>↓AURKA, BIRC5 after SARS-CoV-2<br>↑DDIT3, RHOB, FAS, PIDD1, Fas, DR4, DR5, TNFR2 after SARS-CoV-2<br>↑viability after SARS-CoV-2+Z-DEVD-FMK/Z-IETD-FMK/Z-VAD-FMK<br>No effect on viability after Ac-FLTD-CMK/Belnacasan	5 μg/mL CR3022
**[185]** Gottschalk et al., 2022	Neuroinflamation (Myalgic Encephalomyelitis)	Chronic Fatigue Syndrome (ME/CFS) patients-derived serum, Autophagy-Related protein 13 (ATG13),	N/A	N/A	↑ROS, NO, RAGE after ATG13<br>↓ROS, NO after ATG13-KO	
**[186]** Nicola et al., 2022	Alzheimer's disease	L-lactate, 3CL-HBA	N/A	HCAR1	↓Phagocytosis (25%) after L-lactate 6 h or 3CL-HBA	HCAR1 agonist 3-chloro-5-hydroxybenzoic acid 3CL-HBA 0.16 mM and 0.5 mM
**[187]** Chi et al., 2022	ALS	MATR3-KO, MATR3-KD, siALS, aiCIITA, IFNγ	N/A	ALS	↓HMC II after MATR3-KO/KD or CIITA-KD<br>↑HLA-DR after IFNγ	
**[188]** Gao et al., 2022	Neuinflammation (Ischemic stroke)	particulate matter (PM2.5), OGD/R, shNLRP3, ROS inhibitor N-acetyl-L-cysteine (NAC)	N/A	N/A	↓viability after OGD/R+PM2.5 dose-dependent<br>↑NLRP3, pyroptosis, ASC, pro-caspase-1, Caspase-1, GSDMD, GSDMD-N, IL-1β, IL-18, SYTOX, ROS after OGD/R +PM2.5<br>↑viability, NLRP3 inflammasome activation, pyroptosis after PM2.5 +shNLRP3<br>↓ROS after OGD/R+PM2.5+NAC<br>↓NLRP3 inflammasome activation, pyroptosis after OGD/R+PM2.5+NAC	N/A

(Continued)

10.3389/fimmu.2026.1778798

SUPPLEMENTARY TABLE 1 Continued

ARTICLE	MODEL	TREATMENT	DRUG TESTED	PATHWAY INVOLVED	MAIN RESULTS	NOTES
**[189]** Oron et al., 2022	Alzheimer's disease	dCas9-VP64, dCas9-KRAB, sgRNA-B10, sgRNA-B13	doxycycline (Dox)	N/A	↑APOE after dCas9-VP64+B10/B13 +dox 2 days<br>↑APOE after dCas9-VP64+B10+dox 6 days<br>No effect on APOE after dCas9-KRAB<br>No effect on TOMM40 after dCas9-KRAB/dCas9-VP64	dCas9-VP64 (Activation), dCas9-KRAB (Interference) or dCas9 (control) using lentiviral vectors, short-guide RNAs (sgRNAs) targeting either B10 or B13.
**[190]** Bailey et al., 2022	Neuroinflammation (Ischemic stroke)	Hypoxia, BQ123 (ET-1 receptor A (ETRA) antagonist), ET-1	N/A	N/A	↑ROS, TNFα, IL-6, ET-1 (mRNA 5-fold, protein 1.3-fold) under hypoxia<br>↑ROS, TNFα, IL-6 after ET-1<br>↓ROS, TNFα, IL-6, ET-1 after hypoxia +BQ123	hypoxia using a chamber (1% O2, 5% CO2, and 92% N2) at 37°C for 4 h.
**[191]** Wang et al., 2022	Inflammation (uveitis)	LPS, INFγ, PRDX3-shRNA	icarin (ICA)	GPX4/ SLC7A11/ ACSL4	↑M2 phenotype, ARG1, CD206, IL-10 after LPS + IFN-γ+ICA<br>↓TNFα, COX-2, iNOS, M1 phenotype after LPS + IFN-γ+ICA<br>No effect on viability after ICA<br>↑PRDX3 after ICA 10 μM or ICA+LPS + IFN-γ<br>↓H2O2 after ICA<br>↓M2 phenotype, ARG1, CD206, IL-10, H2O2 after LPS + IFN-γ+ICA +shPRDX3<br>↑TNFα, COX-2, iNOS, M1 phenotype after LPS + IFN-γ+ICA+shPRDX3<br>↑GPX4, SLC7A11, ACSL4 after ICA<br>↓GPX4, SLC7A11, ACSL4 after ICA +shPRDX3	ICA (0.1 μM, 1 μM, 10 μM)
**[192]** Alaqel et al., 2022	Neuroinflammation (ICH)	LPS, thrombin	SZ3, SK-1-32 (cofilin inhibitors synthesized from o-formylation of 4-(tert-butyl)phenol)	N/A	↑survival after LPS + SK-1-32<br>↓NO, neurotoxicity after LPS + SK-1-32 or LPS + SZ-3<br>↓cofilin, activation, migration, proliferation after LPS + SZ-3<br>↓NO, TNFα, PAR1 after thrombin+SZ-3<br>↑phosphocylin after thrombin+SZ-3	N/A
**[193]** Cui et al., 2022	Alzheimer's disease	TMEM16F siRNA, Aβ25–35, nigericin (agonist of NLRP3)	N/A	N/A	↑TMEM16F, iNOS, Cox2 after Aβ25–35<br>↓TMEM16F, iNOS, Cox2 after Aβ25–35 + siTMEM16F<br>↑Arg1, Socs3, IL-4, IL-10, TGF-β after Aβ25–35 + siTMEM16F<br>↓IL-1, IL-6, TNFα, NLRP3	20 μM Aβ25–35 for 24 h. 5 μM nigericin for 1 h.

(Continued)

10.3389/fimmu.2026.1778798

SUPPLEMENTARY TABLE 1 Continued

ARTICLE	MODEL	TREATMENT	DRUG TESTED	PATHWAY INVOLVED	MAIN RESULTS	NOTES
					inflammasome, NLRP3, ASC, IL-18, IL-1β, pro-caspase1 after Aβ25–35 + siTMEM16F<br>↑IL-18, IL-1β, iNOS, Cox2 but ↓Arg1 after Aβ25–35 + siTMEM16F + nigericin	
**[194]** Huang et al., 2022	Neuropathic pain	miR-185-5p mimics, miR-185-5p inhibitor, shMyD88, shCXCR4	N/A	N/A	↓MyD88 and CXCR4 after miR-185-5p mimics<br>↑MyD88 and CXCR4 after miR-185-5p inhibitor<br>↑MyD88-AgO2, CXCR4-AgO2 after miR-185-5p mimics	N/A
**[195]** Tréguier et al., 2022	Viral infection (ZIKV)	ApoE and Zika envelope glycoprotein (Zika E)	N/A	N/A	↑co-immunoprecipitation of ApoE and Zika E protein after ZIKV 48 h<br>↑membrane rearrangements (convoluted membranes, vesicle packets) after ZIKV<br>↑ApoE, Zika E protein secretion after ZIKV	N/A
**[196]** Hernando et al., 2022	Neuroinflammation	LPS, Miglyol-NLC, DHAH-NLC, DHAH-NLC-GDNF, DHAH-NLC-VEGF	N/A	Nrf2/HO-1	↓viability after NLCs 48 h<br>no effect on COX-2 and NF-κβ after LPS<br>↑ameboid morphology after NLCs<br>No effect on IL-6, TNFα, IL1-β, NF-κB, COX-2, Nrf2, HO-1 after NLCs<br>↓IL-6, TNFα after LPS + DHAH-NLC/ DHAH-NLC-GDNF/DHAH-NLC-VEGF (but not Miglyol-NLC)	NLCs: GDNF and VEGF (DAH-NLC-GNDNF) and (DHAH-NLC-VEGF) 25 μM for DHAH lipid content and 25 ng/ml
**[197]** Chen et al., 2022	Cancer (GBM)	N/A	paclitaxel (IONP@PTX)	N/A	↓Viability after PTX or IONP@PTX<br>PTX > IONP@PTX inhibitory effect	IONP@PTX (0, 0.4, 0.8, 1.6, 3.2, 6.4, 12.8, 25.6, and 51.2 μg/ml) 24 h HMC3 used only to assesed viability, the experiment was performed also on U251 and *in vivo*.
**[198]** Murdock et al., 2022	Cancer (Glioma)	Saline-soluble fraction of urinary bladder ECM (ECM-SF)	N/A	N/A	↓Viability after ECM-SF<br>No caspase activity after ECM-SF	ECM-SF 1, 5, or 10 mg/mL 24 h
**[199]** Ding et al., 2022	Neuroinflammation	CCL18, siCCL18, siPITPNM3	N/A	NF-κB/Src	No effect on IL-1β, IL-6, TNFα, iNOS after CCL18<br>↑macrophage markers (MRC-1, ARG-1 M2), phagocytosis after CCL18<br>CCR8 and PITPNM3 expressed in HMC3<br>↓CCR8 and phagocytosis after siCCR8	N/A

(Continued)

10.3389/fimmu.2026.1778798

SUPPLEMENTARY TABLE 1 Continued

ARTICLE	MODEL	TREATMENT	DRUG TESTED	PATHWAY INVOLVED	MAIN RESULTS	NOTES
					No effect on phagocytosis after siPITPNM3	
**[200]** Ramakrishnan et al., 2022	Alzheimer's Disease	TREM2 KO, AβO	K161 (pan SHIP-1/2 inhibitor, SHIPi)	TREM2/SHIP-1	↑ Phagocytic uptake of AβO in both $HMC3^{WT}$ and $HMC3^{T2KO}$ after treatment with K161	N/A
**[201]** Jiang et al., 2022	MS	LPS, sh-hsa_circ_0000518, IL-4, FUS, sh-FUS, STO-609 (inhibitor of the Ca 2+/calmodulin-dependent protein kinase kinase (CaM-KK))	N/A	FUS/CaMKKβ/ AMPK	↑hsa_circ_0000518, NO, iNOS, ROS, FUS apoptosis after LPS<br>↓hsa_circ_0000518, NO, iNOS, ROS, apoptosis after LPS + sh_hsa_circ_0000518<br>↑M1 markers (TNFα, IL-1β, CD16, CD32) but ↓M2 markers (IL-10, TGF-β, CD206, Arg1) after LPS or STO-609<br>↓M1 markers (TNFα, IL-1β, CD16, CD32) but ↑M2 markers (IL-10, TGF-β, CD206, Arg1) after LPS + sh_circ_0000518 or LPS + sh-FUS<br>↓CD16, CD32 but ↑CD206, Arg1 after IL-4 or IL-4+FUS or IL-4+ sh-hsa_circ_0000518<br>↓FUS, iNOS, NO, ROS, apoptosis after LPS + sh-FUS<br>↓CaMKKβ, AMPK, PGC-1α but ↑P-p65 after LPS<br>↑CaMKKβ, AMPK, PGC-1α but ↓P-p65 after LPS + sh-FUS<br>LPS + STO-609.	100 ng/mL of LPS for 24 h, then the short hairpin RNA against hsa_circ_0000518 (sh-hsa_circ_0000518) was transfected into cells and incubated for 48 h. STO-609, a CaMKKβ inhibitor,
**[202]** Jiang et al., 2022	Cancer	GSC406-CM, circKIF18A-KD, circKIF18A-OE, co-culture with hBMCEC	N/A	N/A	↑M2 markers (CD163, CD206, ARG1, IL-6 and TGF-β) but ↓M1 marker (IL1β and TNFα) after GSC406-CM<br>↑circKIF18A in M2-HMC3<br>No effect on KIF18A mRNA after KIF18A-OE/KIF18A-KD<br>↑FOXC2 stabilization and nuclear translocation after hMBECs+M2-HMC3	N/A
**[203]** Murcia et al., 2022	Alzheimer's disease	HT4253, HT4403 (RAB10 inhibitors), Amyloid Precursor Protein (APP) overexpression	N/A	N/A	↓Phosphorylation RAB10 after HT4253 (IC50 = 63nM), HT4403 (IC50 = 82nM)<br>↓LRRK2 after HT4253/HT4403<br>↑LAMP1, Aβ42-40 after HT4253/ HT4403	N/A

(Continued)

10.3389/fimmu.2026.1778798

SUPPLEMENTARY TABLE 1 Continued

ARTICLE	MODEL	TREATMENT	DRUG TESTED	PATHWAY INVOLVED	MAIN RESULTS	NOTES
**[204]** Brown et al., 2022	MS (associated Bacterial Ligand)	IFN-β, Fingolimod (FTY-720), lipid 654, lipoteichoic acid (LTA)	N/A	N/A	↑IL-12 after Fingolimod<br>↑MIP-1β, TNFα after IFN-β<br>↓IL-12, IL-6, GM-CSF, IFN-γ, MIP1β, MCP-1, IL-2, IL-8 after Lipid 654+IFN-β/Fingolimod<br>↓IL-7 after Lipid 654+Fingolimod<br>↓IL-10 after LTA+Fingolimod<br>↑iNOS, CD14, CD86, CD1631, CD206, Cx3CR1 after IFN-β/Fingolimod<br>↑iNOS, CD86 after Lipid 654+IFN-β/Fingolimod<br>↓CD14 after Lipid 654+IFN-β<br>↓CD163, Cx3CR1 after Lipid 654+IFN-β<br>↑CD206, Cx3CR1 after Lipid 654 +Fingolimod<br>↓CD163 after Lipid 654+IFN-β/Fingolimod<br>↓CD1631, CD206, Cx3CR1, iNOS after LTA+IFN-β<br>↓CD206, Cx3CR1, iNOS after LTA +Fingolimod<br>↑CD14, CD86 after after LTA +Fingolimod	10 ng/mL of recombinant IFN-β, 10 nM of Fingolimod, 0.69 μg/mL lipid 654 and 1 μg/mL LTA (TLR-2 ligand)
**[205]** Fernandes et al., 2022	AMD	Nanoemulsions (NEs), free-TA, FD4, co-culture ARPE-19/HMC3	N/A	N/A	↑Papp after NEs<br>↓Paap after TA/FD4	permeability recorded after 4 h
**[206]** Brown et al., 2022	Alzheimer's disease	rs6498140, motif-containing enhancers	N/A	N/A	↑enhancer activity of MEF2 and MEF2C's<br>↓enhancer activity after disruption the MEF2C transcription factor binding sites<br>↑enhancer activity for rs6498140 (proximal to CLEC16A gene) in AD-associated SNP<br>↑CLEC16A gene after rs6498140	WhAMPRA measures the tissue-specificity of candidate enhancers activity.
**[207]** Shukla et al., 2022	Viral infection (ZIKV NS1)	ZIKV-NS1, miR-146a-OE	N/A	N/A	↓ROS after ZIKV-NS1<br>↑miR-146a after ZIKV-NS1<br>↓TRAF6, STAT-1, pNF-κBp65, TNFα, proinflammatory and antiviral response after miR-146a-OE	N/A

(Continued)

10.3389/fimmu.2026.1778798

SUPPLEMENTARY TABLE 1 Continued

ARTICLE	MODEL	TREATMENT	DRUG TESTED	PATHWAY INVOLVED	MAIN RESULTS	NOTES
**[208]** Zeng et al., 2023	Cancer (Gliomas)	N/A	N/A	N/A	↓MYD88 expression in HMC-3 than U87	HMC3 used only for comparison
**[209]** Aoki et al., 2023	Neuroinflammation	pGL3-luciferase reporter vector for OLR-1 gene	N/A	p38-MAPK/NF-κB	Region 1576 and 1682 bp show changes in luciferase activity<br>↑NF-κB transcriptional activity than HIF-1α<br>OCT-1 is a transcriptional repressor with minimum activity on OLR-1 transcription	HMC3 used only for chromatin immunoprecipitation.
**[210]** Sawkulycz et al., 2023	Neuroinflammation (Ischemic stroke)	LPS, mCRP, nCRP, GW3965, MFAT-CM	N/A	N/A	mCRP can alter morphology and physiological function, acting as a proinflammatory mediator<br>↑IL-1b, IL-6, and TNFa after mCRP<br>↓IL-1b, IL-6, and TNFa after GW3965 +LPS/mCRP with no effect on gene expression<br>↓TNFa, IL-1b, IL-6, and IL-10, both protein and gene expression, after MFAT-CM+LPS/mCRP<br>↑IL-1ra, IL-1b, and IL-4 after MFAT	CRP and its isoforms mCRP and nCRP, The liver x receptor agonist (GW3965) or Micro-fragment adipose tissue (MFAT) conditioned media
**[211]** Jung et al., 2023	Gene expression HDAC1/ 2	romidepsin	N/A	Histone acetylation (H3Ac/H4Ac)	↑viability after 72 h, romidepsin (1.0 ng/ml)	0.5, 1.0, and 5.0 ng/ml romidepsin (24/48/72 h)
**[212]** Yoshida et al., 2023	Alzheimer's disease	*Porphyromonas gingivalis* wild-type Pg (Pg OMVs [WT]) or KDP136 (Pg OMVs [K136])	N/A	N/A	↑IL-6, TNFα, IL-8, and IL-1β after Pg OMVs (WT) in gingipain-dependent manner<br>No changes in IL-6, TNFα, IL-8, and IL-1β after Pg OMVs (K136)	500 ng/ml for 3 h outer membrane vesicles (OMVs) Pg, isolated from the culture medium of the KDP136 strain of Pg, which is Kgp/Rgp double-deficient
**[213]** Wagner et al., 2023	Neuroinflammation	LPS, TNF, poly(I:C)	*Amanita muscaria* extract (AME-1), trehalose	N/A	↑CD86, CXCR4, CD45, CD125, and TLR4 after AME-1<br>↑IL-6 and IL-8 after LPS, TNF, and poly(I:C)<br>↑IL-8 after AME-1 high concentration/ +poly(I:C) or trehalose+poly(I:C)<br>Altered TLR3 mRNA but not TLR3 protein, RIG-I gene, and MDA5 protein after AME-1<br>↑Autophagy after trehalose	Comparison to TNF, polyinosinic-polycytidylic acid [poly (I:C)], substance P and LPS.<br>Trehalose is a metabolite of AME-1
**[214]** Sanjay et al., 2023	Neuroinflammation, Oxidative stress	LPS	carbon quantum dots (CQDs)	nuclear factor erythroid 2-related factor 2,	↑Cell viability<br>↓ROS and GPx<br>↑Nrf2, HO-1, HO-2, SOD, and CAT	LPS (1 μg/mL) or LPS (1 μg/mL) + CQD (62,5 μg/mL) co-treatment

(Continued)

10.3389/fimmu.2026.1778798

SUPPLEMENTARY TABLE 1 Continued

ARTICLE	MODEL	TREATMENT	DRUG TESTED	PATHWAY INVOLVED	MAIN RESULTS	NOTES
				(Nrf2)/heme oxygenase-1 (HO-1)	mRNA expression<br>↓IL-1β, TNFα, and IL-6<br>↓DPPH and ABTS levels<br>↑IL-4, IL-10, and TGFβ	
**[215]** Marzouk et al., 2023	Viral infection (HIV)	recombinant HIV-1 Tat	cannabinoids, terpenes	trans activator of transcription (Tat)	↓Inflammation<br>↓Citotoxicity<br>↑Reactive/phagocytic cells	N/A
**[216]** Ahuja et al., 2023	Cancer (Breast)	Conditioned Medium (CM)	N/A	BDNF/TrkB, PDGFA	↑CCL5, CXCL8/IL-8, PTX3, CSF2, RBP4, and FLT3LG in SKBR3 + CM HMC3<br>↑PTX3 in the CM HMC3 + HBEC5i<br>↑PDGFA and BDNF in CM HMC3<br>Proliferation SKBR3 via activation of TrkB and HER2 receptors in CM HMC3	CM combined from serum-deprived astrocytes (NHA), microglia (HMC3) and brain endothelial (HBEC5i) cells<br>CM added to Human HER2+ breast cancer cells (SKBR3) and brain endothelial cells (HBEC5i).
**[217]** Polini et al., 2023	Alzheimer's disease	LPS + TNFα, $A\beta_{25-35}$, T1AM	3-iodothyronamine (T1AM)	T1AM/trace amine receptor 1 (TAAR1)	↑Anti-inflammatory mediators, such as IL-10<br>↓Proinflammatory factors, including IL-6, TNFα, NF-κB, MCP1, and MIP1	0.1, 1, and 10 μM of T1AM
**[218]** Polini et al., 2023	Neuroinflammation	LPS/TNFα, EPPTB, RO5166017, T1AM	T1AM, T1AM +EPPTB, T1AM +RO5166017 or 3-iodothyroacetic acid (TA1)	T1AM/TAAR1	↑IL-10 dose-dependent T1AM<br>↓IL-6 dose-dependent T1AM<br>No changes in TAAR1 expression after LPS/TNFα<br>T1AM protective effect after EPPTB<br>↓IL-6 in T1AM+RO5166017<br>TA1 do not affect cells	0.1, 1, and 10 μM of T1AM and TA1, EPPTB at dose 5 nM and RO5166017 at dose 1 μM.
**[219]** Son et al., 2023	Neuroinflammation	Aβ1–42, LPS, LY294002 (a PI3K inhibitor), U0126 (an ERK1/2 activation inhibitor), and PP2	25-hydroxycholesterol (25OHchol), 27-hydroxycholesterol (27OHchol) or polymyxin B	MHC II, Akt, ERK1/2, and Src	↑ IL-1 β, MHC II, and activation of microglia after 25OHchol or 27OHchol<br>↓IL-1β in LPS + polymyxin B<br>polymyxin B had no effect on IL-1β with 25OHchol and 27OHchol<br>No impact on cell viability in 25OHchol and 27OHchol (2 μg/mL)<br>↓IL-1β and MHC II LY294002, U0126, PP2 induced by 25OHchol and 27OHchol	5 and 10 μM of Aβ1–42 or with 25OHchol or 27OHchol (1 μg/mL each) for 48 h.<br>*In vivo:* drug tested also on ApoE-deficient mice.
**[220]** Fu et al., 2023	ALS	GA-DPR (GA 50)	MCC950 (specific inhibitor of the NLRP3), SQOR knockdown or irisflorentin	TLR-7/MyD 88/ NF-κB	↑NLRP3 after GA50<br>↓ NLRP3 after GA50+MCC950<br>↑NLRP3, ROS and cytoplasmic escape of mitochondrial DNA after SQOR knockdown	N/A

(Continued)

10.3389/fimmu.2026.1778798

SUPPLEMENTARY TABLE 1 Continued

ARTICLE	MODEL	TREATMENT	DRUG TESTED	PATHWAY INVOLVED	MAIN RESULTS	NOTES
					↓ NLRP3 after GA50+SQOR with irisflorentin	
**[221]** Peltier et al., 2023	ASD	valproic acid (VPA)	N/A	N/A	↓ ABCA1 mRNA, but ↑ ABCG1 and CD36 mRNA<br>↓ ABCA1 and ABCG1 protein	
**[222]** Lee et al., 2023	Alzheimer's disease, Aging	H2O2	N/A	APOE	↓ MMP, mtDNA CN and cell viability MPP and viability were not restored to the original state, bur mtDNA CNs was after 48 h (c3)<br>↑CASP3, CRYAB, and return to normal level after c2 and c3<br>↑APOE, APOC1, NECTIN2, and TOMM40 and recovery after c3	400 μM H2O2 for 24 h, then replaced with fresh media and continued the culture for an additional 24 h (referred to as "c2") or 48 h (referred to as "c3").
**[223]** Shu et al., 2023	Inflammation (Behçet's disease (BD) and autoimmune uveitis (EAU))	IL-8, DNase I, NETs	N/A	IL-8-induced neutrophil extracellular trap (NET)	↑NET after IL-8 dose-dependent<br>↓viability<br>↑mRNA levels of IL-6, TNFα, and COX2 after NET<br>↺mRNA levels of IL-6, TNFα, and COX2 after NET+ DNase I	IL-8 at doses 20, 50, 100 e 200 ng/mL, NETs 500 (ng/mL)
**[224]** Wang et al., 2023	Angiogenesis	hypoxia, DCA, rotenone or infection with lentivirus containing cDNA of the Flag-tagged YY1 WT or YY1 K183R mutant	CSF1R inhibitor (PLX3397)	lactate/p300/ YY1 lactylation/ FGF2	↑Lactylation, FGF2, p300, HDAC6, SIRT1 and Tip60 by hypoxia<br>↑FGF2, VEGFA and angiogenesis by Hyperlactylated YY1<br>↓FGF2 and angiogenesis by Hyperlactylated YY1+K183<br>↓lactate/lactylation in DCA<br>↑lactate/lactylation with rotenone<br>↑tube formation, spheroid sprouting, migration, and proliferation of HRMECs with HMC3+hypoxia or HMC3+rotenone<br>↓tube formation, spheroid sprouting, migration, and proliferation of HRMECs in HMC3+DCA<br>↑Lactylation promotes angiogenesis<br>Hypoxia increases YY1 lactylation	DCA 20 mM; rotenone 50 nM;<br>A-485 5, 10, and 20 μM,<br>Co-cultured with human retinal microvascular endothelial cells (HRMECs)
**[225]** Wang et al., 2023	Alzheimer's disease	small EVs, macrosomes + Aβ40, transfection with SIL2B-siRNA, proteinase K	N/A	N/A	↑CD63, TSG101, and Alix in small EVs<br>↑Flotillin-2 and ARF6 in macrosomes<br>Lack of APOA1/2, APOB, and ALB in EVs and macrosomes<br>Aβ40+EVs advanced the time of fibril formation from 42 h to 32 h at dose-dependent manner	EVs (50 μg/ml)<br>macrosome (50 μg/ml)<br>proteinase K (100 μg ml^{-1})<br>Aβ40 (25 μM) was incubated with small EVs or macrosomes for 70 hours at 37°C.

(Continued)

10.3389/fimmu.2026.1778798

SUPPLEMENTARY TABLE 1 Continued

ARTICLE	MODEL	TREATMENT	DRUG TESTED	PATHWAY INVOLVED	MAIN RESULTS	NOTES
					↓Time of fibril formation after Aβ40 +Macrosome, also inhibit Aβ42 fibrillation, even at dose 2.5 µg/ml up to 50 hours<br>↑HSPA1B, LAMC1, CD147, P4 hB, and HSP90AB1 inhibit Aβ aggregation in Aβ40+Macrosome<br>SIL2B-siRNA reduced SIL2B mRNA<br>↑fibril formation after Aβ40 +Macrosome+SIL2B-siRNA or proteinase K	
**[226]** Wang et al., 2023	Cancer (various)	siRNA-SETBP1	N/A	N/A	↓Invasion and proliferation after SETMP1 knockdown<br>↑Apoptosis after SETMP1 knockdown	siRNA-SETBP1 100 mM/L.<br>Experiment conducted also on mice brain and TH22 and U87 cell lines.
**[227]** Tomer et al., 2023	Viral infection (HIV)	cGAMP, LPS, PMA/ionomycin	CBD	IFN1	↓Interferon stimulatory genes (ISGs) in CBD+cGAMP, LPS, or PMA/ ionomycin<br>↑ LC3B/p62 but ↓pSTING levels in CBD+cGAMP, LPS, or PMA/ ionomycin	N/A
**[228]** Guo et al., 2023	Alzheimer's disease	presenilin-1 (PS1)-siRNAs	N/A	N/A	↑GIN (induces genome instability), micronuclei, nucleoplasmic bridges and nuclear buds after PS1 knockdown	N/A
**[229]** Guo et al., 2023	Cancer (Glioma)	hypoxya, neuron derived exosomes (NDEs), activated neuron derived exosomes (A-NDEs), co-cultured with hM3Dq overexpressing neurons, ZC3H13-siRNA, DUSP9-siRNA, PD98059 (ERK inhibitor)	transfection with miR-NC or miR-200c-3p mimics	miR-200c-3p/ ZC3H13/ DUSP9/p-ERK	↑CD163, IL10 and TGFB1 after A-NDEs or activated neurons or siRNA-ZC3H13 or siRNA-DUSP9<br>↑M2 polarization and internalization of A–NDEs<br>↓m6A, DUSP9 but ↑p-ERK1/2, ERK1/2 after A-NDEs<br>↓CD163, IL10 and TGFB1 after A-NDEs+miR-200c-3p<br>↓ZC3H13 after miR-200c-3p or siRNA-ZC3H13<br>↓DUSP9 but ↑p-ERK1/2 and ERK1/2 after siRNA-ZC3H13 or DUSP9-siRNA<br>↓pERK1/2, ERK1/2 and CD163 in A-NDEs+PD98059 or miR-200c-3p+ PD98059	Experiment conducted also on LN229 cells, A172 cells, BG5 cells, BG7.
**[230]** Chu et al., 2023	Neuroinflammation	LPS	N/A	N/A	↑TIAM2S in cytoplasmic fraction of HMC3	The other part of the experiment was conducted on human brain samples and TIAM2S transgenic mice.

(Continued)

10.3389/fimmu.2026.1778798

SUPPLEMENTARY TABLE 1 Continued

ARTICLE	MODEL	TREATMENT	DRUG TESTED	PATHWAY INVOLVED	MAIN RESULTS	NOTES
**[231]** Li et al., 2023	Neuroinflammation (Retinal ischemia)	ATP	baicalein	N/A	[Ca^{2+}]i elevation after ATP+Ba stimulation	Retinal ischemia-reperfusion injury was induced in adult C57BL/6J mice.
**[232]** Zou et al., 2023	Cancer (GBM)	transfection with sh LILRB1#1, sh LILRB1#2	N/A	N/A	No results presented	Transwell assay HMC3 cells were sown in the lower chamber and U87, U251 cells were put in the upper chamber.<br>Article analyze GBM samples.
**[233]** Yin et al., 2023	Cancer (GBM)	transfection with shRNA-ABCF1, ABCF1 plasmids	N/A	PI3K/AKT ABCF1-CXCL12-CXCR4	No results presented	Article analyze GBM samples.
**[234]** Chen et al., 2023	Neuroinflammation (SCI)	LPS, IFN-γ, shRNA-Htr2b, RS-127445	N/A	Nrg-1/ErbB	↑Htr2b, M1 polarization and inflammatory cytokines in LPS- and IFN-γ-treated cells<br>↓Htr2b, Iba-1, iNOS and cytokine after shRNA-Htr2b or RS-127445 Htr2b<br>↑Nrg-1 and p-ErbB4 after knockdown of Htr2b<br>↓M1 microglia polarization and inflammatory cytokines after Htr2b-shRNA	N/A
**[235]** Yi et al., 2023	Neuroinflammation (Ischemic injury)	hypoxia in a serum/glucose-free CM, transfection with MiR-148b-3p mimics or miR-148b-3p inhibitor, pcDNA3.1-Notch1, shRNA-Notch1, pcDNA3.1-DLL4, shRNA-DLL4	N/A	DLL4/Notch	↓miR-148b-3p after oxygen-glucose-deprivation/reperfusion (OGD/R)<br>↑miR-148b-3p but ↓viability, migration, TNFα, IL-6, and IL-1β and cycle remain at G0/G1 phase after OGD/R+BMSC-Exo<br>↓DLL4 or Notch1 after miR-148b-3p-mimics<br>↓OGD/R-induced activation, viability, migration, TNFα, IL-6, and IL-1β after knockdown of DLL4 or Notch1	Hypoxic environment (0.2% O2, 94.8% N2, and 5% CO2) in a serum/glucose-free culture medium for 3 h at 37°C.<br>HMC3 cells were co-cultured with Bone mesenchymal stem cell derived exosome (BMSCs-exo) or BMSCs-exo miR-148b-3p inhibitors/mimics/NC at 10 μg/ml for 12 h during OGD/R induction.<br>Delta-like ligand 4(DDL4) or neurogenic locus notch homolog protein 1 (Notch1)
**[236]** Khan et al., 2023	Alzheimer's disease	NSC-exo, LPS	N/A	NF-κB/ERK/JNK	↓NF-B, MAPK, ERK mRNA after NSC-exos<br>↓iNOS, IL-1β, TNFα, and IL-6 after NSC-exos dose-dependent	Exosomes-derived from neuronal stem cells (NSC-exos). The other part of experiment conducted on SH-SY5Y.
**[237]** Muccio et al., 2023	Cancer (GBM)	multicellular tumor spheroids (MTS)	NPs + bortezomib (BTZ)	N/A	↓Cytotoxicity after NPs + BTZ comparing to BTZ alone<br>HMC3 accumulate in the core or vessels (migration towards core) of Tumor Spheroid<br>HMC3 do not influence tumor spheroid behavior<br>↓Viability of spheroids to 20% after 72 hours BTZ-NPs (500 nM)<br>↑infiltration capabilities after NPs + BTZ	Experiment conducted also on U87-MG, GBM-8 and HASTR-ci35 to determine spheroid tumor model: Tumor U87 spheroid and Tumor Mix spheroids<br>(30% or 50%) of HMC3.<br>Core-shell drug-loaded nanoparticles (NPs) loaded with the proteasome inhibitor, Bortezomib (BTZ) using the unencapsulated drug as control.
**[238]** Ahat et al., 2023	Alzheimer's disease	SHIP1 overexpression or heterozygous/homozygous knockout	N/A	TREM	↑PI but ↓PIP species, and lack of PI(3,4)P2 and PI(3,4,5)P3 after SHIP1 overexpression<br>AKT1 lvl in HMC3<BV2<br>TLR4 lvl HMC3>BV2<br>SHIP1 HMC3=BV2	BV2 and HMC3 proteomics comparison.<br>Since SHIP1 functions as a lipid phosphatase that modulates phosphatidylinositol<br>(PI) species.

(Continued)

10.3389/fimmu.2026.1778798

SUPPLEMENTARY TABLE 1 Continued

ARTICLE	MODEL	TREATMENT	DRUG TESTED	PATHWAY INVOLVED	MAIN RESULTS	NOTES
					Lack of TREM2 in HMC3<br>Differences in the transcript level don't correlate with proteome differences	
**[239]**<br>Wiener et al., 2023	Cholesterol metabolism	5 mM MβCD/5 μM rosuvastatin or 10 mM MβCD/5 μM rosuvastatin,<br>T0901317, IL-1β, IL-6, or TNFα	N/A	N/A	↓ABCA7 and LRP1 after MβCD<br>↓ABCA7 after IL-1β and TNFα<br>T0901317 or IL-6 do not affect ABCA7	MβCD cause cholesterol depletion. Phorbol 12-myristate 13-acetate (PMA). Low-density lipoprotein receptor 1 (LRP1).<br>LXR agonist T0901317.
**[240]**<br>Tang et al., 2023	Neuroinflammation (ICH)	Kallikrein-related peptidase 8 (KLK8)	Maraviroc (MVC, CCR5 inhibitor)	Chemokine receptor 5 (CCR5)/PKA/CREB	MVC binds to CCR5 and CC chemokine ligand (CCL14) in HMC3 cells.<br>↑chemotactic activity after KLK8+CCL4<br>↓chemotactic activity after KLK8+CCL4+MVC<br>↑CCL14/CCR5 after KLK8<br>↓CCL14/CCR5 after KLK8+MVC<br>microglial activation and ICH aggravation after KLK8 overexpression via CCL14	
**[241]**<br>Ni et al., 2023	Alzheimer's disease	LPS, Aβ	Levistilide A (LA)	JAK2/STAT3	↓IL-1β, IL-6 and TNFα after LPS/Aβ+LA<br>↑IL-4 and IL-10 LA+LPS/Aβ<br>reverses M1 to M2 phenotype inhibited ↓Iba-1, IL-1β, IL-6, TNFα and NOS2 after LA<br>↑ARG1, CD206 and CD163 after LA<br>↑↓STAT3 and phosphorylation of JAK2 and STAT3 after LA	The CM from LA-treated BV2 or HMC3 cells enhances the viability of SH-SY5Y and HT-22 cells
**[242]**<br>Zhou et al., 2023	Alzheimer's disease	LPS, transfection with overexpression NEAT1 plasmid, a miR-361-3p mimic/inhibitor and a small interfering (si)-tumor necrosis factor receptor-associated factor 2 (TRAF2) plasmid	Triptolide (Tri)	NEAT1/miR-361-3p/TRAF2	↑Apoptosis, IL-1β, IL-6, IL-18, TNFα and ↓viability after LPS<br>↓LPS-mediated effects in a dose-dependent manner after Tri<br>↓NEAT1 and TRAF2 after Tri+LPS<br>↑miR-361-3p after Tri+LPS dose-dependent<br>↓LPS-mediated HMC3 cell injury, NF-κB after Tri or miR-361-3p-mimic	pre-treated with Tri (5, 10, 20, 50, and 100 nM) for 24 h and then stimulated with LPS (100 ng/ml) for 24 h.<br>Nuclear paraspeckle assembly transcript 1 (NEAT1)
**[243]**<br>Chen et al., 2023	Cancer (Non-small cell lung cancer)	Transfection with miR1246 mimic or miR1246 inhibitor,<br>A549-exosomes	N/A	N/A	HMC3 take up A549-derived exosomes and became rounder and larger and had more branches<br>↑Proliferation after exosomes 12 h, but not migration or inflammatory markers (CD16, CD80, Arg1, MRC1)<br>↑Phagocytosis and secretions of IL-6, IL-8, and CXCL1 after exosomes<br>↑IL-6, IL-8, CXCL1 and miR1246 after exosomes +miR1246 mimic<br>↓IL-8 and miR1246 after exosomes+miR1246 inhibitor	The miR1246 mimic and miR1246 inhibitor at concentration of 100 nM 24 h of transfection.<br>Exosomes derived from A549 (20 ng/mL) were labeled with PKH67 and incubated with HMC3 cells at 37°C for 12, 24 or 42 h.

(Continued)

10.3389/fimmu.2026.1778798

SUPPLEMENTARY TABLE 1 Continued

ARTICLE	MODEL	TREATMENT	DRUG TESTED	PATHWAY INVOLVED	MAIN RESULTS	NOTES
**[244]**<br>Chen et al., 2023	Diabetic retinopathy (DR)	LPS, PIK3IP1-overexpressed	N/A	PI3K-AKT	↑M1phenotype after LPS<br>↓M1phenotype after LPS + PIK3IP1-OE<br>↑VEGFA, FGF2, HGFα and MMP9after LPS<br>↓VEGFA, FGF2, HGFα and MMP9after LPS + PIK3IP1-OE<br>↑PI3K, AKT, and p-AKT after LPS dose-dependent<br>↓AKT phosphorylation, with no effects on total AKT after LPS + PIK3IP1-OE	LPS for 24 h<br>*In vivo:* Oxygen-induced retinopathy (OIR) mice.
**[245]**<br>Chen et al., 2023	Cancer (GBM)	transfection with siRNA-GPX8, GBM-derived GPX8 co-cultured with U251	N/A	GPX8/IL-6/ STAT3	↓Migration of HMC3 cells in a co-culture after siRNA-GPX8<br>↑Migration of HMC3 cells in co-culture after GPX8	siRNA-GPX8 inhibits GPX8 expression that is high in glioma cells
**[246]**<br>Hu et al., 2023	Neurosyphilis (*Treponema pallidum*)	*Treponema pallidum* (Tp), 3-MA	Rapamycin (RAP)	mTORC1/ TFEB	↑MHC-II, BA-1 and cell activation after Tp dose-dependent<br>↑PTGES mRNA and apoptosis after Tp<br>↓Autophagy, DDIT3, HSPA5 and SESN2 mRNA after Tp<br>↑Conversion of LC3-I to LC3-II, P62 and Beclin 1 after Tp dose-dependent<br>↑Autophagosomes and ↓autolysosomes after Tp<br>↓Lamp2, TFEB after Tp<br>↑Lamp2, TFEB and autolysosomes after Tp+RAP<br>↓Apoptosis, autophagosome and Tp polA mRNA/ DNA after Tp+3-MA	Culturing HMC3 cells with Tp (MOI of 10:1, 50:1, 100:1, or 150:1) for 24 h. mTORC1 signaling inhibitor RAP (rapamycin, 100 nM) for 2 h.<br>3-MA (3-methyladenine, autophagosome formation inhibitor, 2 mM) for 24 h
**[247]**<br>Zhao et al., 2023	Alzheimer's disease	Aβ	NB, NBIL, PNBIL	N/A	↓Viability after H_2O_2 ± NBIL<br>↑ROS after H_2O_2 ± NBIL<br>↑Viability after H_2O_2+PNBIL<br>↓ROS after H_2O_2+PNBIL<br>↓IL-1β, IL-6, GM-CSF, ROS after Aβ+PNBIL<br>↑Phagocytosis, ROS after Aβ+PNBIL<br>↓Aβ aggregates after PNBIL	Multivalent Nanobody Conjugate PDDA-NBIL (PNBIL) 8 μm (containing 50 μg mL−1 of PDDA)
**[248]**<br>Zhao et al., 2023	Diabetes	High glucose (HG), LP17	N/A	Lipid peroxidation and iron deposition	↓FPN1 and GPX4, but ↑TFR1, FTL, ROS and Fe^{2+} in HG<br>↑TREM-1 in HG<br>↑FPN1, GPX4, FTL but ↓TFR1 and ROS in HG +LP17<br>↑Iron deposition and lipid peroxidation by HG	HG for 72 h in BV2 or HMC3 and another one high fat diet (HFD) combined with streptozotocin (STZ) in C57 mice.<br>The inhibitor of TREM1 LP17 (30mg/mL) was added to Cultures with 25mmol/L glucose.
**[249]**<br>Zhang et al., 2023	Parkinson's disease	Oligomeric α-Syn, Stattic	N/A	IL6ST/STAT3/ HIF-1α	↑Cytotoxicity<br>↑ROS in lower concentration but ↓ROS in higher concentration of α-Syn<br>↓IL6ST, JAK2, STAT3 and HIF1α after α-Syn<br>↑ferroptosis regulation genes (FRG): TFRC,	α-Syn at < 5 μmol/L or 10–25 μmol/L for 24 h,<br>*In vivo*: PD mouse models.<br>STAT3 activator (Colivelin TFA) and the STAT3 inhibitor (Stattic)

(Continued)

10.3389/fimmu.2026.1778798

SUPPLEMENTARY TABLE 1 Continued

ARTICLE	MODEL	TREATMENT	DRUG TESTED	PATHWAY INVOLVED	MAIN RESULTS	NOTES
					CHAC1, NFE2L2, FTH1, GPX4 and HSPB1 after α-Syn<br>↑PSTAT3, HIF-1α, HSPB1 gene and FRG after α-Syn + Colivelin TFA<br>↓PSTAT3, HIF-1α and FRG but ↑HSPB1 gene after α-Syn + Stattic	
**[250]** Zhang et al., 2023	Cancer (Glioma)	hyaluronidase (HA), rCHI3L1, co-cultured with HS683, U251, and U87 cells	N/A	IL-1/CHI3L1, TGF-b/CHI3L1	↓Migration, M2 polarization markers (CD68/CD163) after HA<br>↑Migration after HA+rCHI3L1	The concentration gradient of hyaluronidase was set as 100, 200, 500, 1000 μg·mL−1
**[251]** Nicholson et al., 2023	Neuroinflammation (Multinucleated microglia)	Phorbol-myristate-acetate (PMA)	N/A	p38/MAPK	↑multinuclearity (lack of both clear nuclear envelops and centrally localized nuclei) after PMA 24/48 h<br>sheet-like connections between cells after PMA 72 h<br>similar phagocytic ability between mononucleated and multinucleated cells	Lead treatment on primary rat microglial cells.
**[252]** Chmielarz et al., 2023	Neuroinflammation (PA)	IFNγ	LPS and PA directly or indirectly (CM)	N/A	↑Viability and proliferation does not change after LPS or PA<br>↑IL-6, MCP-1 (in medium), PGE2 (cell lysate), ROS, COX-2 and lipid peroxidation after LPS + PA directly but less effect after indirect treatment<br>↓IL-6, MCP-1, ROS after LPS + PA+IFNγ directly or indirectly (except LPS directly)	The cells were then stimulated with IFNγ at 400 U/mL for 24 h. After that, HMC3 cells were treated with LPS alone at 5 ng/mL, PA alone at 200 μM, or a combination of LPS and PA at the same concentrations for 20 h.
**[253]** Son et al., 2023	Neuroinflammation, Alzheimer's disease	25-hydroxycholsterol (25OHchol), and 27-hydroxycholesterol (27OHchol), Aβ1–42, LPS, LY294002, U0126, or PP2.	polymyxin B	Akt/ERK/Src	↑IL-1β at both the transcript and protein levels and ↑MHC II after 25OHchol and 27OHchol<br>↑ IL-1β and MHC II stable after cholesterol or 24sOHchol<br>↑IL-1β mRNA and protein but ↓viability after Aβ1–42 10 μM<br>↑IL-1 β after LPS or 25OHchol/27OHchol +Polymyxin B<br>↓IL-1β after LPS + Polymyxin B<br>↓MHC II after 25OHchol/27OHchol+LY294002/U0126/PP2	25OHchol or 27OHchol (1 μg/ml each) did not change the viability of HMC3. Treatment with Aβ1–42 at dose 5 or 10 μM for 48 h. Protein kinase inhibitors on MHC class II expression: LY294002, U0126, or PP2.<br>*In vivo:* ApoE-deficient mice fed a HFD rich in cholesterol.
**[254]** Ahuja et al., 2023	Neuroinflammation	cytokines (IL-4, IL-10, IL-13, TGFβ1, TGFβ2, CCL2)	N/A	N/A	↑Endosomes (6-folds) after cytokinces<br>↑MHC I, phagosome and antigen cross-presentation (5-folds) after cytokines<br>↑Immune response, antigen processing and presentation of peptide antigen (4-folds) after cytokines<br>↑Myeloid cell activation, MHC I antigen processing and presentation, innate immune system (2-folds) after cytokines	Serum-depleted (48 h) and non-depleted HMC3 cells were treated with anti-inflammatory cytokines: IL-4 (40 ng/mL), IL-10 (20 ng/mL), IL-13 (20 ng/ml), TGFβ1 (20 ng/ml), TGFβ2 (20 ng/ml) e CCL2, so MCP-1 (40 ng/ml) for 24 h

(Continued)

10.3389/fimmu.2026.1778798

SUPPLEMENTARY TABLE 1 Continued

ARTICLE	MODEL	TREATMENT	DRUG TESTED	PATHWAY INVOLVED	MAIN RESULTS	NOTES
**[255]** Decarpentrie et al., 2023	Cancer (GBM)	N/A	N/A	N/A	Comparison of epigenetic profile. No direct results about HMC3	SF-126, U-87MG, U-118MG, and U-138MG compared to HMC3
**[256]** Decarpentrie et al., 2023	Cancer (GBM)	N/A	N/A	N/A	Comparison of epigenetic profile. No direct results about HMC3	SF-126, U-87MG, U-118MG, and U-138MG compared to HMC3
**[257]** Emezienna et al., 2023	Neuroinflammation (Fetal)	SVGp12 co-culture, Amniotic fluid (AF)	N/A	N/A	Lack of cell migration, glial activation, or inflammatory changes after LPS + AF<br>↑IL-6 in male > female after AF<br>↑microglia activation, CD11b in male after LPS + AF<br>↓Iba1 in male after LPS + AF<br>↓IL-1b in WH after LPS + AF<br>↓IL-8 in AA after LPS + AF	Co-culture with SVGp12 at 80:20 (inner chamber). The outer chamber - amniotic fluid (AF) from male and female fetuses of White Hispanic (WH) and African-American (AA) pregnant women ± LPS 100ng/ml 48 h
**[258]** Phoraksa et al., 2023	Neurotoxicity (*Albizia lebbeck*)	glutamate	*Albizia lebbeck*	MAPK/ERK	↑Protective and anti-apoptotic effect of Bcl-2 after AHE compared to AME and AEE<br>↓ER stress proteins (calpain1 and caspase-12), the apoptotic proteins (Bax, cytochrome c, cleaved caspase-9, and cleaved caspase-3) and cellular antioxidant activities (SOD, CAT, and GPx) after AHE<br>↑Carotenoids (α-carotene, β-carotene, and lutein) and flavonoids (quercetin, luteolin, and kaempferol) after AHE<br>↓viability and round with abnormal shape cells after glutamate<br>↑p-ERK expression with no effect on p-p38 and p-JNK after glutamate<br>↓p-ERK expression but ↑p-p38 and p-JNK after AHE+glutamate<br>↑calpain1, Bax and cleaved caspase-12 but ↓Bcl-2 after glutamate<br>↓Calpain1, Bax and cleaved caspase-12 but ↑Bcl-2 after AHE+glutamate<br>↓neurotoxicity (ER stress and apoptosis) with normal morphology after AHE+glutamate	Glutamate at various concentrations (0–70 mM) and three different A. lebbeck leaf extracts (A. lebbeck leaves were extracted using hexane (AHE), mixed solvents (AME) and ethanol (AEE) at various concentrations (0–100 µg/mL) for 24 h.<br>AHE was selected for pre-treatement 0.5–10 µg/mL for 24 h, followed by 50 mM glutamate.
**[259]** He et al., 2023	Inflammation (Uveitis)	LPS/IFN-γ,<br>transfection with shFTO, YTHDF3 siRNAs or lentiviral shGPC4 vectors<br>co-cultured with CD4+ T cells	N/A	TLR4/NF-κB	↑M1 markers: iNOS, TNFα, IL6 and migration after LPS + IFN-γ<br>↓FTO and the reader YTHDF3 after LPS + IFN-γ<br>↑m6A modification, iNOS, TNFα, IL6 and CXCL10 after shFTO<br>↑migration after shFTO+LPS + IFN-γ<br>↑m6A methylation of GPC4 and stability after	LPS (1 µg/mL) and IFN-γ (500 ng/mL).<br>From three siRNAs, the siYTHDF3-1 has the highest knockdown efficiency.<br>*In vivo:*with FTO-deficient mice with EAU exhibited

(Continued)

10.3389/fimmu.2026.1778798

SUPPLEMENTARY TABLE 1 Continued

ARTICLE	MODEL	TREATMENT	DRUG TESTED	PATHWAY INVOLVED	MAIN RESULTS	NOTES
					shFTO<br>↑GPC4 mRNA and stability after shFTO + siYTHDF3<br>↑iNOS, TNFα, IL6 but CXCL10 after shGPC4 +shFTO+LPS + IFN-γ<br>↓CXCL10 shGPC4+shFTO<br>↑NF-κB p65 phosphorylation and CD14 and TLR4 after shFTO+LPS + IFN-γ<br>↑NF-κB p65 phosphorylation and CD14 and TLR4 after shGPC4+shFTO+LPS + IFN-γ	
**[260]** Das et al., 2023	Alzheimer's disease	rs242557 knockout	N/A	N/A	272 up- and 861 down-regulated transcripts between WT and KO cells<br>↓ICA1, EPDR1, PTK2B, SORL1, DOC2A, and TSPOAP1 transcripts (connected to AD) and SNCA (connected to PD) after KO<br>↑CASS4 transcript after KO	CRISPR-Cas9 genome editing
**[261]** Xie et al., 2023	Neurosyphilis (*Treponema pallidum*)	*Treponema pallidum* membrane protein (Tp47) alone or in combination with the FOXO1 inhibitor (AS1842856),<br>anti-Tp47 antibody	N/A	PI3K/AKT/ FOXO1	↓Migration and P62 after Tp47<br>↑Beclin-1, LC3-II/LC3-I, and autophagic flux after Tp47<br>↓Autophagy but ↑migration after anti-Tp47<br>↓p-PI3K, p-AKT, and p-mTOR after Tp47-induced autophagy<br>↓p-FOXO1 and FOXO1 nuclear translocation after Tp47<br>↓Migration and Tp47-induced autophagy after AS1842856	N/A
**[262]** Mathen et al., 2023	Neurotoxicity (Glial Crosstalk)	isolated CM or CM after siRNA vector for Sirt2	N/A	Wntβ catenin	↑IL-6, IL-8, GM-CSF and VEGF after CM disruptions in glutamate neurotransmitter metabolism and actin cytoskeletal alterations (changes in pseudopodia and lamellipodia) after CM+Sirt2 KO<br>↑connexin 43 in gap junctions with no effect on E-cadherin in adherens junctions after CM	CM isolated from irradiated glial cells treated with single-dose or fractionated radiation schedules, then transfected with siRNA expression vector for Sirt2, a class III HDAC, versus scrambled controls.
**[263]** Chi et al., 2023	ALS	IFNγ, siRNA CIITA, siRNA FUSR495X	N/A	MHC II	↓DRA, DRB1, CD74 and CIITA after siRNA-CIITA or siRNA-FUS<br>↓HLA-DR siRNA-FUS+IFNγ<br>HLA-A and NLRC5 unchanged after siRNA-FUS +IFNγ	3 ng/mL of IFNγ (24 h).<br>The other part of experiment conducted on HeLa, HPCs or ES cells.
**[264]** Chang et al., 2023	Alzheimer's disease, Cancer (GBM)	co-electroporation of Cas9 RNP for sgRNA-CD40, sgRNA-MERKT, sgRNA-CD47, sg-SIRPA,<br>sgRNA-RAB11A, sgRNA-ACTB	N/A	N/A	↑CD40, P2RY12, CD16, SIRPα, CD47 markers in HMC3<br>↓CD14, CD22, CD96, MERTK markers in HMC3<br>Lack of CD206, CD11b, CD45, CD68, HLA-DR, IL4RA, TGFBRII, CX3CR1, CD64, CD33, Siglec10,	fAβ1-42 (1.5 μM in a 400 μL)<br>Co-cultured with live or apoptotic LN-229 for 24 h

(Continued)

10.3389/fimmu.2026.1778798

SUPPLEMENTARY TABLE 1 Continued

ARTICLE	MODEL	TREATMENT	DRUG TESTED	PATHWAY INVOLVED	MAIN RESULTS	NOTES
		fAβ1-42 aggregates,<br>co-cultured with GFP+ LN-229			PD1, LILRB2, Tim-3, TREM2, CD36 markers in HMC3<br>parental HMC3: 36.8% CD40+ MERTK+ and 61.1% CD40+ MERTK–<br>Knockout sgRNA-CD40, sgRNA-MERKT HMC3: 22.8% CD40+ MERTK– and 72.9% CD40– MERTK–<br>↓Viability to 50% after double KO<br>↑Efficiency after double CD47/SIRPA KO than single KO<br>20-30% of HA+ cells after RAB11A and ACTB KI<br>10% TERM+ cells after SFFV-KI<br>30% CD14+ cells after SFFV-KI<br>↑Phagocytosis after fAβ1-42 aggregated<br>10% phagocytosis after fAβ1-42 in HMC3 and CD47- +<br>15-30% phagocytosis after fAβ1-42 in TREM+ and CD14+<br>5% phagocytosis in SIRPα- and MERTK- cells with liveLN-229<br>10% phagocytosis in TREM2+ cells with live LN-229<br>10% phagocytosis in HMC3, SIRPα- and MERTK- cells with apoptotic LN-229<br>17% phagocytosis in TREM2+ cells with apoptotic LN-229	
**[265]**<br>James et al., 2023	Hyperglycemia	glucose	Immunoglobulin heavy chain protein (BIP) inducer-X (BIX).	N/A	↓BIP after hyperglycemia<br>↑apoptosis, p-eIF2α, CHOP, Bax, Bad, ROS and cleaved caspase-3 after hyperglycemia<br>↓apoptosis, p-eIF2α, CHOP and ER stress but ↑BIP after BIX+hyperglycemia<br>No changes in LC3-BII and autophagy after BIX	cells were cultured in 25 mM glucose, treated with 25 µM BIX on day 3, and cultured until day 5
**[266]**<br>Tang et al., 2023	Viral infection (EV71)	EV71+ miR-342-5p mimics/inhibitor targets CTNNBIP1	N/A	Wnt/CINNBI	↑EV71 VP1 mRNA and protein expression, elevated TNFα, IL-6, and IL-10 levels, but ↓IFN-β levels after miR-342-5p<br>↓miR-342-5p in EV71 replication, and inhibited TNFα, IL-6, and IL-10 levels, but ↑IFN-β levels after CTNNBIP1<br>↑TCF4/CTNNB1, EV71 replication but ↓CTNNBIP1 and IFN after miR-342-5p<br>miR-342-5p targets 3' UTR of CTNNBIP1	Infection of EV71 at MOI = 2
**[267]**<br>Li et al., 2023	Neuroinflammation (Ischemic stroke)	OGD, shRNA-XIST (shXIST), miR-25-3p mimics/inhibitor, siTRAF3	N/A	N/A	↑XIST but ↓miR-25-3p after OGD/R<br>↓XIST, IL-1β, IL-6, IL-8, and TNFα after OGD/R + shXIST	N/A

(Continued)

10.3389/fimmu.2026.1778798

SUPPLEMENTARY TABLE 1 Continued

ARTICLE	MODEL	TREATMENT	DRUG TESTED	PATHWAY INVOLVED	MAIN RESULTS	NOTES
					↓Bcl-2 but ↑Bax, Bad and cleaved caspase 3 after OGD/R<br>↑Bcl-2 but ↓Bax, Bad and cleaved caspase 3 after OGD/R + shXIST<br>↓apoptosis and inflammatory after OGD/R + shXIST + miR-25-3p<br>↓TRAF after miR-25-3p<br>XIST worked as a miR-25-3p sponge to inhibit its expression<br>OGD/R-induced injury after TRAF knockdown	
**[268]** Liu et al., 2023	Neuroinflammation (Neuropathic pain)	LPS, siDDX54, pcMYD88	N/A	NF-κB/NLRP3	↑IL-1β, TNFα, IL-6, DDX54, MYD88, NF-κB, and NLRP3 after LPS<br>↓IL-1β, TNFα, IL-6, MYD88, p-NF-κB p65 (p-p65), and NLRP3 after LPS + DDX54 knockdown<br>↑Stability of MYD88 mRNA after DDX54 overexpression	*In vivo:* experiment performed also on chronic constriction injury (CCI) rat model.
**[269]** Liu et al., 2023	Cancer (Glioma)	Bacoside A (Gyp)	N/A	N/A	No changes in viability after Gyp, IC50 = 864.0 μg/ml	HMC3 used only to assess the viability after Gyp
**[270]** Herrera et al., 2023	Alzheimer's disease	cholesterol (chol), Aβ oligomers (AβO), LPS, and fructose	N/A	N/A	↑IL-6 after chol + LPS or fructose + LPS or LPS alone<br>↓IL-1B after chol + AβO or chol + AβO+LPS or chol alone<br>cellular chol after fructose, or fructose + chol, or chol + AβO+LPS<br>cholesteryl esters (CE) after fructose or fructose + AβO or fructose + LPS or AβO + LPS + chol or AβO + LPS + chol + fructose<br>↓ApoE after chol + AβO alone or + fructose + LPS (stronger effect)<br>↑APOE and TNFα expression, ROS and mitochondrial chol but ↓ATP and phagocytosis after chol + AβO + fructose + LPS	20μg/ml chol (t=18), 100ng/ml LPS (t=21), 2μM Aβ (t=0), and 50mM fructose (t=0) individually and in combination for 24 h
**[271]** Kim et al., 2023	Neurotoxicity (UV-weathered MP)	Weathered MPs (WMPs) and virgin MPs (VMPs)	N/A	N/A	↑Severe inflammatory response after WMP than VMP<br>↑Microglia activation after WMP	
**[272]** Panda et al., 2023	Neuroinflammation	CM from particulate matter (PM2.5) exposed BEAS2B cells	melatonin (ML)	N/A	↓Microglia activation after CM + ML<br>↑Iba-1, CD68 and microglia activation after CM<br>↑oxidative stress after CM	PM2.5 conc. 10, 20, 40, 60, 80, 100 and 120 μg/mL
**[273]** Zhang et al., 2023	Neuroinflammation (Neuropathic pain)	LPS, IL-4	daphnetin	NF-κB	↑Polarization, M1 phenotype, IL-6 and TNFα after LPS<br>↑IL-1β after LPS 100 μg/mL	LPS (100, 500 ng/mL, 1, 100, and 500 μg/mL) and daphnetin (2.5, 5, 10, 20, and 40 μg/mL) is extracted from the *Daphne giraldii Nitsche* and has the

(Continued)

SUPPLEMENTARY TABLE 1 Continued

ARTICLE	MODEL	TREATMENT	DRUG TESTED	PATHWAY INVOLVED	MAIN RESULTS	NOTES
					↓viability after daphnetin concentration-dependent (more than 20 µg/mL)<br>↓IL-1β, IL-6, and TNFα after LPS + daphnetin<br>↑IL-10 after IL-4 or IL-4 + daphnetin<br>↓CD206 after LPS<br>↑PolarizationM0/M2 after Il-4<br>↓PolarizationM0/M1 but ↑polarizationM1/M2 after daphnetin<br>↓Polarization and M1 phenotype, but ↑M2 phenotype after LPS + daphnetin	structure of 7,8-dihydroxy coumarin. IL-4 (2.5, 5, 10, 20, and 40 µg/mL) for 24 h
**[274]** Pradhan et al., 2023	Neuroinflammation	CM after U937 exposed to diesel particulate matter (DPM)	N/A	N/A	Microglia significantly upregulated ↑IL-6, IL-8, IL-1β, TNFα and oxidative stress after CM-DPM<br>↑CD14 and microglia activation after CM-DPM more than direct DPM	Alveolar macrophages (transformed U937) were inoculated with resuspended DPM 24 h, 2.5 µg/mL. HMC3 incubated with CM for 48 h
**[275]** Li et al., 2023	Diabetes	Hyperglicemic plasma, HG, LPS, PA or oleic acid (OA), LP17	N/A	N/A	↑PLIN2, SQSTM1 and TREM after Hyperglycemic palsma<br>↓LC3B cells after T2DM palsma<br>↑PLIN2+ LDs and SQSTM1 after glucose 25mM<br>↓LC3B after glucose 25mM<br>↓TREM1 and TREM2 after HG<br>↑LDs after LPS, PA and OA<br>↑TREM1 clusters with PLIN2+ LDs after Hyperglycemic plasma<br>↑aggregation of SQSTM1 with PLIN2 after HG +LP17<br>↓p-SYK, LC3B-II, SQSTM1, PLIN2 and NLRP3 after HG+LP17<br>↑IL6, NOS2, TNF, IL1B/IL-1β and ROS after HG<br>↓IL6, NOS2, TNF, IL1B/IL-1β and ROS after HG +LP17	Hyperglycemic plasma isolated from T2DM patients. 5.5 mM and 25 mM glucose.
**[276]** Fan et al., 2023	Neuroinflammation	LPS/IFN-γ	parthenolide (PTL)	AKT/MAPK/ NF-κB, MAPK/ TRIM31/ NLRP3	↓IL-6, IL-1β, TNFα and ROS after LPS/IFN-γ+PTL<br>↺Microglial phagocytic activity after LPS/IFN-γ+PTL<br>↑IL-10 and TRIM31 after LPS/IFN-γ+PTL<br>↑TRIM31 after PTL	N/A
**[277]** Ren et al., 2023	Neuroinflammation (Allergic rhinitis - Olfactory dysfunction)	CM from HNEpC+Der p1 ± JNJ- 47965567 (P2X7R inhibitor)	N/A	ATP/P2X7R/ Caspase 1	P2X7R expressed in HMC3<br>↑Caspase 1 after CM+ATP<br>↓IL-1Ra after CM<br>↑IL-1Ra after CM<br>↑enlarged soma, shortened protrusions, IL-1β after CM	N/A

(Continued)

10.3389/fimmu.2026.1778798

SUPPLEMENTARY TABLE 1 Continued

ARTICLE	MODEL	TREATMENT	DRUG TESTED	PATHWAY INVOLVED	MAIN RESULTS	NOTES
					↓enlarged soma, shortened protrusions, IL-1β after CM+JNJ- 47965567	
**[278]**<br>Schlotterose et al., 2023	Neuroinflammation	recombinant human TNFα	Resveratrol	N/A	↑IL6, IL1β, and TGFβ after TNFα 6 h<br>↑Cell activation after TNFα 24 h<br>↓cell activation and cytokine expression levels after TNFα+resveratrol	24 h with 5 nM recombinant human TNFα and/or 100 μM resveratrol.
**[279]**<br>Schlotterose et al., 2023	Neuroinflammation	resveratrol	N/A	N/A	↑IGF-1 after reserveratrol<br>↓IL18, IL1β, caspase-1 and inflammasome activity after resveratrol<br>No changes on profileration or morphology after resveratrol<br>↓Glucose uptake (50%) and mitochondrial activity after resveratrol<br>↑Amino acids (Val, Ile, Phe, Tyr, Gly, Ala) but ↓Fum, Glu and metabolism after resveratrol	resveratrol (100 μM) treatment for 6/24 h
**[280]**<br>Dhyani et al., 2023	Hypoxia	gas-environment-based hypoxia, reoxygenation	Nifedipine (L-type calcium channel blocker)	N/A	↑Cytosolic Ca2+, HIF1A, OXR1 but ↓viability after hypoxia<br>↑ROS, H2O2 and but ↓viability after hypoxia<br>↓Cytosolic Ca2+, HIF1A, OXR1 but ↑viability after hypoxia+nifedipine<br>↑Cytosolic Ca2+, HIF1A, OXR1 but ↑viability after hypoxia+reoxygenation	
**[281]**<br>Zhou et al., 2023	Neuroinflammation	APOE2, APOE3, APOE4, APOE4 mutants (W39A, R114A, W39A/R114A)<br>sgLilrB3-null plasmid	N/A	N/A	167 genes affected after APOE4+ WT<br>↑IFITM3, BST2, MX1, ISG15 after APOE4+ WT<br>↓TNFRSF10D after APOE4+ WT<br>↑18 genes and ↓1 gene after APOE4+ LilrB3-null<br>No changes in TNFRSF10D after APOE4+ LilrB3-null<br>↓APOE4+LilrB3 and microglia activation after W39A, R114A or W39A/R114A	
**[282]**<br>Peng et al., 2023	Viral Infection (HIV-1)	HIV-1 proviral plasmid pNL4-3 (HIV-1-EGFP)	N/A	MAPK/Hippo/PI3K-Akt/Ras, Ras/MAPK/Rap1/PI3K-Akt	↑m6A after HIV-1-EGFP in virus-dose-dependent<br>1109 hyper-methylation and 1055 hypo-methylation peaks of m6A after HIV-1-EGFP<br>↑m6A peaks connected to Ras/MAPK/Rap1/PI3K-Akt pathway after HIV-1-EGFP<br>↑m6A level HLA-B, CFB, and OLR1 in m6A +HIV-1-EGFP<br>↓m6A peaks connected to MAPK/Hippo/PI3K-Akt/Ras after HIV-1-EGFP<br>↓m6A level IL-6 RNA in m6A+HIV-1-EGFP	pseudotyped HIV-1-EGFP at 100 ng (or 0, 25, 50, and 100 ng) of p24/mL for 48 h

(Continued)

10.3389/fimmu.2026.1778798

SUPPLEMENTARY TABLE 1 Continued

ARTICLE	MODEL	TREATMENT	DRUG TESTED	PATHWAY INVOLVED	MAIN RESULTS	NOTES
**[213]** Wagner et al., 2023	Neuroinflammation (*Amanita muscaria*)	TNFα, poly(I:C), substance P and LPS	AME-1 from *A. muscaria*	Surface receptors	↑CD86, CXCR4, CD45, CD125 and TLR4<br>↑IL–8 afterAME-1 at higher concentrations<br>↑IL-8 with AME-1 or its component trealose in response to poly(I:C).<br>Altered expression of TLR3 mRNA but not surface protein.<br>AME-1 also did not significantly alter expression of retinoic acid-inducible gene I or melanoma differentiation-associated protein 5.	LPS (1 mg/mL), poly(I:C) (10 mg/mL), recombinant human TNFα (100 µg/mL), Trehalose (250 mM) and AME-1 (diluted 1:2) Substance P was prepared in 0.1 M acetic acid to a concentration of 1 mg/mL. TNFα, poly(I:C), substance P and LPS used for comparison
**[284]** Lucchi et al., 2023	Neuroinflammation	rotenone	exogenous allopregnanolone	N/A	↑IL-6 and ROS by 40+ after rotenone 24 h (no effect on viability)<br>↓Cell viability after rotenone 48 h<br>↓Neurosteroids (pregnenolone, pregnenolone sulfate, 5α-DHP, and pregnanolone) but ↑allopregnanolone after rotenone 24 h<br>↺Cell viability after exogenous allopregnanolone +rotenone 48 h	rotenone (100 nM) for 24 h and 48 h. Exogenous allopregnanolone (1 nM)
**[285]** Bahader et al., 2023	Neuroinflammation (Brain injury)	H2O2	cofilin inhibitor (CI)	N/A	↑Cofilin and SSH1 with no effect on p-cofilin after H2O2 200µM<br>↓Cofilin, SSH1 and cofilin/p-cofilin ratio after H2O2+CI<br>↑ High Mobility Group Box 1 (HMGB1) and TNFα after H2O2<br>↓HMGB1 and TNFα after H2O2+CI<br>No effect on total NF-κB after H2O2 or H2O2+CI	H2O2 (100 µM or 200 µM) for 24 h, then 200 µM H2O2 in the presence of 10 µM CI or vehicle for 24 h
**[286]** Wei et al., 2023	Neuroinflammation (SCI)	LPS	Bone morphogenetic protein 7 (rhBMP7) overexpression via adeno-associated virus	N/A	↑viability after LPS<br>↓viability and M1 phenotype after LPS + BMP7<br>↑Iba-1 and p-STAT3 after LPS or LPS + BMP7 (higher result)<br>No changes in STAT3 after LPS or LPS + BMP7<br>↑CD86, iNos but ↓arginase1, CD206 after LPS<br>↓CD86, iNos but ↑arginase1, CD206 after LPS + BMP7<br>↑CD86+/Iba1+ but ↓Arg1+/Iba1+ after LPS<br>↓CD86+/Iba1+ but ↑Arg1+/Iba1+ after LPS + BMP7<br>↑p-STAT3, M2 phenotype but ↓IL-1β and TNFα after LPS + BMP7	LPS or LPS + BMP7 (100 ng/ml each) 6 h.
**[287]** Wei et al., 2023	Neuroinflammation	plasma-derived EVs from preeclampsia patient	N/A	N/A	↑PE-EVs uptake in co-culture<br>↑CD11b, IBA1, TNFα, IL-1β and IFN-γ	A co-culture of hCMEC/D3 cells and HMC3 as the two-compartment static model of the BBB.

(Continued)

10.3389/fimmu.2026.1778798

SUPPLEMENTARY TABLE 1 Continued

ARTICLE	MODEL	TREATMENT	DRUG TESTED	PATHWAY INVOLVED	MAIN RESULTS	NOTES
**[288]** Aquino et al., 2023	Neuroinflammation	Diesel Exhaust Particle (DEP)	N/A	N/A	↑GM-CSF and less ↑IL-1, IL-6, TNFα in DEP+co-culture but not in the monolayer<br>No changes in RHO123 accumulation DEP+mono/co-culture<br>No changes in transcriptional or functional modulation of the<br>P-gp efflux transporter in co-culture+DEP<br>↑Fractalkine, IL-4, IL-10, IL-13, IFNg, IL-1β, IL-6 in co-culutre >monolayer (still down-reg.)<br>↓TEER after co-culture+DEP, but not in monoculture<br>↑negative effect of DEP exposure on barrier permeability in co-culture	The effect of DEP exposure (2000 μg/ml) in human *in vitro* BBB model (hCMEC/D3) with/without hMC3
**[289]** Wei et al., 2023	Alzheimer's disease	LPS or Aβ	N,N-dimethylacetamide (DMA)	NF-κB p65	<20% cell death after 20 mM DMA in unstimulated/Aβ42 and 30 mM in LPS<br>↑IL-6, IL-8 and GM-CSF after LPS/Aβ42<br>↓IL-6, IL-8 and GM-CSF after LPS/Aβ42+DMA dose-dependent (0.1–10 mM)<br>↑Aβ42, APP and APP gene after LPS<br>↓Aβ42, APP and APP gene after LPS + DMA dose-dependent (0.1–10 mM)<br>↓IκBα after LPS 15min or Aβ42 1 h<br>↑IκBα after LPS/Aβ42+DMA	1 μg/mL LPS or 3 μM Aβ42 24 h. 0.1–100 mM of DMA 2 h
**[290]** Sangineto et al., 2023	Alzheimer's disease	LPS, Aβ 1-42, dimethyl malonate (DMM), TAK-242 (a specific inhibitor of TLR4), PX-478 (a HIF-1α specific inhibitor),<br>cell-permeable diethyl succinate	N/A	N/A	↑IL-1β, TNFα, glycolysis and mitochondrial respiration after LPS or Aβ1-42<br>↓IL-1β, TNFα after LPS + DMM/TAK-242<br>↓iNOS/Arg1 but ↑M2 phenotype after LPS + DMM<br>↓TLR4 tag, MyD88, glycolysis and mitochondrial respiration after LPS + TAK-242 or Aβ1-42+TAK-242 (lower efficacy)<br>↑Complex I, II, V, H2O2 from complex I-III, MDA and HNE after LPS<br>↑complex I and V after LPS + DMM<br>↓Complex II, H2O2 from complex I-III, MDA and HNE after LPS + DMM<br>↑Mitochondrial biogenesis (TFAM and PGC1-α) and HIF-1α after LPS<br>↓Mitochondrial biogenesis (TFAM and PGC1-α) and HIF-1α after LPS + DMM<br>↓IL-1β, TNFα after LPS + PX-478<br>↓Glycolytic capacity with no effect on basal glycolysis after LPS + PX-478<br>↑HIF-1α after succinate	LPS (1 μg/mL) and DMM (succinate dehydrogenase inhibitor, 10 mM) 24 h; Aβ 1-42 (10 ug/mL). glucose (3 g/L, 7 g/L, 10 g/L) and H2O2 (100 μM, 200 μM, 500 μM). PX-478 (10 μM). Pretreatment for 3 h with diethyl succinate (5 mM).<br>Second part focus on LPS every day for 10 days and treated with DMM only on the last day

(Continued)

10.3389/fimmu.2026.1778798

SUPPLEMENTARY TABLE 1 Continued

ARTICLE	MODEL	TREATMENT	DRUG TESTED	PATHWAY INVOLVED	MAIN RESULTS	NOTES
					↓Glycolytic capacity and maximal respiration after LPS 10days, with no changes after LPS + DMM	
**[291]** Mazrad et al., 2023	Neuroinflammation	LPS	brush-polymer, polymer-NAC conjugates	N/A	↑Accumulation of brush-polymers after LPS than in resting cells<br>↑IL-6 after LPS<br>↓IL-6 after LPS + polymer-NAC conjugates	N/A
**[292]** Campos et al., 2023	Viral infection (Mayaro (MAYV) and Una (UNAV) virus)	MAYV and UNAV	N/A	N/A	↓Viability but ↑cytopathic effect after MAYV or UNAV<br>↑E1 and nsP1 after MAYV or UNAV time-dependent<br>MAYV Guyane, TRVL 4675, UNAV 788382, CoAr 2518 strains replicate in HMC3<br>↑IFN-α, IFN- β, MDA5, OAS2, MxA, AIM2, TNFα, IL-6, IL-8, IRF-3, IRF-7, TLR3 and TLR7 genes after MAYV<br>↑MDA5, OAS2, MxA, IFN-α, IFN- β, TNFα, IL-6, IL-8, CCL5, TLR3, and TLR7 genes after UNAV (< MAYV)	N/A
**[293]** Scordino et al., 2023	Neuroinflammation	Tert-butyl hydroperoxide (TBH)	Grapefruit IntegroPectin (G)	PI3K/NF-κB/ iNOS, MAPK/ERK	↓viability and ↑smaller nuclei, fragmented and condensed after TBH 200 μM<br>↑viability after TBH+G<br>↑ROS after TBH<br>↓ROS after TBH+G<br>Annexin V- PI- (alive 36%) and Annexin V+ PI- cells (apoptotic 63%) after TBH<br>Annexin V- PI- cells (alive 95%) and (apoptotic 4.5%) of Annexin V+ PI- cells after TBH+G<br>↑Caspase-3, but p-ERK1/2after TBH<br>↓Caspase-3 after TBH+G<br>↓protective effect against ROS after G+PD98059/ LY29004 comparing to G alone<br>↓iNOS, p-Akt, p-NF-κB but ↑p-ERK1/2, with no changes in IL-6, IL-1β after G alone	IntegroPectin 1 mg/mL was used in all the subsequent experiments<br>for 24 h, 48 h and 72 h; TBH 200 μM 24 h.<br>PD98059, an inhibitor of MAPK, and LY294002 PI3K/Akt inhibitor (30<br>μM and 10 μM, respectively) were administered 1 h before IntegroPectin and TBH
**[294]** Qin et al., 2023	Neuroinflammation	LPS	Nicotine, GTS-21, MLA	α7 nAChR-PI3K	No effect on viability after LPS or nicotine<br>↓IL-6, MCP-1<br>(MCAF), RANTES, and VEGF after nicotine+LPS or GTS-21+LPS<br>↓IL-17 after nicotine+LPS<br>no changes in NO release after LPS<br>↓GDNF with no changes in BDNF after LPS<br>↑GDNF and BDNF after nicotine + LPS or MLA + LPS<br>↑α7 nAChR mRNA after nicotine or GTS-21	10 μg/mL LPS for 24 hours; (1, 10, 100 μM) of nicotine and 100 μM GTS-21 (dihydrochloride an α7 nAChR-specific agonist) or Methyllycaconitine citrate (MLA,an α7 nAChRspecific inhibitor) 1 h

(Continued)

10.3389/fimmu.2026.1778798

SUPPLEMENTARY TABLE 1 Continued

ARTICLE	MODEL	TREATMENT	DRUG TESTED	PATHWAY INVOLVED	MAIN RESULTS	NOTES
					↑pPI3K/PI3K after nicotine < LPS<br>↓pPI3K/PI3K after MLA or LPS	
**[295]** Dominici et al., 2023	Cancer (GBM)	co-culture with C15 GBM	N/A	N/A	↓Penetration of GFP T cells in C15 GMB GD2 allogenic<br>↑Penetration of GFP T cells in C15 GMB GD2 autologus<br>No changes in viability after co-culture with autologous anti-GD2 CAR T	3Dspheroid co-culture of the C15 GBM line with allogeneic or autologous CAR T cells with/without HMC3 cell line with a GD2 expression of 10.4%
**[296]** Privitera et al., 2023	Neuroinflammation	LPS + ATP	carnosine (Car)	N/A	↓Viability after LPS + ATP<br>↑Viability after LPS + ATP + Car<br>↑ROS, ADP, AMP, ATP, GTP, UTP, CTP but ↓ATP/ADP after LPS + ATP<br>↓ROS, ADP, AMP, ATP, GTP, UTP, CTP but ↑ATP/ADP after LPS + ATP + Car<br>↓NAD+, NADHP, NAD+/NADH after LPS + ATP<br>↑NADH, NADP+/NADPH after LPS + ATP<br>↑NAD+, NADHP, NAD+/NADH after LPS + ATP + Car<br>↑↓NADH, NADP+/NADPH after LPS + ATP + Car<br>↓GSH but ↑nitrite, nitrate after LPS + ATP<br>↑GSH but ↓nitrite, nitrate after LPS + ATP + Car<br>↓UDP-Gal, UDP-Glc, UDP-GalNac, and UDP-GlcNac afte LPS + ATP<br>↑UDP-Glc, UDP-GalNac, and UDP-GlcNac afte LPS + ATP + Car	LPS (100 ng/mL, 24 h) + ATP (5 mM, 30 min), in the absence or presence of Car (10 mM, 1 h pre-treatment).
**[297]** Zou et al., 2023	Neuroinflammation	CM	N/A	N/A	↓IL-6, IL-17A after AC medium<br>↑Immune-related gene expression e.g. cAMP and TGF-β signaling pathways	CM from Caco-2 cells treated with A. muciniphila metabolites (AC medium) medium, to treat HMC3 cells to simulate the gut–brain axis
**[298]** Hussain et al., 2023	Neuroinflammation	LPS, mtOGG1	N/A	pSTING	↑mtDNA in the cytoplasm after LPS<br>↓pSTING and mitochondrial functions after LPS<br>↓mtDNA in the cytoplasm after LPS + mtOGG1<br>↑pSTING and mitochondrial functions after LPS + mtOGG1	*In vivo:* experiment conducted on transgenic mtOGG1Tg mice
**[299]** Hacioglu et al., 2023	Cancer (GBM)	Borax	N/A	N/A	↓Viability, proliferation after borax time-/dose-dependent<br>IC50 = 1.72mM<br>↑G0/G1 phase after borax<br>↓S phase after borax<br>↓Sensitivity for borax than U87-MG	Borax (0–1.6 mM) for 24/48/72 h

(Continued)

10.3389/fimmu.2026.1778798

SUPPLEMENTARY TABLE 1 Continued

ARTICLE	MODEL	TREATMENT	DRUG TESTED	PATHWAY INVOLVED	MAIN RESULTS	NOTES
**[300]** Hacioglu et al., 2023	Cancer (GBM)	Temozolomide (TMZ) and boric acid	N/A	N/A	↓Viability and proliferation after TMZ >1mM<br>↓Viability after boric acid 1mM<br>↓Proliferation after boric acid 2mM	TMZ concentrations ranging from 0 to 2 mM for 72 h, boric acid 250 µM, 500 µM, 1 mM, and 2 mM
**[301]** Fu et al., 2023	Neuroinflammation	IL-1β,. EGFR silencing (KO) or overexpression (OV)	formononetin (FMN)	EGFR/ p38MAPK	No effect on viability after FMN<br>↓TNFα and IL-6 after IL-1β+FMN dose-dependent<br>↓p-EGFR, p-p38, TNFα and IL-6 after IL-1β+FMN/EGFR-KO<br>↑p-EGFR, p-p38, TNFα and IL-6 after IL-1β+FMN+EGFR-OV	N/A
**[302]** Xu et al., 2023	Neuroinflammation (Lyme neuroborreliosis)	Recombinant BmpA (rBmpA), LPS	pure Cucurbitacin IIa, CuIIa (98%)	N/A	↓viability after CuIIa 100 µM<br>↑24 inflammation-related cytokines after rBmpA<br>↑IL-6, IL-8, CCL2, CCL5, CXCL1, and CXCL10 mRNA after rBmpA or LPS<br>↓IL-6, IL-8, CCL2, CCL5, CXCL1, and CXCL10 mRNA after rBmpA+CuIIa<br>↑IL-6, IL-8, and CXCL10 in the supernatant after rBmpA	CuIIa 50 µM,<br>LPS-stimulated group as a positive control group,
**[303]** Wu et al., 2023	DR	Glucose, co-culture HUVEC	Aflibercept, Conbercept, Ranibizumab	N/A	No effect on viability after G5.5, G10 and G25 in HMC3 alone<br>↓Viability dose-dependent in co-cultured after 3 day<br>↑IBA1, activation of microglia in co-culture after G10 and G25<br>↑Tube formation in co-culture G5.5 1 day<br>↓Tube formation and mesh area G10 and G25<br>↑Amoeboid morphology after G25<br>↑1038 genes but ↓574 genes in co-cultured comaring to HMC3 alone<br>↑IL1RL1, PTGS2, NOS3, NLGN1, GIPC2, FAM156A, TLN2, ACVR2A and DNAJB14 in co-cultured after G25<br>↑THBS1-AS1, EPHA1, SPON1, POSTN, ALOX12, PTGS2, CYP1B1, IGFBP3 and NR4A1 in co-cultured after G10<br>↓ISCA1P1 and COX7B in co-culture after G25<br>No changes in viability after Aflibercept, Conbercept or Ranibizumab in HMC3 alone<br>↓Viability after Aflibercept or Conbercept in co-culture at G10 and G25	Medium with<br>5.5 mM glucose (G5.5), and medium with additional glucose of 10 mM (G10), 25 mM (G25). Bioprinted HMC3-RFP cells within the hydrogel and immediately seeded HUVEC-zsGreen cells on top of the bioprinted hydrogel.<br>Aflibercept, Conbercept or Ranibizumab at 10 µg
**[304]** Mishra et al., 2023	Neuroinflammation	Nitrates (1X or 5X), oligomycin (ATP synthase inhibitor)	N/A	N/A	↑Morphology changes after 5X<br>↓Viability after 5X but not 1X<br>↑miR-145 and miR-143 after 1X or 5X	Cells were exposed to nitrates as a mixture of sodium nitrate, potassium nitrate, and magnesium nitrate (320 mg/L nitrates and 1600 mg/L nitrates)

(Continued)

10.3389/fimmu.2026.1778798

SUPPLEMENTARY TABLE 1 Continued

ARTICLE	MODEL	TREATMENT	DRUG TESTED	PATHWAY INVOLVED	MAIN RESULTS	NOTES
					↓miR-199a-3p, miR-213, miR-210, miR-34a, miR-9, and miR-339-5p after 5X<br>Dysregulation of 163 proteins in 1X (connected with endocytosis, virus infection, proteolysis, pyrimidine metabolism)<br>Dysregulation 277 proteins in 5X (multiple disease: ALS, PD, AD, prion disease, HD)<br>SLU7 target of miR-143<br>↓ Oxygen consumption rate (OCR), a sign of dysfunctional oxidative phosphorylation (OXPHOS). OXPHOS and respiratory capacity after 5X<br>no effect on ATP after 1X or 5X	for 72 h.<br>Oligomycin (1 μM)
**[305]**<br>Kocanci et al., 2023	Neuroinflammation	LPS, SH-SY5Y co-culture	pimecrolimus	N/A	↓Viability after LPS 10 μg/ml or LPS + pimecrolimus >10 μM<br>↑NO, IL-1β, IL-6, IL-10, activation after LPS<br>↑Viability after pimecrolimus<br>no effect on viability after LPS + pimecrolimus<br>↓IL-1β, IL-6 in mono-/co-culture after LPS + pimecrolimus<br>↑IL-1β, IL-6 in co-culture after LPS<br>↓IL-10 in co-culture after LPS<br>↺IL-10 in co-culture after LPS + pimecrolimus<br>TAC in co-culture after LPS + pimecrolimus	LPS (0.1– 10 μg/mL) 24 h, pimecrolimus 0.001– 10 μM 24 h.
**[306]**<br>Kocanci et al., 2023	Neuroinflammation	LPS	tacrolimus (FK506)	N/A	↓Activation and ↺morphology after LPS + FK506<br>↑Total antioxidant capacity but ↓total oxidative capacity after LPS + FK506<br>↓IL-1β and IL-6 after LPS + FK506	1 μg/mL LPS in the presence or absence of doses of FK506
**[307]**<br>Ni et al., 2023	Cancer	Lentivirus MS4A7-l OE and MS4A7-s OE, co-cultured with U87MG	N/A	PI3K/AKT/ GSK3β	↓Viability, proliferation and phagocytosis after MS4A7-1 OE, but not MS4A7-s OE<br>↑CD163, M2 phenotype in MS4A7-s OE<br>↓IL-1β and CD11b MS4A7-s OE<br>Differences in PC1 (85.9%) and PC2 (6.8%) after MS4A7-l OE<br>↑PI3K–Akt signaling pathway, angiogenesis and TGF-β pathway after MS4A7-s<br>↑MAPK pathway and cell migration after MS4A7-l OE<br>↑DMKN and MYD88 after MS4A7-s OE	*In vivo:* glioma-associated macrophages' (GAMs). U87MG cells were co-injected with control, MS4A7-l OE or MS4A7-s OE HMC3 cells into the brains of BALB/c immunodeficient (SCID) mice
**[308]**<br>Mason et al., 2023	Alzheimer's disease	LPS, pHrodo-myelin/membrane debris	cytochalasin D, idelalisib, saracatinib	N/A	↑Phagocytosis dose-dependent after pHydro-myelin or Saracatinib<br>↓Phagocytosis after pHydro-myelin+Cytochalasin D or Idelalisib	5 μg/ml pHrodo-myelin/membrane debris<br>up to 10 μg/ml LPS

(Continued)

10.3389/fimmu.2026.1778798

SUPPLEMENTARY TABLE 1 Continued

ARTICLE	MODEL	TREATMENT	DRUG TESTED	PATHWAY INVOLVED	MAIN RESULTS	NOTES
					No effect on viability and nuclear intensity after pHydro-myelin+Cytochalasin D or Idelalisib<br>No response to LPS	
**[309]**<br>Xu et al., 2023	Cancer	EV-derived LINC00482	N/A	LINC00482/ miR-142-3p/ TGF-β1	↑M2 polarization, miR-142-3p and TGF-β1 after LINC00482<br>↑Malignant properties of NSCLC after LINC00482<br>LINC00482 competitively bound to miR-142-3p	LINC00482 derive from NSCLC patients (serum-EVs).<br>*In vivo:* experiment conducted on xenograft mouse models of NSCLC.
**[310]**<br>Yang et al., 2023	Cancer (GBM)	N/A	N/A	N/A	↓KIF18A in HMC3 comparing to GBM cells<br>↓PPP1CA in HMC3 comparing to A172 cells	HMC3 used as the control group
**[311]**<br>Sun et al., 2023	Cancer	LPS	ergosterol	NF-κB,/AKT/ MAPK	No effect on viability after Ergosterol (5-50 µM)<br>↑IL-1β and TNFα, but not IL-18 after LPS<br>↓IL-1β, TNFα, p-JNK, p-P38, p-65 and p-AKT after LPS + Ergosterol<br>↓COX-2 and INOS after LPS + Ergosterol	LPS 1 µg/mL and Ergosterol (0, 5, 10, 30, 50, 100 µM) for 24 h
**[312]**<br>Yang et al., 2023	Neuroinflammation (Ischemic stroke)	pTMEM119-Netrin-1, UNC5a-Fc	N/A	N/A	↑Netrin-1 mRNA and protein after pTMEM119-Netrin-1<br>↑CD163, IL-10, ARG1, M2 phenotype and viability after pTMEM119-Netrin-1<br>↓IL-1β, IL-6, IFN, TNFα, invasion and migration after pTMEM119-Netrin-1<br>↓M2 polarization and Netrin-1 after UNC5a-Fc	TMEM119 promotes the Netrin-1 transgene or a scramble sequence
**[313]**<br>Sun et al., 2023	Alzheimer's disease	Aβ25–35,<br>co-cultured with SH-SY5Y	soluble epoxide hydrolase inhibitor (TPPU)	TLR4/MyD88/ NF-κB, MAPK/NF-κB	↑cell viability after TPPU (0.1, 1, 10 µM) 48 h<br>↑microglobular phagocytosis, ROS, MDA but ↓SOD after Aβ(25–35)<br>↓microglobular phagocytosis, ROS, apoptosis but ↑SOD after Aβ(25–35)+TPPU<br>↑TNF, IL-1β and EPHX2 after Aβ(25–35)<br>↓TNF, IL-1β, IL-6, IL-18 and EPHX2 after Aβ(25–35)<br>↑CD206, SOCS3 and EPHX2 after Aβ(25–35) +TPPU<br>↓11,12-EET and 14,15-EET after Aβ(25–35)<br>↑11,12-EET and 14,15-EET after Aβ(25–35) +TPPU<br>↑TLR4, EPHX2, MyD88 and P-p38 MAPK after Aβ(25–35)<br>↓TLR4, EPHX2, MyD88 and P-p38 MAPK after Aβ(25–35)+TPPU	cells were pretreated with 1 µM TPPU for 3 h and then incubated with 30 µM Aβ25–35 for 48 h.<br>Aβ25–35 (5 µM, 10 µM, 15 µM, 20 µM, 25 µM, 30 µM, or 35 µM) for 48 h and then measured HMC3 cell viability
**[314]**<br>Li et al., 2023	Neuroinflammation	LPS	trans-ferulic acid (TJZ-1) and	NF-κB	↓viability after TJZ-1/TJZ-2 (10 µM)<br>↑NO, TNFα, IL-1β, IKK, p65, and β-EP after LPS<br>↓NO, TNFα, IL-1β, p65 and β-EP after LPS +	10 µM TJZ-1 and TJZ-2 for 24 h. LPS (1 µg/mL), Celecoxib (10 µM) was used as a positive drug.

(Continued)

10.3389/fimmu.2026.1778798

SUPPLEMENTARY TABLE 1 Continued

ARTICLE	MODEL	TREATMENT	DRUG TESTED	PATHWAY INVOLVED	MAIN RESULTS	NOTES
			methyl ferulate (TJZ-2)		TJZ-1/TJZ-2<br>↓IKK after LPS + TJZ-1<br>No effect on IκBα after LPS + TJZ-1/TJZ-2	
**[315]** Sharma et al., 2023	Neuroinflammation	LPS	silver nanoparticles (AgNPs)	TLR4/MyD88, Nrf2/HO-1	↑TNFα, IL-6 but ↓TGF-β, IL-10 after LPS<br>↓TNFα, IL-6 but ↑TGF-β, IL-10 after LPS + AgNPs<br>↓M1 markers (CD80, CD86, CD68) but ↑M2 markers (CD206, CD163) and TREM2 after LPS + AgNPs<br>↓TLR4, My88 ROS and nitric oxide (NO) synthetase after LPS + AgNPs<br>↑Nrf2, HO-1 after LPS + AgNPs	Honeyberry extract was used to synthesize and characterize biogenic AgNPs.
**[316]** Pandey et al., 2023	Viral infection (Chandipura virus, CHPV)	Anti-miR-155, miR-155 mimics, CHPV, IFN- β	N/A	N/A	↑miR-155, pSTAT1, p-STAT1/STAT1, IFN-β, ISG54, ISG56 after CHPV<br>↓SOCS1 after miR-155 mimics<br>↑IFN-β after miR-155 mimics<br>↑SOCS1 after anti-miR-155 ± CHPV<br>↓IFN-β, p-STAT1/STAT1, ISG54, ISG56 after anti-miR-155 ± CHPV<br>↑p-STAT1/STAT1, ISG-54 after miR-155 mimics ± CHPV or IFN-β (90/135 min)<br>↓CHPV P gene after miR-155 mimics+CHPV<br>↑CHPV P gene after anti-miR-155+CHPV	0.1 μg/mL of IFN-β for 45, 90, and 135 min. CHPV MOI=0.1
**[317]** Kang et al., 2023	Neuroinflammation (Ischemic stroke)	OGD/R<br>sh-circPTP4A2, miR-20b-5p mimic/inhibitor, sh-YTHDF1, Oe-TIMP2	actinomycin D	miR-20b-5p/ YTHDF1/ TIMP2	↑circPTP4A2 after OGD/R<br>↓circPTP4A2 after sh-circPTP4A2 or sh-circPTP4A2+OGD/R<br>↓viability and miR-20b-5p after OGD/R<br>↑viability and miR-20b-5p after OGD/R + sh-circPTP4A2<br>↑M1 markers (iNOS, CD16) and M2 markers (Arg1, CD206) after OGD/R<br>↓M1 markers (iNOS, CD16) but M2 markers (Arg1, CD206) after OGD/R+sh- circPTP4A2<br>↑TIMP2, p-p65, and p-IκBα after OGD/R<br>↓TIMP2, p-p65, and p-IκBα after OGD/R + sh-circPTP4A2<br>↓circPTP4A2, YTHDF1 but ↑miR-20b-5p after OGD/R + sh-circPTP4A2<br>↓miR-20b-5p expression and ↑YTHDF1 after sh-circPTP4A2 + miR-20b-5p-inhibitor<br>↓YTHDF1 and TIMP2 after sh-circPTP4A2 or sh-YTHDF1<br>↑TIMP2 mRNA degradation after actinomycin D + sh-circPTP4A2/sh-YTHDF1	OGD/R cells were cultured in DMEM with 2% O2, 93% N2, and 5% CO2 for 2 h, followed by reoxygenation for 22 h.

(Continued)

10.3389/fimmu.2026.1778798

ARTICLE	MODEL	TREATMENT	DRUG TESTED	PATHWAY INVOLVED	MAIN RESULTS	NOTES
					↓NF-κB pathway after sh-circPTP4A2 or sh-YTHDF1<br>↓Arg1, CD206 but ↑iNOS, CD16 after OGD/R + miR-20b-5p-inhibitor + sh-circPTP4A2<br>↑TIMP2, p-p65, and p-IκBα after OGD/R + miR-20b-5p-inhibitor<br>↓TIMP2, p-p65, and p-IκBα after OGD/R + miR-20b-5p-knockdown<br>↑miR-20b-5p but ↓TIMP2 after OGD/R + miR-20b-5p-mimic<br>↑miR-20b-5p and TIMP2 after OGD/R + miR-20b-5p-mimic + OE-TIMP2<br>↓iNOS, CD16 but ↑Arg1, CD206 after OGD/R + miR-20b-5p-mimic<br>↓iNOS, CD16, NF-κB but ↑Arg1, CD206 after OGD/R + miR-20b-5p-mimic + OE-TIMP2	
**[318]** Caruso et al., 2023	Neuroinflammation	N/A	Car	N/A	↓Viability after Car >20mM<br>No effect on IL-1β, TGF-β1, TGFβ-R2 mRNA after Car<br>↑IL-6 after Car dose-dependent<br>↓iNOS nor Nox-2 mRNA after Car dose-dependent<br>No effect on Nrf2 mRNA, Keap-1 mRNA, GSH, ATP, AMP, GTP, NAD, NAD+, NADH, UDP-derivatives and $O_2^{-\bullet}$after Car<br>↑HO-1 mRNA after Car >10mM<br>↓NO, ADP but ↑ATP/ADP, NADP and NADP+ after Car 10mM	Car, 1, 10, and 20 mM) for 24 h
**[319]** Mitra et al., 2023	Alzheimer's disease	Aβ40, Aβ42, IFN-γ, IL-1β,	N/A	N/A	↓NGF, proliferation, viability of NGC0211 after MCM<br>↑Activation of HMC3 after Aβ40-42/IFN-γ/IL-1β	CM from HMC3 1μM Aβ40-42, 10 ng/mL IFN-γ, IL-1β; 24 h. Encapsulated cell biodelivery on NGC0211 cells
**[320]** Liang et al., 2023	Cancer	co-culture with T98G/si-ENO1-NC, T98G/si-ENO1-1, and T98G/si-ENO1-2	N/A	N/A	High-GS neoplastic cells targeted microglia in CSF1/CSF1R, POSTIN/(ITGAV and ITGB5), PROS1/AXL, and VEGFA/VEGFR1 mediated signaling pathways<br>↓ENO1, CD163 after si-ENO1-1, si-ENO1-2<br>↑ENO1, CD163 after ENO1-OE	N/A
**[321]** Unbehau et al., 2023	Metabolism	mitomycin C (MMC)<br>chromic acid (CA),<br>Mg-seeding	N/A	N/A	↑Mg degradation but ↓ΔpH density-dependent<br>↓viability after MMC<br>↑lactate and DNA contents after MMC<br>↓collagen and elastin after MCC<br>↓GAG/cell ratios density-dependent	N/A

(Continued)

10.3389/fimmu.2026.1778798

SUPPLEMENTARY TABLE 1 Continued

ARTICLE	MODEL	TREATMENT	DRUG TESTED	PATHWAY INVOLVED	MAIN RESULTS	NOTES
**[322]** Sawkulycz et al., 2023	Neuroinflammation (Ischemic stroke)	hypoxia, TNFα and platelet releasate	N/A	N/A	↑IL-1B, IL-6 after hypoxia+TNFa+platelet releasate<br>↑mediators of thrombosis (vWF and CD39) after hypoxia+TNFa+platelet releasate<br>↑LXRα, LXRβ, Pim-1, Pim-2, Pim-3 and SIRT1, P2Y2 after hypoxia+TNFa+platelet releasate	
**[323]** Merlo et al., 2023	Neuroinflammation	LPS	melatonin (MEL)	N/A	↑NF-κB+ round, ameboid cells after LPS<br>↑BDNF+ round, ameboid cells after MEL or MEL +LPS<br>↓NF-κB after MEL+LPS<br>↓CASP1 but ↑SIRT1 after MEL+LPS<br>No effect on HMC3 after MEL alone	LPS (1 μg/mL) 24 h or 48 h, MEL (1 μM) was added immediately after plating
**[324]** Moutinho et al., 2023	Alzheimer's disease	TREM2-230, TREM2-222, TREM2-219	N/A	N/A	↑TREM2-230/222/219 in HMC3 under permeabilized cond.<br>↑TREM2-230 in HMC3 under non-permeabilizing cond.<br>↑purified soluble TREM2-222/219 in HMC3 lysates	Transfection of TREM2 isoforms (230, 222, 219) 10 ng/ml to HMC3 under permeabilizing and non-permeabilizing conditions
**[325]** Chiu et al., 2023	Parkinson's disease	MPP +	NC009	N/A	Viability >80% after NC009<br>↓Viability after MPP+ dose-dependent<br>↑Viability after NC009-1+MPP+<br>↑NO, NLRP3, CASP1, iNOS, IL-1β, IL-6, and TNFα after 2.5–10 mM MPP+<br>↓NO, CD68, CD11B, IL-1β, IL-6, TNFα and NLRP3 after NC009+MPP+	MPP + 3 mM 20 h (neurotoxic metabolite of MPTP); pre-treatment with NC009 1-10 μM 8 h
**[326]** Hu et al., 2023	Cancer	CYP2E1 plasmids, Q11	N/A	PPAR-γ-STAT-1/NF-κB/ STAT-3/STAT-6	↑proliferation, migration after CYP2E1<br>↓proliferation, migration after CYP2E1+Q11<br>↑PPAR-γ, accompanied by increased IL-4, IL-10, and TGFβ after CYP2E1<br>↓PPAR-γ, accompanied by increased IL-4, IL-10, and TGFβ after CYP2E1+Q11<br>↓IL-1α and IL-1β after CYP2E1<br>↑IL-1α and IL-1β after CYP2E1+Q11	Q11 (3,1, 12.5, 50 μM) 24 h is the inhibitr of tumor-promoting effects.
**[327]** Imbesi et al., 2023	Neuroinflammation	TNFα	KJ3 and KJ9 (Novel amide derivatives)	ERK1/NF-κB	↓viability after TNFα >10ng/ml, KJ3 <10 μM, KJ9 >20 μM<br>↑p-NF-κB, p-IkB-α, ROS, cytoplasmic ubiquitin, p-ERK, IL-1β and IL-6 RNA after TNFα<br>↓p-NF-κB, p-IkB-α, ROS, cytoplasmic ubiquitin, p-ERK, IL-1β and IL-6 RNA after TNFα+KJ3/KJ9	TNFα (10 ng/mL); KJ3 (1 μM) and KJ9 (1 μM) for 24 h.
**[328]** Polini et al., 2023	Neuroinflammation	LPS/TNFα, Aβ25-35, RO5166017, EPPTB, TA1	3-Iodothyronamine (T1AM)	T1AM/TAAR1	No effect on viability after T1AM<br>↑Cytotoxicity after Aβ25-35 is dose-dependent<br>↓Cytotoxicity after Aβ25-35 + T1AM<br>↑TA1 after T1AM	Pre-treatment 0.1, 1, and 10 μM) of T1AM for 1 h, TAAR1 selective antagonist 5 nM (EPPTB) and a selective agonist 1 μM (RO5166017). TA1 (0.1, 1, and 10 μM)

(Continued)

10.3389/fimmu.2026.1778798

SUPPLEMENTARY TABLE 1 Continued

ARTICLE	MODEL	TREATMENT	DRUG TESTED	PATHWAY INVOLVED	MAIN RESULTS	NOTES
					↑IL-6, TNFα, NF-κB, MCP1, MIP1 but ↓IL-10 after LPS/TNFα or LPS/TNFα + T1AM + EPPTB<br>↓IL-6, TNFα, NF-κB, MCP1, MIP1but ↑IL-10 after LPS/TNFα + T1AM or LPS/TNFα + RO5166017<br>No effect on IL-6 and IL-10 after TA1<br>↑IL-6, TNFα, p-P65 after Aβ25-35<br>↓IL-6, TNFα, p-P65 after Aβ25-35 + T1AM	
**[329]** Solan et al., 2023	Neuroinflammation	PFBS, PFHxA, HFPO-DA, 6:2 FTOH, PFHxS	N/A	N/A	↑GPX after PFBS 1 µM<br>↑Activity of antioxidant enzyme after PFBS, PFHxA, HFPO-DA, 6:2 FTOH	Short-chain PFAS compounds: PFBS, PFHxA, HFPO-DA, 6:2 FTOH, PFHxS) at low (1 nM) and high (1 µM) concentrations
**[330]** Lin et al., 2023	Neuroinflammation	α-Syn, IL6ST-AS UP, IL6ST-AS KO, h-IL6ST-3UTR and has-miR-488-3R mimics, h-CTD-2031P19.5-3UTR	N/A	IL6ST-AS/ STAT3/HIF-1α	↑lncRNA IL6ST-AS in cytoplasm and IL6ST mRNA after α-Syn<br>↓IL6ST after α-Syn or IL6ST-AS UP<br>↑IL6ST after IL6ST-AS KD<br>↓IL6ST-AS and IL6ST after has-miR-488-3R+h-IL6ST-3UTR or has-miR-488-3R+ h-CTD-2031P19.5-3UTR<br>↓JAK2-STAT3, HIF-1α and NF-κB after IL6ST-AS UP + α-Syn<br>↑p-JAK2, p-STAT3, HIF-1α and NF-κB after IL6ST-AS KD + α-Syn	25 µmol/L α-Syn
**[331]** Raber et al., 2023	Neuroinflammation	*A. muscaria* (AME-1), trehalose	N/A	N/A	↑CD86, CXCR4, CD125, TLR4 after AME-1 at higher concentrations<br>↑IL-8 poly(I:C)-mediated after AME-1 or trehalose	AME-1 contains the autophagy inducer trehalose
**[332]** Liu et al., 2023	Diabetes	LPS/HG/PA; BMS, 5′-cholesteroi SiRNA-Mfn1, si-Mfn1-KO, FABP4-OE	genipin (GEN)	FABP4/NF-κB	↓TNFα, IL-6, iNOS mRNA, CCL2 mRNA and CD68 after GEN+LPS/HG/PA<br>↑IL-4, IL-10, ARG1 mRNA, YM1 mRNA and CD206 after GEN+LPS/HG/PA<br>↓nucleus translocation of NF-κB and lipid accumulation, Mfn1 but no effect on IL-6, TNFα, Mfn1 mRNA after GEN alone<br>No effect on ACOX1, ACAA2, ECHS1, FASN and ACLY after GEN or LPS/HG/PA<br>↑SLC27A1, FABP4 and FABP7 after LPS/HG/PA<br>↓FABP4 and p-NF-κB after GEN+LPS/HG/PA<br>↓MMP after LPS/HG/PA<br>↑Mfn1, Mfn2, p-S637-Drp1, MMP after GEN +LPS/HG/PA<br>↑MDA, ROS but ↓SOD, GSH-Px after LPS/HG/PA<br>↓MDA, ROS, but ↑SOD, GSH-Px after GEN+LPS/ HG/PA<br>↓translocation of p47phox and p67phox into the cell membrane, NADP+/NADPH, NOX after GEN +LPS/HG/PA	After the transfection cells were treated with GEN (5 µM, 10 µM, 20 µM) or FABP4 selective inhibitor BMS (40 µM) for 4 h and then stimulated with LPS (1 µg/mL), high glucose (HG, 33 mM) and PA (100 µM) for another 12 h.

(Continued)

10.3389/fimmu.2026.1778798

SUPPLEMENTARY TABLE 1 Continued

ARTICLE	MODEL	TREATMENT	DRUG TESTED	PATHWAY INVOLVED	MAIN RESULTS	NOTES
					↓TNFα, IL-6, FABP4, p-NF-κB, ROS but ↑Mfn1 after BMS<br>↓TNFα, IL-6, p-NF-κB, bodipy intensity after si-Mfn1<br>↑TNFα, IL-6 after GEN+si-Mfn1-KO or FABP4-OE<br>↓Mfn1 after GEN+LPS/HG/PA+si-Mfn1-KO<br>↑IL-6 after BMS+si-Mfn1-KO<br>↑TREM2, MSR1 and phagocytosis after GEN+LPS/HG/PA<br>↓Hrd1 after GEN+BMS<br>↓Ubiquitination of Mfn1after GEN+LPS/HG/PA	
**[333]** Dandan et al., 2023	Neuroinflammation	LPS; si-IL-33	N/A	IL-33/ST2	↑IL-33, ST2, IL-6, IL-8 after LPS dose-dependent<br>↓IL-33, ST2, IL-6, IL-8, NF-κB after LPS + si-IL-33	LPS (0, 100 ng/ml and 500 ng/ml) for 24 h
**[334]** Baek et al., 2023	Neuroinflammation	LPS, JQ1 (an inhibitor of the bromodomain and extra-terminal (BET) protein BRD4)	N/A	N/A	5 DElncRNAs (all upregulated) and 99 DEmRNAs (98 upregulated and 1 downregulated) after LPS<br>↑AC083837.1 and LINC02605 after LPS + JQ1<br>↓MIR3142 hG, MIR155HG, IRF1-AS1 after LPS + JQ1<br>↓22 mRNAs, 2 lncRNAs but ↑15 mRNAs, 1 lncRNA after LPS + JQ1	100 ng/ml LPS and/or 500 nM JQ1 for 4 h.
**[335]** Paškan et al., 2023	Neurotoxicity	4-Isobutylmethcathinone	N/A	N/A	↑Cytotoxicity (IC50 = 61 μM)	N/A
**[336]** Wang et al., 2023	Transcriptome (esosomes/ nanoparticles)	ICH-exos, pcDNA3.1-SND1-IT1	N/A	lncRNA SND1-IT1/miR-124-3p/MTF1	↑lncRNA SND1-IT1 after ICH-exos<br>↓viability and ↑apoptosis via miR-124-3p/MTF1 after pcDNA3.1-SND1-IT1+ICH-exos<br>162 DE-lncRNAs affected by pcDNA3.1-SND1-IT1 +ICH-exos	N/A
**[337]** De Chirico et al., 2023	Parkinson's disease	HG, IFN-γ; OHDA, MPTP	N/A	p38MAPK/NF-κB	↑iNOS, Caspase 1, IL-1β after IFN-γ + HG<br>↑TREM2, IBA1, heterogenicity (globular and elongated, ameboid morphology), dynamic polarization after IFN-γ + HG<br>↑NF-κB(p65)/NF-κB, iNOS, IBA1 after 6-OHDA or MPTP	HG 5 g/l w/or w/o IFN-γ (1 μg/ml). 6-OHDA 1 and 10 μM, or 1-methyl-4-phenyl-1,2,3,6-tetrahydropyridine (MPTP) 0,01, 0,1 and 1 μM, in serum free medium for 24 h.
**[338]** Muñoz et al., 2023	Alzheimer's disease	chol + AβO + fructose + LPS	N/A	N/A	↑Effluxed cholesteryl esters in HDL after chol + AβO + HDL fasted (10-fold more than after chol + AβO + fructose)<br>↓ApoE after chol + AβO<br>↑ApoE after chol + AβO + HDL fasted<br>↑Activation, ameboid phenotype after chol + AβO	cholesterol (chol), AβO, lipopolysaccharide (LPS), and fructose individually and in combination. HDL from 36 h fasted or postprandial individuals.

(Continued)

10.3389/fimmu.2026.1778798

SUPPLEMENTARY TABLE 1 Continued

ARTICLE	MODEL	TREATMENT	DRUG TESTED	PATHWAY INVOLVED	MAIN RESULTS	NOTES
					+ fructose + LPS<br>↑IL-6 after chol + LPS<br>↓IL-1β after chol + AβO<br>↑Cellular chol and cholesteryl esters (CE) after chol + fructose or fructose alone or chol + AβO + LPS or AβO + fructose or chol + AβO + fructose + LPS<br>↑Mitochondrial chol after chol + AβO + fructose + LPS<br>↓ApoE after chol + AβO or chol + AβO + fructose + LPS (strongest effect)<br>↑APOE, TNFα, ROS but ↓ATP, phagocytosis after chol + AβO + fructose + LPS<br>↓Phagocytosis after AβO alone or AβO + fructose or LPS alone or LPS + fructose or chol + LPS + fructose	
**[339]** Zhang et al., 2023	Neuroinflammation (Ischemic stroke)	HBMECs exosome, OGD, miR-3613-3p-inhibitor, si-RC3H1	N/A	N/A	↑miR-3613-3p after OGD+BMECs<br>↓miR-3613-3p after OGD+BMECs+miR-3613-3p-inhibitor<br>↑IL-1β, iNOS, IL-6 but ↓IL-10 in OGD+BMECs 24 h<br>↓IL-1β, iNOS, IL-6 but ↑IL-10 in BMECs or OGD +BMECs+miR-3613-3p-inhibitor or OGD-BMECs + miR-3613-3p-inhibitor+si-RC3H1 group<br>↓RC3H1 after OGDD+BMECs or OGD-BMECs + miR-3613-3p-inhibitor+si-RC3H1 group<br>↑RC3H1 after OGD+BMECs + miR-3613-3p-inhibitor<br>↑CD86 but ↓CD206 after OGD+BMECs<br>↓CD86 but ↑CD206 after OGD+BMECs+miR-3613-3p-inhibitor	Exosomes from normal and OGD-cultured HBMECs were collected. 200 μg of exosomes were added to 50 μl of the PKH67 working solution for 10 min.
**[340]** Inoue et al., 2023	Neuroinflammation	Pg LPS, E64d, CA-074Me, pepstatin A	β-defensin 3 (hBD3)	N/A	↑IL-6 after Pg-LPS<br>↓IL-6 after Pg-LPS + hBD3/E64d/CA-074Me<br>No effect on IL-6 after hBD1, hBD2, hBD4 or pepstatin A	Porphyromonas gingivalis (Pg) LPS 30 μg/mL for 3 h. hBD3 (1 μM), E64d, a cysteine protease inhibitor, CA-074Me, a specific inhibitor for cathepsin B, pepstatin A, an aspartic protease inhibitor
**[341]** Wagner et al., 2023	Neuroinflammation	γ-amino butyric acid (GABA), muscimol, AME-1	bicuculline	N/A	CHRNB2, CHRNB3, CHRNB4, ABAT, ACHE, BSN, GAD1, GCH1, GABRA2, GABRA4, GABRB1, IL-8 and ROS occur in HMC3<br>Absence of CHRNA2, DRD3, GABAA1, GABRA1, GABRA6, GABRG1, GABRG2, GABRG3, GABRR1, GLRA2, HTR1E, HTR2A, HTR3A, HTR3B, SYN3, SLC32A1, SLC5A7, SLC6A11, SLC6A18, SLC6A2, SLC6A20, SLC6A5, SLC6A7,	15 μg/mL GABA, 15 μg/mL muscimol, 500 μg/mL Amanita muscaria extract (AME-1) contained GABAR agonists: GABA and muscimol, 24 h

(Continued)

10.3389/fimmu.2026.1778798

SUPPLEMENTARY TABLE 1 Continued

ARTICLE	MODEL	TREATMENT	DRUG TESTED	PATHWAY INVOLVED	MAIN RESULTS	NOTES
					and SLC6A in HMC3<br>GABAAR possess pentameric structure of α2, α4, α5, and β3 subunits<br>↑IL-8, Iba1, round morphology after muscimol/GABA/AME-1<br>no effect on ROS after muscimol/GABA/AME-1<br>↑metabolic activity after GADBA<br>↓IL-8 was after GABA+bicuculline<br>↑IL-8 after AME-1+bicuculline	
**[342]**<br>Zhang et al., 2023	Neuroinflammation (Ischemic stroke)	OGD/R, pcDNA3.1-OIP5-AS1 (OIP5-AS1 OE), shOIP5-AS1, miR-155-5p inhibitor/mimics, pcDNA3.1-IRF2BP2 (IRF2BP2 OE), si-IRF2BP2	N/A	miR-155-5p/ IRF2BP2	↓OIP5-AS1, IRF2BP2, SOD after OGD/R<br>↑miR-155-5p, TNFα, IL-1β, IL-6, ROS, MDA after OGD/R<br>↑OIP5-AS1, SOD but ↓miR-155-5p, TNFα, IL-1β, IL-6, ROS, MDA after OGD/R+OIP5-AS1 OE<br>↑miR-155-5p after shOIP5-AS1 or miR-155-5p mimics<br>↓miR-155-5p, ROS, MDA but ↑SOD after OGD/R +miR-155-5p inhibitor<br>while OIP5-AS1 or<br>↓IRF2BP2 after miR-155-5p mimics<br>↑IRF2BP2 after miR-155-5p inhibitor or OGD/R + IRF2BP2 OE or OGD/R + OIP5-AS1 OE<br>↑SOD but ↓ROS, MDA after OGD/R + IRF2BP2 OE or OGD/R + OIP5-AS1 OE + si-IRF2BP2	N/A
**[343]**<br>Alsegiani et al., 2023	Neuroinflammation	LPS, thrombin	SZ-3 (A novel small molecule cofilin inhibitor)	N/A	↓Microglial activation and suppressed cell proliferation and migration after LPS + SZ-3<br>↓NO, TNFα, PAR-1 after thrombin + SZ-3	N/A
**[344]**<br>Zhao et al., 2023	Transcriptome (esosomes)	POCD-derived exosomes, miR-584-5p mimics	N/A	miR-584-5p/ BDNF	301 miRNAs dysregulated (184 up-regulated and 117 down-regulated) after POCD-exosomes<br>↓viability, BDNF and p-TrkB after POCD-exosomes or miR-584-5p mimics<br>↑IL-1β, Caspase 3 and TNFα after POCD-exosomes and miR-584-5p mimics	Exosomes were isolated from the plasma of Sevoflurane (Sevo)-induced POCD or non-POCD patients
**[345]**<br>Ahmed et al., 2023	Neuroinflammation	H2O2, LPS, Mh-P2	α-glucan from the mollusk *Marcia hiantina*	N/A	↑ROS after LPS/H2O2<br>↓ROS after LPS/H2O2+Mh-P2	Mh-P2 (10, 50, 100 and 200 μg/mL), of H2O2 (10 or 50 μM), LPS (0.2 or 1.0 μg/mL)
**[346]**<br>McGraw et al., 2023	Viral infections (VEEV)	VEEV or VEEV-TrD and Eastern Equine Encephalitis Virus (EEEV)	NSC697923 (NSC), bardoxolone methyl (BARM) and omaveloxolone (OMA)	N/A	No effect on viability after OMA, BARM, NSC, YH1 and P00<br>↓VEEV titers after OMA/BARM/NSC/YH1/P00<br>↓VEEV titers after OMA/BARM/NSC in pre +post-/post-treatment<br>↓VEEV intracellular RNA after OMA/BARM/NSC pre+post-treatment	Inhibitors of ubiquitin proteasome system (UPS)-mediated signaling: OMA (0.1 μM), BARM (0.1 μM), NSC (0.5 μM), YH1 (0.5 μM), P00 (1 μM) and DMSO (0.1%) for 24 h

(Continued)

10.3389/fimmu.2026.1778798

SUPPLEMENTARY TABLE 1 Continued

ARTICLE	MODEL	TREATMENT	DRUG TESTED	PATHWAY INVOLVED	MAIN RESULTS	NOTES
					↓VEEV extracellular RNA after OMA/BARM/NSC pre + post/post-treatment<br>↓IL-1β, IL-6 and IL-8 after OMA/NSC 6 hpi<br>↓IL-6 and IL-8 after BARM 6 hpi or 18 hpi<br>No effect on IFNγ after OMA/BARM/NSC<br>↓IL-1β, IL-6 and IL-8 after OMA<br>↓IL-6 after NSC 18 hpi<br>↓VEEV-TrD and EEEV after OMA/BARM/NSC	
**[347]**<br>Yan et al., 2023	Neuroinflammation	CM (MC-LR-SH) and Microcystin-LR (MC-LR), TLR4-nAb	N/A	TLR4/NF-κB/ NLRP3	↑α-Syn, p-α-Syn, Iba1, IL-6, TNFα, INOS, COX2, CD86 after MC-LR-SH<br>↑Iba1, CD86 after 15 μg/L MC-LR<br>↓Arg-1, CCL22, CD163, IL-10, CD206 after MC-LR-SH<br>↑NLRP3, Caspase1, IL1β, and IL18 RNA after MC-LR-SH<br>↑ASC, NLRP3, Caspase1 p10, and IL-1β proteins after MC-LR-SH<br>↑TLR4, activation and nucleus translocation of NF-κB after MC-LR-SH<br>↓p-NF-κB, NLRP3, IL-1β, IL-18 after TLR4-nAb+ MC-LR-SH/MC-LR	CM of SH-SY5Y cells induced by 500 nM Microcystin-LR (MC-LR) (MC-LR-SH) and MC-LR (0, 500 nM)
**[348]**<br>Zong et al., 2023	Neuroinflammation	LPS	TD139	N/A	↑Galectin-3 expression, inflammation and oxidative stress after LPS dose-dependent<br>↓NLRP3, neuroinflammation and oxidative stress after LPS + TD139	2 h, 20 μM TD139 inhibitor of Galectin-3, prior to LPS intervention (0, 100 ng/ml and 500 ng/ml) for 24 h
**[349]**<br>Chakraborty et al., 2023	Neurodegenerative diseases (such as Alzheimer's, Parkinson's, and Huntington's diseases)	α-Syn, co-culture with SH-SY5Y	N/A	N/A	70% of the TNTs contained only F-Actin, 30% of the TNTs contained microtubule along with F-Actin<br>↑Mitochondrial particles inside F-Actin<br>↑Fibrils after α-Syn<br>↑α-Syn in HMC3 and within the TNT after co-culture<br>No effect on heterotypic TNTs after α-Syn	α-Syn for 16 h. Co-culture with α-Syn containing SH-SY5Y cells (donor cells).<br>Tunnelling nanotubes (TNT) >10 μm
**[350]**<br>Manzoor et al., 2023	Neuroinflammation	LPS	Novel triazole–pyrimidine hybrids	NF-κB pathway, iNOS	↓ NO<br>↓ pro-inflammatory cytokines (TNFα, IL-6)	LPS (1 μg/mL)
**[351]**<br>Sobral et al., 2023	Neuroinflammation	LPS and IFN-γ	N/A	NF-κB pathway	↓ pro-inflammatory cytokines	LPS (100 ng/mL) and IFN-γ. (20 ng/mL)
**[352]**<br>Zuniga et al., 2023	Neuroinflammation	LPS ± IFN-γ	N/A	NF-κB	↓ pro-inflammatory cytokines (TNFα, IL-6, IL-1β)	N/A

(Continued)

10.3389/fimmu.2026.1778798

SUPPLEMENTARY TABLE 1 Continued

ARTICLE	MODEL	TREATMENT	DRUG TESTED	PATHWAY INVOLVED	MAIN RESULTS	NOTES
[353]<br>Latif et al., 2024	Neuroinflammation	[3H]L-arginine isotope, unlabeled arginine, glutamate, TNFα, LPS	donepezil, quinidine, verapamil, tramadol	cationic amino acid transporter-1 (CAT-1)	↑L-arginine transport<br>time- and concentration-dependent arginine transport via CAT-1<br>↓Viability after glutamate<br>↑[3H]L-arginine uptake after glutamate, arginine, TNFα, or LPS<br>↓[3H]L-arginine uptake after co-treatment with unlabeled L-arginine<br>↓[3H]L-arginine uptake after donepezil, quinidine, verapamil, or tramadol	N/A
[354]<br>Fazzina et al., 2024	Parkinson's disease	IFN-γ boosted with glucose, 6-hydroxidopamine (6-OHDA), 1-methyl-4-phenyl-1,2,3,6-tetrahydropyridine (MPTP), rotenone	N/A	N/A	↑Stability of genes: HPRT1, RPS18, and B2M after rotenone<br>↓Stability of genes: GAPDH, HMBS, and ACTB after rotenone	6-OHDA 1 μM, IFN-γ 1 μg/mL + glucose 5 g/L, rotenone 0.1 μM to evaluate the list of GOI.
[355]<br>González et al., 2024	Neuroinflammation	IL-1β, LPS, and PMA, ethyl imidazo[1,2-a]pyridine-2-carboxylate (5a), ethyl imidazo [1,2-a]pyrimidine-2-carboxylate (5b), imidazo[1,2-a]pyridine-2-carboxylic acid (8a), imidazo[1,2-a]pyrimidine-2-carboxylic acid (8b), 4-(imidazo[1,2-a]pyridin-2-yl)<br>benzoic acid (9a), 4-(imidazo [1,2-a]pyrimidin-2-yl) benzoic acid (9b)	N/A	N/A	↑COX after IL-1β, LPS, and PMA<br>↑IC50 PGE2 after LPS + 9a/9b<br>↓PGE2 after 5a and 5b, concentration-dependent<br>↑imidazo[1,2-a]pyridines effective than imidazo[1,2-a] pyrimidines<br>↑8a active (IC50 = 50.97 μM)<br>5a and 5b as prodrugs, hydrolyzed by carboxylesterases to 8a and 8b	
[356]<br>Consoli et al., 2024	Neuroinflammation	carbon-BNQDs, phenol-BNQDs, 5-FU, PB4	N/A	N/A	↑Viability after carbon-/phenol-BNQDs+5-FU/PB4 (6 h and 24 h)<br>100% viability after phenol-BNQDs+PB4<br>↑HMOX1 gene after BNQDs<br>↓HMOX1 gene after PB4 (6 h and 24 h)<br>↓GPX1 and IL-8 after PB4<br>↑TXN and GSTP1 after PB4<br>↑IL-8 after BNQDs+PB4<br>↓TXN and GSTP1 after BNQDs+PB4 24 h<br>No changes in differentially expressed genes	Boron nitride quantum dots (BNQDs) at concentration 4 μg 200 μL^{-1}.<br>5-FU (100 μM) or PB4 (0.15 μM).
[357]<br>Stangis et al., 2024	Viral infection (SARS-CoV-2)	HBMEC conditioned media after S1 protein (S1-CM) or S1 D614 (S1D-CM), LPS, IL-6	N/A	IL-6/STAT3	↑Nuclear/cytoplasm STAT3 ratio after S1-CM (15 min)<br>No changes in STAT3 expression after IL-6	Human brain microvascular endothelial cells (HBMEC) CM after 48 h treatment with D614 S1 protein from SARS-CoV-2.<br>LPS or recombinant human

(Continued)

10.3389/fimmu.2026.1778798

SUPPLEMENTARY TABLE 1 Continued

ARTICLE	MODEL	TREATMENT	DRUG TESTED	PATHWAY INVOLVED	MAIN RESULTS	NOTES
						IL-6 (350 pg/mL) for 15 min or 6 h were used as positive controls
**[358]**<br>Naaldijk et al., 2024	Neuroinflammation	IFN-γ, IL-4	N/A	N/A	↑ Human leukocyte antigen (HLA)-DR and CD14 but no changes in CD11b after IFNγ<br>No changes in HLA-DR, CD11b and CD14 after IL-4	50 ng/ml IFNγ (M1 induction) or 20 ng/ml IL-4 (M2 induction) for 24 h
**[359]**<br>Zhang et al., 2024	Neuroinflammation	H2O2, 10% DMSO, LPS	ellorarxine	N/A	↑Cyp26b1 and RARβ, but not RARα or RARγ after ellorarxine<br>↓viability after ellorarxine alone or +H202<br>↑cell death after ellorarxine alone or +H202/LPS<br>↑TNFα and Il-6 after LPS<br>↓IL-6 after ellorarxine+LPS<br>↑LC3BII and autophagy after ellorarxine	10 nM ellorarxine 4 h, 100 or 200 mM H2O2, 15 μg/mL LPS
**[360]**<br>Gentili et al., 2024	Alzheimer's disease	DAG-MAC-BHB (novel ketone diester), low glucose, Aβ1-42, CLI-095 (TLR4 inhibitor), Nigicerin, ATP, monosodium urate crystals (MSU), flagellin/ DOTAP complex, Bacillus anthracis lethal factor, poly(dA: dT)/lipofectamine complex.	DAG-MAC-BHB	Cellular viability, Acetyl-CoA, ATP, metabolic enzymes, NLRP3, mitochondrial activity, ROS, phagocytosis	• No NLRP3 activation, no change in IL-1β by Aβ1-42.<br>• Altered morphology, ↓viability, ↑pyruvate, ↓lactate by low glucose.<br>• NLRP3 activation, ↑IL-1β, ↑caspase-1 p20, ↑NLRP3 mRNA, ↑ASC, ↓phagocytosis, ↓ΔΨm, ↓ATP, ↑ROS by Aβ1-42 + low glucose.<br>• ↑Acetoacetate, ↑Acetyl-CoA, ↓pyruvate, ↑BDH1, ↑SCOT, ↑ATP by DAG-MAC-BHB + low glucose.<br>• ↓NLRP3 activation, ↓IL-1β, ↓caspase-1 p20, ↓NLRP3 mRNA, ↓ASC, ↓phagocytosis, ↑ΔΨm, ↑ATP, ↓ROS by DAG-MAC-BHB + Aβ1-42 + low glucose.	*In vivo:* ICR mice.<br>*In vitro:* human intestinal adenocarcinoma Caco-2, human brain endothelial hBMEC and human microglial HMC3 cells.<br>DAG-MAC-BHB: 10 mM for 0-24 h.<br>Normal glucose: 25 mM.<br>Aβ1-42: 1 μM for 24 h.<br>Low glucose: 5 mM for 18-24 h.<br>CLI-095: 50 nM for 24 h.<br>Nigericin: 10 g/ml for 1 h.<br>ATP: 2 mM for 2 h.<br>MSU: 200 μg/ml overnight.<br>Flagellin/DOTAP complex: 1 μg/ml e 3 μl, respectively, for each well.<br>Bacillus antracis lethal factor: 1 μg/ml for 3-6 h.<br>poly(dA:dT)/lipofectamine complex: 2 μg/ml.
**[361]**<br>Spigarelli et al., 2024	Cancer (GBM)	N/A	B-Caryophyllene (BCA), eugenol (EU)	Metabolic activity, viability, cell cycle, mitochondrial membrane potential, BCL2, BAX, BAK1, TP53, PTEN, MDM2, VGFA, IL-6, IL-4, IL-8, TNFα, caspase 9	• No changes (metabolic activity, viability, cell cycle, ΔΨm, caspase 9), ↓BCL2, ↑BAK1, ↑TP53, ↑MDM2, ↓VGFA, ↓IL-6, ↓IL-4, ↓IL-8, ↓TNFα by BCA.<br>• No changes (metabolic activity, viability, cell cycle, ΔΨm, caspase 9), ↓BCL2, ↓BCL2L1, ↓BAX, ↓MDM2, ↑PTEN, ↓VGFA, ↓IL-4 by EU.<br>• No changes (metabolic activity, viability, cell cycle,	*In vivo:* male Sprague-Dawley rats.<br>*In vitro:* U87 GB and HMC3 cells.<br>BCA: 1.25, 12.5, 125 μM for 24, 48, 72 h.<br>EU: 2, 20, 200 μM for 24, 48,

(Continued)

10.3389/fimmu.2026.1778798

SUPPLEMENTARY TABLE 1 Continued

ARTICLE	MODEL	TREATMENT	DRUG TESTED	PATHWAY INVOLVED	MAIN RESULTS	NOTES
					ΔΨm, caspase 9), ↓BCL2, ↓BAK1, ↓MDM2, ↓IL-6, ↓IL-4, ↓IL-8, ↓TNFα by BCA + EU<br>• EU and BCA demonstrated marked multitarget cytotoxic/anti-proliferative synergistic activity in U87 cells, whereas such effects were not observed in HMC3 cells.	72 h.<br>BCA+EU: 1.25 + 2 or 12.5 + 20 or 125 + 200 µM, respectively, for 24, 48, 72 h.
**[362]** Karimi Roshan et al., 2024	Alzheimer's disease	High- and low-linear energy transfer (LET) radiation, ^{241}Am alpha (α) particles, X-ray radiation	N/A	Viability, DNA damage, DDR genes, Clonogenic survival, NF-κB, pro-inflammatory cytokines, anti-inflammatory markers, phagocytosis, morphology	• ↑DNA damage, ↑DDR genes (greater upregulation with higher α proportion), ↓viability, ↑IL-1β, ↑nuclear and cell size by acute treatment.<br>↑DNA damage, ↑DDR genes, ↓viability, ↑IL-1β, ↑TNFα, ↑nuclear and cell size by fractionated treatment.	Radiation treatment: acute single dose 0.5-2 Gy, fractionated 0.4 Gy/day x 5 days = 2 Gy.<br>Beams: α-particles, X-rays, or mixed.
**[363]** Son et al., 2024	Alzheimer's disease	4′-O-Methylalpinum isoflavone (mAI), cholesterol, 24-hydroxycholesterol (24OHchol), 25-hydroxycholesterol (25OHchol), 27-hydroxycholesterol (27OHchol)	N/A	Cell viability, IL-6, IL-1β, MHC II, ERK	• No change in viability by mAI.<br>• No change in viability, ↑IL-6, ↑MHC II, ↑IL-1β by 25OHchol or 27OHchol.<br>• ↓IL-6, ↓MHC II, ↓IL-1β by mAI + 25OHchol or 27chol compared to 25OHchol or 27OHchol.<br>• ↑p-ERK by 25OHchol at 3, 6 and 9 h.<br>• ↓p-ERK by mAI + 25OHchol at 6 and 9 h compared to 25OHchol.	mAI: 1 µg/ml for 3, 6, 9, 48 h.<br>25OHchol: 1 µg/ml for 3, 6, 9, 48 h.<br>cholesterol, 24OHchol: 1 µg/ml for 48 h.<br>27OHchol: 1 µg/ml for 48 h.
**[364]** Sood et al., 2024	Parkinson's disease	malvidin-3-O-glucoside (M3G), α-Syn), 1-methyl-4-phenyl-1,2,3,6-tetrahydropyridine (MPTP) mitochondrial neurotoxin, ML385 (Nrf2 inhibitor)	N/A	Viability, Bax, Casp-3, Casp-8, apoptosis, IL-1β, IL-6, TNFα, IL-4, TGF-β, ROS, Nrf2, HO-1, HO-2, CAT, SOD, GPx	• No change in viability by M3G.<br>• ↓viability, ↑Bax, ↑Casp-3, ↑Casp-8, ↑apoptosis, ↑IL-1β, ↑IL-6, ↑TNFα, ↑IL-4, ↑TGF-β, ↑ROS, ↑Nrf2, ↑HO-1, ↑CAT, ↑SOD, ↑HO-2, ↑GPx by α-Syn + MPTP.<br>↑viability, ↓Bax, ↓Casp-3, ↓Casp-8, ↑Bcl-2, ↓apoptosis, ↓IL-1β, ↓IL-6, ↓TNFα, ↑IL-4, ↑TGF-β, ↓ROS, ↑CAT, ↓SOD, ↑HO-2, ↑GPx by M3G + α-Syn + MPTP compared to α-Syn + MPTP.	M3G: 6.25 - 100 µM for 24 h.<br>α-Syn: 0.5 - 8 µg/ml for 24 h.<br>MPTP: 0.25 - 2 mM for 24 h.<br>ML385: 5 µM for 24 h.
**[365]** Filaferro et al., 2024	Parkinson's disease	Walnut oil (WO), rotenone (ROT)	N/A	Cell viability, ROS	• ↓viability, ↑ROS by ROT.<br>↑viability, ↓ROS by WO + ROT compared to ROT.	Identification and quantification of fatty acids in WO.<br>*In vitro:* SH-SY5Y neuroblastoma and HMC3 microglial cells.<br>WO: 12.5 - 500 µg/ml for 24 h (pre-treatment) and 24 - 48 h (treatment).<br>ROT: 0.1 - 15 µM for 24 - 48 h.<br>CM from WO-treated HMC3 cells enhanced SH-SY5Y cell survival.

(Continued)

10.3389/fimmu.2026.1778798

SUPPLEMENTARY TABLE 1 Continued

ARTICLE	MODEL	TREATMENT	DRUG TESTED	PATHWAY INVOLVED	MAIN RESULTS	NOTES
**[366]**<br>Feng et al., 2024	Alzheimer's disease	Aβ1-42, ML385 (Nrf2 inhibitor)	quercetin	Cell viability, Nrf2, HO-1, Nrf2, HO-1, LDH, IL-1β, IL-6, TNFα, MDA, SOD, GSH/GSSG, CD86, CD80, CD206, CD163, apoptosis	• No change in viability by quercetin.<br>• ↓viability, ↓Nrf2, ↓HO-1, ↑LDH, ↑IL-1β, ↑IL-6, ↑TNFα, ↑MDA, ↓SOD, ↓GSH/GSSG, ↑CD86, ↑CD80, ↓CD206, ↓CD163, ↑apoptosis by Aβ1-42.<br>• ↑viability, ↑Nrf2, ↑HO-1, ↓LDH, ↓IL-1β, ↓IL-6, ↓TNFα, ↓MDA, ↑SOD, ↑GSH/GSSG, ↓CD86, ↓CD80 ↑CD206, ↑CD163, ↓apoptosis by quercetin + Aβ1-42 compared to Aβ1-42.<br>ML385 reversed quercetin's effects.	*In vivo:* male wild-type C57BL/6 mice<br>Que: 1-10 µM for 24 h.<br>Aβ1-42: 1 µM for 24 h.<br>ML385: 5 µM for 24 h.
**[367]**<br>Madsen et al., 2024	Neuroinflammation	Urolithin A (UA), Nicotinamide Riboside (NR), doxorubicin (DOX), cGAMP, cGAS-STING pathway stimulation by transfection (DNA fragments), poly (I:C) (transfection by an RNA mimic to stimulate the RIG-I/MAVS pathway)	N/A	Senescence, pSTING, IRF3, TREM2, OAS1, CXCL10, OCR, spare respiratory capacity, glycolytic function, mito-ROS	• No change in senescence, ↑maximal OCR, ↑spare respiratory capacity, ↑maximal glycolytic function, ↑mito-ROS by UA.<br>• No change in senescence, ↑maximal OCR, ↑spare respiratory capacity by NR.<br>↓SA-β-gal by UA or NR + doxorubicin compared to doxorubicin.<br>• ↑pSTING, ↑IRF3 nuclear translocation, ↑TREM2, ↑OAS1, ↑CXCL10 by UA + DNA fragments.<br>• ↓IRF3 nuclear translocation, ↓TREM2, ↓OAS1, ↓CXCL10 by NR + DNA fragments or poly(I:C).<br>• ↓IRF3 nuclear translocation by NR + poly(I:C).<br>•	UA: 5-20 µM for 6-7 days.<br>NR: 1.5-6 mM for 6-7 days.<br>Doxorubicin: 100 nM for 24 h.<br>cGAMP: 30 µM added to the full medium.<br>Poly(I:C): 0.1 µg/ml<br>DNA fragments: 25 ng/well (8-well) for 12 h.
**[368]**<br>Hacioglu et al., 2024	Neuroinflammation	Sucralose	N/A	Cell viability, proliferation, migration, morphology, microglial activation, inflammation, ferroptosis, SIRT1/NLRP3/IL-1β/GPx4 pathway	• ↓Viability, ↓proliferation, ↓migration, membrane and nuclear abnormalities induced by sucralose.<br>↓Fe^{+2}, ↓GPx4, ↓GSH, ↓SIRT1, ↑IL-1β, ↑MDA, ↑NLRP3, ↑8-OHdG, ↑caspase 3/7 activity by long-term sucralose treatment.	Sucralose: 0-50 mM for 24, 48, 72 h for short term effects/1 mM for 7, 14, 21 days for long term effects.
**[369]**<br>Alami et al., 2024	Neuroinflammation, Alzheimer's disease	human Aβ1-42, TBHP, LPS	pomegranate polyphenols (PPs) (punicalagin, ellagic acid, peel, and aril extracts)	ROS, lipid peroxidation, CD86, CD163	• ↑ROS by Aβ1-42.<br>• ↓ROS by peel or aril or punicalagin extracts pre-treatment + Aβ1-42 compared to Aβ1-42.<br>• ↑Lipid peroxidation by TBHP.<br>• ↓Lipid peroxidation by peel or aril or punicalagin or ellagic acid extracts pre-treatment + TBHP compared to TBHP.<br>• ↑CD86, ↓CD163 by LPS.<br>• ↓CD86, ↑CD163 by peel or aril or punicalagin extracts + LPS co-treatment compared to LPS.<br>• ↑CD163 by ellagic acid extract + LPS co-treatment compared to LPS.	*In vitro:* THP-1 macrophages, human microglia HMC3 cells and U373-MG human astrocytes.<br>Peel and aril extracts: 100, 200, 400 µg/ml for 2, 24 h.<br>Punicalagin and ellagic acid extracts: 20, 50, 100 µg/ml for 2, 24 h.<br>Aβ1-42. 5 µg/ml for 4, 24 h.<br>TBHP: 200 µM for 1 h.<br>LPS: 1 µg/ml for 24 h.
**[370]**<br>Barczuk et al., 2024	Neuroinflammation, Alzheimer's disease	Noradrenaline, LPS, Aβ	N/A	Cell viability, morphology, proliferation, DNA damage, Caspase-3, DDIT-	• ↑Cell viability, better morphology, ↑proliferation, ↓DNA damage, ↓Caspase-3 activity, ↓DDIT3, ↓BAX, ↑BCL-2, ↓HIF-1α, ↓CHOP, by NA + LPS or Aβ	NA: 1, 2.5, 5,10, 25, 50, 100, 200 µM for 24, 48 h.<br>LPS: 5-100 µg/ml (IC50:

(Continued)

10.3389/fimmu.2026.1778798

SUPPLEMENTARY TABLE 1 Continued

ARTICLE	MODEL	TREATMENT	DRUG TESTED	PATHWAY INVOLVED	MAIN RESULTS	NOTES
				3, BAX, BCL-2, Aβ deposits, OCR, ECAR	compared to LPS or Aβ.<br>• ↓Aβ extracellular and intracellular deposits by NA +Aβ compared to Aβ.<br>No effects on p-JNK/JNK and respiration (OCR, ECAR) by NA treatment.	101.28 μg/ml) for 24, 48 h.<br>Aβ: 1-200 μM (IC50: 24.52 μM) for 24, 48 h.
**[371]**<br>Luo et al., 2024	Neuroinflammation, Cancer (GBM)	Co-culture with MC9999 chimeric antigen receptor (CAR) T cells targeting programmed death-ligand 1 (PD-L1)	N/A	PD-1/PD-L1 immune checkpoint	• PD-L1 is present in HMC3.<br>↑cytolysis and monolayer disruption by MC9999 CAR T cells.	Study on CAR T cell therapy.<br>*In vivo:* NOD scid gamma (NSG) mice.<br>*In vitro:* MDA-MB-231, Calu-1, SH-4, LN229, 293FT, Jurkat, HMC3, patient-derived GBM and T cells.
**[372]**<br>Huynh et al., 2024	Alzheimer's disease	Myelin debris, K604 (ACAT1 inhibitor), F12511 (ACAT1 inhibitor), GSK2033 (LXR antagonist)	N/A	cholesterol metabolism, ABCA1, LXR	• ↑cholesteryl esters, ↑ABCA1 by myelin debris.<br>• ↓cholesteryl esters, ↓cholesterol, ↑ABCA1 by K604 or F12511 + myelin debris compared to myelin debris.<br>• ↓ABCA1 by GSK2033 + K604 or F12511 + myelin debris compared to K604 or F12511 + myelin debris.<br>•	*In vitro:* mouse N9 and human HMC3 cells.<br>Myelin debris treatment: cholesterol 5-25 μg/ml for 24 h.<br>K604: 0.5 μM for 24 h.<br>F12511: 0.5 μM for 24 h.<br>GSK2033: 5 μM for 24 h.
**[373]**<br>Chmielarz et al., 2024	Metabolic endotoxemia (ME)	TNFα, LPS, PA	N/A	TLR4, lipid metabolism, TREM2	• ↓Viability by LPS + PA compared to controls.<br>• ↑TGF-β by LPS or PA or LPS + PA compared to controls.<br>• ↑Trem2 mRNA by LPS or PA compared to controls.<br>• ↓Trem2 mRNA by LPS + PA compared to LPS.<br>• ↑Triacylglyceride 48:0, ↑triacylglyceride 60:11 by PA compared to controls.<br>•<br>• ↑Triacylglyceride 48:0, ↑triacylglyceride 60:11 by LPS + PA compared to controls.<br>• ↓Sphingomyelin d18:1/14:0, ↓Sphingomyelin d18:1/24:1 by LPS + PA compared to controls.<br>• ↑Long-chain ceramides by LPS compared to controls.<br>• ↓Phosphatidylcholine 36:2, ↑phospatidylcholine 32:0, ↑phospatidylcholine 34:0 by LPS + PA compared to controls.	TNFα pre-treatment: 25 ng/ml for 24 h.<br>LPS: 10 ng/ml for 24 h.<br>PA: 200 μM for 24 h.
**[374]**<br>Miao et al., 2024	Alzheimer's disease	SP3-12 (competitive SHIP-1 inhibitor)	N/A	SHIP-1, phagocytosis	• ↑Phagocytosis by SP3-12 EC_{50} 2 μM.<br>No changes in nuclear intensity or cell counts by SP3-12 up to 10 μM concentration.	High-throughput screening of 49,260 central nervous system-penetrate compounds.<br>*In vitro:* HMC3 cells.<br>SP3-12: 3 nM -60 μM for 48 h.

(Continued)

10.3389/fimmu.2026.1778798

SUPPLEMENTARY TABLE 1 Continued

ARTICLE	MODEL	TREATMENT	DRUG TESTED	PATHWAY INVOLVED	MAIN RESULTS	NOTES
**[375]** Ontiveros-Angel et al., 2024	Childhood overweight/obesity	PA, hydrocortisone (Cort), FKBP5 silencing (siRNA)	N/A	Cell proliferation, inflammation, ROS.	• ↑Cell proliferation, ↑ROS by PA.<br>• ↓TNFα, ↓IL-6 by PA.<br>• ↑TNFα, ↑IL-6, ↑ROS, ↑CD68, ↑NF-κB by PA + Cort.<br>↓TNFα by PA + CORT + FKBP5 siRNA.	*In vivo:* adolescent Lewis rats. Treatment with PA 50 μM for 24 h, followed by an additional incubation with CORT 100 nM for 24 h.
**[376]** Lin et al., 2024	Parkinson disease	Synthetic coumarin chalcone derivatives (LM-009, LM-016, LM-021, LM-036), 1-methyl-4-phenyl pyridinium (MPP^+)	N/A	Cell viability, NLRP3, CASP1, iNOS, inflammation, microglial activation	• ↓Cell viability, ↑NO, ↑CD68/MHCII, ↑NLRP3, ↑iNOS, ↑CASP1, ↑IL-1β, ↑IL-6, ↑TNFα by MPP^+.<br>• ↑Cell viability, ↓NLRP3, ↓CASP1, ↓iNOS, ↓IL-1β, ↓IL-6, ↓TNFα, ↓NO by LM-016 or LM-021 or LM-036 + MPP+ compared to MPP^+.<br>• These effects were not induced by LM-009.<br>•	*In vitro:* neuroblastoma BE (2)-M17 and human microglia HMC3 cells. LM compounds pre-treatment: 1-10 μM for 8 h. MPP^+ treatment: 0-8 mM for 20 or 24 h.
**[377]** Gao et al., 2024	Neuroinflammation	LPS, poly IC	N/A	Inflammation, microglial activation, phagocytosis	• HMC3 showed lower microglial markers expression and phagocytosis than iMG.	Comparison between microglia-like cells derived from stem cells (iMG) and HMC3.<br>*In vitro:* iMG cells, cocultured with retinal organoids, and HMC3 cells.<br>LPS: 1 ng/ml for 6-24 h.<br>Poly IC: 1 μg/ml for 24 h.
**[378]** Apaza Ticona et al., 2024	Alzheimer's disease	N/A	3 pentacyclic triterpenes compounds extracted from *Mauritia flexuosa*	NF-κB, PGE2, IDO1, EGFR	Inhibition of NF-κB, PGE2, IDO1 and EGFR by compound 3 ((3β)-3-hydroxy-11-oxours-12-en-28-oic acid) with IC_{50} values 9.83, 3.86, 1.63 μM, and 49.57 nM, respectively.	*In vitro:* SVG p12 and HMC3 cell lines.
**[379]** Tan et al., 2024	Viral infection (Borna disease virus 1 (BoDV-1) encephalitis (BVE))	BoDV-1 infection, ferrostatin 1 (Fer-1) for ferroptosis inhibition, MG132 for proteasome inhibition, deferoxamine (DFO) for iron chelation, N-acetylcysteine (NAC) to scavenge ROS	chloroquine (autophagy inhibitor)	Cell viability, oxidative stress, ferroptosis, iron overload, NRF2	• ↓Cell viability, ↓GSH, ↓SOD, ↑iron, ↑MDA, ↑ROS, ↓GPX4, ↓HO-1, ↓SLC7a11, ↓FTH1, ↓pNrf2, ↑Keap1, ↑PTGS2 by BoDV-1 infection.<br>• ↓BoDV-1-induced ferroptosis by Fer-1, DFO or NAC.<br>• MG132 reversed alteration in cell viability, iron, MDA, SOD, GSH, Nrf2, Keap1, HO-1, SLC7a11, GPX4, FTH1 induced by BoDV-1 infection.<br>Chloroquine reversed only cell viability reduction induced by BoDV-1 infection.	*In vivo:* Sprague-Dawley rats.<br>*In vitro:* primary rat cortical neurons and HMC3 cells.<br>Chloroquine: 10 μM on Day 1.<br>Fer-1: 10 μM on Day 1.<br>MG132: 10 μM on Day 1.<br>DFO: 10 μM on Day 1.<br>NAC: 5 mM on Day 1.<br>BoDV-1: MOI 5 on Day 2.<br>Assays on Day 4.
**[380]** Tan et al., 2024	Neuroinflammation	LPS, PKA (CREB activator), 653-47 (CREB inhibitor), PDCD4 overexpression plasmid (oePDCD4), PDCD4 silencing (sh-PDCD4), miR-181c mimics/inhibitor	Oxycodone	CREB, miR-181c, PDCD4, iNOS, cytokines, inflammation	• ↑miR-181c, ↑p-CREB, ↓PDCD4, ↓iNOS, ↓TNFα, ↓IL1-β, ↓IL-6, ↓IL-8 by oxycodone + LPS compared to LPS.<br>• ↓cytokines, ↓PDCD4, ↓iNOS by miR-181c mimics + LPS compared to LPS, while these effects were countered by miR-181c inhibition or PCDC4 overexpression.	LPS pre-treatment: 100 ng/ml for 24 h.<br>Oxycodone: 2.5, 5, 10, and 20 μg/ml for 24 h.<br>PKA: 5 μg/ml for 6 h.<br>653-47: 100 nM for 3 h.

(Continued)

10.3389/fimmu.2026.1778798

SUPPLEMENTARY TABLE 1 Continued

ARTICLE	MODEL	TREATMENT	DRUG TESTED	PATHWAY INVOLVED	MAIN RESULTS	NOTES
					↑miR-181c, ↓inflammation by CREB activation, while CREB inhibition had the opposite effect.	
[381] Distefano et al., 2024	Neuroinflammation	1R6F cigarette smoke aqueous extract (AqE), IQOS heated tobacco product aerosol AqE, nicotine	N/A	Cell viability, oxidative stress, lipid peroxidation, nitrosative stress, mitochondrial functionality, Unfolded Protein Response, DNA damage, phagocytosis	• ↓Cell viability, ↑ROS, ↑lipid peroxidation, ↓GSH, ↑nitrite+nitrate, ↓ATP/ADP, ↓energetic charge, ↓NAD +/NADH, ↓total nucleoside triphosphates, ↑DSBs, ↑MFN1, ↑MFN2, ↑FIS1, ↓DRP1, ↑SIRT1, ↓IRE1α, ↑BIP, ↑PERK, ↓BAX, ↑HSP60, ↓CLPP, ↓NRF2 nuclear translocation, ↑NF-κB nuclear translocation, ↑HMOX1 mRNA, ↓HO-1 protein, ↑IL-18 mRNA, ↓Klotho β, ↓phagocytosis by 1R6F AqE compared to untreated.<br>• IQOS AqE induced milder toxicity and stress compared to 1R6F AqE with ↑viability, ↓oxidative stress, ↓Nitrosative stress, ↓mitochondrial and energetic impairments, ↓ inflammation, ↓DNA damage, ↑Phagocytosis.<br>Nicotine standard was largely non toxic and induced ↓Oxidative stress and ↓inflammation than 1R6F AqE.	Quantification of nicotine concentration in 1R6F AqE and IQOS AqE.<br>Cells were exposed to 1R6F AqE, IQOS AqE and a nicotine standard, all at the same nicotine concentration of 340 nM for 6-24 h.<br>IC_{50} determination for 1R6F AqE by MTT: 340 nM nicotine concentration.
[382] Li et al., 2024	Uveitis	LPS, IFN-γ, EGR2 overexpression (oeEGR2), GDF15 knockdown (shGDF15)	N/A	EGR2, GDF15, inflammation, migration, proliferation	• ↑iNOS, ↑IL-1β, ↑TNFα, ↓EGR2, ↑migration, ↑proliferation by LPS + IFN-γ.<br>• ↓iNOS, ↓IL-1β, ↓TNFα, ↓migration, ↓proliferation by oeEGR2+LPS + IFN-γ compared to LPS + IFN-γ.<br>ShGDF15 reversed the anti-inflammatory, anti-migratory and anti-proliferative effects of EGR2.	*In vivo:* mice model with conditional knockout of EGR2 in microglia.<br>LPS 1 μg/ml + IFN-γ 500 ng/ml for 24 h.<br>oeEGR2 transfection: MOI=5.<br>shGDF15 transfection: MOI=30.
[383] Di Chiano et al., 2024	Neuroinflammation	LPS	Lactobacilli-derived cell-free supernatants (CFSs) from 3 human gut probiotic species (Lp10, Lr13 and Lrh19)	NRF2, GPx, SOD1, TNFα, IL-10	• ↑NRF2 by Lp10 + LPS treatment compared to LPS.<br>• ↑NRF2, ↑GPx, ↑SOD1 by LPS + CFS compared to LPS.<br>• ↑IL-10 by LPS + Lrh19 compared to LPS.<br>• ↑IL-10 by CFS + LPS compared to LPS.<br>• ↑TNFα by LPS + Lr13 compared to LPS.<br>↓TNFα by CFS + LPS compared to LPs.	CFS 5% (v/v) 20 h then LPS 1 μg/ml 3h or LPS 3 h then CFS 20 h.
[384] Sangineto et al., 2024	Neuroinflammation	LPS	Dimethyl-fumarate (DMF), itaconate (ITA)	Glycolysis, Mitochondrial respiration, inflammation	• ↑Glycolysis, ↑mitochondrial respiration, ↑complex I/II/V activities, ↑TNFα, ↑IL-1β, ↑IL-6 by LPS.<br>• ↓Glycolysis, ↓mitochondrial respiration, ↓TNFα, ↓IL-1β, ↓IL-6 by DMF or ITA + LPS co-treatment compared to LPS.<br>• DMF + LPS co-treatment restores complex I activity compared to LPS.<br>• ITA + LPS co-treatment reduces complex I, restores complex II and increases complex V activities compared to LPS.<br>•	LPS: 1 μg/ml for 4 or 24 h.<br>DMF: 50 μM for 4 or 24 h.<br>ITA: 5 mM for 4 or 24 h.

(Continued)

10.3389/fimmu.2026.1778798

SUPPLEMENTARY TABLE 1 Continued

ARTICLE	MODEL	TREATMENT	DRUG TESTED	PATHWAY INVOLVED	MAIN RESULTS	NOTES
**[385]** Zhang et al., 2024	Diabetic neuropathic pain (DNP)	HG, miR-34a-5p	N/A	Inflammation, proliferation, polarization	• ↑Onflammation, ↑proliferation, ↑polarization by high glucose.<br>Mir-34a-5p knockdown showed a significant protective effect of microglia activation by high glucose. This effect was reversed by silencing ENPP3.	*Clinical study*: 181 diabetes patients enrolled, among which 74 patients developed DNP.<br>*In vitro:* HMC3 cells.
**[386]** Kim et al., 2024	Neuroinflammation	25-hydroxyclolesterol (25OHchol), 27-hydroxycholesterol (27OHchol), Aβ1-42	N/A	Expression of HSP60 on the surface of microglia activated by oxysterols	↑HSP60 by 25OHchol or 27OHchol or Aβ1-42.	25OHchol: 1 μg/ml for 48 h.<br>27OHchol: 1 μg/ml for 48 h.<br>Aβ1-42: 5 or 10 μM for 48 h.
**[387]** Hu et al., 2024	Retinal microglial inflammation	Hypoglycemia	N/A	Cell viability, inflammation	↓Cell viability, ↑TNFα, ↑IL-6, ↑IL-1β by hypoglycemia	*In vivo:* male Sprague-Dawley rats.<br>*In vitro:* HMC3 and HRMECs. Co-culture HMC3 and HRMECs.<br>Glucose control: 25 mmol/l.<br>Hypoglycemia: glucose 1 or 2.5 mmol/l for 3, 6, 12, 24 h.<br>Reduced claudin-1 and occluding expression in HRMECs co-cultured with hypoglycemic HMC3.
**[388]** Li et al., 2024	Alzheimer's disease	*P. gengivalis*, QNZ (NF-κB signalling pathway inhibitor)	N/A	Cell viability, NF-κB signaling, inflammation, polarization	• ↓Cell viability, ↑IL-6, ↑IL-17, ↓IL-10, ↑iNOS, ↑CD86, ↓CD206, ↑p-P65, ↓p-IKBα by *P. gingivalis*.<br>QNZ pretreatment reversed reduced viability, M1 polarization and increased inflammatory factors induced by *P. gingivalis*.	*In vivo:* sex-week old C57BL/6 mice.<br>*P. gingivalis* supplementation: 1-10% for 24 h.<br>QNZ: 9 μM for 30 min before *P. gingivalis* supplementation.
**[389]** Shu et al., 2024	Neuroinflammation/ neurotoxiciy	triclosan (TCS), siRNAs: si-STAT3 and si-hnRNPA1, PKM2 overexpression plasmids, TEPP-46 (PKM2-specific allosteric activator that promotes PKM2 tetramer formation), Stattic (inhibitor of STAT3 phosphorylation at Tyr705)	N/A	STAT3, inflammation, polarization	• ↑IL-1β, ↑IL-6, ↑TNFα, ↑Iba-1, ↑p-STAT3, ↑PKM2 by TCS.<br>• Si-STAT3 or Stattic reduces cytokine induction.<br>• TEPP-46 suppresses nuclear PKM2 and cytokine induction.<br>• Si-hnRNPA1 decreases cytokines and shifts PKM2/PKM1 balance.	*In vivo:* 8-week-old female rats (SD strain).<br>TCS: 0.1, 1, 10 μM for 24 h (for cell viability: 0.01 – 100 μM for 24 h, toxicity above 20 μM).
**[390]** Li et al., 2024	Neuroinflammation (Cerebral ischemia-reperfusion injury (CIRI))	OGD/R, GPR68 overexpression, LPS	N/A	GPR68, NF-κB, HIF-1α, inflammation, oxidative stress, nitrosative stress, apoptosis	• ↓GPR68, ↑TNFα, ↑IL-1β, ↑IL-6, ↑NO, ↑ROS, ↑apoptosis by OGD/R.<br>• ↑GPR68, ↓TNFα, ↓IL-1β, ↓IL-6, ↓NO, ↓ROS, ↓apoptosis, ↓p-NF-κB, ↓HIF-1α by GPR68 overexpression + OGD/R compared to OGD/R.<br>• ↑TNFα, ↑IL-1β, ↑IL-6, ↑NO, ↑ROS, ↑p-NF-κB, ↑HIF-1α by GPR68 overexpression + OGD/R + LPS	*In vivo:* middle cerebral artery occlusion/reperfusion (MCAO/R) mouse model.<br>*In vitro:* SH-SY5Y and HMC3 cells.<br>OGD/R: 6 h in glucose-free, serum-free medium at 1% O2,

(Continued)

10.3389/fimmu.2026.1778798

SUPPLEMENTARY TABLE 1 Continued

ARTICLE	MODEL	TREATMENT	DRUG TESTED	PATHWAY INVOLVED	MAIN RESULTS	NOTES
					compared to GPR68 overexpression + OGD/R.<br>•	then reoxygenation for 24 in high-glucose medium.
**[391]** Zhao et al., 2024	Cancer (GBM)	co-culture with U118 and U251 glioma cell lines,<br>BRMS1 overexpression (lentiviral vector)	N/A	BRMS1, SPP1, inflammation, polarization	• ↑CD209, ↑CD204, ↑TGF-β1, ↑BRMS1, ↑SPP1, ↑CXCL2, ↑IL-10 by GBM cells co-culture.<br>Significant upregulation of M2 markers and SSP1 after BRMS1 overexpression compared to HMC3 cells after co-culture.	U118 and U251 glioma cell lines and HMC3 cells. Co-culture of U118/U251 and HMC3 cells.<br>HMC3 BRMS1-overexpression: 24 h post-transfection, co-culture with U118/U251 cells for 72 h.
**[392]** Li et al., 2024	Post-traumatic stress disorder (PTSD)	co-culture with peripheral blood mononuclear cells (PBMCs) collected from Veterans with and without PTSD, with and without friend leukemia virus integration 1 (FLI1) Gapmer (antisense oligonucleotide) knockdown.	N/A	PBMCs-HMC3 crosstalk, inflammation	• ↑IL-6, ↑IFN-γ in HMC3 supernatants co-cultured with LPS-stimulated PBMCs from PTSD patients.<br>FLI1 Gapmer in PTSD PBMCs suppresses HMC3 IL-6 and IFN-γ increases.	co-culture model: PBMCs and HMC3 cells.
**[393]** Wang et al., 2024	Neuroinflammation	Neoprzewaquinone (NEO) from *Salvia miltiorrhiza*, IL-15	Cephalothin (CEP), inhibitory effect on IL-15 and IL-15α	Cell proliferation and migration, phagocytosis, glycolysis, respiration	• ↑Cell proliferation, ↑migration, ↑phagocytosis by IL-15 compared to untreated.<br>• ↓Proliferation, ↓migration, ↓phagocytosis, ↓glycolysis, ↓non-mitochondrial respiration, ↓basal respiration, ↓ATP turnover, ↓respiration capacity, ↓H+ leak by NEO or CEP compared to untreated.<br>• ↓Proliferation, ↓migration, ↓phagocytosis by NEO or CEP + IL-15 compared to IL-15.	IL-15: 30 ng/ml for 24-72 h.<br>NEO: 0.3 μg/ml for 24-72 h.<br>CEP: 100 μg/ml for 24-72 h.
**[394]** Palma et al., 2024	Candidiasis caused by *Candida albicans*	*Candida albicans* infection	N-Nitroso-N-phenylhydroxylamine ammonium salt (cupferron) with antifungal activity	Cell viability	• Cupferron cytotoxicity: 83% at 400 μg/ml for 24 h, <17% at 200 μg/ml for 24 h.<br>• Cupferron concentration inducing 50% cell death (CC_{50}): 350.54 μg/ml.	Cupferron treatment:1.56-400 μg/ml.
**[395]** Murtinheira et al., 2024	Autosomal recessive ataxia of Charlevoix-Saguenay	sacsin knockout (CRISPR/Cas9)	N/A	Cytoskeletal and mitochondrial functionality, STAT3, SMAD1, ERO1, TREM2, inflammation	Vimentin filaments collapse into the juxtanuclear region in >80% of cells, displacement of mitochondrial networks with altered morphology, ↑DRP1, ↑MFN-2, ↓STAT3, ↓SMAD1, ↓ERO1, ↑TREM2, ↓TNFα, ↑IL-1β by sacsin deletion (HMC3 Sacs -/-).	
**[396]** Beretti et al., 2024	Alzheimer's disease, Viral infection (SARS-CoV-2)	human Coronavirus OC43 (HCoV-OC43) infection, LPS	N/A	Cell viability, oxidative stress, inflammation, ERK, Akt	↓Cell viability, ↑ROS, ↑CD86, ↑IL1R1, ↑IL-6, ↑pERK, ↑pAkt by HCoV-OC43 infection.	SH-SY5Y, astrocytic D54MG and HMC3 cells.<br>Co-culture experiments.<br>LPS: 1 μg/ml for 24 h prior to infection/co-culture.<br>HCoV-OC43: 10^2-10^5 $TCID_{50}$.

(Continued)

10.3389/fimmu.2026.1778798

ARTICLE	MODEL	TREATMENT	DRUG TESTED	PATHWAY INVOLVED	MAIN RESULTS	NOTES
						Readouts at 1-5 days post exposure.
**[397]** Singh et al., 2024	Alzheimer's disease	LPS + ATP	Ferulic acid-piperazine derivative 13a	NLRP3 inflammasome	↓NLRP3, ↓mitochondrial ROS, ↑ΔΨm by 13a + LPS + ATP compared to LPS + ATP.	*In vivo:* Drosophila and mice AD models.<br>*In vitro:* PC12 and HMC3 cells.
**[398]** Gupta et al., 2024	Neuroinflammation induced by radiation therapy	X-ray irradiation, iPSC-derived mesenchymal stromal cells (iMSC) CM (secretome), human monocytes THP1 co-culture	N/A	THP1-HMC3 crosstalk, NF-κB	↓NF-κB by irradiated HMC3 co-cultured with THP1	iMSC cells, THP1 monocytes, hCMEC/D3 endothelial cells, and HMC3 cells.<br>Irradiaton at 0-5 Gy.<br>NF-κB activation in THP1-NFκB-Luc2 cells was significantly increased when they were cocultured with HMC3 cells irradiated at 5 Gy in RPMI media, as opposed to when they were cocultured with nonirradiated HMC3 cells.
**[399]** Li et al., 2024	Viral infection (Echovirus 30 (E30)-induced brain injury)	E30 infection	N/A	Inflammation, response to viral infection	• E30 excibited efficient replication in HMC3 cells, with titers ranging from $10^{2.5}$ to $10^{5.5}$ $TCID_{50}$ (2 to 48 h).<br>↑1284 genes and ↓831 genes, with substantial elevation of IL6, IL1A, CXCL8, CXCK1, NLRP3, IFI16 and PLK1 following E30 infection.	*In vivo:* neonatal mouse model of E30 (IFNAR-/-).<br>E30 infection: MOI=5.
**[400]** Kopp et al., 2024	Neurodegeneration	H2O2	Incretin peptides Exendin-4 (GLP-1R agonist), Twincretin and LY329 (GLP-1R/GIPR dual agonists), Triagonist (GLP-1R/GIPR/GcgR triple agonist)	Cell viability, oxidative stress	• ↑Cell viability by Twincretin and LY329 at 10 nM 48 h.<br>• ↑Cell viability by Exendin-4, Twincretin, LY329 and Triagonist at 10 nM 24 h + H2O2 400 µM 24 h.<br>↓ROS by Twincretin, LY329 and Triagonist at 100 nM 2 h + H2O2 150 µM 1 h.	SH-SY5Y, mouse microglial IMG and human microglial HMC3 cell lines.<br>Exendin 4, Twincretin, LY329 and Triagonist treatments: 10 nM for 48 h or 10 nM for 24 h followed by H2O2 400 µM for 24 h or 100 nM for 2 h followed by H2O2 150 µM for 1 h.
**[401]** Rana et al., 2024	Brain glucose deprivation	glucose deprivation	β-hydroxybutyrate (BHP)	Metabolic activity, mitochondrial functionality, oxidative and nitrosative stress, inflammation, phagocytosis	• ↓Metabolic activity, impaired mitochondrial staining, ↓Superoxide, ↓nitrite, ↑Nox2 mRNA, ↓NOX2 protein, ↓Tnf mRNA, ↓TNF, ↑Il1β mRNA, ↓IL-1β, ↓Phagocytic activity by glucose deprivation.<br>↑BDH1, ↑metabolic activity, ↑mitochondrial staining, ↑Superoxide, ↑IL-1β, ↑phagocytosis by BHB during glucose deprivation.	Glucose deprivation: glucose-free DMEM for 24 h.<br>Controls in high-glucose DMEM (25 mM) for 24 h.<br>BHB: 5 mM for 24 h.

(Continued)

10.3389/fimmu.2026.1778798

SUPPLEMENTARY TABLE 1 Continued

ARTICLE	MODEL	TREATMENT	DRUG TESTED	PATHWAY INVOLVED	MAIN RESULTS	NOTES
**[402]** Moon et al., 2024	AMD and DR	rAAV2-sVEGFRv-1 (encodes a truncated variant of the alternatively spliced soluble version of VEGFR1), rAAV2-GFP (control vector), placental growth factor (PIGF)	N/A	VEGFR1, inflammation	• ↑IL-1β, ↑IL-6 by PIGF (after rAAV2-GFP).<br>↓IL-1β, ↓IL-6 by rAAV2-sVEGFRv-1 compared to PIGF + rAAV2-GFP.	HUVECs and HMC3 cells. Treatment: rAAV2-sVEGFRv-1 or rAAV2-GFP at 25,000 MOI 48 h, then serum-starvation 24 h, followed by PIGF 300 ng/ml 4 h.
**[403]** Marino et al., 2024	Neuroinflammation	reduced glutathione (GSH), LPS	sodium L-ascorbate + 2-phospho-L-ascorbate (ASC), JS0208MD botanical antioxidant extract	Cell viability, SVCT2, NF-κB, inflammation	• ↓Cell viability, ↑SVCT2, ↑NF-κB, ↑IL-6, ↑IL-1β by LPS.<br>• ↑Cell viability, ↓NF-κB, ↓IL-6, ↓IL-1β by ASC or GSH or JS0208MD + LPS compared to LPS.<br>• ↓SVCT2 by ASC or JS0208MD + LPS compared to LPS.	*In vivo:* two separate genetically engineered mouse lines that differed in their expression of SVCT2.<br>*In vitro:* primary microglial cells derived from SVCT2-heterozygous and transgenic animals, and HMC3 cells.<br>HMC3 treated with LPS 1 μg/ml ± antioxidants for 24 h.<br>ASC: sodium L-ascorbate 10 μM + 2-phospho-L-ascorbate 100 μM.<br>GSH: 10 mM.<br>JS0208MD: 100 μM ascorbate content.
**[404]** Przepiorska-Dronska et al., 2024	Neuroinflammation (Ischemic stroke and perinatal asphyxia)	hypoxia or ischemia followed by reoxygenation	amorfrutin B	Inflammation, microglial activation, mitochondrial functionality, apoptosis	• ↑IBA1, ↑mitochondrial membrane potential, ↑caspase-1 activity, ↑TNFA mRNA, ↓IL-10 protein, ↓PPARγ protein, ↓PGC1α protein, ↑BCL2 mRNA by hypoxia or ischemia.<br>• ↓IBA1, ↓mitochondrial membrane potential, ↓caspase-1 activity, ↓TNFA mRNA, ↓IL-1β protein, ↑IL-10 protein, ↑PPARγ protein, ↑PCG1α protein, ↓BCL2 mRNA by Amorfrutin B + hypoxia or ischemia compared to hypoxia or ischemia.	hypoxia (6 h, DMEM 4.5 g/l glucose, 1% FBS) or ischemia (6 h, glucose-free DMEM, 1% FBS), then 18 h reoxygenation (DMEM 4.5 g/l glucose, 5% FBS); alternatively, hypoxia or ischemia 2 h, then reoxygenation 5h.<br>Amorfrutin B (during reoxygenation): 1 or 5 μM for 18 h.
**[405]** Haddad et al., 2024	Viral infection (ZIKV)	N/A	Dermaseptin S4 analogs (K4K20S4, K4K20K27S4, K4S4(1-16)), dermaseptin B2, dermaseptin B2 derivative K3K4B2	Cytotoxicity, viral infection	• S4 analogs inhibited Zika virus (K4K20S4: ~ 90% reduction at 3 μg/ml, K4K20K27S4: ~ 90% reduction at 6.25 μg/ml, K4S4(1-16): ~ 80% reduction at 12.5 μg/ml).<br>• K3K4B2 did non inhibit ZIKV, while B2 increased infection at 50 μg/ml.<br>• Concentration of the peptide which causes 50% cytotoxicity of HMC3 cells (CC_{50}): K4K20S4 20.89 μg/ml, K4K20K27S4 33.60 μg/ml, K4S4(1-16) 37.30 μg/ml. Concentration of the peptide which induces 50% reduction of HMC3 cells infected by ZIKV (IC_{50}):	*In vitro:* HeLa and HMC3 cell lines.<br>Dermaseptin peptides and analogs were incubated with ZKV (MOI 1.5) for 1 h at 37° C, then the mix (peptide +virus) was addet to HMC3 for 1.5 h AT 37°C.

(Continued)

10.3389/fimmu.2026.1778798

SUPPLEMENTARY TABLE 1 Continued

ARTICLE	MODEL	TREATMENT	DRUG TESTED	PATHWAY INVOLVED	MAIN RESULTS	NOTES
					K4K20S4 0.42 μg/ml, K4K20K27S4 0.82 μg/ml, K4S4(1-16) 21.71 μg/ml.	
[406]<br>Cao et al., 2024	Uveitis	PVP-CUR nanoparticles (curcumin (CUR) conjugated with polyvinylpyrrolidone (PVP)), LPS	N/A	Inflammation, microglial polarization	• ↑CD86, ↑TNFα, ↑IL-1β, ↑IL-6, ↑COX-2, ↓CD206, ↓IL-10, ↓TGF-β1, induced amoeboid morphology by LPS treatment.<br>• ↓CD86, ↓TNFα, ↓IL-1β, ↓IL-6, ↓COX-2, ↑CD206, ↑IL-10, ↑TGF-β1, alleviated amoeboid morphology by PVP-CUR + LPS cotreatment compared to LPS treatment.	*In vivo:* female Lewis rats.<br>*In vitro:* ARPE-19 and HMC3.<br>PVP-CUR nanoparticles: 1, 2.5, 5 μg/ml for 24 h.<br>LPS: 1 μg/ml for 24 h.
[407]<br>Si et al., 2024	Cancer<br>(Glioma hypoxia)	Hypoxia, si-HIF-1α, si-IL-1β	N/A	HIF-1α, inflammation	• ↑HIF-1α, ↑IL-1β, ↑TNFα, ↑IL-1α, ↑IL-8 by hypoxia. ↓IL-1β by si-HIF-1α + hypoxia compared to hypoxia.	*In vivo:* four-week-old BALB/c male nude mice.<br>*In vitro:* U251 and U87 glioma cells, HMC3 cells.<br>Glioma cells were cultured with HMC3 CM.<br>Hypoxia treatment: 1% O_2 for 24 h.
[408]<br>Cui et al., 2024	Neuropathic pain<br>(NP)	LPS	Beta-sitosterol (active component of *Lingusticum chuanxiong Hort.* (LCH))	PI3K, AKT, mTOR, inflammation, polarization	• ↑iNOS, ↑CD86, ↑COX-2, ↑TNFα, ↑IL-6, ↑IL-1β, ↑SRC, ↑BCL2, ↑HSP90AA1, ↑p-AKT, ↑p-PI3K p85α, ↑p-mTOR by LPS.<br>• ↓iNOS, ↓CD86, ↓COX-2, ↓TNFα, ↓IL-6, ↓IL-1β, ↓SRC, ↓BCL2, ↓HSP90AA1↓p-AKT, ↓p-PI3K p85α, ↓p-mTOR by β-sitosterol + LPS compared to LPS.<br>• No cytotoxicity by β-sitosterol up to 60 μM.	LPS: 100 ng/ml for 24 h.<br>Beta-sitosterol: 30 or 60 μM for 24 h.
[409]<br>Mannino et al., 2024	Neuroinflammation<br>(Hypovolemic shock)	LPS	Levosimendan (cardioprotective inodilator), dobutamine (β1-adrenergic agonist)	Nrf2, NF-κB, NLRP3, inflammation	• ↑ROS, ↓Nrf2, ↓HO-1, ↑p-NF-κB, ↑NLRP3, ↑caspase-1, ↑IL-1β, ↑TNFα, ↑IL-6, ↓IL-10 by LPS.<br>• ↓ROS, ↑Nrf2, ↑HO-1, ↓p-NF-κB, ↓NLRP3, ↓caspase-1, ↓IL-1β, ↓TNFα, ↓IL-6, ↑IL-10 by levosimendan or dobutamine + LPS compared to LPS.<br>Levosimendan and dobutamine did not affect cell viability at tested doses.	LPS: 0.1 μg/ml for 24 h.<br>Levosimendan: 10 μM for 24 h.<br>Dobutamine: 50 μM for 24 h.
[410]<br>Polini et al., 2024	Neurodegenerative diseases,<br>Cancer<br>(GBM)	FD22a (CB2R bitopic/dualsteric ligand), Aβ25-35, SR144528 (CB2R selective antagonist)	N/A	Inflammation, activation, NF-κB, mTOR	• ↓cell viability, ↑TNFα, ↑IL-6, ↓LC3, ↓SIRT1, ↓SIRT6, ↑MCP, ↑NF-κB, ↑mTOR, ↑Sigmar1, ↑SIRT5 by Aβ25-35.<br>• ↑cell viability, ↓TNFα, ↓IL-6, ↑IL-10, ↑LC3, ↑SIRT1, ↑SIRT6, ↓MCP, ↓NF-κB, ↓mTOR, ↓Sigmar1, ↓SIRT5 by FD22a pretreatment + Aβ25-35 compared to Aβ25-35.<br>• Co-administration of SR144528 almost completely abolished FD22a protective effects against Aβ25-35-induced microglial activation.	GBM U87-MG and microglial HMC3 cells.<br>FD22a: 0.1-20 μM for 24 h.<br>Aβ25-35: 1 or 10 μM for 48 h.<br>SR144528: 1 μM
[411]<br>Wu et al., 2024	Retinal barrier dysfunction	Lipopolysaccharide (LPS), interleukin (IL)-4, Nothch-1	N/A	Jagged1-Notch1, microglial polarization	• ↑iNOS, ↑IL-1β by LPS.<br>• ↑CD206, ↑Arg-1 by IL-4.	HRMECs and HMC3 cells.

(Continued)

10.3389/fimmu.2026.1778798

SUPPLEMENTARY TABLE 1 Continued

ARTICLE	MODEL	TREATMENT	DRUG TESTED	PATHWAY INVOLVED	MAIN RESULTS	NOTES
		inhibitor, RNAi targeting Jagged1			M1 polarization blocked by Notch 1 inhibitor or RNAi targeting Jagged1.	
**[412]** Ahat et al., 2024	Neurodegeneration	SHIP1 overexpression (oeSHIP1)	N/A	SHIP1, TREM2	• No PI(3,4)P2 or PI(3,4,5)P3 were detected in wild-type or oeSHIP1 HMC3.<br>• ↑Total PI, ↓monophosphorylated and bisphosphorylated PIP (PI(3)P1, PI(4)P1, and PI(4,5)P2) by oeSHIP1 compared to wild-type HMC3.<br>• ↑TLR4, ↑RPTOR, ↓AKT1 in HMC3 compared to BV2.<br>TREM2 was detected in BV2 but not in HMC3, whereas SHIP1 levels were comparable between them.	*In vitro:* mouse primary microglia, BV2 and HMC3 cells.<br>Transcriptomic, proteomic and lipidomic analyses.
**[413]** Ma et al., 2024	Sepsis-associated encephalopathy (SAE)	Mesenchymal stem cells-derived exosomes (MSCs-exo), MSCs-exo transfected with miR-140-3p mimics (Exo), S-lactoylglutathione, LPS	N/A	HMGB1, NLRP3, pyrroptosis	• ↓miR-340-3p, ↑HMGB1, ↑iNOS, ↑IL-1β, ↑IL-6, ↑TNFα, ↑p-p65/p65, ↑NLRP3, by LPS.<br>• ↑miR-340-3p, ↓HMGB1, ↓IL-1β, ↓IL-6, ↓iNOS, ↓TNFα, ↓p-p65/p65, ↓NLRP3 by MSCs-exo or Exo in LPS-induced HMC3.<br>↑GSH, ↓LD, ↓HMGB1, ↓IL-1β, ↓IL-6, ↓iNOS, ↓TNFα, ↓NLRP3, ↓caspase 1, ↓p-p65/p65, ↓GSDMD-N, ↓GLO2 by Exo or S-lactoylglutathione or Exo + S-lactoylglutathione in LPS-induced HMC3.	*In vivo:* C57BL/6J mice.<br>LPS: 100 ng/ml for 4 h.<br>MCSs-exo: 1 μg/ml for 4 h.<br>S-lactoylglutathione: 10 mM for 4 h.<br>MSC-exo + miR-140-3p mimics: MSCs-exo transfected with miR-140-3p mimics 1 μg/ml.
**[414]** Munan et al., 2024	Neuroinflammation, Viral infection (SARS-CoV-2)	LPS, IFN-γ, PDM-NO (NO selective fluorescent probe), L-NAME (iNOS inhibitor), SARS-CoV-2 RNA transfection	N/A	iNOS, inflammation, SARS-CoV-2 infection	• PDM-NO specifically shows fluorescence with NO only in lysosomal pH 4.5-5.5 and colocalizes with lysosomes in activated HMC3.<br>• ↑Endogenous NO after LPS or LPS + IFN-γ.<br>• NO signal is suppressed by L-NAME.<br>↑IL-1β, ↑TNFα, ↑lysosomal PDM-NO signal by SARS-CoV-2 RNA transfection.	Development of lysosome-specific NO probes to monitor phagocytosis in microglia during COVID-19 infection.<br>Microglial activation with LPS 500 ng/ml or LPS 500 ng/ml + IFN-γ 20 ng/ml for 24 h.
**[415]** Zhang et al., 2024	Neuroinflammation, neurodegenerative disorders	$PM_{2.5}$, ROS scavenger acetylcysteine (NAC), BAY11-7085 (NF-κB inhibitor, TLR4 neutralizing antibody	N/A	Oxidative stress, inflammation, NF-κB, TLR4	• ↑IL-6, ↑COX-2, ↓BDNF, ↑ROS, ↑TLR4, ↑NF-κB activation by $PM_{2.5}$.<br>• ↓IL-6, ↓COX-2, ↓TLR4 expression, ↓NF-κB activation by NAC pre-treatment + $PM_{2.5}$.<br>↓IL-6, ↓COX-2 by BAY11-785 or TLR4 neutralizing antibody pre-treatment + $PM_{2.5}$.	$PM_{2.5}$: 12.5, 25, 50 mg/l for 24 h.<br>NAC: 5 mM for 3 h.<br>BAY11-7085: 1 μM for 2 h.<br>TLR4 neutralizing antibody: 2 mg/l for 4 h.
**[416]** Hu et al., 2024	Viral infection (Hand, food and mouth disease, HFMD)	Coxsackievirus-A10 (CV-A10) infection, MST1/2 overexpression or knockdown	N/A	CV-A10 replication, inflammation, NF-κB, Hippo	• CV-A10 replicates in HMC3 inducing ↑IL-6 at 6 h, ↑IL-8 at 12 h, ↑IL-1β at 36 h, ↑PRR pathway proteins, ↑TBK1, ↑IRAK1 and ↑NF-κB with nuclear translocation.<br>• ↑IL-1β, ↑IL-6, ↑TNFα, ↑TBK1, ↑IRAK1, ↑NF-κB by MST1/2 knockdown + CV-A10 compared to CV-A10.<br>• ↓IL-1β, ↓IL-6, ↓TNFα, ↓TBK1, ↓IRAK1, ↓NF-κB by MST1/2 overexpression + CV-A10 compared to CV-A10.<br>•	CV-A10 infection: MOI 0.1 for 2 h.<br>The CV-A10 (subgenotype C) was isolated during an epidemic in Xiangyang (China) in 2017 and, for this study, proliferated in Vero cells.

(Continued)

10.3389/fimmu.2026.1778798

SUPPLEMENTARY TABLE 1 Continued

ARTICLE	MODEL	TREATMENT	DRUG TESTED	PATHWAY INVOLVED	MAIN RESULTS	NOTES
**[417]** Ramakrishnan et al., 2024	Alzheimer's disease	Aβ1-42-APC, TREM2 R27H variant, TREM2-knockout (KO), apoptotic neurons	K161 (pan-SHIP1/2 inhibitor), 3AC (SHIP1 selective inhibitor), Dox (SHIP1 overexpression by inducible vector), oligomycin (mitochondrial electron transport chain inhibitor), FCCP (mitochondrial uncoupling agent), rotenone/antimycin A (electron transport chain inhibitors)	TREM2, SHIP1, phagocytosis	• ↓Aβ phagocytosis, ↓lysosomal capacity, ↓OCR, ↓ATP, ↓SRC by TREM2 KO or R47H.<br>• ↑Apoptotic neurons phagocytosis by R47H.<br>• ↓Aβ phagocytosis, ↓lysosomal capacity, ↓OCR, ↓ATP, ↓SRC by SHIP1 expression in TREM2 expressing cells but not in TREM2 KO cells.<br>• ↑Aβ phagocytosis, ↑lysosomal capacity by K161 independently of TREM2 or SHIP1 expression.<br>• ↓Apoptotic neurons phagocytosis, ↑OCR, ↑ATP, ↑SRC by K161 in TREM2 KO cells.	Aβ1-42-APC treatment for 8 h.<br>K161: 1, 2,5 or 5 μM for 1 or 16 h.<br>3AC: 5 μM for 16 h.<br>Dox: 1 μg/ml in cell media.<br>Oligomycin (2.5 μM), FCCP (1 μM) and rotenone/ antimycin A (0.5 μM) were injected sequentially during mitochondrial activity assessment.<br>Apoptosis was induced in N2A neurons by 0.5 μM Staurosporine for 16 h.
**[418]** Ji et al., 2024	Immunotherapy	LPS/IFN-γ for M1, IL-4 for M2	N/A	PTPN7 (a phosphatase proposed as a therapeutic target), MAPK/ERK and JAK/STAT signaling pathways	• ↑ PTPN7 in M2-like tumor-associated macrophages/ microglia;<br>↓ PTPN7 restores an M1 immune profile and enhances anti-tumor immunity;<br>PTPN7 correlates with poor prognosis and reduced PD-1 immunotherapy response.	N/A
**[419]** Mahajan et al., 2024	Neuroinflammation (Cerebral ischemia)	OGD, hypernatremia, hyperchloremia	N/A	Apoptosis (Caspase-3 activation), NF-κB inflammatory signaling, SOD and GPx activities	Hypernatremia and hyperchloremia reduced survival of HMC3 and SH-SY5Y cells after OGD/R:<br>↑ ROS and mitochondrial depolarization;<br>↑ apoptosis and neuroinflammation.<br>Elevated electrolytes worsen ischemic brain injury, suggesting caution with hypertonic solutions during ischemia/reperfusion.	co-culture of HMC3 human microglial cells and SH-SY5Y neurons.<br>Hypernatremia: elevated Na^+, 160–180 mM<br>Hyperchloremia: elevated Cl^-, 140–150 mM
**[420]** Verma et al., 2024	Neuroinflammation	*P. gingivalis* LPS	N/A	TLR4/MyD88/NF-κB, oxidative stress, synaptic, and mitochondrial pathways.	Pg-LPS strongly activates inflammatory signaling in HMC3;<br>Activated HMC3 release soluble factors that impair SH-SY5Y metabolism and viability;<br>Identified key deregulated proteins: HSP70, VDAC1, NDUFS3, GFAP.	*P. gingivalis* LPS (100–500 ng/ mL for 24 h).
**[421]** Chen et al., 2024	Neuroinflammation	LPS	Lipofundin®, a clinically used parenteral nutrition lipid emulsion tested for anti-inflammatory properties	Inflammatory processes	Lipofundin:<br>↓ neuroinflammatory activation in LPS-stimulated Lipofundin reduces neuroinflammatory activation in LPS-stimulated HMC3.<br>- Restores balance between pro- and anti-inflammatory mediators.<br>- Effects are dose-dependent and non-cytotoxic.HMC3;<br>↺ balance between pro- and anti-inflammatory mediators;	LPS (100 ng/mL)

(Continued)

10.3389/fimmu.2026.1778798

SUPPLEMENTARY TABLE 1 Continued

ARTICLE	MODEL	TREATMENT	DRUG TESTED	PATHWAY INVOLVED	MAIN RESULTS	NOTES
					Effects are dose-dependent and non-cytotoxic.<br>Lipofundin exhibits anti-neuroinflammatory activity and may serve as an adjuvant in chronic neuroinflammatory conditions	
**[422]** Kocanci et al., 2024	Neuroinflammation	LPS, H2O2	pimecrolimus, a topical calcineurin inhibitor with anti-inflammatory and neuroprotective properties.	Inflammatory processes	Pimecrolimus attenuates microglial activation and the neurotoxic profile of HMC3 secretome.<br>In SH-SY5Y neurons: ↑ viability, ↓ ROS, ↓ apoptosis.<br>Combined anti-inflammatory and antioxidant effects observed.	HMC3 cells and neuron-like SH-SY5Y cells (differentiated).<br>LPS 100 ng/mL<br>H2O2 100 μM
**[423]** Kocanci et al., 2024	Neuroinflammation	LPS	Tacrolimus (FK506)	NF-κB, IL-6/TNFα/ROS, inflammation.	Tacrolimus inhibits the NF-κB pathway, blocking p65 nuclear translocation.<br>↓ expression of iNOS and COX-2.<br>↓ release of TNFα, IL-6, and IL-1β.<br>↑ antioxidant defense via Nrf2/HO-1 activation and increased SOD and GPx activity	LPS 100 ng/mL
**[424]** Chevalieret al. 2024	Neuroinflammation (Ischemia-induced)	LPS	CBD encapsulated in a biodegradable polymer-based sustained-release formulation	ROS and inflammatory pathways	In HMC3: ↓ pro-inflammatory cytokines and ROS, ↑ cell survival.<br>In ischemic mice: ↓ infarct volume, ↓ microglial activation, ↑ neuronal protection.<br>Polymeric formulation provided sustained release and greater efficacy than free CBD.<br>Polymeric CBD downregulates inflammation via NF-κB inhibition and PPARγ activation.<br>↓ expression of IL-1β, TNFα, iNOS, COX-2.<br>↑ antioxidant response through Nrf2/HO-1 activation.<br>Improves mitochondrial metabolism and reduces ROS generation.	*In vivo:* mouse model of cerebral ischemia (MCAO) treated with OGD<br>LPS 100 ng/mL
**[425]** Scordinio et al., 2024	Neurinflammation	H2O2	Grapefruit IntegroPectin, a natural pectin rich in flavonoids (naringin, hesperidin) and limonoids, extracted from grapefruit using ultrasound-assisted cavitation.	Antioxidant and anti-inflammatory pathways	IntegroPectin protects HMC3 cells from H2O2-induced oxidative stress.<br>↓inflammation, apoptosis, and mitochondrial damage.<br>↑cell viability and redox balance.<br>- Antioxidant action: ↓ ROS generation and restored mitochondrial function.<br>- Anti-inflammatory effect: inhibition of NF-κB pathway and reduced expression of IL-6, TNFα, iNOS, and COX-2.<br>- Anti-apoptotic effect: ↑ Bcl-2, ↓ cleaved caspase-3.	H2O2 200 μM
**[426]** Goksu et al., 2024	Neurodegeneration	LPS	VIP	NF-κB, TNFα, IL-1β, and NO.	↓reduction neurotoxicity and lower inflammatory cytokine release in HMC3 cells<br>Co-cultured SH-SY5Y neurons display higher survival, less apoptosis, and preserved synaptic activity.<br>VIP binds VPAC1/VPAC2 receptors, inhibiting the NF-κB pathway and decreasing secretion of TNFα, IL-1β,	LPS 100 ng/mL

(Continued)

10.3389/fimmu.2026.1778798

SUPPLEMENTARY TABLE 1 Continued

ARTICLE	MODEL	TREATMENT	DRUG TESTED	PATHWAY INVOLVED	MAIN RESULTS	NOTES
					and NO.<br>- ↑ Expression of IL-10 and BDNF (neurotrophic/anti-inflammatory factors).<br>- Shifts microglia phenotype from M1 (pro-inflammatory) to M2 (neuroprotective).	
**[427]**<br>Van et al., 2024	Neurodegeneration	LPS	amlexanox (TBK1 and IKKε kinases inhibitor)	Viability, NF-κB, inflammation	↑ cell viability and reduces oxidative stress.<br>↓ both NF-κB and STAT3 signaling pathways.<br>Amlexanox inhibits NF-κB p65 and STAT3 phosphorylation, suppressing transcription of inflammatory genes.<br>↓ Expression of IL-1β, IL-6, TNFα, COX-2, and iNOS.<br>↓ Production of ROS and NO.<br>↑ HO-1 and Nrf2 activation, enhancing antioxidant defense.	LPS 100 ng/mL
**[428]**<br>Zhang et al., 2024	Neuroinflammation (Ischemic injury)	overexpression or silencing of TRIM59	N/A	Inflammation, pyroptosis.	TRIM59 overexpression protects HMC3 from OGD/R-induced ischemic injury.<br>↓ pyroptosis (↓GSDMD-N and cleaved caspase-1).<br>In MCAO/R mice: smaller infarct size and decreased microglial activation.<br>TRIM59 mediates ubiquitination and degradation of NLRP3.<br>↓ activation of the NLRP3 inflammasome, ↓ caspase-1 activation, ↓ IL-1β and IL-18 secretion.<br>Suppression of microglial pyroptosis and inflammatory response	*In vivo:* mouse model of cerebral ischemia/reperfusion (MCAO/R) with overexpression or silencing of TRIM59<br>TRIM59 acts as a negative regulator of the NLRP3 pathway, providing neuroprotection against ischemic injury by inhibiting microglial pyroptosis.
**[429]**<br>Steinmaurer et al., 2024	Neuroinflammation	N/A	ibrutinib (Bruton's Tyrosine Kinase (BTK) inhibitor)	NLRP3 and M1 polaritazion	↑ BTK expression and phosphorylation in microglia/macrophages by intracellular iron accumulation<br>↑ M1 pro-inflammatory polarization, ↑ IL-1β, TNFα, and ROS by BTK activation.<br>↓ NLRP3, IL-1β, and ferro-lipid peroxidation, indicating a link to ferroptosis regulation by BTK inhibition	Post-mortem human brain tissue fromMS patients and healthy controls.<br>*In vitro:* HMC3 cells and monocyte-derived macrophages to assess ibrutinib, 1–5 µM)
**[430]**<br>Yang et al., 2024	Neuroinflammation	homocysteine	Betaine (1–5 mM)	pyroptosis,<br>m6A methylation and	Betaine suppresses homocysteine-induced pyroptosis and microglial activation in HMC3 cells.<br>YTHDF2 expression, promoting NLRP3 mRNA degradation.<br>↑ Nrf2/HO-1 signaling, ↓ ROS and oxidative stress	*In vivo:* mouse of hyperhomocysteinemia homocysteine 200 µM
**[431]**<br>Deng et al., 2024	Neuroinflammation	Zymosan	anti-CCL2 antibody, CCR2 antagonist, or Notch2 inhibition using DAPT (γ-secretase inhibitor)	CCL2,<br>(IL-1β, TNFα).<br>Notch2/Jagged1	The chronic pelvic pain model exhibited enhanced mechanical and thermal hypersensitivity.<br>- CCL2 and Notch2 were upregulated in the dorsal horn of the spinal cord.<br>- Anti-CCL2, CCR2 blockade, or Notch2 inhibition	*In vivo:* chronic pelvic pain rat (male Sprague–Dawley rats) induced by intraprostatic zymosan injection<br>Zymosan (1 mg in 50 µL)

(Continued)

10.3389/fimmu.2026.1778798

SUPPLEMENTARY TABLE 1 Continued

ARTICLE	MODEL	TREATMENT	DRUG TESTED	PATHWAY INVOLVED	MAIN RESULTS	NOTES
					significantly reduced microglial activation and central sensitization	
**[432]** Chen et al., 2024	Neuroinflammation	LPS	dapagliflozin	NF-κB, IL-1β/IL-6	↑Estrous cyclicity and ovulatory capacity in HFD-fed mice.<br>↓Microglial activation and hypothalamic inflammation.<br>↻ Reproductive hormone secretion and energy metabolism.<br>↓ LPS-induced inflammation in HMC3 cells	*In vivo:*models: female C57BL/6 mice fed a HFD for 16 weeks.<br>LPS (100 ng/mL)
**[433]** Huang et al., 2024	Cancer (GBM)	N/A	DOX encapsulated in hybrid cell membrane-coated supramolecular micelles (H-GM@DOX).	tumor microenvironment targeting → endocytosis → DOX release → GBM apoptosis (↓ Bcl-2, ↑ caspase-3)	H-GM@DOX micelles show high brain permeability, tumor-specific targeting, and controlled drug release. greater cytotoxicity against GBM cells compared to free DOX.	Hybrid membranes = fusion of microglial (HMC3) and tumor (U87MG) membranes for improved targeting and immune evasion.
**[434]** Ou et al., 2024	Neuroinflammation	Intermittent hypoxia	HDAC6 inhibitor (Tubastatin A) or TDP43 siRNA.	Intermittent hypoxia → ↑ TDP43 → ↑ HDAC6 → ↓ Prdx1 → ↑ ROS + microglial inflammation → cognitive impairment and the opposite effect of Tubastatin A/siTDP43 → ↓ inflammation → improved cognition.	OSA mice showed significant cognitive deficits (spatial memory and maze tests).<br>↑ Expression of TDP43, HDAC6, and dysfunctional Prdx1 in cortex and hippocampus.<br>Tubastatin A and TDP43 siRNA improved cognitive function and reduced activated microglia and inflammatory cytokines	*In vivo:* exposure to intermittent hypoxia cycles (21%–5% O_2) to mimic OSA.
**[435]** Gopalakrishnan et al., 2024	Neuroinflammation	LPS	α-Gal nanoparticles (α-Gal NP, 10–100 μg/mL) for 24–48 hours	α-Gal NP → binding with anti-Gal antibodies → microglial FcγR activation → M2 polarization (↑ IL-10, TGF-β) → ↓ inflammation/↑ neuroregeneration.	α-Gal NP interact with anti-Gal antibodies, which bind to Fcγ receptors on microglia, driving a shift toward the M2 pro-healing phenotype.<br>↑ Expression of IL-10, Arg-1, TGF-β, and CD206.<br>↓ Expression of IL-1β, TNFα, iNOS, and M1 markers.<br>↑ Secretion of neurotrophic factors (BDNF, NGF)	LPS 100 ng/mL
**[435]** Peltier et al., 2024	Neuroinflammation (ASD)	VPA	N/A	, impairing synapse formation and myelination. - oxidative stress and inflammatory cytokines	VPA-treated HMC3 cells exhibited enhanced pro-inflammatory activation and impaired lipid metabolism.	*In vivo:* rat model induced by VPA administered to pregnant female rats (gestational day 12.5). sodium valproate 600 mg/kg i.p.<br>*In vitro:* studies using human microglial HMC3 cells and cortical neurons exposed to VPA(1–5 mM) exposure for 24 h
**[436]** Yin et al., 2024	Neuroinflammation, Cancer (GBM)	AMD3100 or ABCF1 silencing	N/A	ABCF1 ↑ → CXCL12 ↑ → CXCR4 → PI3K/AKT	↑ ABCF1 expression correlates with poor prognosis in GBM patients.<br>ABCF1–CXCL12–CXCR4 axis drives GBM growth and	*In vitro:* human GBM cell lines U87 and U251.<br>Co-culture with HMC3 cells

(Continued)

10.3389/fimmu.2026.1778798

10.3389/fimmu.2026.1778798

SUPPLEMENTARY TABLE 1 Continued

ARTICLE	MODEL	TREATMENT	DRUG TESTED	PATHWAY INVOLVED	MAIN RESULTS	NOTES
					invasiveness.<br>↓ AKT phosphorylation, migration, and colony formation by AMD3100 or ABCF1 silencing.	*In Vivo:* orthotopic GBM mouse model.<br>Treatment with CXCL12 (SDF-1, 100 ng/mL) and CXCR4 inhibitor AMD3100 (10 µM) to prove the pathway
**[437]**<br>Chen et al., 2024	Neuroinflammation (SCI)	LPS, Htr2b modulators	N/A	Htr2b ↑ → ↓ NRG1/ErbB → ↑ M1 (IL-1β, TNFα) → neuroinflammation/↓ regeneration.<br>Htr2b antagonist (SB204741) → ↑ NRG1/ErbB → ↑ M2 (IL-10, Arg1) → neuroprotection and recovery.	After SCI, Htr2b is markedly upregulated in activated microglia.<br>Htr2b activation drives M1 polarization, exacerbating neuroinflammation and axonal damage.<br>Htr2b blockade (SB204741) reduces inflammation, enhances M2 microglial profile, and improves motor recovery.	*In vivo:* SCI mice treated with Htr2b agonist (BW723C86) or Htr2b antagonist (SB204741).<br>LPS (100 ng/mL)
**[438]**<br>Yi et al., 2024	Neuroinflammation (Ischemic injury)	OGD/R	exosomes derived from bone marrow mesenchymal stem cells or exosomes enriched with miR-148b-3p	OGD/R → ↑ DLL4/Notch1 → M1 (IL-1β, TNFα) → neuroinflammation.<br>BMSC-exo miR-148b-3p → ↓ DLL4/Notch1 → ↑ M2 (IL-10, Arg1) → neuroprotection.	BMSC-miR-148b-3p exosomes markedly reduced OGD/R-induced microglial activation and inflammation.<br>↓ Notch1, DLL4, IL-1β, TNFα; ↑ IL-10 and Arg1.<br>Protected microglial cells and prevented secondary ischemic injury.	OGD/R model (for 6 hours + reperfusion for 24 hours to induce ischemic stress)<br>Control group treated with miR-148b-3p inhibitor to confirm specificity
**[439]**<br>Mutlu et al., 2024	Imaging	N/A	N/A	Optical absorption → heating → thermoelastic expansion → acoustic wave; spectral features reflect cell size	• - Correlation between spectral slope and cell size<br>• - spectral analysis (PASA) parameters discriminate cell types based on size<br>•	HMC3 and EoL-1 (eosinophilic leukemia)
**[440]**<br>Guerrero-González et al., 2024	Neuroinflammation	Imidazo[1,2-α] azine derivatives with acidic groups at position 2, including imidazo[1,2-α] pyridines and imidazo[1,2-α] pyrimidines	N/A	COX/mPGES axis	• ↓ PGE_2 levels in both HMC3 and SH-SY5Y cells.<br>↓ PGE_2 in HMC3 microglia by two selected esters<br>• ↑ selectivity and anti-inflammatory activity in microglia by Imidazo[1,2-α]azine esters<br>•	HMC3 and human neuroblastoma SH-SY5Y cells under pro-inflammatory conditions
**[441]**<br>Alateeq et al., 2024	Neuroinflammation	CSE 10% or H2O2	apocynina(NOX2 inhibitor/ROS scavenger)	MAPK/JNK pathway	• ↓ Il6, ↓ Il1β in the amygdala (TNFα not modified)<br>• ↻ Gpx in amygdala and prefrontal cortex.<br>• In HMC-3: ↓ Il6, ↓ Tnfα, ↓ Tlr4 (and ↓ Il1β in prevention mode), plus ↓ lipid/protein oxidation,<br>• ↓ p-JNK and<br>• ↑ ERK1/2<br>• ↓ CRP e ↓ MDA (stress ossidativo)	Apocynina:<br>*In vivo*: 5 mg/kg (i.p.);<br>*In vitro*: 500 nM applied to BEAS-2B in preventive (co-treatment) or therapeutic mode before CM transfer to HMC3. HMC-3 were also exposed to CSE 10% or H2O2 for 24 h.

(Continued)

SUPPLEMENTARY TABLE 1 Continued

ARTICLE	MODEL	TREATMENT	DRUG TESTED	PATHWAY INVOLVED	MAIN RESULTS	NOTES
**[442]** Wijenayake et al., 2024	Neuroinflammation	mEVs isolated from bovine milk	N/A	DNMT1 (DNA methyltransferase 1) and TET2 (demethylation enzyme), global DNA methylation patterns	Alterations in DNMT1 and TET2 expression/activity (epigenetic modulation)	Efficient internalization of EVs by HMC3
**[443]** Owusu et al., 2024	Neuroinflammation	Aβ	Hancinone (HAN, 0.5 μM, 2.5 μM, 10 μM)	TREM2 → Syk → PI3K → AKT → mTOR	• ↑ fagocytosis<br>↓ inflammation markers expression (TREM2, CX3CR1, P2RY12)	Aβ (2.5 μM)
**[444]** Pehar et al., 2024	Neuroinflammation	Histamine, receptor antagonists of histamine (e.g., ranitidine, HRH2 blocker)	N/A	HRH2 (histamine receptor type 2)	↑ regulation cellular prion protein (PrP^C) expression	histamine at increasing concentrations (1 μM-100 μM)
**[445]** Ceyhan Hacioglu 2024	Neuroinflammation	sucralose	N/A	NLRP3 inflammasome	↓ SIRT1 → activation of NLRP3 inflammasome.<br>↑ IL-1β and other pro-inflammatory cytokines.<br>• ↓ GPx4, a key enzyme preventing lipid peroxidation<br>↑ ROS production	sucralose at increasing concentrations (0–50 mM) for 24–72 hours
**[393]** Wang et al., 2024	Neuroinflammation	IL-15	Neoprzewaquinone A (NEO) (a natural quinone compound isolated from medicinal plants)	glycolysis and OXPHOS	↓ migration<br>↓ phagocytosis<br>Altered metabolism (reduced glycolysis and mitochondrial respiration	N/A
**[446]** Mayer et al., 2024	Neuroinflammation	LPS	N/A	M1-like and NF-κB	↑ pro-inflammatory activation (IL-6 and IFN-γ)	LPS (10–100 ng/mL)
**[447]** Zhang et al., 2024	Neuroinflammation	LPS	N/A	C15orf39 → PRMT2 (Protein Arginine Methyltransferase 2) activation → IκBα stabilization → NF-κB p65 inibition	↑ pro-inflammatory activation (IL-6 and TNFα)	LPS (10–100 ng/mL)
**[448]** Lunghi et al., 2025	Neuroinflammation, Parkinson's disease	α-Syn pre-formed fibrils (PFFs) ± GM1 oligosaccharide (OligoGM1)	N/A	Microglial activation, αSyn accumulation/clearance, TNFα, IL-6	↑Iba1, ↑TREM2, ↑intracellular α-Syn, ↑TNFα and ↑IL-6 after αSyn PFFs treatment<br>↓Iba1(+) cells after αSyn PFFs + OligoGM1 pre-treatment<br>↓αSyn accumulation in TREM2(+) microglia after αSyn PFFs + OligoGM1 pre-treatment<br>↓IL-6 release after αSyn PFFs + OligoGM1 pre-treatment	OligoGM1 selectively attenuates αSyn induced activation without compromising basal immune function.<br>Treatment αSyn (1 μM, 48 h) ± Pre-treatment OligoGM1 (100 μM, 4 h)
**[449]** Ding et al., 2025	Neuroinflammation (Tuberous sclerosis complex (TSC))	TSC2 knockdown (lentiviral)	rapamycin	mTOR, NLRP3, microglial activation, ROS	↑AIF1 mRNA, ↑CD68 mRNA and ↑ROS in TSC2 KD cells<br>↑NLRP3 mRNA and ↑IL1β mRNA in TSC2 KD cells<br>↑NLRP3 and Pro-IL-1β proteins in TSC2 KD cells<br>↑Cleaved-caspase-1 and Cleaved-IL-1β proteins in the	TSC2 knockdown (TSC2 KD) HMC3 generated by lentivirus.<br>rapamycin 10 nM, 24 h.

(Continued)

10.3389/fimmu.2026.1778798

SUPPLEMENTARY TABLE 1 Continued

ARTICLE	MODEL	TREATMENT	DRUG TESTED	PATHWAY INVOLVED	MAIN RESULTS	NOTES
					supernatant of TSC2 KD cells<br>↓Microglial activation markers and ↓inflammasome after rapamycin	
**[450]**<br>Zhang et al., 2025	DR	HG, laminarin (LAM, Dectin 1 antagonist)	LAM	Microglial activation, inflammatory cytokines, BRB tight junctions, apoptosis	↑Dectin-1 expression in HG-exposed HMC3<br>↑microglial activation (IBA-1), ↑TNFα, ↑IL-1β and ↑IL-6 in HG HMC3<br>↓BRB TJ proteins (ZO-1, occludin and claudin-5) in HG HMC3<br>↓microglial activation, ↓ TNFα, ↓IL-1β, ↓IL-6, ↺BRB TJ proteins in HG HMC3 after LAM treatment	*In vivo*: diabetic C57BL/J6 male mice established by intraperioteal injection of STZ.<br>*In vitro:* HMC3 under high glucose (HG HMC3).<br>HMC3 + human retinal endothelial cells (HRECs) co-culture.<br>Control HMC3 (5.5 mM glucose), HG HMC3 (25 mM glucose, 24 and 48 h).<br>Use of mannitol (19.5 mM) as osmotic control.<br>LAM treatment 150 or 300 µg/ml.
**[451]**<br>Mendoza-Mari et al., 2025	Neuroinflammation, Traumatic brain injury (TBI)	Low frequency electromagnetic field (EMF) stimulation after TNFα exposure	N/A	NRLP3, NF-κB, ERK-MAPK, CASP1, IL-1β, IL-18, IL-6	↑NF-κB, ↑NRLP3, ↑CRASP1, ↑IL-1β, ↓IL-18, ↑IL-6 by TNFα<br>↑NF-κB (2.5 Hz), ↓NRLP3 (5 Hz), ↑CRASP1 (2.5 Hz), ↑IL-1β (2.5 Hz), ↑IL-6 (2.5 Hz) expression 24 h after EMF + TNFα compared to TNFα<br>↓NF-κB (5 Hz), ↓CRASP1 (2.5 and 5 Hz), ↓IL-1β (5 Hz), ↓IL-18 (2.5 and 5 Hz), ↓IL-6 expression (2.5 and 5 Hz) 48 h after EMF + TNFα compared to TNFα	HMC3 microglia and HCN-2 neurons.<br>TNFα treatment: 50 ng/ml, 20 min.<br>EMF stimulation: 2.5 or 5 Hz with 1 V signal intensity, 3 min.
**[452]**<br>Canseco-Rodriguez et al., 2025	Neuroinflammation	LPS	FGA139 (irreversible cysteine protease inhibitor)	Cysteine proteases (calpains/caspases/ cathepsins), TNFα, microglial polarization, zinc transport/vesicle trafficking pathways, neuroprotective extracellular metabolites (purines, linoleic acid, phenyllactic acid)	↓viability, ↑TNFα after LPS<br>↺viability, ↓TNFα after FGA139 pretreatment + LPS compared to LPS<br>↑neuroprotective extracellular metabolites (purines, linoleic acid, phenyllactic acid) in supernatant with FGA139<br>↓M1-like microglial polarization with FGA139	RAW264.7 murine macrophages, HMC3 microglia, SH SY5Y neuron like differentiated cells (retinoic acid 10 µM, 1,5% FBS, 7 days).<br>LPS treatment:.0.1 or 1 µg/ml, 48 h.<br>FGA139 pre-treatment: 1 and 3 µM, 24 h.<br>Metabolomic and proteomic analysis.
**[453]**<br>Ke et al., 2025	Neuroinflammation, (Tourette syndrome)	LPS, miR 429 mimic, miR 429 inhibitor.	N/A	IKKβ, NF-κB, IL 6	↓IKKβ, ↓NF-κB p65, ↓IL-6 in LPS-stimulated HMC3 with miR-429 mimic vs MiRNA negative control<br>↑IKKβ, ↑NF-κB p65, ↑IL-6 in LPS-stimulated HMC3 with miR-429 inhibitor vs MiRNA negative control	*In vivo:* TS Sprague-Dawley (SD) male rats established by intraperitoneal injection of IDPN (150 mg/kg/day for 7

(Continued)

10.3389/fimmu.2026.1778798

SUPPLEMENTARY TABLE 1 Continued

ARTICLE	MODEL	TREATMENT	DRUG TESTED	PATHWAY INVOLVED	MAIN RESULTS	NOTES
						successive days).<br>*In vitro:* HMC3 human microglia, HEK-293T human embryonic kidney cells.<br>LPS stimulation: 100 ng/ml.
**[454]**<br>Lai et al., 2025	Alzheimer's disease	LPS, Aβ42	ω-3 PUFAs derivatives isolated from *Prorocentrum lima*	Chemotactic migration, Aβ1-42 phagocytic clearance, viability, inflammation	↑IL-6 and ↑IL-1β in cells induced with LPS<br>No changes in IL-6 and IL-1β in cells induced with FA18:4 or FA22:6<br>↑HMC3 migration with FA20:5 butanediol ester, FA18:5, FA18:4, FA22:6, and (Z)-10-nonadecenoic acid<br>↑Aβ42 phagocytosis with FA18:4	LPS treatment:100 or 1000 ng/ml.<br>Aβ42 treatment: 1 μM.<br>ω-3 PUFAs treatment: 20 μM, 3 or 12 or 24 h.
**[455]**<br>Su et al., 2025	Neuroinflammation Hypertension	Angiotensine II (AngII), PapRIV (Pap, NF-κB activator)	anatabine	NF-κB, NLRP3, caspase 1–dependent pyroptosis, oxidative stress, cytokines (IL 1β, IL 18, TNFα, MCP 1)	↑inflammation, ↑oxidative stress, ↑inflammasome/ cytokines by AngII<br>↺inflammation, ↺oxidative stress, ↺inflammasome/ cytokines by anatabine<br>↑inflammation, ↑oxidative stress, ↑inflammasome/ cytokines by PapRIV	*In vivo:* spontaneously hypertensive (SHR) and Wistar Kyoto (WKY) rats.
**[456]**<br>Deng et al., 2025	Paracrine signaling profiling in human cells	N/A	N/A	Secreted cytokines, chemokines, EVs	Paracrine-mediated attenuation of secretory function in homotypic system Exosome secretion largely cell-number-independent<br>Microglia enhanced neuronal secretion, effect strongly dependent on paracrine cell number	THP 1 macrophages, HMC3 microglia, SH SY5Y neurons.
**[457]**<br>Tian et al., 2025	CCL11-induced cognitive impairment	genetic CCR3 modulation	Qifuyin (and its brain penetrant components)	CCL11/CCR3, microglial senescence (SA-β-Gal, p16, p21), senescence associated secretory phenotype (SASP); synaptic plasticity markers (GAP 43, PSD 95, SYN)	↑microglial senescence and SASP with CCL11<br>↺CCL11/CCR3-induced cell cycle arrest (↓G0, ↓G1), ↓p16, ↓p21, ↓SASP by Qifuyin brain components	*In vivo:* C57BL/6 mice.<br>*In vitro:* HMC3 microglia (CCR3 KO/overexpression), HT 22 neuron co-culture.
**[458]**<br>Zhu et al., 2025	Alzheimer's disease, DNA methylation	Aβ1-42 peptide	N/A	Tissue specific AD pathways, key genes (THBS1, TGFB1, HIF1A, KLF4), poly-methylation score (PMS) associated with AD phenotypes	↑KLF4 and ↓TGFB1 in Aβ42 treatment group<br>Higher PMS is correlated with faster decline in cerebral metabolic rate and cognitive function	SH-SY5Y neurons, HMC3 microglia, and THP-1 cells.<br>Aβ42 treatment: 10 μM, 24 h.<br>Identified tissue specific AD pathways and key genes.<br>.
**[459]**<br>Leung et al., 2025	Alzheimer's disease	CRISPR interference (CRISPRi) targeting EGFR regulatory element	N/A	Microglial enhancer at SEC61G locus regulating EGFR<br>Risk allele rs74504435 predicted to increase EGFR	Identified 42 candidate variant–effector gene pairs across late onset AD (LOAD) loci<br>Rs74504435 risk allele linked to increased EGFR<br>CRISPRi validated enhancer to EGFR regulation	N/A

(Continued)

10.3389/fimmu.2026.1778798

SUPPLEMENTARY TABLE 1 Continued

ARTICLE	MODEL	TREATMENT	DRUG TESTED	PATHWAY INVOLVED	MAIN RESULTS	NOTES
**[460]** Liu et al., 2025	Neuroinflammation (ICH)	Haemoglobin (hemin), monoclonal antibody SZ168 targeting Podoplanin	SZ168	NLRP3, caspase 1, GSDMD mediated pyroptosis; inflammatory cytokines IL 1β, TNFα	• ↓invasion, ↓migration, ↓proliferation, ↓NLRP3, ↓cleaved caspase-1, ↓GSDMD-N; ↓IL-1β and ↓TNFα by SZ168 dose-dependently in cells under hemin stimulation	*In vivo:* collagenase-induced ICH in C57BL/6 mice.<br>SZ168 treatment: 0-50 μg, 2 h.
**[461]** Ricardi et al., 2025	Alzheimer's disease	CB2R- and PPARγ-selective antagonists	β-Caryophyllene (BCP, CB2 receptor agonist)	NF-κB; TNF α, IL-6, IL-10, SIRT1, PGC-1α, BDNF	BCP protected against Aβ25–35 cytotoxicity<br>↓TNFα, ↓IL-6, ↑IL-10),<br>↓NF-κB<br>↺SIRT1<br>↺PGC-1α<br>↺BDNF	*Ex vivo:* Aβ1–42–treated mouse brain slices.<br>*In vitro:* Aβ25–35–stimulated (1 μM, 48 h) HMC3 microglia.<br>BCP pretreatment: 5, 10, 25 μM for 24 h.
**[462]** Sha et al., 2025	Neuroinflammation (SCI)	LPS, ELAV1, USP29, and TAK1 overexpression by lentivirus transfection (oe).<br>knock down by lentivirus transfection (sh)	N/A	ELAVL1, USP29, TAK1, M1/M2 microglial polarization	↑M1 by LPS<br>↓M1 and ↑M2 by oeUSP29<br>↑TAK1 signaling by oeELAVL1 or shUSP29	*In vivo:* SCI rat,<br>LPS treatment: 1 μg/ml for 24 h<br>.
**[463]** Costantino et al., 2025	Blood–brain barrier (BBB)	TNFα, IFNγ, metabotropic glutamate (mGlu) 2/3 receptors agonist LY379268, mGlu2 negative allosteric modulator VU6001966	LY379268, VU6001966	mGlu2, mGlu3, BBB tight junctions, cytokine/ chemokine expression, TEER/permeability	↓TEER and ↑permeability after TNFα+IFNγ in all culture settings<br>↺BBB properties (↑TEER, ↓permeability, preservation of junctional proteins) after TNFα+IFNγ+LY379268, especially in endothelial+astrocyte and triple co-cultures<br>↓microglial cytokine expression by LY379268 (effect attenuated by mGlu2)	*In vitro:* human BBB model with human derived microvascular endothelial cells (TY–10) cultured either alone or cocultured with astrocytes (hAST) or in a triple coculture with astrocytes and microglia (HMC3).<br>TNFα and IFNγ 1 ng/ml each
**[464]** Huang et al., 2025	Neuroinflammation, Epilepsy	Kainic acid (KA), activating transcription factor 2 (ATF2) knockdown/overexpression, TSC1 overexpression	topiramate (TPM)	ATF2, TSC1, microglial M1/M2 polarization, TNF –α, IL–6, TGF–β, IL–10	↑M1, ↓M2, ↑TNFα, ↑IL-6, ↓TGF-β, ↓IL-10 with KA ↑GluK5/GluK2 with longer KA exposure<br>↓M1, ↑M2, ↓neuroinflammation after ATF2 knockdown	*In vivo:* KA mouse hippocampal model.<br>*In vitro:* KA-induced HMC3 microglia.<br>KA cell treatment: 100 μM for 2, 4 and 6 h.<br>TPM pretreatment: 10 or 100 μg/ml for 18 h. Following this, 100 μM of KA was added for 6 h.
**[465]** Storm et al., 2025	Neuroinflammation	IFN–γ	human milk–derived EVs (MEVs)	Heat shock response (HSR): HSF1, Hsp70, Hsp90, Hsp40, Hsp27	↑HSF1, ↑Hsp70, ↑Hsp90, ↑Hsp40, ↑Hsp27 after MEVs in IFN-γ-polarized microglia<br>↑HSR duration after MEVs in polarized microglia compared to homeostatic cells	HMC3 cells primed with 10 ng/mL IFN–γ to induce polarization. A subset of cells was supplemented with 200 μg of MEVs, isolated from unpasteurized human donor milk.
**[466]** Sun et al., 2025	Neuroinflammation, Alzheimer's disease	Soluble epoxide hydrolase (sEH) inhibitor TPPU, sEH gene expression and EET levels assessed mechanistically, Aβ25–35	N/A	she, TPPU, TLR4,NF–κB p38 MAPK/NF–κB, microglial polarization	↑ROS and ↑oxidative damage, ↓viability induced by Aβ25–35 in HMC3<br>↑Viability, ↓apoptosis, ↓lipid oxidation/ oxidative damage, ↓TNFα, ↓IL-1β, ↓IL-6, ↓IL-18; ↑CD206, ↑SOCS3 expression by TPPU pretreatment	*In vivo:* Aβ42-transgenic Drosophila *In vitro:* Aβ25–35-stimulated human SH –SY5Y–HMC3 co-culture for neuroinflammation.<br>HMC3 cells were pretreated with 1 μM

(Continued)

10.3389/fimmu.2026.1778798

ARTICLE	MODEL	TREATMENT	DRUG TESTED	PATHWAY INVOLVED	MAIN RESULTS	NOTES
						TPPU for 3 h and then incubated with 30 µM Aβ25–35 for 48 h.
**[467]** Hao et al., 2025	Neuroinflammation (Environmental toxicant-induced brain injury)	Dibutyltin dilaurate (DBTDL)	N/A	Predicted core targets AGT, AGTR1, GNB1, GNG2, POMC, oxidative stress (intracellular ROS), BDNF, NT3	↑Cytotoxicity, ↑ROS by DBTDL<br>↑AGT, ↑AGTR1, ↑GNB1, ↑GNG2, ↑POMC, ↑BDNF, ↑NT3 after DBTDL	HMC3 microglia and HBMEC.<br>DBTDL exposure at 0.1–20 µM for 24 h.
**[468]** Jozwiak-Bebenista et al., 2025	Neuroinflammation, MDD	S–ketamine, R–ketamine, 2S,6S –HNK, 2R,6R–HNK, tunicamycin, LPS	S-ketamine, R-ketamine, HNKs	GRP78, HSPA5, CHOP, DDIT3, ATF6, ATF4, IRE1, IL–6, IL–8	↑GRP78, ↑CHOP, ↑ATF6, ↑ATF4, IRE1 activation, ↑IL-6, ↓IL-8 by tunicamycin<br>↓GRP78, ↓CHOP by S-ketamin, R-ketamin, HNKs under tunicamycin-induced ER stress condition<br>↓IL-6, ↓IL-8 by S-ketamin, R-ketamin, HNKs in LPS-stimulated microglia	S-ketamin, R-ketamin, HNKs treatment: 0.1-100 µM for 24 or 48 h.<br>Tunicamycin treatment: 0.5 µg/ml for 24 or 48 h.<br>LPS treatment: 10 ng/ml for 24 or 48 h.
**[469]** Zhou et al., 2025	Alzheimer's disease	Hancinone, Syk inhibitor, Aβ1-42	hancinone	TREM2, Syk, PI3K, AKT, mTOR, microglial polarization, Aβ phagocytosis	• ↑TREM2, ↑Syk, ↑PI3K, ↑AKT, ↑mTOR, shiftedM1→M2, ↑Aβ phagocytosis by hancinone under Aβ or Syk-inhibitor conditions	Hancinone treatment: 0.5, 2.5, 10 µM for 24 h.<br>Syk inhibitor: 100 nmol/l for 24 h.<br>Aβ1-42: 2.5 µM for 24 h.
**[470]** Hou et al., 2025	Chronic insomnia	sh-miR-29a delivered via PLGA nanoparticles (sh-miR-29a-NP), LPS, sh-miR-29a, PER2 siRNA	sh-miR-29a	PER2, NF-κB, microglial polarization	↑miR-29a, ↑PER2 targeting, ↑IBA-1, ↑NF-κB, ↑CD86, ↓CD206 (↑pro-inflammatory M1 phenotype) by LPS stimulation<br>↑PER2, ↑CD206, ↓IBA-1, ↓CD86, ↓NF-κB (↑anti-inflammatory M2 phenotype) after sh-miR-29a treatment in LPS-stimulated cells; these effects were blocked by PER2 knockdown using siRNA	*In vivo:* chronic sleep deprivation (CSD) rat model.<br>LPS: 100 ng/ml for 24 h.<br>h-miR-29a-NP: 0.02, 0.04, 0.08, 0.1, 0.2 mg/100 µL for 24 h.
**[471]** Liu S. et al., 2025	Neuroinflammation, Alzheimer's disease	LPS, Signaling Lymphocytic Activation Molecule Family Member 8 (SLAMF8) overexpression, NINJ2 knockout	N/A	SLAMF8, NINJ2, TLR4, NF–κB, cytokines, oxidative stress	• ↑TLR4, ↑NF-κB activation, ↑inflammation, ↑oxidative stress by LPS<br>• ↑TLR4, ↑NF-κB activation, ↑inflammation, ↑oxidative stress by SLAMF8 overexpression<br>• These effects were blocked by NINJ2 KO	*In vivo:* APP/PS1 transgenic mice.<br>*In vitro:* Aβ1–42-exposed SH-SY5Y cells and LPS-exposed (0.1 µM for 24 h) HMC3 cells.
**[472]** Sang et al., 2025	DR	High glucose, small interfering RNA (siRNA) targeting Dectin-1 (si-Dectin-1), siRNA targeting Syk (si-Syk)	si-Dectin-1, si-Syk	Dectin-1, Syk, NF-κB, TNFα, IL-1β, iNOS, microglia polarization	↑Dectin-1, ↑Syk, ↑NF-κB activation, ↑pro-inflammatory cytokines, and ↑M1 polarization (↑iNOS, ↓Arg-1) induced by HG<br>si-Dectin-1 or si-Syk inhibition reversed these effects	*In vivo:*treptozotocin (STZ) DR-induced C57BL/6J mouse model.<br>*In vitro:* HMC3 cells stimulated with HG (25 mM for 24 h).<br>Control HMC3 cells (glucose 5 mM).
**[473]** Cheng et al., 2025	Alzheimer's disease	Aβ25–35, lncRNA PRR34-AS1 silencing (si-PRR34-AS), lncRNA PRR34-AS1 overexpression (oe-	si-PRR34-AS, oe-PRR34-AS, miR-29c-3p mimic, miR-29c-3p inhibitor	PRR34-AS1/miR-29c-3p axis modulating pro-inflammatory cytokine	• ↓Cell viability, ↑apoptosis, ↑IL-1β, ↑IL-6, ↑TNFα induced by Aβ25–35 treatment<br>• ↑Cell viability, ↓apoptosis, ↓cytokine release by PRR34-AS1 knockdown	Also performed on BV2 cells.<br>Aβ25-35 20 µM 24 h to simulate neuroinflammation in AD.

(Continued)

10.3389/fimmu.2026.1778798

SUPPLEMENTARY TABLE 1 Continued

ARTICLE	MODEL	TREATMENT	DRUG TESTED	PATHWAY INVOLVED	MAIN RESULTS	NOTES
		PRR34-AS), miR-29c-3p manipulation (mimic/inhibitor)		release, cell viability, and apoptosis	• Silencing miR-29c-3p counteracted the anti-inflammatory effects of PRR34-AS1 silencing	
[474] Potapov et al., 2025	Alzheimer's disease	Synthetic Aβ, NOX2 inhibitors of the 3-(indolin-6-yl)-4-(N-pyrazole-sulfonamide)-1 h-pyrrolo [2,3-b] pyridine class (GSK2795039, NCATS-SM7270, isomeric derivative IMBIOC-1)	GSK279503, NCATS-SM7270, IMBIOC-1 (NOX2 inhibitors)	NOX2, superoxide/ROS generation, toxicity, viability	• ↑Oxidative stress, ↓cell survival by Aβ stimulation<br>• ↓Oxidative stress, ↓toxicity by GSK2795039, NCATS-SM7270, IMBIOC-1 in cells under Aβ stimulation (NCATS-SM7270 and IMBIOC-1 conferred stronger protection)	*In vitro:* experiments on human HMC3 microglia cells.<br>Aβ treatment: 10 μM for 24 h.<br>NOX2 inhibitors treatment: 0-100 μM for 24 h.
[475] Cobas-Carreno et al., 2025	DAM phenotype, Alzheimer's disease	anti-TREM2 mAb	varenicline, parbimostat	TREM2–TYROBP, SYK phosphorylation (pSYK/SYK)	• ↑pSYK/SYK by anti-TREM2 mAb, pSYK/SYK further increased by varenicline, no effect was observed with parbimostat<br>•<br>• varenicline could be considered as TREM2–TYROBP transmembrane agonist.	Use of a bacterial two-hybrid (B2 h) system.<br>varenicline or parbimostat treatment: 150 μM
[476] Woolf et al., 2025	*In vitro:* microglia models comparison	Inflammatory stimulation (LPS, IL1β, IFNγ, TNFα), phagocytosis	N/A	Lineage markers (Iba1, CD45, PU.1, PDGFRβ, αSMA, NG2), secretome (IL-6, IL-8, MCP-1, ICAM-1), NO production, phagocytosis	• iPSC-derived microglia showed the strongest inflammatory cytokine release, mouse microglia uniquely produced NO, HMC3 responses were comparatively modest<br>• Primary human and iPSC-derived microglia had significantly higher phagocytic activity than HMC3<br>•	Compared four *in vitro* microglia models: primary human microglia, human iPSC-derived microglia, HMC3 cell line, and primary mouse microglia.<br>Human brain pericytes served as a negative control.
[477] Liu et al., 2025	OIR	Hypoxia, sh-METTL14 (METTL14 knockdown)	N/A	BARD1, METTL14, MXD1, YTHDF2, microglial polarization (iNOS, TNFα, CD206, ARG1), VEGFA, VCAM1, ICAM1	• ↑METTL14 protein after hypoxia<br>• ↓Angiogenic capacity by sh-METTL14<br>• ↑METTL14 by BARD1 in hypoxic microglia<br>• ↓VCAM1, ↓ICAM1, ↓VEGFA after sh-METTL14 in hypoxic microglia<br>• ↑Angiogenesis after METTL14-dependent m6A modification of MXD1 and VEGFA/VCAM1 modulation<br>• METTL14 did not influence polarization in hypoxic microglia	*In vivo:* METTL14 conditional knockout (cKO) mouse.<br>*In vitro*: HMC3 and HRMECs co-culture angiogenesis assays using<br>Normoxia (21% oxygen), hypoxia (2% oxygen)
[478] Oyamada et al., 2025	Neuroinflammation	Recombinant IL-21	N/A	IL-21, HIF-1α, IL-6, Bodipy, CD36, TREM-2, ApoE	• ↑HIF-1α, ↑IL-6, ↑Bodipy, ↑CD36, ↑ApoE after IL-21<br>•	Blood collected from aged (65–90 years) and young (20–40 years) human subjects.<br>*In vivo:* healthy C57BL/6 mice.<br>IL-21 treatment: 10 ng/ml for 72 h.

(Continued)

10.3389/fimmu.2026.1778798

SUPPLEMENTARY TABLE 1 Continued

ARTICLE	MODEL	TREATMENT	DRUG TESTED	PATHWAY INVOLVED	MAIN RESULTS	NOTES
**[479]** Wang et al., 2025	Neurobrucellosis model	*B. suis* S2–infected cells	Dox	CALR, intracellular Ca^{+2}, IRE1, Caspase-12, Caspase-3, apoptosis	• ↓CALR, ↑intracellular Ca^{+2}, ↑apoptosis (activation of IRE1/Caspase-12/Caspase-3 pathway) by Dox in both uninfected and *B. suis* S2–infected cells	*In vitro:* HMC3 cells and *Brucella suis* S2-infected HMC3 cells. Also used CALR-overexpressing (CALR), and CALR-knockdown (sh-CALR) cell lines. Dox treatment: 20, 40, 80, 160 µM for 6, 12, 24 h (160 µM for 12 h was the key treatment).
**[480]** Polini et al., 2025	Neuroinflammation, Alzheimer's disease	LPS, TNFα, Aβ25-35	TG68 (THRβ agonist)	IL-6, IL-10, TNFα	• ↑IL-6 with LPS/TNFα<br>• ↓IL-6, ↑IL-10 by TG68 pretreatment with LPS/TNFα<br>• ↑TNFα, ↑IL-6 with Aβ25-35<br>• ↓TNFα, ↓IL-6, ↑IL-10 by TG68 pretreatment with Aβ25-35<br>•	*In vivo:* High-fat diet mouse model treated with TG68 (10 mg/kg/day, 2 weeks). LPS (10 µg/ml)/TNFα (50 ng/ml) for 24 h or Aβ25-35 (10 µM) for 24 h. TG68 pretreatment: 0.1–10 µM for 24 h.
**[481]** Zhao et al., 2025	Neurosyphylis	Recombinant TpF1 (*Treponema pallidum* protein), 740Y-P (PI3K/AKT agonist)	N/A	TLR4, PI3K, AKT, Rac1, actin polymerization (F-actin/G-actin)	• ↓Migration in both horizontal and vertical directions by TpF1<br>• ↓F-actin/G-actin ratio, ↓TLR4, ↓P-PI3K/PI3K ratio, ↓P-AKT/AKT ratio, Rac1 by TpF1	Recombinant TpF1 treatment: 6.25, 12.5, 25, and 50 µg/ml for 24 h
**[482]** Nong et al., 2025	Alzheimer's disease	Aβ1-42, MHY1485 (mTOR activator)	mangiferin (natural polyphenol from *Mangifera indica*)	AMPK, mTOR, IRF5, IL-1β, TNFα	• ↑IL-1β, ↑TNFα, ↑mTOR, ↑IRF5 by Aβ1-42<br>• ↑AMPK phosphorylation, inhibition mTOR activation, ↓IRF5, ↓IL-1β, ↓TNFα by mangiferin	*In vivo:* SAMP8 accelerated aging mice. *In vitro:* HMC3 and SH-SY5Y. Aβ1-42 treatment: 20 µM for 72 h. mangiferin treatment: 12.5, 25, 50, 100, 200, 300, 400, 500, 600, and 700 µM for 24 h. MHY1485 treatment: 10 µM for 6 h.
**[483]** Hu et al., 2025	Neuroinflammation (Chronic cerebral ischemia (CCI)), Kidney injury	PX-478 (HIF-1α inhibitor), OGD	gaicalin + geniposide (BC/GD)	HIF-1α, EPO, NF-κB, microglial polarization, cytokines release	• ↑NF-κB phosphorylation, ↑pro-inflammatory cytokines by OGD<br>• ↑HIF-1α, ↑EPO, ↓NF-κB activation, ↓pro-inflammatory cytokines by BC/GD<br>• PX-478 blocked BC/GD's modulation of HIF-1α, EPO, NF-κB and cytokines changes	*In vivo:* SD rats. *In vitro:* HMC3 and HK-2 kidney cells. Network pharmacological analysis. BC/GD treatment: 1.56, 3.125, 6.25, 12.5, 25, 50, 100, 200, 400, and 800 µM for 24 h. OGD model: 94% N_2, 5% O_2, 1% O_2 for 1, 2, 3, 4, 5, 6 h.
**[484]** Kilic et al., 2025	Cancer (GBM)	Boric acid, ferroptosis inhibitor Fer-1	Boric acid	SOX10, GPX4, ACSL4, $Fe^{+2,}$ TFR, GSH, MDA, ROS	• IC50: HMC3 are ~6-fold more resistant to boric acid than U87 cells<br>• Baseline expression of SOX10 is lower in HMC3 compared to U87 cells	HMC3 cells served as a comparative cell line. Boric acid treatment: 1.6-25 mM for 24 h.
**[485]** Evora et al., 2025	Alzheimer's disease	APP695 (SH-SY5Y cells transfected with the amyloid precursor with the Swedish mutation), H2O2	miR-124-3p–loaded SH-SY5Y exosomes (ET124), exosomes collected from miR-124–transfected SH-SY5Y cells (CT124)	Cell viability, microglial activation	• APP695 + H2O2 induced microglial activation.<br>• ET124 redirected HMC3 profiles toward	*In vitro:* Primary microglia from B6SJLF1/J mouse pups. Organ-on-chip microfluidic triculture of human SH–SY5Y neuroblastoma cells (±

(Continued)

10.3389/fimmu.2026.1778798

SUPPLEMENTARY TABLE 1 Continued

ARTICLE	MODEL	TREATMENT	DRUG TESTED	PATHWAY INVOLVED	MAIN RESULTS	NOTES
					a steady state and attenuated inflammatory condition more effectively than CT124.	APP695), HMC3, and immortalized human astrocytes IM-HA.<br>Exosome treatment: 2.16 ± 1.41 × 10^7 particles<br>per µg of exosomal protein for 24 h.<br>H2O2 treatment: 10 µM for 24 h.
**[486]**<br>Wang et al., 2025	Viral infection (EV71)	EV71 infection, IFNα, miR-362-3p mimic or inhibitor	IFNα	Mir-362-3p, cell viability	• ↑miR-362-3p expression, ↑cell viability by IFNα<br>• ↑Cell viability by miR-362-3p mimic<br>• ↓Cell viability by miR-362-3p inhibitor<br>•	*In vitro:* RD rhabdomyosarcoma and HMC3 microglia cells.<br>EV71 infection: MOI=0.1, 0.5, and 1 for 24 h.<br>Mir-362-3p transfection: 20 µM stock solutions in ddH_2O.<br>IFNα treatment: 10, 100<br>and 1000 U/mL for 2 or 6 h.
**[487]**<br>King et al., 2025	Alzheimer's disease	LPS, ATP, cleavable RILP (Rab-interacting lysosomal protein) transfection, noncleavable RILP transfection, shRab27 transfection, phosphomimetic tau(E14) transfection, cycloheximide	N/A	NRLP3, Rab7, RILP cleavage, tau, EVs secretion	• ↑RILP cleavage, ↓tau(E14) degradation, ↑tau(E14) in the EVs by LPS + ATP.<br>• These effects were mitigated by noncleavable RILP.<br>• ↑inflammatory state by tau(E14) internalization.<br>• Impaired tau degradation and ↑EVs secretion by cleaved RILP.	Total brain lysate analysis from AD and non-AD patients.<br>*In vitro:* neuroblastoma BE(2) and HMC3 cells.<br>LPS/ATP treatment: 1 µg/ml LPS for 3 h and then 5 mM ATP for an additional 60 min.<br>Cycloheximide treatment: 100 µg/µl for 2 and 4 h.
**[488]**<br>Sinclair et al., 2025	Neuroinflammation	LPS, Aβ1-42, HIV glycoprotein (GP120)	Cannabidiol (CBD)	Inflammation, ROS	• ↑ROS by LPS, CBD attenuated LPS-induced ROS levels.<br>• ↑ROS by Aβ42, CBD attenuated Aβ42-induced ROS levels.<br>• ↑ROS by GP120, CBD did not attenuated GP120-induced ROS levels.	*In vitro:* After CBD pre-treatment, cells were challenged with LPS, Aβ1-42 or GP120.<br>CBD pre-treatment: 10 µg/ml for 12 h,<br>LPS treatment: 100 ng/ml for 12 hours.<br>Aβ42 treatment: 100 nM for 12 hours.<br>GP120 treatment: 500 pM for 12 hours.
**[489]**<br>Hu et al., 2025	Alzheimer's disease	LPS, PI3K-AKT pathway inhibitor LY294002	Berberine (BBR)	iNOS COX2, ARG1, IL-1β, IL-6, TNFα, IL-10, PI3K-AKT	• BBR at ≤8 µM had no cytotoxicity.<br>• LPS induced classical M1 phenotype.<br>• BBR dose-dependently suppressed M1 polarization.<br>• BBR shifted microglia to M2 phenotype.<br>• ↺PI3K-AKT pathway activity by BRB when suppressed by LPS.<br>•	Bioinformatic analysis (WGCNA).<br>*In vitro:* SH-SY5Y and HMC3 cells.<br>BBR treatment: 1, 2, 4, 8 and 16 µM for 3 or 24 h. LPS treatment: 1 µg/ml for 24 h.<br>LPS and BBR co-treatment: BBR 2, 4, 8 µM for 3 h followed by BBR and LPS (1 µg/ml) for 24 h.
**[490]**<br>Kim et al., 2025	AMD	TNFα, AAV2-shmTOR vector infection	N/A	IL-6. IL-1β, NF-κB	• ↑IL-6, ↑IL-1β, ↑NF-κB by TNFα.<br>• TNFα did not compromise HMC3 viability.<br>• ↓IL-6, ↓IL-1β, ↓NF-κB (TNFα-induced) by AAV2-shmTOR.	HMC3, HUVECs and ARPE.<br>TNFα treatment: 50, 100 and 200 ng/ml for 24 h or 72 h.<br>AAV2-shmTOR infection: 1 × 10^4 vg/cell for 72 h.

(Continued)

10.3389/fimmu.2026.1778798

SUPPLEMENTARY TABLE 1 Continued

ARTICLE	MODEL	TREATMENT	DRUG TESTED	PATHWAY INVOLVED	MAIN RESULTS	NOTES
**[491]**<br>Rifa et al., 2025	Cytotoxicity	bisphenol analogs BPAF, BPAP, BPE and BPP	N/A	ROS, mitochondrial membrane potential (ΔΨm), mitochondrial Ca^{+2} levels	• ↑ROS by BPAF, BPAP and BPP.<br>• ↓ΔΨm by BPP.<br>• ↑Ca^{+2} by BPAF and BPP.	HepaRG, Caco-2, HMC3 and HMEC-1.<br>Bisphenol analogs treatment: 0.01-10 µM for 24 h.
**[492]**<br>Gao et al., 2025	Neuroinflammation (Stroke)	OGD/R, C5orf24 gene overexpression (GFP-tagged gene transfection), C5orf24 gene knockdown (siRNA transfection)	N/A	IL-1β, IL-6	• ↓C5orf24 by OGD/R.<br>• ↓IL-1β, ↓IL-6 by C5orf24 overexpression under OGD/R.<br>• ↑IL-1β, ↑IL-6 by C5orf24 knockdown under OGD/R.<br>•<br>•	OGD (1% O2) for 6 h, reperfusion for 0, 6, 12, 18, 24, 48 h.
**[493]**<br>Du et al., 2025	Neurosyphilis	flaB3 (bacterial flagellin), TAK-242 (TLR4 inhibitor), shTLR4 (TLR4 knockdown), rapamycin, LY294002, LPS, FITC-Aβ1-42.	rapamycin	IL-6, IL-8, IL-10, IL-1β, TNFα, TLR4, mTOR, Aβ degradation, Beclin1, LC3II/I, p62	• ↑inflammation, inhibited autophagy, ↓Aβ degradation by FlaB3.<br>• ↓IL-6, ↓IL-8 by TAK-242 under FlaB3.<br>• ↓IL-6, ↓IL-8, ↑autophagy by shTLR4 under FlaB3.<br>• ↓IL-6, ↓IL-8, ↑autophagy, ↑Aβ degradation by rapamycin under FlaB3.<br>• ↓IL-6, ↓IL-8, ↑autophagy, ↑Aβ degradation by LY294002 under FlaB3.	FlaB3 treatment: 1, 5, 10 µg/ml for 6, 12, 24, 48 h.<br>TAK-242 pretreatment: 250, 500, 1000 nM for 1 h.<br>shTLR4 treatment: 12 h.<br>Rapamycin pretreatment: 50 µM for 1 h.<br>LY294002 pretreatment: 5 µM for 1 h.<br>LPS treatment: 100 ng/ml for 12 h.<br>FITC-Aβ1-42: 0.3 µM.
**[494]**<br>Wang et al., 2025	Neurobrucellosis	*B. suis S2*, CALR overexpression, sh-CALR	N/A	IRE1, caspase-12, caspase-3, ubiquitination, ER integrity, intracellular Ca^{2+}, apoptosis	• ↓p-IRE1, ↓cleaved-caspase-12, ↓cleaved caspase-8, ↑CALR, ↓apoptosis by *B. suis S2* for 2 h and MOI 50.<br>• ↑p-IRE1, ↑cleaved-caspase-12, ↑cleaved caspase-8, ↓CALR, ↑ER-stress by *B. suis S2* for 8 h and MOI 200.<br>• ↑ubiquitination by *B.suis S2*.<br>• ↓p-IRE1, ↓cleaved-caspase-12, ↓cleaved-caspase-3 by CALR overexpression.<br>• ↑p-IRE1, ↑cleaved-caspase-12, ↑cleaved-caspase-3, ↓ER integrity, ↑Ca^{+2}, ↑apoptosis by shCALR.<br>•	*B. suis* S2 treatment: MOI 25, 50, 100 or 200 for 1, 2, 4 or 8 h.
**[495]**<br>Fang et al., 2025	Cancer (Glioma)	substrate stiffness (collagen I-coated hydrogel), yes-associated protein (YAP) overexpression, YAP knockdown (shYAP)	cytochalasin D (inhibitor of actin polymerization), Y-27632 (Rho-associated protein kinase (ROCK) inhibitor). blebbistatin (myosin II inhibitor), leptomycin-B (nuclear export inhibitor)	YAP nuclear localization, NF-κB, IL-6, IL-10	• ↓nuclear YAP, ↓NF-κB, ↑IL-6, ↓IL-10 by softer hydrogels.<br>• ↑nuclear YAP, ↑NF-κB, ↑IL-10, ↓IL-6 by stiffer hydrogels.<br>• ↓IL-10, ↑IL-6, ↓stiffness dependence by shYAP.<br>• ↓nuclear YAP, ↓IL-10, ↑IL-6 by cytochalasin D.<br>• ↑nuclear YAP, ↑IL-10 by leptomycin-B.<br>•	U87-MG, U251-MG, human embryonic kidney 293T (HEK293T) and HMC3.<br>Substrate stiffness: 0.4-40 kPa.<br>Cytochalasin D: 10 µM for 30 min.<br>Y-27632: 30 µM for 30 min.<br>Blebbistatin: 30 µM for 30 min.<br>Leptomycin B: 1 ng/ml.

(Continued)

10.3389/fimmu.2026.1778798

SUPPLEMENTARY TABLE 1 Continued

ARTICLE	MODEL	TREATMENT	DRUG TESTED	PATHWAY INVOLVED	MAIN RESULTS	NOTES
**[496]** Graur et al., 2025	HIV associated neurocognitive disorders (HAND)	gp120B/C, LPS, bafilomycin A1 (inhibitor of autophagy)	nicotine	Mitochondrial proteins (PHB2, MFN2, FIS1, cyt c), mitochondria morphology, APP peptides, APP vesicles, autophagy	• ↑PHB2, ↑MFN2, ↓FIS1, ↓volume/area/branch length, ↓APP vesicles, ↑extracellular APP peptides by gp120. • ↑PHB2, ↑MFN2, ↓FIS1, ↑cyt c, ↑volume/area/branch length, ↑APP vesicles, ↓extracellular APP peptides, ↑LC3B-II by nicotine+gp120.	nicotine pretreatment: 10 μM for 48 h, then 500 pM gp120 was added for an additional 24 h. Gp120B/C treatment: 500 pM for 24 h. Bafilomycin A: 20 nm for 24 h. LPS 100 pM for 2 h.
**[497]** Wang et al., 2025	Neuroinflammation (SCI)	LPS, Olfactory mucosal mesenchymal stem cells (OM-MSCs) exosomes, oe-RMRP, sh-RMRP, oe-SIRT1, sh-EIF4A3	N/A	Pyroptosis (NLRP3, cleaved caspase-1, GSDMD-N), EIF4A3, RMRP, SIRT1, IL-1β, IL-18 viability	SIRT1 stabilized by exosomal lncRNA RMRP via EIF4A3 mitigated LPS-induced microglial pyroptosis. Overexpression of SIRT1 lightened LPS –induced microglial pyroptosis.	*In vivo:* C57BL/6 male mice. LPS: 100 ng/ml for 24 h. Co-culture with OM-MSC exosomes for 48 h.
**[498]** Zeng et al., 2025	Mitochondrial proteome	LPS, IR780, 3-ethynylaniline (3-EA), 808 nm irradiation	N/A	Mitochondrial proteins, ROS	• 624 mitocondria-associated proteins were identified, of which 314 proteins were located in mitochondria. • After LPS stimulation, most of the differentially expressed proteins showed an upregulated trend (proteins involved in cell proliferation and differentiation and related to stress response and neuroinflammation).	HeLa and HMC3. LPS: 0.1, 1, 10 μg/ml for 48 h. IR780 2 μM for 30 min. 3-EA: 5 mM for 10 min. 808 nm irradiation, 0.4 W/cm^2, 60-90 s.
**[499]** Zhang et al., 2025	Parkinson's disease	oligomeric α-Syn,	Stattic (STAT3 inhibitor), Colivelin TFA (STAT3 activator)	IL6ST, JAK2, STAT3, HIF-1α, CD-206, ACSL4, SLC7A11, oxidative stress, lipid peroxidation, Fe^{+2}	• ↓Viability at α-Syn > 25 μM • ↑ROS at α-Syn < 5 μM. • ↓ROS at α-Syn > 10 μM. • ↑CD-206 by α-Syn. • ↑Lipid peroxidation, ↑ferroptosis by α-Syn. • ↑Lipid peroxidation, ↑ferroptosis by Stattic. • ↓Lipid peroxidation, ↓ferroptosis by Colivelin TFA • ↓Ferroptosis by STAT3. • Identification of IL6ST/JAK2/STAT3/HIF-1α as control pathway for ferroptosis in α-Syn stress.	α-Syn: 0-25 μM for 24 h. Stattic: 10 μM for 4 h. Colivelin TFA: 1 μM for 24 h.
**[500]** Gunasegaran et al., 2025	Neuroinflammation	LPS, IFN-γ	N/A	Proteomics, morphology, inflammation, antigen presentation pathways, cytokines, kynurenine pathway	• Detection of 3713 proteins (591 proteins unique in HMC3 compared to C20). • ↑NFYA, ↓OSGEPL1, ↑increased cell size, ↑IL-6 by LPS. • ↑WARS1, ↑ISG20, ↓SDHAF2, ↑increased cell size, ↑kynurenine by IFN-γ.	Comparison between HMC3 and C20 microglial cell lines. LPS: 1 μg/ml for 24 and 48 h. IFN-γ: 50 ng/ml for 24 and 48 h.

(Continued)

10.3389/fimmu.2026.1778798

SUPPLEMENTARY TABLE 1 Continued

ARTICLE	MODEL	TREATMENT	DRUG TESTED	PATHWAY INVOLVED	MAIN RESULTS	NOTES
**[501]** Akinduro et al., 2025	Neuroinflammation (Neonatal)	Human breast milk-derived exosomes (HBME), LPS	N/A	NF-κB, IL-1β, microglial activation	• ↑NF-κB p65, ↑IL-1β, ↑Iba1, activated morphology by LPS.<br>• ↓NF-κB p65, ↓Iba1, cell morphology toward resting state by HBME.	HBME characterization.<br>BV2 and HMC3.<br>LPS: 100 ng or 1 μg/ml for 0.25, 0.5, 1, or 24 h.<br>HBME: 5 or 10 μg/ml for 0.25, 0.5, 1, or 24 h.
**[502]** Jia et al., 2025	Neuroinflammation (Cerebral ischemia/ reperfusion after ischemic stroke)	OGD/R, LAMP3 overexpression, Rapamycin (mTOR inhibitor), Chloroquine (autophagy inhibitor), BAY 11-7082 (NF-κB pathway inhibitor)	N/A	LAMP3, NF-κB, mTOR, LC3-II/I, Beclin-1, P62, LAMP3, IL-6, IL-10, IL-13, TNFα, oxidative stress, cell viability	• ↑ LC3-II/I, ↑Beclin-1, ↑P62, ↓LAMP3 expression, mild oxidative stress by OGD/R.<br>• ↓LC3-II/I, ↓Beclin-1, ↓P62, ↑p-p65/p65, ↑p-IκBa/IκBa, ↑ROS, ↑LDH, ↑TNFα (mRNA and protein), ↑IL-6 (mRNA and protein), ↓IL-10 mRNA, ↓IL-13 mRNA, ↓cell viability by LAMP3 overexpression.<br>• No changes in LAMP3 levels and effects by Rapamycin or Chloroquine. BAY 11-7082 blocks or attenuates LAMP3 effects.<br>•	*In vivo:* male C57BL/6 J mice.<br>*In vitro:* SH-SY5Y and HMC3.<br>OGD/R treatment: 0-10 h (1% oxygen), then reoxygenation for 2-24 h.<br>Rapamycin pre-treatment: 0-200 nM for 1, 2, 4 h before OGD/R.<br>Chloroquine pre-treatment: 0-50 μM for 1, 2, 4 h before OGD/R.<br>BAY 11-7082 pre-treatment: 24 h before OGD/R.
**[503]** Agarwal et al., 2025	Neuroinflammation following injury	Injectable human peripheral nerve (iHPN) hydrogel crosslinked with genipin, LPS, collagen hydrogel crosslinked wity genipin	N/A	CD68, IL-4, TNFα, IL-1β	• ↑IL-1β, ↓IL-4, no change in TNFα by LPS compared to control.<br>• ↓IL-1β, ↑IL-4, no change in TNFα by iHPN compared to LPS.<br>• ↓IL-1β, ↑IL-4, ↓CD68, no change in TNFα by iHPN+LPS compared to LPS.	Study on a novel iHPN, created from decellularized and delipidaded human sciatic nerves.<br>primary brain neurons, SH-SY5Y, neonatal human astrocytes, HMC3, mouse RAW 264.7 macrophages.<br>LPS: 10 μg/ml added to the cultured media on days 3, 5 and 7.
**[504]** Wang et al., 2025	Neuroinflammation	p-Synephrine (p-SYN), LPS, SB-271046 (5-HT6R antagonist), WAY-181187 (5-HT6R agonist), SCH772984 (ERK1/2 inhibitor)	p-SYN	ERK1/2, IL-10, CD86, iNOS, Arg-1, CD206	• ↑CD86, ↑iNOS, ↓CD206, ↓Arg-1, ↓IL-10, ↑5-HT6R by LPS.<br>• ↑IL-10, ↓CD86, ↓iNOS, ↑Arg-1, ↑CD206, ↓5-HT6R by p-SYN + LPS compared to LPS.	*In vivo:* 8-week-old male CD1 (ICR) and 8-week-old male C57BL/6 mice.<br>p-SYN: 0.001-100 μM for 8-24 h.<br>LPS: 1 μg/ml for 12 h.
**[505]** Yang et al., 2025	Neurotoxicity	Polystyrene microplastics (PS-MPs)	N/A	Microglial activation (CD68, CD16), morphological changes, inflammation (IL-6, IL-1β), circadian rhythm genes (CRY2, PER3)	• No change in viability/proliferation, ↑vesicle density, ↑morphological abnormalities, ↑CD68, ↑CD16 ↑IL-6, ↑IL-1β, ↑CRY2, ↑PER3 by PS-MPs	*In vivo:* zebrafish.<br>PS-MPs: 0-250 μg/l for 12, 24, 48 h.
**[506]** Peng et al., 2025	Neuroinflammation (Ischemic stroke)	OGD/R	N/A	NLRX1, TNFα, autophagy, ATG5, NLRP3	• ↓NLXR1 by OGD/R.<br>• ↓neuronal damage, ↓TNFα, ↑autophagy, ↑ATG5 by NLXR1 overexpression.<br>• ↓NLRP3, ↓cleaved caspase 1 by OGD/R + NLXR1 overexpression.<br>• ↓effects of NLRX1 elevation on NLRP3 inflammasome signaling by ATG5	*In vivo:* rat middle cerebral artery occlusion (MCAO)-induced cerebral ischemia/reperfusion injury (CIRI) model.

(Continued)

10.3389/fimmu.2026.1778798

SUPPLEMENTARY TABLE 1 Continued

ARTICLE	MODEL	TREATMENT	DRUG TESTED	PATHWAY INVOLVED	MAIN RESULTS	NOTES
					depletion.<br>•	
**[507]**<br>Sakai et al., 2025	Neuroinflammation	α-pyrrolidinooctanophenone (α-POP), 3′,4′-methylenedioxy ring (MD-α-POP), 4′-fluoro group (F-α-POP), methamphetamine (METH), Stattic (STAT3 inhibitor), LY294002 (PI3K inhibitor), UO126 (MAPK inhibitor), N-acetyl-L-cysteine (NAC)	N/A	Cell viability, LDH, ROS, IL-6, NOX2, NOX4, NADP+/NADPH, STAT3, ERK, Akt	• ↑viability, ↓LDH, ↑ROS by α-POP 5-20 μM.<br>• ↑IL-6, ↑NOX2, ↑p-STAT3, ↑p-ERK, ↑p-Akt, ↑NADP+/NADPH ratio by α-POP 10-20 μM.<br>• ↓viability by α-POP + Stattic or LY294002 or UO126 compared to α-POP.<br>• MD-α-POP and F-α-POP increased viability at similar concentrations to α-POP.<br>• ↑viability by METH at 100 μM.<br>•	α-POP: 0-80 μM for 0-48 h.<br>MD-α-POP: 0-40 μM for 24 h.<br>F-α-POP: 0-40 μM for 24 h.<br>METH: 0-200 μM for 24 h.<br>Stattic: 0.5-1 μM for 2 h (pre-treatment).<br>LY294002: 1 μM 2 h (pre-treatment).<br>UO126: 1 μM 2 h (pre-treatment).<br>NAC: 1-2 mM 2 h (pre-treatment).
**[508]**<br>Zeng et al., 2025	Alzheimer's disease	Airborne ultrafine particulate matter (PM$_{0.1}$), circ_0061183 silencing through two circ_0061183 small interfering RNAs (siRNAs), miR-98-5p mimic	N/A	Cell viability, LDH, IBA1, CD16, CD32, IL-1β, IL-6, iNOS, CD163, CD206, IL-4, IL-10, TGF-β pathway	• ↓viability, ↑LDH, ↑IBA1, ↑CD16, ↑CD32, ↑IL-1β, ↑IL-6, ↑iNOS, ↓CD163, ↓CD206, ↓IL-4, ↓IL-10, ↑circ _0002210, ↑circ_0030223, ↑circ_0046395, ↓circ_0006469, ↓circ_0030224, ↓circ_0040038, ↓0046394,<br>• ↓circ_0053318, ↓circ_0061183, ↑miR-98-5p by PM$_{0.1}$.<br>• ↑IBA1, ↑IL-1β, ↑IL-6, ↑iNOS, ↓CD163, ↓CD206, ↓IL-4, ↓IL-10 by circ_0061183 siRNA + PM$_{0.1}$.<br>• ↓IL-10 by mIR-98-5p mimic + PM$_{0.1}$.<br>• ↓IL-10 by circ_0061183 siRNA + mIR-98-5p mimic + PM$_{0.1}$.<br>• ↓TGFBR1 by mIR-98-5p mimic and/or circ_0061183 siRNA + PM$_{0.1}$.	*In vivo:* male C57BL/6 J mice.<br>PM$_{0.1}$: 6.25, 12.5, 25, 50 and 100 μg/ml for 24, 48 and 72 h.
**[509]**<br>Silva et al., 2025	Neuroinflammation, Alzheimer's disease	Human plasma-derived EVs (pEVs), adipose-derived mesenchymal stem cells EVs (ADMSC-EVs), Donepezil (DNZ), LPS	DNZ	Cell viability, IFN-γ, IL-8, IL-12p40, MCP1, TNFα, IL-6, ROS, phagocytosis	• No change in viability by ADMSC-EVs or pEVs or free DNZ or ADMSC-EVs-DNZ or pEVs-DNZ.<br>• ↑IFN-γ, ↑IL-8, ↑IL-12p40, ↑MCP1, ↑TNFα, ↑IL-6, ↑phagocytosis, ↑ROS by LPS.<br>• ↓IFN-γ, ↓IL-8, ↓IL-12p40, ↓MCP1, ↓TNFα, ↓IL-6 by ADMSC-EVs + LPS compared to LPS.<br>• ↓IL-6 by pEVs + LPS compared to LPS.<br>• ↓ROS by free DNZ+LPS compared to LPS.<br>↓ROS, ↓phagocytosis by pEVs-DNZ or ADMSC-EVs-DNZ + LPS compared to LPS.<br>• DNZ loaded EVs reduced LPS-induced ROS and phagocytosis more than free DNZ.	*In vivo:* zebrafish Tg(flk:EGFP) and Tg(mpeg1:mCherry) lines (*Danio rerio*).<br>*In vitro:* HMC3 cells.<br>LPS: 0.1-1 μg/ml for 24 h.<br>DNZ: 10, 20, 40 μM for 24 h.<br>EVs: 5x10^8-5x10^9 particles/cm^2.

(Continued)

10.3389/fimmu.2026.1778798

10.3389/fimmu.2026.1778798

SUPPLEMENTARY TABLE 1 Continued

ARTICLE	MODEL	TREATMENT	DRUG TESTED	PATHWAY INVOLVED	MAIN RESULTS	NOTES
**[510]** Sakrajda et al., 2025	Bipolar disorder	innate-immune cytokines cocktail (IMC) TNFα + IL1-β + IFN-γ	lithium	TSPO, TLR4, NKFB1, CASP1, CASP4, NLRP3, IL-1β, IL-6, caspase activity, extracellular IL-1β, phospho-GSK-3β(Ser9), lactate	• ↑CASP1, ↑CASP4, ↑IL1B, ↑IL6, ↑IL-1β by IMC short-term.<br>• ↓NLRP3, ↓CASP1, ↑TSPO, ↓NF-KB1 by IMC then Lithium short-term compared to IMC short-term.<br>• ↑TSPO, ↑CASP4, ↑IL1B, ↑IL6, ↑NLRP3 by Lithium then IMC short-term compared to IMC then Lithium short-term.<br>• ↑Caspase activity by Lithium then IMC short-term compared to IMC short-term or IMC then Lithium short-term.<br>• ↑TSPO, ↑TLR4, ↑NLRP3, ↑CASP1, ↑CASP4, ↑IL1B, ↑IL6 by IMC long-term.<br>• ↓TSPO, ↓TLR4, ↓NF-KB1, ↓NLRP3, ↓CASP1, ↓CASP4 by IMC then Lithium long-term compared to IMC long-term.<br>• ↑NLRP3, ↑CASP4, IL1B by Lithium then IMC long-term compared to IMC then Lithium long-term.<br>• ↑IL-1β by Lithium then IMC long-term compared to IMC then Lithium long-term.<br>• ↑phospho-GSK-3β by Lithium then IMC or IMC then Lithium short- and long-term.	Lithium: 0.5 mM Li_2CO_3 for 1-6 days.<br>IMC: TNFα 10 ng/ml + IL-1β 50 ng/ml + IFN-γ 20 ng/ml for 24 h.
**[511]** Fang et al., 2025	White matter injury (WMI)	OGD, recombinant human fibroblast growth factor (rhFGF) 21, PD173074 (FGFR1 inhibitor), PPARγ siRNA	N/A	FGFR1/β-klotho, inflammation, oxidative stress, microglial polarization, NF-κB, NRF1	• ↑TNFα, ↑IL-1β, ↑IL-6, ↑COX-2, ↑IL-10, ↑ROS by OGD.<br>• ↓TNFα, ↓IL-1β, ↓IL-6, ↓COX-2, ↑IL-10, ↓ROS, ↓iNOS, ↓CD16/32, ↑CD206, ↑Arg1, ↓cytosolic HMGB1, ↓NF-κB activation, ↑NRF1 nuclear translocation by rhFGF21 pretreatment + OGD in comparison to OGD.<br>• PD173074 or PPARγ siRNA reversed rhFGF21 effects.<br>•	*In vivo:* WMI neonatal mice model.<br>OGD: 1% O2 for 18 h.<br>rhFGF21: 100 nM for 2 h prior to OGD.<br>PD173074: 10 nM for 2 h with rhFGF21 prior to OGD.<br>PPARγ siRNA: 10 nM transfection 48 h prior to OGD.
**[512]** Zhao et al., 2025	Perioperative neurocognitive disorders	Sevo, PTPN2 overexpression (lentivirus vector) or siliencing (shRNA), static (STAT3 inhibitor)	N/A	PTPN2, STAT3, NLRP3, microglial polarization	• ↑p-STAT3, ↑NLRP3, ↑clv-Casp1, ↑IL-1β, ↑IL-18, ↑CD16, ↑iNOS, ↓CD206, ↓Arg1 by Sevo.<br>• PTPN2 overexpression reversed these effects, while PTPN2 knockdown increased them.<br>• Pro-inflammatory changes driven by PTPN2 knockdown were abrogated by stattic treatment.	*In vivo:* male Sprague-Dawley 18-month rats.<br>*In vitro:* IMR-32 neuroblastoma and HMC3 cells.<br>Sevo: 2% concentration for 5 h.<br>Stattic: 100 μM for 5 h.

(Continued)

SUPPLEMENTARY TABLE 1 Continued

ARTICLE	MODEL	TREATMENT	DRUG TESTED	PATHWAY INVOLVED	MAIN RESULTS	NOTES
**[513]** Li et al., 2025	TBI	LPS + ATP, Mdivi-1 (mitophagy inhibitor)	Nicotinamide n-oxide (NAMO)	Cell viability, mitophagy, mitochondrial function, oxidative stress, pyroptosis	• ↑viability, ↓NLRP3, ↑GSDMD, ↓Casp1 p20, ↓ROS, ↑JC-1 red/green ratio, ↑LC3B, ↓p62 by NAMO + LPS + ATP compared to LPS + ATP treatment.<br>• Mdivi-1 treatment reversed NAMO's effects.	*In vivo:* male Sprague-Dawley rats.<br>NAMO: 160 μM for 24 h.<br>LPS 100 ng/ml for 24 h + ATP 5 mM for 30 min.<br>Mdivi-1: 10 μM for 24 h.
**[514]** Zhao et al., 2025	Diabetes-associated cognitive impairment	High glucose (HG), LP17 (TREM1 inhibitor)	N/A	ER stress, iron metabolism, lipid peroxidation, ferroptosis	• ↑TREM1, ↑TFR1, ↑FTL, ↓FPN1, ↑ROS, ↓GPX4 by HG.<br>• ↓TREM1, ↓TFR1, ↓ROS, ↑GPX4 by LP17 + HG compared to HG.<br>•	*In vivo:* C57BL/6J mice.<br>*In vitro:* BV2 and HMC3 cells.<br>HG treatment: 25 mM for 72 h.<br>LP17: 10 μM for 72 h.
**[515]** Akinlusi et al., 2025	Neuroborreliosis	*Borrelia burgdorferi* infection	N/A	Microglial activation and polarization	• Early ↓ followed by a later ↑iNOS, ↓CX3CR1, ↑MIP-1α, ↑MIP-1β, ↑IP-10, ↑MCP-1, ↑IL-8, ↑VEGF by *B. burgdoferi* infection.	*B. burgdoferi* infection: 4-24 h.
**[516]** Shu et al., 2025	Neuroinflammation (Hypoxic-ischemic brain damage)	OGD/R, bone mesenchymal stem cells-derived exosomes overexpressing miR-653-3p (Exo/miR), miR-653-3p mimics	N/A	Proliferation, TRIM21, p62, Keap1, Nrf2, inflammation, apoptosis	• ↑miR-653-3p, ↑proliferation, ↓apoptosis, ↓lipid ROS, ↓IL-1β, ↓TNFα, ↓IL-6 by Exo/miR in HMC3-OGD.<br>• ↓TRIM21, ↑p62, ↓Keap1, ↑Nrf2, ↑HO-1 by miR-653-3p mimics.	*In vivo:* Sprague-Dawley rats 3 days old.<br>*In vitro:* bone marrow mesenchymal stem cells (BMSCs), human embryonic kidney (HEK)-293T cells and HMC3 cells.<br>OGD/R: 6 h in deoxygenated glucose-free medium followed by 24 h reoxygenation.
**[517]** Lipari et al., 2025	Alzheimer's disease	Aβ1-42, mild hypoxia	Melatonin and naringenin (SIRT1 inducers), EX-527 (selective SIRT1 inhibitor)	SIRT1, NF-κB,	• ↓SIRT1 nuclear translocation, ↓BDNF, ↑NF-κB, ↑caspase-1, ↓mitochondrial oxygen flows, altered mitochondrial morphology by hypoxia after Aβ42 compared to Aβ42.<br>• These changes were contrasted by melatonin or naringenin. The use of EX-527 suggested partial and differential SIRT1 involvement in the observed effects between the two agents, which resulted greater for naringenin.	Aβ42 pre-treatment: 0.2 μM for 3 h.<br>Hypoxia: 3% O2 for 1 h followed by normoxia for 0 - 20 h.<br>Melatonin pre-treatment: 1 μM for 3 h.<br>Naringenin pre-treatment: 10 μM for 3 h.<br>EX-527 pre-treatment: 5 μM for 3 h and 15 min (always added 15 min prior the other drugs).
**[518]** Sha et al., 2025	Neurodegeneration/ Neuroinflammation (SCI)	Mechanical trauma or H2O2	N/A	Nrf2	Nrf2 inhibits GAPDH/Siah1 nuclear translocation, suppressing transcription of pro-inflammatory genes.<br>↓Expression of IL-6, TNFα, iNOS, and COX-2.<br>↓Microglial proliferation and ROS generation.<br>↑Expression of antioxidant genes HO-1 and NQO1.	*In vivo:* SCI model in adult rats.<br>H2O2 200 μM

(Continued)

10.3389/fimmu.2026.1778798

SUPPLEMENTARY TABLE 1 Continued

ARTICLE	MODEL	TREATMENT	DRUG TESTED	PATHWAY INVOLVED	MAIN RESULTS	NOTES
**[519]** Sancer et al., 2025	Toxicology	Abamectina (ABA), insecticide/ anthelmintic belonging to the avermectin family	N/A	MAPK/p38 → NF-κB. Bax/Bcl-2 e caspasi-3	• ↓Viability<br>• ↑Apoptosis induction<br>• ↑ROS<br>• ↑ytokine production, cell migration, and inflammatory response<br>•	ABA: 1 μg/mL-50 μg/mL
**[520]** Shehjar et al., 2025	Neuroinflammation	$FeSO_4$	deferoxamine<br>Novel cofilin inhibitor	NF-κB p65, ferritin (FTH/ FTL), DMT1	• DFX and CI:<br>• ↓Iron levels<br>• ↑cofilin expression<br>• ↑cell viability<br>• ↓pro-inflammatory cytokines (TNFα, IL-1β, IL-6; IL-12)<br>• ↓NF-κB p65	$FeSO_4$ (100–300 μM, 48 h)<br>- Deferoxamine (DFX, 150 μM)<br>- Novel cofilin inhibitor (CI, 5 μM) first-in-class cofilin inhibitor
**[521]** Zavala et al., 2025	Neuroinflammation	LPS	EVs derived from royal jelly	N/A	• LPS:<br>• ↓stiffness, ↑fluidity, and ↑migration<br>• RJEVs: ↑stiffness, ↓motility, and ↓inflammatory cytokine secretion.<br>•<br>•	N/A
**[522]** Zhang et al., 2025	Neuroinflammation	H_2O_2, LPS	ellorarxine (also DC645/NVG0645), a synthetic CNS-permeable retinoid, RARβ modulator	Cyp26b1 and RARβ (upregulation), NF-κB	• ↑LC3B-II, p62 modulation (autophagy regulation)<br>• ↓IL-6 (Ellorarxine pretreatment)<br>•	H2O2 (for MTT: 100 μM; for LDH: reported 200 mM)<br>LPS 10 μg/mL<br>ellorarxine pretreatment 10 nM for 4 h
**[523]** Distefano et al., 2025	Neuroinflammation (Ischemic injury)	Hypoxia–reoxygenation (H/R) to mimic ischemic injury exposure to:<br>– Cigarette smoke extract (CS)<br>– Heated Tobacco Product (HTP) aerosol extract	N/A	CS: induces strong oxidative stress, lipid peroxidation,<br>HTP: Nrf2/HO-1	• CS: ↑ROS, ↑proinflammatory cytokines (IL-1β, IL-6, TNFα).<br>HTP: ↓toxic profile, ↑ microglia activation<br>•	N/A
**[524]** Rahm et al., 2025	Transcriptomic study	baseline gene expression profiling by RNA-seq	N/A	N/A	• ↓markers expression (TREM2, CX3CR1, P2RY12)	N/A
**[525]** Araújo-Rodrigues et al., 2025	Neurodegeneration	LPS	Mushroom biomass digestive fractions, short-chain fatty acids, γ-aminobutyric acid (GABA)	Oxidative stress and ROS-related pathways<br>Neuroinflammation-associated signaling<br>Redox homeostasis	In HMC3 cells: ↓ ROS production and improved redox balance<br>In C. elegans: ↓Tau- and Aβ–induced neurotoxicity, ↑chemotaxis performance, delayed paralysis<br>• Effects were dose-dependent and species/ metabolite-specific	LPS: 100 ng/mL<br>*In vitro* model:<br>HMC3 cells<br>*In vivo* model:<br>Caenorhabditis elegans
**[526]** Chen et al., 2025	Neuroinflammation (Focal cerebral ischemia)	HDAC1 knockdown via siRNA<br>HDAC1 enzymatic reactivation using compound 5104434	HDAC1 siRNA<br>Compound 5104434 (HDAC1 activator)	HDAC1–NF-κB inflammatory signaling axis<br>MAP3K8/AP-1/SAT1	HDAC1 knockdown promotes ↑CD86<br>↑IL-1β, IL-6, TNF-α, ROS, LDH release and MMP activity<br>↑T-cell infiltration in ischemic brain tissue	*In vitro* model: HMC3 cells.<br>*In vivo* model: Mouse ischemic stroke model (MCAO/R: middle cerebral artery occlusion/reperfusion, OGD).

(Continued)

SUPPLEMENTARY TABLE 1 Continued

ARTICLE	MODEL	TREATMENT	DRUG TESTED	PATHWAY INVOLVED	MAIN RESULTS	NOTES
				pathway<br>STAT3 signaling (IFN-γ–dependent)<br>Oxidative stress–related pathways	HDAC1 deficiency sensitizes microglia to IFN-γ–induced inflammatory responses<br>• HDAC1 reactivation suppresses NF-κB signaling and improves functional recovery after stroke	
**[527]**<br>Eixarch et al., 2025	MS	H_2O_2	Cladribine.<br>It is a purine nucleoside analog used in MS therapy.	Cladribine ↓ ROS and preserves mitochondrial function.<br>↑ NF-κB, IL-1β, TNF-α, IL-6<br>↑Antioxidant gene expression (Nrf2, HO-1).	Cladribine ↓ inflammatory activation under oxidative stress in HMC3 cells.<br>SH-SY5Y neurons exhibit higher survival and lower oxidative damage.<br>• Cladribine does not impair normal cellular activity but exerts indirect neuroprotective effects.	H_2O_2: 200 μM<br>Cladribine: 0.05–1 μM<br>*In vitro* models: HMC3 cells<br>Primary human astrocytes<br>SH-SY5Y cells<br>Primary human neuronal progenitor cells
**[528]**<br>Khan et al., 2025	(GBM)<br>Cancer	N/A	Cell culture in 2D collagen-coated substrates versus 3D collagen-based bioactive matrices, followed by reseeding on collagen I–coated polyacrylamide gels	Cell–matrix interaction and mechanotransduction pathways<br>Cytoskeletal organization and focal adhesion dynamics<br>Biomechanical memory of the cellular microenvironment	GBM cells cultured in 2D exhibit ↑ traction stresses compared to cells previously cultured in 3D collagen matrices<br>3D bioactive matrices ↓ cellular traction forces, mimicking *in vivo* tumor mechanics<br>Significant cell line–dependent differences observed in single-cell stiffness and focal adhesion organization<br>• Microglia and GB cells display distinct biomechanical responses depending on prior 2D or 3D culture conditions	*In vitro*:<br>human glioblastoma cell lines (LN229, T98G)<br>HMC3 cells
**[529]**<br>Yanaizu et al., 2025	Transcription	Overexpression and knockdown of candidate transcription factors<br>Luciferase reporter assays using a 5 kb upstream region of TREM2	N/A	Transcriptional regulation of TREM2<br>ZEB2-dependent gene transcription<br>Zinc finger–mediated DNA binding	SPI1 (PU.1), MAFB, CEBPA, ZEB2 and SALL1 enhance TREM2 promoter activity<br>ZEB2 prioritized based on co-expression with TREM2<br>ZEB2 knockdown in HMC3 cells ↓ TREM2 mRNA and protein expression<br>ZEB2 transcriptional activity requires intact zinc finger domains<br>• ZEB2 interacts with multiple sites in the TREM2 upstream regulatory region	*In vitro* models:<br>HMC3 cells<br>HEK293T cells
**[530]**<br>Wang et al., 2025	Neuroinflammation	IFN-γ, IFN-α2b,<br>TNF-α, LPS, or LPS/IFN-γ<br>Stable knockdown of lncRNA BISPR (BISPR-KD)	N/A	BISPR–JAK–STAT1 signaling axis<br>Inflammatory response pathways<br>IL-6 regulation	↑BISPR induced by IFN-γ, IFN-α2b, and TNF-α<br>BISPR knockdown ↓ LPS- and LPS/IFN-γ–induced IL-6 production<br>Differentially expressed genes are enriched in inflammatory and JAK–STAT signaling pathways<br>• BISPR knockdown does not affect TLR4 pathway components but ↓ STAT1 (Tyr701) phosphorylation	LPS: 100 ng/mL<br>*In vitro* models:<br>HMC3 cells Knockdown of lncRNA BISPR using siRNA.

(Continued)

10.3389/fimmu.2026.1778798

10.3389/fimmu.2026.1778798

SUPPLEMENTARY TABLE 1 Continued

ARTICLE	MODEL	TREATMENT	DRUG TESTED	PATHWAY INVOLVED	MAIN RESULTS	NOTES
**[531]** Rahm et al., 2025	Comparative analysis with primary human microglia, iPSC-derived microglia, and astrocytes	N/A	N/A	Cell-type identity and transcriptional profiling<br>Gene expression signature analysis	Gene-pair ratio–based models using bulk RNA-seq and scRNA-seq data reveal heterogeneous cellular identity of HMC3<br>HMC3 cells show the highest similarity score to astrocytes rather than to microglia<br>Canonical microglial gene signatures are poorly represented in HMC3 cells<br>•	
**[532]** Kritika et al., 2025	Neuroinflammation and oxidative stress	LPS	Nobiletin (NOB)	TLR4/MyD88/NF-κB inflammatory signaling pathway<br>TLR10-mediated negative regulation of TLR4 signaling<br>Nrf2/HO-1 antioxidant pathway<br>Oxidative stress–related pathways	NOB attenuates LPS-induced cytotoxicity in HMC3 cells<br>↓IL-1β and IL-6 production<br>Inhibition of TLR4/MyD88/NF-κB pathway activation<br>↑TLR10 expression, acting as a negative regulator of inflammation<br>↑Nrf2 and HO-1 expression and antioxidant enzymes (CAT, GPx, SOD)<br>↓intracellular ROS levels<br>•	LPS stimulation (1 µg/mL) ± NOB (5–40 µM) for 24 h
**[533]** Altahrawi et al., 2025	Neuroinflammation	Polyinosinic–polycytidylic acid (poly I:C) stimulation	Polyinosinic–polycytidylic acid (poly I:C)	NLRP3 inflammasome signaling<br>NF-κB–mediated inflammatory pathway<br>Microglia–neuron inflammatory crosstalk<br>Apoptosis-related pathways	Poly(I:C) induces strong pro-inflammatory activation in HMC3 cells<br>↑TNF-α, IL-6, IL-1β, IL-8, IL-12, IL-18 and chemokines<br>↑NF-κB nuclear translocation in microglia<br>Conditioned medium from poly(I:C)-activated microglia induce ↑ Bax, Bad, cleaved caspase-3, cleaved PARP, AIF<br>Poly(I:C)-treated mice show ↑ hippocampal IL-6 and TNF-α expression<br>• Behavioral deficits, including impaired memory, reduced locomotion, and anxiety-like behavior	poly (I:C) (5, 10, 15, 20, 25, 50, 75, and 100 µg/mL) for 24 h. After 24 h.<br>CCK: 10 µl, for 1 h<br>*In vitro* model:<br>HMC3 cells; differentiated SH-SY5Y neuronal cells<br>*In vivo*: poly(I:C)-treated mice
**[534]** Garay-Mayol et al., 2025	Neuroinflammation	Gut microbiota–derived urolithins (Uro-A, Uro-B, IsoUro-A) and their phase II conjugates (glucuronides and sulphates)<br>LPS or TNF-α	Urolithin A, Urolithin B, IsoUrolithin A<br>Phase II conjugates (glucuronide and sulphate derivatives)	NF-κB–mediated inflammatory signaling<br>TLR4/MyD88-dependent pathway<br>BBB integrity and endothelial inflammatory responses	All urolithins and conjugates cross the BBB *in vitro*, with Uro-B and its sulphate showing highest permeability<br>Urolithins preserve BBB integrity against TNF-α–induced damage<br>In HMC3 cells, all urolithins ↓ IL-6 secretion under LPS stimulation<br>Only free urolithins ↓ IL-8 levels<br>All urolithins inhibit NF-κB nuclear translocation in LPS-treated microglia<br>• Uro-A selectively interferes with the MyD88-dependent arm of TLR4 signaling	*In vitro* models:<br>Human brain microvascular endothelial cells (HBMECs) as BBB model<br>HMC3 cells

(Continued)

SUPPLEMENTARY TABLE 1 Continued

ARTICLE	MODEL	TREATMENT	DRUG TESTED	PATHWAY INVOLVED	MAIN RESULTS	NOTES
**[535]** Li Y et al., 2025	Alzheimer's disease	Aβ-amyloid<br>RRBP1 knockdown via siRNA	PD98059 (ERK pathway inhibitor)	ERK pathway	RRBP1 inhibition ↓ M1 microglial markers (iNOS, TNF-α, IL-1β)<br>↑M2 marker ARG1<br>↑pERK/ERK ratio<br>• ↑cell viability and SOD and ↓ apoptosis and ROS ERK inhibition switches RRBP1-mediated anti-inflammatory and neuroprotective effects	*In vitro* models:<br>BV-2 and HMC3 cells
**[536]** Ceccarelli MC et al., 2025	Cancer (GBM)	Internalization of lipid-based magnetic nanovectors (LMNVs) by microglia<br>Alternating magnetic field (AMF) stimulation to induce magneto-thermal activation	LMNVs (lipid matrix doped with iron oxide nanoparticles)	Ca^{2+} signaling–dependent inflammatory activation<br>Magneto-thermal conversion → sustained intracellular Ca^{2+} increase	LMNVs high biocompatibility and efficient uptake by HMC3 cells<br>AMF-stimulated LMNVs ↑intracellular Ca^{2+}<br>Microglia polarized toward M1-like phenotype<br>↑CD40, CD86<br>↑IL-6, IL-8, TNF-α<br>Transcriptomic profile similar to IFN-γ–activated microglia<br>Conditioned medium ↓ GBM cell viability and proliferation<br>• GBM cell death associated with immunogenic cell death: ↑HMGB1 and calreticulin (CRT) exposure	IFN-γ used as positive control
**[537]** Kilic M et al., 2025	Ferroptosis	Cromolyn treatment in HMC3 cells	Cromolyn	Ferroptosis–ferritinophagy axis<br>GPX4-regulated ferroptosis<br>NCOA4-mediated ferritinophagy<br>Iron and lipid peroxidation pathways	Cromolyn induces time- and dose-dependent cytotoxicity (IC_{50} ≈ 9.4 μM at 48 h)<br>G0/G1 arrest and nuclear abnormalities at 48 h<br>↑MDA, ↑intracellular iron, ↓GSH<br>↓GPX4, ↑ACSL4, ↓SLC7A11<br>Activation of NCOA4-dependent ferritinophagy ↓ FTH1<br>• Ferroptosis/autophagy inhibitors and NCOA4 silencing rescue cell viability and reduce lipid peroxidation	Cromolyn treatment for 24, 48, and 72 h
**[538]** Ceccarelli MC et al., 2025	Neuroinflammation	IFN-γ stimulation to induce M1 polarization<br>Co-treatment with Polydopamine nanoparticles	PDNPs	Microglial M1 polarization and oxidative stress pathways<br>-ROS production<br>-Pro-inflammatory cytokine signaling	PDNPs show high biocompatibility with minimal cytotoxicity<br>Efficient microglial uptake; preferential lysosomal localization<br>IFN-γ induces ↑ROS, ↑CD40/CD86, ↑ IL-6, IL-8, TNF-α<br>PDNPs ↓oxidative stress<br>Suppress M1 surface markers (CD40, CD86)<br>↓pro-inflammatory cytokine release	

(Continued)

10.3389/fimmu.2026.1778798

SUPPLEMENTARY TABLE 1 Continued

ARTICLE	MODEL	TREATMENT	DRUG TESTED	PATHWAY INVOLVED	MAIN RESULTS	NOTES
					• Prevent microglial shift toward M1 phenotype	
**[539]**<br>Liu D et al., 2025	Neuroinflammation (SCI)	LPS	Sevoflurane	USP11/UHRF1<br>-De-ubiquitination pathway<br>- BCL2/BAX<br>-Inflammatory and oxidative stress pathways	Sevo improves functional recovery, ↑ BBB scores and ↓neuronal death,↓Inflammatory cytokines and oxidative damage *in vivo*<br>Suppression LPS-induced HMC3 apoptosis, ↓IL-1β, IL-6, ↓ROS, MDA, ↑GSH<br>↓USP11 and UHRF1 expression in activated microglia<br>USP11-UHRF1 interaction via deubiquitination<br>• Sevo disrupts USP11/UHRF1 interaction, promoting UHRF1 degradation	
**[540]**<br>Bonaccorso A et al., 2025	Intranasal brain delivery	Design and optimization of RSV nanocrystals via Quality by Design (QbD)<br>Surface functionalization with poly-L-arginine hydrochloride	Resveratrol nanocrystals (RSV NCs), naked and poly-L-arginine–functionalized	ROS scavenging activity<br>Mucoadhesion and intranasal transport mechanisms	RSV NCs formed truncated cubic crystals (~240 nm) with ~80% drug content<br>Poly-L-arginine functionalization yields positively charged, mucoadhesive nanosuspension<br>Nanonization ↑aqueous solubility without altering antioxidant activity<br>Both formulations show stability in artificial CSF and during storage<br>• All treatments are biocompatible and non-toxic in HMC3 cells	
**[541]**<br>Li X et al., 2025	Cancer (GBM)	CMTM6 knockdown Microglia/ macrophage-specific Cmtm6 deletion *in vivo*	N/A	CMTM6–PD-L1 immune checkpoint axis	↑CMTM6 expression with glioma grade and in M2-like GAMs<br>CMTM6 co-localization with CD68, IBA1, TMEM119 in GBM tissue<br>CMTM6 knockdown in HMC3 cells ↓PD-L1 (CD274) and TGFβ isoforms<br>↑IL-6 and CCL3, indicating M1-like shift<br>• CMTM6 deletion suppresses tumor growth and prolongs survival in mice	*In vitro* model: CMTM6 knockdown in HMC3 cells<br>*In vivo* model: Microglia/macrophage-specific Cmtm6 deletion
**[542]**<br>Martinez-Orellana P et al., 2025	Viral infection (SARS-CoV-2)	SARS-CoV-2 infection	cGAS–STING pathway antagonist	cGAS–STING DNA-sensing pathway<br>IFN-β signaling<br>Pro-inflammatory cytokine and chemokine release	Astrocytes are highly permissive to SARS-CoV-2 infection; HMC3 show minimal viral replication<br>Astrocytes↑ IFN-β response<br>Both glial cell types produce chemoattractants<br>Infection ↑ glial senescence and cGAS–STING pathway<br>Infected glia cause loss of synaptic connectivity and ↓neuronal electrical activity	*In vitro* models:<br>glial cells; neuronal cultures exposed to infected glia

(Continued)

10.3389/fimmu.2026.1778798

SUPPLEMENTARY TABLE 1 Continued

ARTICLE	MODEL	TREATMENT	DRUG TESTED	PATHWAY INVOLVED	MAIN RESULTS	NOTES
					↑Pro-inflammatory cytokines/chemokines and DNA damage foci in neuronal cultures<br>• cGAS–STING inhibition partially rescues neuronal electrical activity early post-infection	
**[543]** Inostroza-Nieves Y et al., 2025	Oxidative stress	ET-1 stimulation of microglia	BQ788 (selective ETRB antagonist)<br>BQ123 (ETRA antagonist)	ET-1/ETRB–STAT1 signaling axis	ET-1 ↑NO, ROS and ↑iNOS mRNA<br>↑TNF-α and ↑IL-6<br>ET-1 induces STAT1 phosphorylation<br>BQ788 blocks proinflammatory and oxidative responses<br>• EAE brains ↑Edn1, ↑Ednrb expression and ↑ET-1	
**[544]** Su Y et al., 2025	Neuroinflammation (SCI)	Urolithin B (UB) treatment Oral gavage of UB	UB<br>BMS-986299 (NLRP3 agonist)	NLRP3/Caspase-1/IL-1β	UB protects PC12 cells from oxidative stress–induced injury<br>UB suppresses LPS-induced M1 polarization of HMC3<br>UB improves motor function recovery<br>↓M1 microglia, ↑M2 polarization<br>↓Glial scar formation and enhanced nerve regeneration<br>UB inhibits NLRP3, Caspase-1 activation, and IL-1β production *in vivo* and *in vitro*<br>• BMS-986299 reverses UB-mediated therapeutic effects	*In vitro* model:<br>PC12 neuronal cells<br>*In vivo* model:<br>SCI mice
**[545]** Yang H et al., 2025	Ferroptosis	Silver nanoparticles (AgNPs)	Silver nanoparticles (AgNPs)<br>COG1410 (TREM2 agonist, 5 μg/mL)	Ferroptosis–inflammation crosstalk	AgNPs induce dose-dependent cytotoxicity in HMC3 cells<br>Mitochondrial ultrastructural and functional damage observed<br>↑Intracellular Fe^{2+}, ↑ROS, ↑lipid peroxidation, ↓GSH<br>Dysregulation of ferroptosis-related proteins<br>↓TREM2 with ↑pro-inflammatory and ↓anti-inflammatory markers<br>COG1410 restores inflammatory balance via TREM2<br>• COG1410 suppresses AgNPs-induced ferroptosis	Exposure to silver nanoparticles (AgNPs) at 0, 50, 100, and 200 μg/mL for 48 h
**[546]** Hartmann C et al., 2025	ALS	Induction of progerin to trigger premature cellular aging	N/A	SASP activation<br>DNA damage response<br>Stress response and nucleocytoplasmic transport	Progerin-expressing microglia show activation of SASP and ↑DNA damage<br>↓responsiveness to LPS stimulation<br>↓migration and ↓phagocytosis<br>Transcriptomic shifts consistent with aging and neurodegeneration signatures	

(Continued)

10.3389/fimmu.2026.1778798

10.3389/fimmu.2026.1778798

SUPPLEMENTARY TABLE 1 Continued

ARTICLE	MODEL	TREATMENT	DRUG TESTED	PATHWAY INVOLVED	MAIN RESULTS	NOTES
					Defective stress responses and impaired nucleocytoplasmic transport<br>• Mislocalization of ALS-associated protein FUS	
**[547]** Skóra B et al., 2025	Disease-associated microglial signature (DAM)	Exposure to small-size silver nanoparticles	Silver nanoparticles (AgNPs)	ROS–TLR4–NF-κB inflammatory axis	AgNPs induce dose- and time-dependent cytotoxicity in both HMC3 and SH-SY5Y cells<br>In co-culture, AgNPs ↑ROS-dependent microglial inflammation<br>Activation of TLR4 and NF-κB signaling<br>Upregulation of ICAM1, ITGAX, ApoE, B2M<br>↑NO production over time<br>↓SYN1 and ↓SNAP-25<br>• ↑CAT, ↑SOD, ↑TRAF6, ↑TLR4 in neurons	Exposure to small-size silver nanoparticles (dose- and time-dependent)<br>AgNPs:1 µg/mL for 24 h and 48 h
**[548]** Yang S et al., 2025	Environmental chemical exposure	LPS stimulation<br>IFN-γ<br>Catecholamines<br>Nine environmental chemicals (pesticides such as picoxystrobin)	HTRF-based cytokine assay	Microglial inflammatory signaling	hiMG cells exhibit cytokine response profiles most similar to *in vivo* microglia<br>LPS induces ↑IL-6 and ↑TNF-α in hiMG cells<br>↑IL-6 and TNF-α assay<br>EC_{50} ≈ 50 ng/mL (IL-6) and ≈ 90 ng/mL (TNF-α) for LPS<br>All nine environmental chemicals tested ↑IL-6 and TNF-α<br>• Picoxystrobin ↑IL-6 and TNF-α	
**[549]** Wang S et al., 2025	Encephalitis	Mendelian randomization + colocalization<br>Anti-NMDAR-IgG stimulation/ injection for functional validation in different models.	Candidate proteins: SIRPA, LGALS3, CASP3, TREM2, IL1RN	Microglial activation<br>Caspase-mediated apoptosis<br>Immune checkpoint and innate immunity signaling	37 genetically predicted proteins associated with NMDAR-E risk<br>Five prioritized causal proteins: SIRPA, LGALS3, CASP3, TREM2, IL1RN<br>Anti-NMDAR-IgG induces protein expression changes in HMC3 cells<br>Overlapping antibody-responsive proteins in microglia and mouse brain tissue<br>Enriched expression in $CD20^+$ B cells, mast cells, and $CD16^+$ monocytes in PBMCs<br>• PPI and druggability analyses highlight translational relevance	In silico:<br>• Proteome-wide MR using pQTLs from 7 GWASs<br>• NMDAR-E GWAS meta-analysis<br>*In vitro*:<br>• Human microglial HMC3 cells stimulated with patient-derived anti-NMDAR-IgG<br>*In vivo*:<br>• Passive immunization mouse model (ICV anti-NMDAR-IgG)
**[550]** Xu L et al., 2025	Cerebral ischemia-reperfusion injury	HMC3 Microglia-specific RHBDF2 knockdown<br>OGD/R injury induction	N/A	RHBDF2–STING–TBK1–IRF3/NF-κB signaling axis	RHBDF2 expression is upregulated in MCAO/R mouse brains and OGD/R-treated HMC3 cells<br>Microglial RHBDF2 knockdown ↓ cerebral infarct volume and improves neurological	*In vitro* model:<br>HMC3 cells<br>*In vivo* model:<br>MCAO/R mice

(Continued)

SUPPLEMENTARY TABLE 1 Continued

ARTICLE	MODEL	TREATMENT	DRUG TESTED	PATHWAY INVOLVED	MAIN RESULTS	NOTES
					outcomes<br>Suppresses M1 polarization and promotes M2 polarization in ischemic penumbra<br>RHBDF2 interacts with STING and activates STING–TBK1–IRF3/p65 signaling<br>Knockdown of RHBDF2 inhibits STING–TBK1 and neuroinflammation<br>↑ m6A methylation of RHBDF2 mRNA during CIRI<br>• m6A reader YTHDF1 enhances RHBDF2 expression by recognizing m6A-modified transcripts	
**[551]**<br>Tong J et al., 2025	Diabetic retinopathy	Intravitreal injection of MSC-derived sEVs<br>miR-29a-3p silencing or overexpression in sEVs	Mesenchymal stem cell–derived small extracellular vesicles (MSC-sEVs) carrying miR-29a-3p	miR-29a-3p/HMGB1/TLR4 signaling axis	MSC-sEVs ↓retinal inflammation and vascular leakage in diabetic rats<br>Suppress M1-like polarization *in vivo* and *in vitro*<br>miR-29a-3p identified as a key effector miRNA in MSC-sEVs<br>miR-29a-3p directly targets and ↓HMGB1<br>Silencing miR-29a-3p abolishes therapeutic effects of MSC-sEVs<br>• miR-29a-3p overexpression suppresses M1 polarization reversed by HMGB1 overexpression	*In vitro* model:<br>HMC3 cells<br>*In vivo* model:<br>diabetic rats
**[552]**<br>Zhang H et al., 2026	Bisphenol S (BPS) toxicity	Exposure to BPS with<br><br>hydroxytyrosol (HT)	BPS<br>(HT; antioxidant polyphenol)	CYP1A1/CYP1A2/CYP2B6 signaling<br>ROS-mediated microglial activation<br>Pro-inflammatory polarization	Computational toxicology predicts significant neurotoxic potential of BPS<br>BPS ↓viability of BV2 and HMC3 cells and ↑ROS<br>BPS induces microglial shift toward a pro-inflammatory phenotype<br>CYP1A is key mediators of BPS toxicity<br>• HT reverses BPS-induced cytotoxicity, oxidative stress, and inflammatory activation	*In vitro* models:<br>BV2 and HMC3 cells<br>*In vivo* model:<br>murine model of BPS-induced neurotoxicity
**[553]**<br>Shukla S et al., 2026	Alzheimer's disease	Stable expression of mutant APP (APPswe/F/L: Swedish, Florida, London mutations)	N/A	Mitochondrial Ca^{2+} homeostasis<br>mtCU (mitochondrial calcium uniporter)<br>mCa^{2+} flux regulation<br>Mitochondrial permeability transition pore (mPTP)<br>Bioenergetic dysfunction	Mutant APP induces mitochondrial dysfunction:<br>↑vulnerability in SHSY5Y cells: mCa^{2+} overload, ATP depletion, ↓OCR, ↑cell death<br>↑mtCU expression, ↑ mCa^{2+} uptake at high [Ca^{2+}], ↑mitochondrial Ca^{2+} retention capacity (mito-CRC) in glial-like cells<br>SH-SY5Y cells unveil faster mCa^{2+} uptake at low [Ca^{2+}]<br>All cell types display ↓membrane potential,	*In vitro* models:<br>SHSY5Y, HMC3 and SVGp12 (astrocytic-like cells)

(Continued)

10.3389/fimmu.2026.1778798

10.3389/fimmu.2026.1778798

SUPPLEMENTARY TABLE 1 Continued

ARTICLE	MODEL	TREATMENT	DRUG TESTED	PATHWAY INVOLVED	MAIN RESULTS	NOTES
					impaired respiration, ↑ROS, and mitochondrial structural damage	
**[554]**<br>Wang Z et al., 2026	Neuroinflammation	Overexpression of Brucella BvrR throught transfection with pcDNA3.1-BvrR-His and AAV2/9-IBA-1-BvrR *in vivo* injection	IXA4 (IRE1 activator)<br>GSK2850163 (IRE1 inhibitor)	ER stress–IRE1–ATF2/ NF-κB p65 signaling axis	BvrR induces ER expansion and phosphorylation of IRE1 in HMC3 cells<br>IRE1 activation enhances phosphorylation and nuclear translocation of ATF2 and NF-κB p65<br>↑IL-6 and ↑TNF-α<br>IRE1 inhibition suppresses BvrR-induced inflammatory signaling<br>• IRE1 activation mimics the proinflammatory effects of BvrR	*In vitro* model:<br>HMC3 cells<br>*In vivo* model:<br>Mouse injection of AAV2/9-IBA-1-BvrR
**[555]**<br>Yao D et al., 2026	Neuroinflammation	Developmental and cellular exposure to Nonylphenol (NP)<br>Genetic knockdown of GPER	GPER knockdown (GPER-KD)<br>STAT3 activation	GPER–EGFR–STAT3 signaling axis	NP exposure causes learning and memory deficits and hippocampal neuronal damage in rats<br>NP induces microglial activation with M1 polarization and pro-inflammatory cytokine shift *in vivo*<br>GPER is a key NP-responsive target<br>NP activates GPER and downstream EGFR–STAT3 in hippocampus and HMC3 cells<br>Conditioned medium from NP-exposed microglia induces severe neuronal damage in SHSY5Y cells<br>GPER knockdown suppresses microglial activation, M1 polarization, and cytokine release, reducing neuronal injury<br>STAT3 activation reverses the protective effects of GPER knockdown<br>•	*In vitro* model:<br>SHSY5Y, HMC3 cells and co-culture system<br>*In vivo* model:<br>NP exposure in rats
**[556]**<br>Chen PQ et al., 2026	Obesity	High-fat diet feeding<br>PA stimulation	CTRP9 knockdown<br>CTRP9 overexpression	CTRP9–lipophagy–PI3K/ AKT/FOXO1 axis	HFD mice exhibit cognitive impairment and ↑hippocampal microglial lipid droplets<br>Autophagic activity is inhibited and CTRP9 expression ↓in obese mice<br>PA induces ↑lipid droplet and lipophagy impairment in BV2 and HMC3 cells<br>PA ↓CTRP9<br>CTRP9 knockdown exacerbates lipophagy dysfunction and lipid accumulation<br>• CTRP9 overexpression restores PI3K/ AKT/FOXO1 and improves lipophagy	*In vitro* model:<br>BV2 and HMC3 cells<br>*In vivo* model:<br>HFD mice

(Continued)

SUPPLEMENTARY TABLE 1 Continued

ARTICLE	MODEL	TREATMENT	DRUG TESTED	PATHWAY INVOLVED	MAIN RESULTS	NOTES
**[557]**<br>Qian Y et al., 2026	Ischemic stroke	Microglia-specific deletion of Tia1<br>TIA1 knockdown or overexpression in HMC3 cells	TIA1 (stress granule component)	Stress granule–IGF2 signaling axis	TIA1 is markedly upregulated during the acute phase of IS<br>TIA1 deletion ↓infarct volume, neuronal death, and motor deficits in IS mice<br>Suppresses microglial pro-inflammatory responses and enhances anti-inflammatory responses<br>Promotes phagocytic clearance of infarct debris<br>TIA1 enhances pro-inflammatory activation and neuronal toxicity after OGD<br>TIA1 deletion impairs SG formation and ↓ Igf2<br>• ↑IGF2 enhances microglial phagocytosis and anti-inflammatory responses	*In vitro* model:<br>HMC3 cells<br>*In vivo* model:<br>IS mice
**[558]**<br>Kang M et al., 2026	Inflammation	Structure-guided mutagenesis of hIL-1Ra in HMC3 and cortical neurons<br>Acute administration of optimized variants in Nlrp3 D301N knock-in mice	Engineered hIL-1Ra variants (E127Q)	IL-1R antagonism and neuroinflammatory signaling	Molecular simulations predict improved receptor binding (ΔΔG ≈ −7.8 kcal/mol vs WT)<br>All six variants suppress IL-1β and IL-6<br>E127Q induces anti-inflammatory effects<br>E127Q inhibits IL-1β–induced NMDAR hyperactivation in neurons at lower doses<br>• In Nlrp3 D301N mice, E127Q normalizes elevated NMDAR activity in medial prefrontal cortex	
**[559]**<br>Chu CT et al., 2026	Alzheimer's disease	Pharmacological activation of NRF2 in<br>HMC3 cells stimulated with IFN-γ or Aβ	CDDO-Im (NRF2 activator)	NRF2-dependent cytoprotective signaling	CDDO-Im–mediated NRF2 activation suppresses inflammatory responses in HMC3 cells induced by IFN-γ or Aβ<br>Integrative RNA-seq and ChIP-seq identify direct NRF2 target genes as IL6, CDK6 and autophagy (TFE3, SQSTM1)<br>NRF2 ↑ autophagic flux, ↑ LC3-II/LC3-I<br>• Single-cell transcriptomic analyses highlight microglia as key mediators of NRF2-driven autophagy regulation in AD brains	*In vitro* models:<br>HMC3 cells<br>In silico:<br>public single-cell transcriptomic datasets from AD brains
**[560]**<br>Xie L et al., 2026	Neurosyphilis	p47 stimulation of HMC3 cells<br>TNFAIP3 knockdown (siRNA)<br>Neutralization of Tp47 or IL6	Tp47, IL6, TNFAIP3	TNFAIP3–SOCS3–IL6 inflammatory axis	Tp47 enhances HMC3 migration toward endothelial cells<br>Tp47-conditioned microglia promote endothelial tube formation<br>RNA-seq and cytokine profiling identify IL6 and TNFAIP3 as central mediators<br>Tp47 upregulates TNFAIP3, suppressing SOCS3 and ↑ IL6	*In vitro* models:<br>HMC3 and human umbilical vein endothelial cells (HUVECs)

(Continued)

10.3389/fimmu.2026.1778798

SUPPLEMENTARY TABLE 1 Continued

ARTICLE	MODEL	TREATMENT	DRUG TESTED	PATHWAY INVOLVED	MAIN RESULTS	NOTES
					TNFAIP3 knockdown restores SOCS3, ↓IL6, and attenuates angiogenesis<br>Neutralizing Tp47 or IL6 abolishes angiogenic effects<br>• Recombinant IL6 alone recapitulates endothelial network formation	
**[561]** Zhu H et al., 2026	TBI	CX3CR1 overexpression or knockdown<br>Pharmacological modulation of MAPK signaling<br>Intranasal recombinant CX3CL1	Recombinant CX3CL1 (r-CX3CL1) MAPK pathway modulators	CX3CL1–CX3CR1–MAPK signaling axis	CX3CR1 ↑after oxidative stress and TBI<br>CX3CL1 is mainly expressed by neurons and astrocytes, while CX3CR1 localizes to microglia<br>CX3CR1 overexpression promotes M2 polarization and suppresses inflammatory cytokine release<br>CX3CR1 knockdown enhances M1 polarization and inflammation<br>Effects are mediated via inhibition of p38 MAPK and activation of ERK1/2<br>Intranasal r-CX3CL1 improves microglial polarization and neurological recovery in TBI mice<br>• Therapeutic effects are abolished in CX3CR1 knockout mice	*In vitro* model:<br>HMC3 cells<br>*In vivo* model:<br>Mouse<br>TBI model
**[562]** Shen X et al., 2026	MDD	Measurement of peripheral cytokines<br>3-hydroxykynurenine (3-HK)	Key molecules: cytokines (IL-1α, IL-1β, IL-6, IL-17A, IL-18, TNF-α, IFN-γ, G-CSF, M-CSF); 3-HK	Kynurenine pathway–mitochondrial pyroptosis axis	Adolescents with MDD show peripheral IL-1α levels<br>IL-1β, IL-6, IL-17A, IL-18 and ¯TNF-α, IFN-γ<br>¯G-CSF and M-CSF levels in MDD patients<br>3-HK ¯microglial viability in a dose-dependent manner<br>3-HK induces microglial pyroptosis via a mitochondrial-dependent pathway<br>• Pyroptotic microglia release IL-1β	*In vitro* model:<br>HMC3 cells<br>Clinical cohort:<br>• 60 adolescents with MDD<br>• 25 healthy controls

10.3389/fimmu.2026.1778798
